# Determinants of instructional leadership: A survey of public secondary schools

**DOI:** 10.1371/journal.pone.0355674

**Published:** 2026-08-14

**Authors:** Suman Pande, Laxman Acharya, Prakash C. Bhattarai

**Affiliations:** Department of Development Education, Kathmandu University School of Education, Hattiban, Lalitpur, Nepal; Woldia University, ETHIOPIA

## Abstract

Instructional leadership is central to teaching and learning processes and is undertaken by academic leaders to promote higher levels of student learning. Despite its recognized importance, students’ academic performance in public schools in Nepal remains persistently low, raising concerns about how instructional leadership is practiced in the schools. There is a need for a validated model to implement the required instructional leadership practices in schools. The existing instructional leadership models are primarily Western and not entirely suitable for implementation in Nepal. Therefore, it seemed essential to develop a model by identifying the determinants of instructional leadership in Nepal’s local context. Kathmandu, home to the country’s largest concentration of public schools, provides a critical context for examining instructional leadership practices. Given this, the present study examined the determinants of instructional leadership in Kathmandu, Nepal. The study employed a quantitative, non-experimental, cross-sectional research design to examine those factors. The framework for this study is grounded in Hallinger and Murphy’s (1985) model of instructional leadership, which serves as the conceptual basis. Using the Classical Delphi method, the model was adapted to develop a measurement scale. At the same time, the study was situated within a post-positivist paradigm, considering that the reality of instructional leadership exists independently. Exploratory factor analysis was then conducted to extract the underlying factors. The findings reveal three major factors influencing instructional leadership: (a) planning, managing, and supervising instructional programs; (b) motivating and developing the competence of teachers and students; and (c) involving parents in the learning process. The inclusion of parental involvement emerged as a novel dimension in Nepal. These results provide meaningful insights for school head teachers, other educational stakeholders, government policymakers, and international researchers interested in instructional leadership.

## Introduction

The role of instructional leadership is vital in schools, as it significantly influences students’ academic achievement [1–3]. In schools in Nepal, the headteacher is primarily the educational leader, serving as the instructional leader as well. In this regard, head teachers are considered accountable for students’ academic achievement. In Nepal, policies to strengthen the role of the head teacher as an effective leader were enacted in the Education Rule 2002 [4]. Although some instructional leadership roles were defined, they were embedded within broader administrative duties and not explicitly recognized as distinct instructional leadership responsibilities. The other policies formulated for the development of school education are Three Year Interim Plan (2007/08-2009/10) [5] and other sequential Three-Year Interim Plans, School Sector Reform Plan (SSRP) (2009/10-2015/16) [6] School Sector Development Plan (SSDP) (2016/17-2022/23) [7], National Education Policy, 2076 B.S. [8], School Education Sector Plan (SESP) (2022/23-2031/32) [9]. In the policy documents, policymakers have emphasized the role of head teachers’ leadership in promoting students’ academic progress. However, the instructional leadership role, as the duty of the head teacher, has been mentioned clearly only in SSDP (2016/17-2022/23) [7]. This work by the Nepal Government demonstrates that the Ministry of Education has consistently worked to enhance Nepal’s educational system. Still, the concept of instructional leadership was introduced in the policy document only in 2016.

Despite several efforts by the Nepal Government to improve school education, public schools in Nepal have not achieved satisfactory results for a long time. A review of School Level Certificate (SLC) exam results in Nepal from 1994 to 2013 revealed that the average pass percentage was only 45.50% over those 20 years [10]. After replacing the SLC with Secondary Education Examination (SEE) and reforming the existing assessment system by changing the numerical grading system to a letter system under the Education Act 2016, the results have become even worse. The SEE results from recent years have shown a steady decline in public schools [11]. The SEE, evaluated using a letter grading system, is not satisfactory in Nepal [12]. The study of SEE result data showed that only 45% of students were above the acceptable level in the academic years 2018 and 2019; however, it reached 78% in the academic year 2020, due to the use of student scores from schools rather than center-based exams in the framework of Covid-19 [13]. In 2021, the SEE pass percentage increased as the board issued grade sheets based on internal evaluations conducted by individual schools in response to the pandemic [14]. In the subsequent years, the results reverted to a similar trend, with only 47.86% of students passing the SEE exam in 2024 [15].

The persistently unsatisfactory performance of schools is reflected in the SLC (School Leaving Certificate) results before the amendments to the Education Act 2016 and in the SEE (Secondary Education Examination) results that followed its implementation [11,12,16–22]. The unsatisfactory performance of public schools in Nepal is a blatant indication of the head teachers’ inadequate effort in the teaching and learning process in these schools. This responsibility falls under their instructional leadership role. In this regard, there is an urgent need to identify the factors that influence head teachers’ instructional leadership. Although there is some literature on instructional leadership, no study has been found that examines the factors influencing the instructional leadership of head teachers in the local context. Therefore, it was deemed imperative to identify the factors influencing head teachers’ instructional leadership. Accordingly, the research question guiding this study is: What factors determine head teachers’ instructional leadership? Identifying these factors helps head teachers recognize areas where they may be falling short and where improvement is needed. This research is expected to support head teachers in developing their professional capacities as effective instructional leaders, thereby enhancing students’ achievement in schools. Furthermore, the findings of this study can contribute to the existing body of knowledge on instructional leadership.

## Reviewing the literature

Instructional leadership is one of the leadership approaches that has gained steadily increasing importance within the overall role of school principals over the past six decades [23]. This growing emphasis arises from evidence demonstrating its substantial impact on student achievement. For instance, a meta-analysis by [24] found that the average effect of instructional leadership on student outcomes was three to four times greater than that of transformational leadership. A few years later, a study of elementary schools in the USA [25] yielded a similar result, showing that instructional leadership accounted for more variance in student outcomes than transformational leadership. Similarly, a study of Malaysian secondary schools by [26] found a positive correlation between instructional leadership and students’ academic achievement, suggesting that the instructional leadership role is crucial for achieving better educational outcomes in schools.

Similarly, a meta-analysis conducted in 2017 that included 14 studies from the past decade [27] found a positive effect of instructional leadership on students’ academic outcomes, along with transformational leadership and distributed leadership. This study confirmed the argument that the more leaders engage in the business of teaching and learning with their students, the better the students’ academic performance. Similarly, a quantitative study in Indonesia by [28] found that instructional leadership is efficacious in improving learning quality. In line with previous findings, a recent meta-analysis conducted in 2025 by [29] revealed that the relationships between principal leadership and student achievement have been more frequently investigated through supportive, instructional, distributed, and transformational leadership approaches (23.64%, 21.82%, and 20.00%), respectively. These findings suggest that the relationship between educational leadership and student achievement remains one of the most widely researched topics, with instructional leadership being the most studied leadership approach. Moreover, more than half of the studies included in the meta-analysis were conducted in the USA, indicating that such research is predominantly undertaken in that context. Further evidence of the prominence of instructional leadership research in the United States is demonstrated by the studies included in Hallinger’s [30] review, which identified 130 doctoral dissertations employing the Hallinger and Murphy instructional leadership model (PIMRS), produced by doctoral students from more than 85 American universities.

The literature on instructional leadership indicates that it has become an important area of educational leadership in broader international contexts. A review study on instructional leadership by [31] examined conceptual and topical trends in instructional leadership research, demonstrating the development of a knowledge base and research trends in this field. The contemporary studies have found the effect of instructional leadership on school organizational climate [32], school culture [33], teacher teaching performance [33], teacher professional learning [34], teacher instructional practices [34,35], teacher autonomy, teacher collective innovativeness [35], and teacher professional development [36]. This shows that the recent literature illustrates the importance of instructional leadership in strengthening school- and teacher-related outcomes. Some studies have also examined the relational process through which instructional leadership affects school- and teacher-related outcomes. For example [33], found that school culture mediated the direct and indirect effects of instructional leadership on teachers’ teaching performance [34] found the influence of teacher professional learning on the relationship between instructional leadership and teachers’ instructional practices, while [35] identified teacher autonomy and teacher collective innovativeness as serial mediators of this relationship. Such a development in the study trend shows a gradual shift in instructional leadership research from simple direct-effect models to more complex relational effects.

### Conceptualizing instructional leadership

The instructional leadership has been defined in various ways. According to [37], pioneering scholars who first articulated a clear understanding of instructional leadership, focusing on U.S. schools in the 1980s and 1990s, identified the principal’s role in aligning school goals, instructional practices, and student outcomes through both direct and indirect management activities. Their framework has been applied internationally. It involves defining the school’s mission, managing the instructional program, and promoting a favorable learning climate to enhance teaching and learning. This framework illustrates that instructional leaders influence classroom practices not only through direct supervision and feedback but also by developing and enforcing school-wide policies, expectations, and structures that guide teacher and student behavior toward common educational goals. However, the central concept of instructional leadership is the leadership role focused on teaching and learning activities to enhance students’ academic achievement. Various educators have interpreted this concept in different ways, each from their own perspective. A Canadian researcher [38] portrayed instructional leadership as an active role in classroom practice, grounded in high levels of pedagogical expertise. Similarly, a researcher from Malaysia [26] defined it as leadership focused on teaching and learning activities that enhance interactions between teachers and students centered on the curriculum. Likewise, researchers from the UK [39] emphasized the importance of setting clear educational objectives, planning the curriculum, and evaluating teachers and their educational practices.

Among all researchers, those from America have made significant contributions to the study of instructional leadership. They have provided various interpretations of how effective schools develop through instructional leadership. For example, the American educationist Lee Shulman, who observed schools in the late 1960s and early 1970s, argued that while general principles of effective teaching are essential, they should not be treated as final measures, as they may not hold in particular circumstances [40]. He emphasized that effective teaching requires teachers’ more specific professional knowledge, such as “wisdom of practices”, and “intersection of content and pedagogy”. Similarly, Grissom, Loeb, and Master, who examined the relationship between principals’ time spent in instructional leadership behaviors and student achievement gains in schools, asserted that the effect of instructional leadership does not depend on time spent engaging in instruction, but on the type and quality of those time investments [41]. Likewise, Hallinger and Heck, who reviewed research from 1980 to 1995 on the impact of principals on school effectiveness and student achievement, identified several pathways through which principals influence learning outcomes, including school goals, school structure, social network, people, and organizational culture [42].

Nepali researchers have also studied instructional leadership from various perspectives and provided their own interpretations. In Nepal, although the instructional leadership role of head teachers has been incorporated into the School Sector Development Plan (2016/17-2022/23) [7], research on instructional leadership began to appear in the literature only after 2020. This suggests that in Nepal, researchers’ focus on this concept has primarily emerged since around 2020. In Nepal, a principal of a public school is referred to as a head teacher. Danai defined instructional leadership as the behavior of head teachers that guides a school in educating all students to achieve high academic standards [43]. Lamsal conceptualized instructional leadership as the position within an educational institution that utilizes individual expertise and knowledge to ensure students’ academic performance [44]. Dahal described the head teacher’s role in coordinating instructors and parents, with a clear focus on achieving their shared objectives, as an instructional leadership role [45]. Drawing on the existing literature, we developed our understanding of instructional leadership as a process in which the head teacher provides support and guidance to the team by managing curriculum and instruction to improve students’ learning outcomes.

The “Australian Effective Schools Project,” which studied school effectiveness by viewing student achievement as one of several key outcomes, identified school leadership as a vital element of effective schools [46]. Based on the literature, it can be argued that among the various forms of leadership, instructional leadership is even more important for effective schools. Instructional leadership promotes student outcomes by positively influencing the factors that affect the teaching-learning process. A recent empirical study conducted in Nigeria demonstrated that instructional leadership significantly influences teachers’ professional development and growth [36]. Similarly, a study on the effectiveness of instructional leadership in public secondary schools in Buea, employing a non-experimental descriptive-correlational research design, showed significant relationships between teachers’ perceptions of principals’ instructional leadership and key dimensions such as defining the school mission, managing the instructional program, and developing the school learning climate [47]. These findings suggest that instructional leadership positively influences key factors in teaching and learning, thereby enhancing students’ learning achievement.

### Global perspectives on instructional leadership

Instructional leadership is found to be influenced by country context. In some countries, particularly those with developed educational systems, it has been practiced more effectively. In a paper, Hallinger illustrated the evolution of research on school leadership over the past three decades and cited the assertions of Edmonds and Bossert, along with his co-authors, that “strong instructional leadership from the principal” is a hallmark of effective urban elementary schools in the USA [48]. Similarly, a meta-analysis of 27 studies published between 1978 and 2006 revealed that the majority of instructional leadership research had been conducted in the USA, with two studies in Canada and one each in Australia, England, Hong Kong, Israel, the Netherlands, New Zealand, and Singapore [24]. This meta-analysis identified five core dimensions of instructional leadership: (i) establishing goals and expectations, (ii) resourcing strategically, (iii) planning, coordinating, and evaluating teaching and the curriculum, (iv) promoting and participating in teacher learning and development, and (v) ensuring an orderly and supportive environment. These findings demonstrate the effective implementation of instructional leadership practices in these school contexts. On the contrary, in a study incorporating systematic literature review and qualitative interviews across six sub-Saharan African countries, Nigeria, Sierra Leone, Sudan, Tanzania, Zambia, and Zimbabwe [49] reported that the concept of instructional leadership is weakly understood in these countries. There is a lack of contextualized definitions and a proper understanding of this concept, which is essential for connecting it to broader school policies and practices in these settings. These studies illustrate that, in developed countries, the concept of instructional leadership has been well contextualized and effectively implemented. In contrast, in developing countries, the concept remains relatively new and has yet to be fully operationalized or grounded in local educational realities.

A contextual understanding of instructional leadership is necessary for its effective enactment, as principals’ practices are shaped by local factors such as government policy, available resources, and culture. A systematic “state-of-the-art” research review, including 302 quantitative and mixed methods studies from Asia [50], found that a school’s cultural context moderated the effects of instructional leadership on teaching and learning. Therefore, contextual adaptation of the definition and model of instructional leadership is essential for its effective implementation. In Nepal, a model of instructional leadership that aligns with the local context is crucial, though it has yet to be developed. Keeping this in mind, this study attempted to create an instructional leadership model by conducting a Delphi on Hallinger and Murphy’s (1985) model of instructional leadership. The classical Delphi technique was used to establish consensus among experienced panelists [51] by incorporating indigenous practices relevant to the Nepali context.

### Instructional leadership models

Notable models of instructional leadership have been proposed by various researchers [e.g., 24,37,38,52–55]. They have offered a variety of indications of instructional leadership, but the theoretical underpinnings are often the same [24,55]. Researchers most often use the Hallinger and Murphy (1985) model of instructional leadership in their research. The widespread acceptance of this model is primarily due to Hallinger and Murphy’s (1985) core concept, which provides head teachers with valuable insights into all aspects of leadership related to teaching and learning in schools. The primary objective of this model is to develop and communicate school goals, coordinate and supervise the school curriculum, monitor and evaluate student progress, encourage teachers and students, and maintain high visibility and a hands-on approach [56,57]. Their model is a scale known as the PIMRS (Principal/Head Teacher Instructional Management Rating Scale).

Among the models of instructional leadership proposed by various researchers, Hallinger and Murphy’s (1985) model was used as a theoretical guide for this research because it has received top priority and is more dominant in the literature on instructional leadership. This model of instructional leadership comprises three key factors: defining the school’s mission, managing the instructional program, and fostering a favorable learning climate. There are two functions under the factor describing the school’s mission, which are framing the school goals and communicating the school goals; three functions under the factor managing the instructional program, which are supervising and evaluating instruction, coordinating curriculum, and monitoring students’ progress; and five functions under the factor promoting a favorable learning climate which are protecting instructional time, providing incentives for teachers, providing incentives for learning, promoting professional development of teachers, and maintaining high visibility. There are five items under each of these ten functions, forming fifty variables altogether.

## Research methods

This study aims to identify the determining factors of instructional leadership using a quantitative research approach. Consistent with the assumptions typically underpinning quantitative research [58], this study is situated within the post-positivist philosophical paradigm. Post-positivists explore a single reality previously unknown [59]. They assume that reality exists independently, which can be approximated through careful observation and measurement of those variables [60]. Therefore, we argued that detailed observation and measurement of variables are essential for acquiring knowledge [60,61]. Following quantitative research approaches, the study employed a non-experimental, cross-sectional survey design, serving as the architectural blueprint for the investigation.

### Scale construction

Classical Delphi was employed in Hallinger and Murphy’s (1985) instructional leadership model, the Principal/Head Teacher Instructional Management Rating Scale (PIMRS), to develop a PIMRS suitable for Nepal’s context. The 50 items of Hallinger and Murphy’s (1985) PIMRS were thoroughly studied and subjected to a Delphi panel of experts, followed by three rounds of meetings to gather their ideas and opinions. The literature lacks consensus on the exact number of experts in the Delphi process [62]. However, following [63], a total of 20 panelists, one teacher, and a head teacher from 10 schools across diverse areas of Kathmandu were selected for the Delphi process. The criteria for their selection as experts in instructional leadership were more than five years of experience in the teaching-learning process in public secondary schools and their professional expertise as head teachers or teachers. The consensus in the Delphi process was measured as percent agreement, and the cut-off value in this study was 75%. It is in line with [62], which, on the basis of a review study, suggested that there is no widely accepted cut-off value (the value from which consensus is assumed) for defining consensus. It varies considerably; however, a threshold of 60% or higher was identified in most cases.

Based on the opinions of Delphi participants, some items- for example, (i) draw upon the results of class-wise testing when making curriculum decisions, (ii) participate actively in the review of curricular materials, (iii) acknowledge teachers’ exceptional performance by writing memos for their personal files, (iv) recognize superior students’ achievement or improvement by seeing in the office the students with their work, (v) review student work products when evaluating classroom instruction- were deselected from this PIMRS. Some items- for example, (i) head teacher announces mandatory presence of parents during terminal report card distribution, (ii) head teacher meets the parents of poor-performing students and shares with them what roles the school and parents may together take for their improvements, (iii) head teacher learns students’ family problems and talks to their parents to resolve them, (iv) the head teacher is transparent about every financial detail of the school to all stakeholders- were added to the new PIMRS.

Some items were changed to best suit Nepali schools, such as (i) monitoring the classroom curriculum to see that it covers the school’s curricular objectives was changed to the head teacher checking teachers’ log books regularly to see if they are going in accordance with the syllabus, (ii) limiting interruption of instructional time by public address announcements was changed to head teacher trying his best to ensure uninterrupted instructional time, (iii) leading or attending teacher in- service activities concerned with instruction was changed to head teacher seeking to provide training to teachers during vacations so as not to interrupt daily classes, (v) conducting informal observations in classrooms regularly was changed to head teacher regularly monitoring the activities of teachers and students while they are in class. In this way, a new PIMRS was finally developed for this research. This PIMRS consists of 11 factors and 33 items. A well-structured questionnaire was prepared using the new PIMRS. The questionnaire developed from the new PIMRS with 11 factors and 33 items asked the respondents to rate their view on a 5-point Likert scale with the descriptors: 1 = almost never, 2 = seldom, 3 = sometimes, 4 = frequently, and 5 = almost always, on which the head teacher was engaged in those particular activities of the school.

### Population and sample

The population of this study includes all public secondary schools in Kathmandu Valley. Kathmandu Valley was chosen for the study because it has the highest concentration of public secondary schools in the nation. In total, 303 public secondary schools in the Kathmandu Valley comprised the study population, and 172 schools were selected for the sample. The 172 schools were randomly selected from a pool of 303 for visits and data collection. Data (see S1 Dataset in the Appendix) were collected from one teacher and the head teacher at each of 172 schools to examine differences in ratings between head teachers and teachers. One teacher from each school was selected by lottery. The demographic and professional variables of respondents are presented at the end of this paper. Factor analysis was conducted on the data obtained to identify factors affecting head teachers’ instructional leadership (see S2_output@FactorAnalysis in the Appendix). The findings were analyzed and interpreted in the light of the relevant literature.

### Ethical consideration

Ethical standards were maintained throughout the research process, including during data collection. Before initiating the study, ethical approval was obtained from the Research Committee of Kathmandu University, School of Education (Approval No. 181028-1). The recruitment period for the pilot study was from July 24, 2019, to August 24, 2019. Similarly, the recruitment period for the main study began on August 25, 2019, and concluded on February 5, 2020. Informed consent was secured from all participants. Head teachers, who served as respondents, were initially contacted by phone to request their time and contribution. Consent from the teacher respondents was obtained during school visits. A questionnaire with a cover letter was distributed to each respondent. Some respondents provided data on the same day, while others scheduled a later time. Thus, the data was gathered in accordance with established ethical standards.

### Accessing the validity and reliability of the questionnaire

Pilot testing of the questionnaire was conducted with assistance from 30 head teachers and teachers at the 172 randomly selected schools. A default sample size of 30 is recommended for questionnaire pilot testing [64]. The pilot test results showed that, for most questionnaire items, the mean scores were more than four times the corresponding standard deviations. A low standard deviation indicates that most values are very close to the mean, indicating a normal distribution. The idea ensured the reliability of the questionnaire [65]. The data were collected from 172 public schools in the Kathmandu Valley using this questionnaire and subjected to factor analysis to determine the factors influencing instructional leadership.

### Safety check in the process of factor analysis

All the conditions required for factor analysis were checked. Factor analysis of Likert-scale data is highly valued [66]. The criteria were met as a 5-point Likert scale was used in this study. Fewer categories cannot offer more options or yield more reliable responses; on the other hand, a wide range of scale options can affect respondents’ responses and reduce their chances of providing the correct answer to a specific item [67]. Therefore, the questionnaire was constructed using a 5-point Likert scale. The ratio of sample size to variable should be 3–6 [68]. Given a sample size of 172 and 39 variables, this ratio falls within the recommended range of 3:1–6:1. An exploratory factor analysis was conducted using principal component analysis as the extraction method. Regarding the criteria for factor retention, one of the most common approaches is to retain factors with Eigenvalues greater than 1 [69,70]. In this study, all factors had Eigenvalues greater than 1. The rotation method applied was varimax. This study also met the item-loading requirement of 0.40 to 0.50 [71], with an item loading of 0.50.

Another condition is that the average commonality or extraction value for items is greater than 0.5 [71]. The average extraction value for items was 0.593. Therefore, this requirement was also fulfilled. Another criterion is Kaiser-Meyer-Olkin sampling adequacy. The KMO measure of sampling adequacy was 0.961. A KMO measure exceeding 0.50 suggests that the dataset has adequate sampling adequacy for factor analysis [69,72]. So, this condition was fully satisfied. The Bartlett’s test of sphericity was significant, [χ²(325)= 5190.76, p < 0.001], indicating that variables were sufficiently correlated for factor analysis. A further requirement is that a factor must have at least three components to be considered genuine [71,73]. This criterion was also met because there were 3–14 items in each of the three instructional leadership factors. All the conditions for factor analysis were met, and three factors of instructional leadership were obtained. The Cronbach’s alpha values for factors 1, 2 & 3 were 0.92, 0.90, & 0.75, respectively. The literature suggests that an alpha value of 0.75–0.95 is adequate [74]. Therefore, this value was acceptable. Thus, the reliability of factor analysis was assessed.

## Result

The factor analysis yielded three instructional leadership factors. Fourteen items were loaded on the first factor, eight on the second, and three on the third.

### Factor One: Planning, managing, and supervising instructional programs

All the items of factor one are shown below in the rotated component matrix of this factor (Table 1).

**Table 1:** Factor analysis (extraction method: principal component analysis).

Serial Number	Items	Factors		
		1	2	3
1.	The head teacher analyses students’ performance from the last year while planning for the new academic session.	.610		
2.	The head teacher specifies the school’s whole year educational plan in the presence of all concerned people.	.652		
3.	The head teacher seeks to incorporate teachers’ inputs in planning during his formal or informal meetings.	.588		
4.	The head teacher shares academic plans with everyone involved and discusses how those plans may be effectively implemented.	.651		
5.	The head teacher displays the school’s whole year plan on the notice boards and communicates it to students during assembly.	.639		
6.	The head teacher reports educational planning of the school to the local government and the Province/Local level education offices.	.604		
7.	The head teacher regularly monitors the activities of teachers and students while they are in class.	.634		
8.	The head teacher ensures that students complete their class work and homework on time and that all notes are marked by assigned teachers at least a month before terminal examinations.	.695		
9.	The head teacher seeks to identify the areas for improvement in instructional practices among the teachers.	.711		
10.	The head teacher gets every subject department head to implement the syllabus of all subjects in each class.	.653		
11.	The head teacher checks teachers’ logbooks regularly to ensure they are being kept in accordance with the syllabus.	.630		
12.	The Head teacher reviews whether the curriculum goal is achieved through students’ terminal examination results, their discipline, and overall change indicators.	.664		
13.	The head teacher regularly discusses with subject teachers regarding each student’s progress	.530		
14.	The head teacher instructs teachers to ensure no student has bunked classes	.528		

#### Explanation of variables in factor one.

These fourteen variables are connected to five distinct instructional leadership roles: planning and controlling school educational activities, overseeing and evaluating instruction, coordinating the curriculum and other educational objectives, monitoring students’ progress, and utilizing instructional time effectively. These specific instructional leadership functions of the head teacher, except the head teacher directs teachers to make sure no student has skipped class, are similar to the items in Hallinger and Murphy’s (1985) framework of IL falling under their two factors, defining the school mission, and managing instructional program [75–78]. Therefore, by including the meaning of those three leadership functions, namely defining the school mission, managing the instructional program, and ensuring no student has bunked the class, this factor was appropriately named as planning, managing, and supervising the instructional program. All the variables of the factor planning, managing, and supervising instructional program show that it is about making an educational plan for the school, coordinating the curriculum, sharing plans, observing instructors to ensure they are practicing good teaching techniques, communicating with teachers to learn about their students’ development, and making attempts to improve. There were two factors: framing and communicating school goals, and supervising and evaluating instruction for the particular instructional leadership activities in Hallinger and Murphy’s (1985) model [37]. They mentioned that it is about setting and expressing goals, working with staff members and teachers, and managing the instructional process and curriculum. According to [79], this involves establishing the school’s goals, reminding teachers of its purposes and mission, and informing teachers that they need to plan their classes properly. As stated by [80], this factor relates to selecting the school’s primary goals and to coordinating and managing curriculum and instruction. Similarly, [81] notes that planning, managing, and supervising instructional programs entail articulating and communicating the school’s goals, coordinating the curriculum, and monitoring and controlling the program through instruction supervision and evaluation.

This factor also resonates with the conceptualization of instructional leadership by [82], which is grounded in head teachers’ core practices and developed to explain how Norwegian school leaders build their instructional capacity. Their multidimensional model, consisting of goal orientation, collaboration with teachers, professional development, observation, and supervision, is similar to the variables identified in this study as factors in planning, managing, and supervising the instructional program. Both underscore head teachers’ active role in shaping and monitoring instructional processes as a foundation for improving teaching and learning.

This finding can also be compared with [36], which found that coordinating the curriculum and providing professional learning opportunities for teachers were statistically significant predictors of teachers’ professional development in the Nigerian context. Coordinating the curriculum is parallel to the variable “the head teacher gets every subject department heads to implement the syllabus of all subjects in each class” within the factor of planning, managing, and supervising instructional program. It reinforces the importance of this variable for developing teaching and learning ability. The next predictor, providing professional learning opportunities for teachers, corroborates the variables of the second factor, which motivate and develop the competence of teachers and students, as observed in this study.

### Factor Two: Motivating and developing the competence of teachers and students

All the items of factor two are shown below in the rotated component matrix of this factor (Table 2).

**Table 2:** Factor analysis (extraction method: Principal component analysis).

Serial Number	Items	Factors		
		1	2	3
1.	The head teacher publicly praises teachers’ superior performance, but meets them in private for correction.		.608	
2.	The head teacher tries to manage opportunities for sincere teachers to work in a higher position as a reward for their good work.		.668	
3.	The head teacher provides a letter of appreciation or honor certificates to teachers for their contribution.		.725	
4.	The head teacher seeks to provide training to teachers during vacations or so as not to interrupt daily classes.		.735	
5.	The head teacher actively supports teachers to use those learnt skills in the classrooms.		.695	
6.	The head teacher sometimes provides opportunities for observation or an educational tour for teachers.		.719	
7.	The head teacher publicly honors students for their excellent performance or discipline.		.489	
8.	The head teacher learns about students’ family problems and talks to their parents to resolve them.		.549	

#### Explanation of variables in factor two.

Factor two comprises eight variables that explain the instructional leader’s role in motivating and supporting teachers and students in their academic growth. These eight variables serve functions similar to those in Hallinger and Murphy’s (1985) Framework of instructional leadership, grouped under the functions of providing incentives for teachers, providing incentives for learning, and promoting professional development, and under the factor of creating a positive school climate [75–78]. Since the eight variables obtained from this factor share the same aim of inspiring teachers and students for their development, a suitable name, ‘motivating and developing competence of teachers and students’, was given to this factor.

All variables related to the factor of motivating and developing the competence of teachers and students indicate that it involves encouraging teachers and students to develop their abilities and academic excellence. Praising and rewarding teachers and students for their good deeds, assisting teachers with their professional development, and supporting students in enhancing their self-confidence and academic focus are key roles of an instructional leader, all of which are crucial to achieving academic success in the school. As stated by [79], recognizing and rewarding teachers’ and students’ accomplishments is important for promoting their interest and engagement. These instructional leadership roles align with two functions in Hallinger and Murphy’s (1985) instructional leadership model: upgrading professional development and providing incentives for learning. According to [81], this factor emphasizes inspiring and enhancing the competence of both teachers and students. The overall focus is on helping both teachers and students get better.

The variable within the factor, offering training to teachers during vacations, supporting the classroom application of acquired skills, and facilitating educational tours, corroborates the finding of [36], who found that providing professional learning opportunities to teachers supports their professional development. In contrast to the finding of [36], who did not see providing incentives for teachers to be a significant predictor of teachers’ professional development, the present study suggests that motivational strategies such as public praise, appreciation letters, honor certificates, and rewarding sincere teachers with opportunities for career development are integral to strengthening teachers’ competence. Additionally, the inclusion of student-focused practices, such as recognizing excellent performance and addressing family issues, reflects a broader and more relational approach to the holistic development of both teachers and learners.

### Factor Three: Involving parents

All the items of factor three are shown below in the rotated component matrix of this factor (Table 3).

**Table 3:** Factor analysis (extraction method: principal component analysis).

Serial Number	Items	Factors		
		1	2	3
1.	The head teacher calls class class-wise parents’ meeting and discusses students’ progress.			.553
2.	The head teacher announces the mandatory presence of parents during the distribution of the terminal report cards.			.839
3.	The head teacher meets with the parents of poor-performing students and shares with them the roles that the school and parents can take together to improve their performance.			.653

#### Explanation of variables in factor three.

The factor three of instructional leadership consists of three items. These items are linked to the head teachers’ policy to involve parents/guardians in being aware of their children’s condition and taking the appropriate action if necessary. In accordance with the meaning of these variables, a suitable name involving parents was given to this factor. The core idea, summarized by the variables of this factor, is to enhance parent- or guardian-school communication to support students’ academic development. The parents/guardians are also made accountable, along with the school, through this strategy. Parental role is found to be more critical to students’ academic progress in the Nepali context. Several studies have demonstrated the significance of the parent-school relationship. For example, studies [83,84] have shown a positive relationship between parental involvement and students’ academic achievement. [85] reported that increasing parental and community involvement in the educational process results in stronger relationships between school leaders and the community. Similarly, [86] emphasized the need to develop relational behavior among school administrators, including interactions with parents. Recent research [87] conducted in Hong Kong and Macao also demonstrated that leadership can influence student achievement through Parental Academic Commitment (PAC). The study reinforces the idea that parent involvement is not just a peripheral activity but a leadership-mediated pathway to better student outcomes.

## Discussion

The meaning of the variables under three different factors, the planning, managing, and supervising instructional program; motivating and developing competence of teachers and students; and involving parents, are closely aligned with some international findings on factors influencing leadership that contribute to improving teaching and learning in the schools. For example, these three factors identified in this study are parallel to the leadership pathways proposed by [88] in their study conducted in Chicago, USA. The planning, managing, and supervising of instructional programs aligns with the ‘learning climate’ pathway, in which head teachers establish conditions necessary to maintain a strong instructional focus. Motivating and developing the competence of teachers and students reflects the ‘professional capacity’ pathway, emphasizing the development of teachers’ skills, professional quality, and capacity to work together. Finally, involving parents directly corresponds to the ‘parent-community ties’ pathway, which strengthens collaboration between parents and school to enhance student outcomes. Again, [88] concluded that leadership affects students’ achievement primarily by improving the learning climate, thereby supporting the planning, management, and supervision of the instructional program as a factor in enhancing students’ academic outcomes through improved teaching and learning.

The findings of the present study can also be compared with the research on head teachers’ competencies by [89] in Vietnam, as instructional leadership is one of the various competencies of head teachers. Three of the eight competency domains identified in the Vietnam study, the ‘instructional program management’, ‘staff development’, and ‘engagement with families’, closely align with the three factors found in the present study. The idea is that the three dimensions developed in the present study are not only central to improving teaching and learning in Nepal but are also recognized internationally as essential aspects of effective school leadership. A meta-analysis [90] encompassing 24 studies and 9,178 teachers found that head teachers’ instructional leadership is moderately related to teacher self-efficacy, thereby enhancing student achievement. This finding aligns with the result of the present study, in which motivating and developing the competence of teachers and students emerged as one of the factors of instructional leadership. A systematic review of 349 Asian studies by [50] revealed that the PIMRS (Principal/Head Teacher Instructional Management Rating Scale) dominates the literature focusing on managing instructional programs and promoting a favorable learning climate. In this regard, the first two factors of the present study parallel those dimensions.

“Involving parents” is a new factor developed in this study. The role of head teachers in involving parents as a factor in head teachers’ instructional leadership in Nepal is a noteworthy finding. Although studies have shown that parental involvement is crucial for students’ achievement, none of the established instructional leadership frameworks explicitly recognize it as a distinct factor of instructional leadership.

An Indonesian study found a positive correlation between parental participation and academic achievement in school [91]. The study identified that students with active parental involvement had higher test scores and grade point averages. Numerous studies have shown that parental involvement has a significant impact on students’ academic performance. For example, a meta-analysis of 37 studies published between 2000 and 2013, encompassing kindergarten, primary, and secondary schools, found a modest and favorable impact of parental involvement on students’ academic attainment [92]. The mediation test results in [93] revealed a partially mediating role of parental involvement in the relationship between students’ socioeconomic status and academic achievement, as well as a strong mediating effect of the family environment on students’ academic achievement in rural China. A meta-analytic study employing a random-effects approach by [94] across Scopus-indexed publications found a significant positive correlation between parental involvement and student achievement.

While substantial research supports the positive impact of parental involvement on student achievement, some studies indicate that parental involvement is not always beneficial. A study conducted in Canada found no significant effect of parental participation on students’ academic progress [95]. A study conducted in the United States of America, involving 450 students from kindergarten to fourth grade, showed that students who scored higher academically had lower levels of parental involvement in school [96]. Such findings suggest that the country’s and locality’s cultural and societal institutions and practices may influence whether parental participation affects students’ academic achievement.

As discussed above, the study’s findings reveal three major factors influencing instructional leadership. Moreover, several studies [1–3,97] have shown that head teachers’ instructional leadership enhances students’ academic performance. Likewise, a recent study [98] found that head teachers’ instructional leadership influenced student achievement indirectly through the school’s physical environment and teacher performance. The other study [99] reported that head teachers’ instructional leadership indirectly affected student academic outcomes by shaping the school’s learning climate and instructional environment. Taken together, this evidence suggests that implementing the identified components of instructional leadership strengthens instructional practices, which in turn directly or indirectly contribute to improved student academic achievement.

## Conclusion

In response to the research question, “What factors determine head teachers’ instructional leadership?”, the study identified three key factors. The first factor, planning, managing, and supervising instructional programs, explains the head teacher’s role in collaborating with teachers to ensure the school has a clear mission focused on students’ academic achievement. The second factor, motivating and developing the competence of teachers and students, describes the head teacher’s activities aimed at increasing teachers’ and students’ interest in their work. The third factor, involving parents, highlights the roles of parents/guardians in collaborating with the school to support students’ academic progress. The study’s key finding is that parents’ or guardians’ involvement in students’ progress is viewed as an essential factor in schools in Nepal. This finding differs from Hallinger and Murphy’s (1985) model of instructional leadership. In Hallinger and Murphy’s (1985) instructional leadership model, parents’ or guardians’ involvement is not explicitly recognized as a factor in student success. Instead, the model includes only one related item, contacting parents to communicate improved or exemplary student performance or contributions, which falls under the factor ‘providing incentives for learning’.

This suggests that Nepal’s unique cultural and societal framework places greater emphasis on the role of parents/ guardians in students’ academic achievement. In Nepal, there is a family-oriented society, and within the family, there is a culture of obedience to parents/guardians. Parents/guardians instill values such as discipline, respect, hard work, and responsibility in their children. Such guidance and care affect children’s learning habits and achievement. Parents/guardians take an interest in monitoring their children’s academic performance and solving their problems. Therefore, parents’/guardians’ involvement emerged as a factor in Nepal’s cultural and societal context. By identifying these factors, this study makes a significant contribution to the field of instructional leadership, highlighting the specific requirements for effective teaching and learning in Nepali public schools.

### Implications and limitations of the study

The results of this study can help head teachers, in their role as instructional leaders, stay informed and offer recommendations to enhance teaching and learning in schools. The finding can also assist policymakers and bureaucrats in formulating and implementing necessary policies to improve head teachers’ instructional leadership. Additionally, it can support local authorities in improving schools, as the responsibility for school development is entrusted to local bodies in Nepal’s federal democratic system. In this way, the results of this study can help enhance school effectiveness and achieve quality education. Furthermore, this study has contributed new knowledge to the construct of instructional leadership in the local Nepali context by identifying the factors that influence it. This finding may be of interest to international researchers in this field.

As the present study employed a cross-sectional design, the results may be time-specific, limiting their generalizability across periods. Future studies using longitudinal approaches could allow examination of changes or developmental patterns over time. Future researchers can also employ mixed methods approaches to integrate both subjective perceptions and objective practices associated with instructional leadership. The present non-experimental, cross-sectional study also limited the causal interpretation. Use of an experimental design could provide more substantial evidence of causal relationships. Although data triangulation between head teachers and teachers was conducted, self-reported bias may still have occurred. Future research can be conducted by implementing a method that supports collecting data from both participants and through observations or objective records.

Since this study is confined to public secondary schools in Kathmandu, such a study could in the future also be conducted in institutional or public schools in other provinces for comparative analysis. Additionally, the effect of instructional leadership on students’ academic achievement can also be studied in different contexts.

## Supporting information

S1 Dataset This is dataset. (PDF)

S1 File Annex. Demographic information of the respondents. (DOCX)
