# Supplementary material for: Determinants of instructional leadership: A survey of public secondary schools
Source: PLoS One. 2026 Aug 14;21(8):e0355674. doi: 10.1371/journal.pone.0355674 (PMC13475869; doi:10.1371/journal.pone.0355674)
Supplement: Supplementary file 1 [file pone.0355674.s001.pdf]

Code	Q201	Q202	Q203	Q204	Q205	Q206	Q207	Q208	Q209	Q210	Q211	Q212	Q213	Q214	Q215	Q216	Q217	Q218	Q219	Q220	Q221	Q222	Q223	Q224	Q225	Q226	Q227	Q228	Q229	Q230	Q231	Q232	Q233
1	4	3	4	4	4	3	4	3	4	1	3	3	3	4	3	5	5	5	5	5	5	4	4	3	3	4	4	5	5	4	4	5	4
2	5	5	5	5	5	5	5	5	5	5	5	5	5	5	5	5	5	5	5	5	5	5	5	5	4	5	4	5	5	4	4	5	5
3	5	4	5	4	3	4	5	3	4	3	4	5	4	5	4	5	3	4	4	4	3	5	4	5	3	4	3	3	4	5	4	5	4
4	5	5	5	5	4	5	4	4	4	4	4	5	4	4	5	5	5	5	5	4	5	5	5	5	5	5	5	4	4	5	5	5	5
5	5	4	4	3	3	4	5	4	4	3	3	3	4	4	4	5	4	5	4	4	5	4	4	4	3	4	3	4	4	4	3	4	4
6	3	4	4	3	3	4	4	2	4	2	1	2	3	3	2	3	2	5	4	5	4	4	2	1	3	4	4	3	3	4	4	5	4
7	5	5	5	5	5	5	5	5	5	4	4	5	5	5	5	5	5	5	5	5	5	5	5	5	5	5	5	5	5	5	5	5	5
8	5	5	5	4	5	4	5	5	5	4	4	5	5	5	5	5	5	5	1	5	5	4	4	5	5	5	5	5	5	5	5	5	4
9	5	4	4	4	3	3	4	4	4	3	4	5	4	4	4	5	4	4	3	5	5	5	4	4	2	3	5	3	5	3	4	5	4
10	5	4	4	4	3	4	4	3	4	3	3	4	3	3	4	4	3	4	3	5	4	4	3	3	3	3	4	3	3	3	4	5	4
11	5	4	3	5	5	4	5	4	4	3	4	4	4	4	4	5	5	5	4	4	4	5	5	5	4	4	4	5	5	5	4	4	4
12	5	4	5	4	1	1	3	3	4	3	1	1	3	4	4	4	5	5	4	5	4	4	3	3	3	5	4	4	3	5	3	5	5
13	4	4	4	5	5	5	5	5	5	4	4	4	4	4	4	5	4	5	4	5	5	5	4	4	4	5	5	4	4	4	4	4	4
14	5	5	4	5	5	5	5	4	5	4	4	5	5	3	5	5	3	5	5	4	4	5	4	3	5	5	5	4	5	5	4	4	5
15	5	5	5	5	5	5	5	4	5	5	4	5	5	5	5	5	5	4	5	5	5	5	5	4	4	4	3	5	5	5	4	5	5
16	5	5	5	5	5	5	4	4	4	4	4	4	5	5	4	5	5	4	5	5	5	5	4	4	3	5	3	5	5	5	5	5	5
17	5	4	5	5	5	4	5	3	5	5	3	4	5	5	5	5	5	5	5	5	5	5	5	5	5	5	5	3	5	5	4	5	5
18	5	5	5	4	3	4	5	3	5	3	3	3	4	4	5	5	2	3	4	5	5	4	3	2	5	4	5	4	4	5	4	5	3
19	5	5	5	5	4	5	5	4	5	4	4	5	5	5	5	5	4	5	5	5	4	5	5	5	4	4	5	5	5	5	5	5	5
20	4	4	4	4	4	5	4	3	5	4	4	4	3	5	4	5	5	5	4	3	5	4	4	4	4	5	3	5	5	5	5	5	4
21	5	5	5	5	5	5	5	5	5	4	4	5	4	5	5	5	5	5	5	5	4	5	5	5	4	5	4	5	5	5	5	5	5
22	4	5	5	5	5	5	4	5	5	5	4	5	4	4	5	5	5	5	5	5	5	5	5	5	5	5	5	5	5	5	5	5	5
23	5	5	5	5	5	5	5	4	5	5	4	5	5	5	5	5	5	5	5	5	5	5	5	5	5	5	5	5	5	5	4	5	4
24	4	5	5	4	4	4	4	3	3	3	4	3	5	5	5	4	3	5	4	5	4	5	5	5	4	4	4	4	4	4	5	4	4
25	4	4	4	5	4	3	4	4	4	4	4	4	4	4	3	4	4	4	3	4	3	4	3	4	4	4	3	3	4	5	4	5	4
26	2	1	3	2	3	3	1	3	1	3	4	3	2	2	1	2	1	1	2	3	1	2	1	1	2	2	1	2	1	2	2	1	1
27	4	4	5	5	4	4	5	4	4	4	4	5	4	5	5	5	5	5	4	4	5	5	5	5	4	4	4	5	5	4	5	5	5
28	3	4	4	4	4	3	2	4	2	2	3	2	2	4	3	4	3	4	3	2	4	4	3	4	3	4	2	3	4	2	2	4	3
29	5	5	5	5	4	4	5	4	5	3	4	5	5	5	5	5	4	5	5	5	5	4	1	2	3	4	3	4	3	2	4	4	2
30	3	2	3	2	3	3	1	3	3	3	2	4	4	4	3	2	2	3	4	2	3	2	2	2	3	3	2	2	4	3	3	3	2
31	5	5	5	5	5	4	4	4	5	4	4	5	5	5	5	5	5	5	5	5	5	4	5	4	4	5	5	5	5	5	5	4	5
32	5	5	5	4	4	4	4	4	4	4	4	4	4	5	5	5	5	5	4	5	5	4	4	4	4	5	4	5	5	5	4	4	4
33	5	5	5	4	5	3	5	4	4	4	3	4	4	4	4	5	5	5	3	4	5	5	4	4	5	5	5	4	4	5	4	5	4
34	5	5	5	4	5	3	5	4	5	4	3	3	4	4	4	5	5	5	3	3	5	5	4	3	5	5	5	3	4	4	4	5	5
35	4	4	5	5	3	4	3	3	3	2	3	3	3	4	4	5	5	4	5	5	3	5	5	4	4	4	4	2	5	4	4	5	4
36	4	4	4	4	5	5	4	3	4	3	3	5	4	5	5	4	4	5	5	5	5	5	5	4	4	4	4	5	5	5	4	5	5
37	5	4	5	5	5	5	4	4	4	5	4	5	3	5	5	5	5	5	1	5	5	5	4	3	3	5	3	4	5	5	4	5	5
38	3	3	4	4	3	2	4	3	3	3	3	3	4	3	5	4	5	4	4	4	4	4	3	4	4	4	4	4	5	4	4	4	4
39	4	3	4	4	5	4	4	4	5	5	5	5	5	5	5	5	5	5	5	5	5	5	4	4	4	4	4	5	4	4	3	5	5
40	5	5	4	5	5	5	4	5	5	5	4	5	5	5	5	4	4	5	4	4	4	4	4	4	5	5	4	4	4	4	4	5	5
41	5	5	5	5	5	5	5	4	4	5	5	5	5	5	5	5	5	5	5	5	5	5	5	5	4	4	4	5	5	5	4	5	5
42	5	5	5	5	4	4	3	4	5	5	3	5	4	5	5	4	5	5	3	4	5	4	3	4	3	5	3	5	5	5	4	5	5
43	5	5	5	5	5	4	3	4	4	5	5	5	4	4	5	4	3	5	4	3	3	4	4	4	3	3	3	5	4	4	4	5	4
44	5	4	2	5	4	4	3	4	4	5	4	4	2	4	2	5	2	5	2	2	3	2	1	2	5	4	4	5	5	4	5	5	5
45	4	5	4	4	4	3	3	3	5	3	2	4	3	4	4	4	3	4	4	5	3	4	4	3	3	4	3	3	4	4	3	4	3
46	5	5	5	5	5	5	4	4	5	4	4	5	5	5	5	5	5	5	5	5	5	5	5	5	5	5	5	5	5	5	5	5	5
47	4	4	5	5	4	4	4	3	3	4	4	5	4	4	5	5	3	4	3	4	5	4	3	4	3	4	4	4	5	4	4	4	4
48	3	5	5	4	3	5	4	4	3	5	4	4	4	5	4	4	5	5	2	5	5	4	5	5	3	4	4	5	3	5	3	4	4
49	5	4	4	4	5	4	5	4	4	4	4	5	4	5	5	5	4	5	5	4	5	5	5	4	4	5	4	3	5	5	4	4	4
50	3	2	4	2	2	3	2	2	1	2	1	2	2	3	4	2	1	1	2	3	1	2	1	1	2	2	1	1	1	1	1	1	2
51	4	4	4	5	5	4	4	4	5	5	4	4	5	4	4	5	5	5	5	5	5	4	5	4	5	5	5	5	5	4	4	4	4
52	5	5	5	5	5	4	4	3	4	5	5	5	5	5	5	5	5	5	4	4	5	5	4	3	5	5	4	4	5	5	5	5	5
53	5	4	5	4	5	3	5	5	5	4	5	4	5	5	5	5	4	4	5	4	5	5	5	4	5	5	5	5	5	4	4	5	4
54	5	4	5	5	4	3	5	4	4	5	4	5	4	5	5	5	3	5	3	5	5	4	3	4	4	5	3	5	4	5	5	5	5
55	5	5	5	5	5	5	5	5	5	5	4	5	4	5	5	5	5	5	5	5	5	5	5	5	5	5	5	5	5	5	5	5	5
56	5	4	5	5	5	5	3	3	4	4	3	4	3	4	5	5	5	5	5	5	5	5	3	5	5	4	5	5	5	5	3	5	4
57	5	4	5	5	5	5	5	4	5	4	4	5	5	5	5	5	5	5	5	5	5	5	4	5	4	5	4	5	5	5	4	5	4
58	4	5	5	4	3	3	4	3	4	4	4	5	5	5	5	5	5	5	5	4	5	5	5	3	2	4	4	5	5	5	2	3	4
59	4	4	4	4	4	4	4	3	4	5	2	5	3	3	5	5	5	5	5	5	5	4	5	5	4	5	2	5	5	5	5	5	4
60	4	4	5	4	5	4	3	3	4	3	3	5	3	4	5	5	4	5	4	3	5	4	5	5	3	3	4	3	4	5	3	5	4
61	5	5	5	5	5	5	5	5	5	5	5	5	5	5	5	5	5	5	5	5	5	5	5	5	5	5	5	5	5	5	5	5	5
62	4	4	5	5	5	4	5	5	5	4	5	5	5	5	5	5	4	5	5	4	5	5	5	5	5	5	5	5	5	5	5	5	5
63	5	5	5	5	5	5	5	5	5	4	4	5	4	5	5	5	4	5	5	4	5	5	5	5	4	5	4	5	5	5	5	5	4
64	5	5	5	4	5	4	4	4	4	5	5	5	5	5	5	5	5	5	5	5	5	4	5	4	4	4	5	4	5	5	5	5	5
65	5	5	5	5	5	5	5	4	5	5	4	5	5	5	5	5	5	5	5	5	5	5	5	5	3	5	5	5	5	5	5	5	5
66	5	3	4	5	3	4	5	4	3	5	4	4	3	4	5	5	5	4	4	4	5	5	5	3	4	4	4	4	5	5	5	4	5
67	5	4	4	4	4	5	4	4	5	3	3	5	4	5	4	5	4	4	5	4	5	5	5	5	4	4	3	5	4	4	4	5	4
68	1	1	1	2	3	2	1	1	1	2	1	1	1	1	2	1	1	1	2	1	2	1	1	1	1	2	1	2	1	2	2	3	1
69	5	5	4	5	5	4	4	4	4	5	3	4	4	4	5	5	5	5	4	5	5	5	5	3	4	5	4	5	5	5	3	5	5
70	2	2	4	4	4	4	2	2	3	2	2	2	3	4	2	4	3	4	4	4	4	3	4	2	3	3	3	4	4	4	2	4	3
71	5	5	5	4	5	5	4	3	4	5	4	4	4	5	5	5	4	5	2	5	5	5	4	5	4	5	5	5	5	5	5	5	5
72	4	4	3	2	3	3	1	1	2	3	2	4	2	2	3	4	1	3	4	3	3	2	2	1	4	2	3	1	3	3	5	5	3
73	4	5	4	4	4	5	4	3	4	4	3	5	5	5	4	5	5	5	4	4	4	5	5	5	4	5	5	4	5	5	4	5	5
74	4	3	3	4	4	4	3	3	3	2	3	5	3	4	4	4	4	5	4	5	4	4	5	3	3	3	2	3	4	4	4	3	3
75	5	4	4	4	4	4	5	4	4	3	4	4	4	4	4	5	5	5	3	5	4	5	4	4	4	4	4	4	4	5	4	4	4

76	2	1	4	1	4	2	3	1	1	1	1	2	2	4	4	4	3	4	3	4	4	3	3	2	1	3	2	1	4	5	4	5	3
77	5	4	5	4	4	5	5	4	3	5	5	4	4	5	5	4	3	4	4	4	5	5	3	3	3	4	3	5	3	4	3	4	3
78	2	1	2	1	1	2	2	3	1	1	3	1	1	3	1	4	1	3	2	1	3	2	1	1	1	3	2	2	3	2	3	1	2
79	4	4	5	4	4	4	5	2	4	3	2	3	3	4	4	4	3	5	4	3	4	4	4	2	5	4	5	4	3	4	2	5	2
80	4	4	4	4	3	3	2	2	3	2	2	3	3	4	4	4	3	4	4	3	4	3	2	1	3	3	4	2	3	4	4	5	3
81	5	4	5	4	5	5	4	5	5	4	4	5	5	5	4	5	4	5	2	4	4	5	4	3	3	4	4	5	4	5	3	5	4
82	4	1	5	2	1	2	1	1	2	3	1	2	3	5	2	2	3	5	3	2	2	4	2	3	1	3	2	4	2	3	3	4	4
83	5	5	5	5	5	5	5	5	5	5	5	5	5	5	5	5	5	5	5	5	5	5	5	5	5	5	5	5	5	5	5	5	5
84	5	5	5	5	5	5	4	5	5	4	5	4	5	5	5	5	5	5	5	5	5	5	5	5	5	5	5	5	5	5	5	5	5
85	5	4	5	5	4	3	4	5	4	3	4	4	5	5	4	5	4	4	5	5	4	5	4	4	4	4	4	5	5	4	4	4	4
86	4	4	5	5	4	5	4	4	4	3	4	4	4	5	5	4	5	5	5	5	4	5	4	5	4	5	4	5	4	5	4	5	5
87	5	5	5	5	5	4	5	4	4	5	5	5	4	4	5	5	5	5	5	5	5	5	5	4	5	5	5	5	5	5	5	5	5
88	5	5	5	5	5	4	5	5	4	5	5	4	4	5	5	4	4	5	5	3	5	5	4	4	4	5	4	4	5	5	5	4	4
89	5	4	3	4	5	3	3	3	4	2	2	4	5	4	5	5	3	3	2	5	5	5	5	5	3	4	2	5	3	5	5	5	5
90	4	3	4	4	2	3	2	3	4	3	1	1	4	5	5	4	4	3	5	4	3	4	4	5	2	3	2	3	4	3	5	5	4
91	4	3	5	4	5	3	4	3	4	4	3	4	3	4	4	4	3	5	4	5	5	4	4	5	4	3	4	3	4	4	4	5	4
92	5	5	5	5	5	5	5	5	5	5	5	5	5	5	5	5	5	5	4	5	5	5	5	5	5	5	5	5	5	5	5	5	5
93	5	4	5	3	3	4	5	3	4	3	3	3	3	5	5	5	3	4	4	3	4	3	4	4	3	3	3	4	4	4	3	4	3
94	5	4	5	4	5	4	3	3	4	3	5	5	4	5	5	4	3	5	5	5	4	5	5	5	4	5	3	5	5	4	5	5	4
95	5	5	5	5	4	4	4	3	3	3	3	3	3	4	4	5	4	4	4	4	4	4	4	3	3	4	3	4	4	4	3	4	4
96	3	4	4	3	4	2	3	4	3	4	3	4	4	3	4	4	3	4	3	4	4	4	4	2	4	4	3	4	3	4	4	2	5
97	5	4	5	4	5	5	5	4	4	4	2	3	4	5	5	5	4	5	4	5	4	5	4	4	3	4	4	5	4	5	4	5	4
98	5	5	4	4	4	5	5	4	3	5	5	5	5	5	5	5	5	5	5	5	5	5	5	5	5	5	5	5	5	5	5	5	3
99	4	4	4	4	5	4	4	3	4	4	4	4	3	4	5	5	4	4	3	4	5	5	4	4	4	4	5	5	5	4	4	5	4
100	5	4	5	4	5	4	4	4	5	4	5	4	5	5	5	5	4	5	4	5	5	5	4	3	4	5	5	5	4	5	4	5	4
101	5	5	5	5	5	4	4	4	4	3	4	3	5	5	5	5	5	4	4	4	3	5	4	3	3	3	3	5	3	3	4	3	4
102	4	4	5	3	4	4	3	3	4	2	4	3	3	5	3	4	2	4	2	2	2	1	1	1	2	3	2	5	4	4	3	3	3
103	4	4	5	5	5	5	3	2	3	4	4	2	2	2	2	5	3	4	2	4	4	4	5	1	5	3	3	5	5	2	4	5	5
104	5	4	5	4	5	5	4	5	4	4	4	5	5	5	5	4	4	5	4	5	5	5	5	5	4	5	5	5	4	5	5	5	5
105	4	5	5	4	5	5	4	3	4	3	3	3	4	4	4	4	3	5	5	5	4	3	4	4	3	3	4	4	4	4	4	5	4
106	5	5	5	5	5	4	4	4	5	5	4	5	5	5	5	5	5	5	4	5	5	5	4	5	5	5	5	4	5	5	4	5	5
107	5	5	5	5	5	5	5	4	5	5	5	5	5	5	5	5	3	5	5	5	5	5	5	5	5	5	5	5	5	5	5	5	5
108	4	4	3	4	4	4	4	3	4	3	4	4	4	4	4	3	4	3	4	4	4	4	4	4	4	4	4	4	4	3	4	4	4
109	4	2	4	4	3	2	3	4	4	2	1	4	5	5	5	5	3	4	4	5	4	4	4	4	3	4	3	4	3	4	3	5	4
110	5	5	5	5	4	5	3	2	5	3	4	2	4	5	5	5	5	1	3	5	5	5	5	4	1	5	5	5	5	4	4	5	3
111	5	4	5	4	4	5	3	3	4	4	3	4	4	5	4	4	4	4	4	4	5	4	5	4	4	4	5	4	5	5	4	5	4
112	4	2	2	2	4	4	3	3	3	1	1	4	1	3	4	5	4	4	5	4	2	2	3	4	4	4	3	1	5	4	2	5	2
113	4	5	5	5	5	5	4	4	4	4	3	4	5	5	5	4	4	4	4	4	4	4	4	4	5	4	4	5	5	4	5	5	5
114	5	4	5	4	4	4	3	3	4	4	4	4	3	5	5	5	5	4	1	5	4	4	5	5	4	5	4	5	4	4	5	5	5
115	5	5	5	5	4	5	4	5	5	5	5	5	5	5	5	5	4	5	4	5	5	5	5	5	5	5	3	5	5	5	5	5	5
116	4	4	5	3	5	4	4	5	4	5	4	5	5	5	5	4	5	4	4	5	5	5	3	3	3	4	5	4	4	5	5	5	4
117	5	5	4	4	5	4	4	4	5	3	3	4	5	4	5	5	5	4	5	5	4	5	5	5	4	3	4	4	5	5	4	3	4
118	4	2	4	4	4	4	4	3	4	3	3	3	3	3	4	4	4	4	4	2	3	4	3	3	3	3	3	4	4	4	4	4	4
119	5	5	5	5	5	4	4	4	4	4	4	4	4	4	5	4	4	5	4	4	5	5	5	4	5	4	4	5	5	4	5	5	4
120	4	4	5	5	4	2	3	2	3	3	2	3	3	4	3	5	4	4	2	5	4	4	4	2	3	4	2	5	3	4	5	3	1
121	5	4	5	5	5	5	5	4	5	5	5	5	5	5	4	5	4	5	5	5	5	5	5	4	3	3	4	4	5	5	5	5	5
122	5	5	5	5	4	5	4	5	4	4	3	5	5	5	5	5	5	5	4	5	5	5	4	5	3	5	4	5	5	5	3	5	5
123	4	3	4	3	4	3	3	4	3	3	3	3	3	4	3	4	3	4	4	3	4	3	2	2	1	2	2	2	2	4	4	4	4
124	4	3	3	2	3	2	4	3	2	1	2	3	3	4	4	4	4	4	3	4	4	2	2	3	2	3	3	2	3	3	4	4	3
125	5	4	4	4	5	3	3	4	3	2	4	4	4	4	5	4	5	5	4	4	5	5	5	3	4	4	4	5	5	4	5	5	5
126	4	4	5	4	5	3	3	4	4	3	4	3	4	5	5	5	5	5	4	4	5	5	5	3	3	3	3	3	4	4	4	5	5
127	5	5	5	5	5	4	4	4	4	4	3	5	4	5	5	5	5	5	5	4	5	4	4	4	3	5	3	5	5	4	5	5	2
128	4	4	4	4	3	4	3	3	4	3	3	3	3	3	4	4	4	4	3	3	5	4	3	2	2	3	2	3	4	3	3	5	3
129	4	4	4	4	4	4	4	4	4	4	3	4	4	4	4	4	4	4	3	4	4	4	3	3	4	4	3	4	4	4	3	4	3
130	5	5	5	4	4	2	4	4	3	4	2	3	3	4	4	5	5	3	3	5	5	5	5	2	4	5	5	3	5	4	4	5	5
131	3	5	5	5	4	4	4	4	5	5	4	5	3	3	5	4	5	4	5	4	4	5	5	5	3	4	5	5	4	4	4	4	4
132	4	4	5	4	4	5	4	4	5	5	4	5	3	4	5	4	5	4	5	4	4	5	5	5	3	4	5	5	4	4	4	4	4
133	5	5	5	5	5	5	3	3	3	1	1	5	5	5	5	5	5	5	5	5	5	5	5	5	5	5	5	5	5	5	5	5	5
134	5	5	5	5	5	5	3	3	3	1	1	3	1	1	1	5	3	1	2	1	1	1	1	1	1	1	1	1	2	2	3	3	3
135	4	4	5	4	5	4	4	4	4	3	4	4	5	5	5	5	4	5	1	5	4	4	4	2	3	4	4	4	5	4	5	5	4
136	5	4	4	4	5	5	5	4	5	3	4	5	5	5	5	4	5	5	1	5	5	4	4	2	3	4	4	4	4	4	5	5	5
137	5	5	5	5	5	4	4	4	5	5	5	5	5	5	5	5	5	5	5	5	5	5	5	5	4	5	5	5	5	5	4	5	4
138	5	4	4	5	5	5	4	3	4	3	4	3	3	5	5	5	2	5	4	4	4	5	5	5	4	4	4	5	4	4	5	5	5
139	5	4	5	5	4	4	5	5	4	3	3	5	5	5	5	4	4	4	3	4	4	5	3	3	2	5	3	3	3	4	4	5	4
140	4	4	4	3	5	3	4	5	4	3	2	5	5	5	4	4	3	4	3	4	4	3	4	4	3	3	3	3	4	4	4	3	3
141	5	5	5	4	4	5	5	4	5	5	5	5	4	4	5	5	5	5	5	5	4	5	4	3	3	5	5	5	5	5	4	5	5
142	4	5	5	4	5	5	4	5	4	3	5	5	4	4	5	5	4	5	3	4	5	5	4	4	4	5	4	5	5	4	4	3	4
143	5	5	4	5	5	5	4	4	5	4	4	5	4	5	5	5	5	4	4	5	5	5	4	4	4	4	3	3	4	5	3	3	4
144	4	5	5	5	5	4	4	4	5	5	5	5	5	5	5	5	4	5	4	5	5	5	3	3	4	5	4	5	5	5	5	5	5
145	3	4	5	4	5	2	3	2	3	2	2	5	4	4	3	5	3	4	3	5	5	5	5	3	4	5	4	3	2	4	4	4	4
146	4	5	4	4	4	3	2	3	2	2	2	3	2	3	2	4	2	2	3	4	4	4	4	4	3	3	3	4	4	4	4	4	4
147	5	5	5	5	5	5	5	5	5	4	4	5	5	5	5	5	5	5	5	5	5	5	4	5	4	5	5	5	5	5	3	3	3
148	5	5	4	4	5	4	5	5	4	3	4	5	4	5	5	4	5	5	4	5	4	4	3	3	4	5	5	5	4	4	2	3	3
149	3	4	5	4	5	4	3	3	4	4	5	5	4	4	5	4	5	4	4	5	4	4	4	3	4	4	5	4	5	4	4	5	4
150	3	3	4	4	4	3	3	2	3	4	4	4	3	4	5	5	4	4	3	4	3	4	2	2	3	4	3	4	3	5	4	5	4
151	5	5	4	4	5	5	4	5	5	5	4	4	5	5	4	5	4	5	5	5	5	5	4	4	4	4	5	5	5	5	4	4	3

152	5	5	5	4	5	4	4	5	4	3	3	5	4	5	4	4	3	4	3	2	4	3	2	4	2	3	3	5	4	4	5	5	4
153	4	3	4	4	5	5	3	4	4	4	4	5	4	5	4	5	4	4	3	5	5	4	4	4	3	3	3	4	5	5	4	5	4
154	4	4	4	4	3	2	4	2	2	2	2	3	1	2	2	3	3	4	3	4	2	2	3	3	2	3	3	3	3	4	3	3	2
155	4	4	5	5	4	4	5	5	4	5	5	4	3	3	4	5	5	5	5	5	5	5	4	5	5	4	5	5	5	5	5	5	4
156	5	5	4	4	5	5	4	3	3	2	4	3	3	4	4	5	4	3	4	4	4	5	3	5	5	4	5	5	5	4	4	5	5
157	5	5	5	4	4	4	4	4	4	5	5	5	4	4	5	5	5	5	5	5	5	4	5	3	2	5	4	5	5	5	3	4	4
158	4	3	3	4	5	4	4	5	4	3	2	3	3	4	4	5	4	5	2	3	4	3	2	2	2	5	4	5	5	4	2	5	4
159	5	4	5	5	4	3	4	3	3	4	3	4	5	5	5	4	3	3	5	4	4	3	4	4	3	3	4	5	4	4	4	4	3
160	4	4	5	5	5	5	5	4	5	5	5	5	4	5	5	5	5	5	5	5	5	5	5	4	5	5	4	5	5	5	4	4	5
161	5	5	4	5	4	4	4	4	4	4	4	5	4	4	5	4	3	4	5	5	5	5	5	4	4	4	5	4	4	5	4	5	5
162	4	5	5	5	5	4	4	3	4	4	4	4	4	4	5	5	5	4	5	5	5	4	4	4	4	4	5	4	5	5	4	4	5
163	4	4	4	5	5	4	3	4	5	4	5	4	5	4	4	4	3	5	3	4	4	3	4	2	3	2	2	4	4	3	3	5	3
164	5	5	5	4	5	3	5	4	4	3	3	4	5	5	5	5	4	4	5	5	5	5	5	5	3	3	3	4	4	5	4	5	3
165	3	4	4	4	5	4	5	4	4	4	2	3	3	5	5	4	3	4	5	5	3	4	4	2	2	3	4	4	4	4	2	5	5
166	2	2	3	2	2	4	4	2	1	1	1	2	2	2	3	4	1	4	4	2	4	3	2	1	1	2	2	3	4	2	3	5	4
167	5	5	5	5	4	3	5	5	5	4	5	5	5	5	4	5	5	5	5	5	5	5	5	5	5	5	5	5	5	5	3	5	5
168	1	2	5	3	5	5	3	5	5	1	2	5	2	5	5	5	4	5	5	5	5	4	3	3	4	5	4	5	4	5	4	5	4
169	4	3	5	5	5	3	4	5	4	4	5	5	2	3	4	5	2	4	2	4	3	4	4	1	3	4	1	2	4	4	5	5	4
170	3	3	3	3	3	3	2	3	3	3	3	2	3	4	4	4	4	4	4	4	5	4	4	4	4	4	3	4	4	4	4	4	4
171	5	4	5	4	5	4	4	3	5	3	3	5	3	4	5	5	5	5	3	5	5	4	5	5	3	4	2	5	5	5	4	5	5
172	4	5	5	4	5	5	5	4	5	5	4	5	5	5	5	5	3	5	5	5	5	2	1	1	2	2	2	5	5	3	1	5	3
173	5	4	5	4	5	4	5	4	4	2	5	5	5	4	5	5	4	5	4	5	5	5	5	4	3	5	5	5	4	4	5	5	4
174	5	5	5	5	4	5	5	5	4	3	5	5	5	5	5	5	5	5	5	5	5	5	5	5	5	4	5	5	5	5	5	5	5
175	4	4	4	5	4	4	4	4	4	3	4	4	5	5	5	5	5	4	4	4	4	4	3	3	3	4	4	4	4	4	4	4	4
176	5	4	4	5	5	3	4	5	4	4	3	4	5	4	4	5	4	5	4	5	4	4	4	4	4	5	5	5	5	4	4	4	4
177	4	5	5	4	5	5	4	5	4	4	4	5	5	5	5	5	5	5	4	4	5	5	5	5	4	5	5	5	5	5	5	5	4
178	5	4	4	4	4	4	3	3	5	4	5	5	5	5	5	5	5	5	4	5	5	5	5	5	5	5	5	4	4	4	3	5	5
179	5	5	5	5	5	5	2	4	4	3	3	3	5	5	5	5	5	5	5	5	5	4	4	4	3	3	3	4	4	5	5	5	4
180	5	5	5	5	5	5	2	4	4	2	2	2	4	5	5	5	5	4	5	5	5	4	4	3	3	3	3	3	4	5	5	5	4
181	4	5	5	5	5	5	5	5	5	5	5	4	4	4	5	5	5	5	5	5	5	4	4	4	4	5	4	4	4	5	4	4	4
182	5	4	4	4	5	5	4	4	5	5	5	5	4	3	4	5	5	5	5	5	5	5	4	5	5	5	5	4	5	5	5	4	5
183	4	4	5	4	5	4	5	4	4	4	3	4	4	4	5	5	4	5	5	5	5	4	4	5	5	5	5	5	5	4	4	5	4
184	4	4	4	4	5	4	5	4	4	3	3	4	4	4	4	4	4	5	5	5	5	4	4	4	4	4	4	4	5	5	4	4	4
185	5	5	5	4	5	5	5	4	4	5	5	4	3	5	5	5	5	4	5	5	5	4	3	4	3	5	5	5	5	5	4	4	4
186	5	5	5	5	4	4	3	3	4	5	5	4	4	5	4	5	5	5	5	5	5	4	4	2	5	5	3	4	4	5	2	2	4
187	4	3	5	3	5	4	2	3	3	3	4	5	4	5	4	5	5	5	3	4	5	3	4	4	3	5	2	5	4	4	3	5	4
188	3	2	4	3	5	1	2	3	2	3	4	3	1	5	4	5	3	4	1	1	4	3	1	3	3	4	1	2	3	4	3	3	2
189	5	5	5	4	5	5	5	4	4	5	4	5	5	5	5	5	5	5	5	5	4	5	5	4	4	5	4	4	5	5	5	4	5
190	4	3	5	4	4	3	3	3	3	4	3	3	4	5	3	5	3	5	4	5	5	4	4	3	3	3	4	4	5	4	4	3	3
191	4	3	4	3	5	5	3	3	3	1	1	2	1	2	1	5	3	5	1	3	5	2	5	4	5	5	4	5	5	3	3	5	4
192	2	1	3	2	1	1	2	2	1	1	1	1	1	2	3	2	2	4	1	2	3	3	2	4	1	1	3	3	3	2	1	1	1
193	5	4	4	5	5	5	5	4	4	4	5	5	5	4	4	5	5	5	5	5	4	5	4	3	3	5	3	4	5	5	3	5	5
194	5	4	4	3	4	4	3	3	4	2	4	5	4	3	4	5	4	5	3	5	4	3	4	2	3	4	3	3	4	3	2	2	3
195	5	4	3	3	4	5	5	4	2	3	4	5	5	5	4	5	2	5	5	4	5	5	5	5	3	4	5	5	3	5	5	5	4
196	4	4	4	4	4	4	4	4	3	4	4	4	5	5	4	5	3	4	5	5	5	5	5	5	3	5	3	5	4	5	5	5	4
197	5	5	4	5	4	5	4	3	4	4	5	5	5	5	5	4	3	4	5	5	5	5	5	5	5	4	5	5	5	5	5	5	4
198	5	5	4	5	5	5	4	4	4	5	5	5	5	5	5	5	5	4	4	5	5	5	5	5	5	5	5	5	5	5	5	5	5
199	5	5	5	5	5	4	5	5	4	4	3	5	4	5	5	5	5	5	5	5	4	4	5	3	3	5	4	5	4	4	5	5	5
200	4	4	3	3	4	4	4	3	3	2	2	3	2	3	4	4	5	4	4	2	4	3	2	2	3	3	4	3	4	4	4	5	4
201	5	5	4	4	5	5	3	3	3	3	3	4	3	3	5	5	3	5	3	5	5	4	5	5	3	3	3	5	4	4	3	5	4
202	5	4	4	4	4	4	3	1	3	1	1	2	4	3	4	5	5	5	4	1	3	1	1	2	2	4	2	3	2	3	4	5	5
203	5	5	5	5	5	5	5	5	5	5	5	5	5	5	5	5	5	5	5	5	5	5	5	5	3	5	4	4	5	5	5	4	5
204	5	5	4	4	4	4	4	5	4	4	4	5	4	4	5	5	4	5	4	5	5	5	4	3	2	4	4	4	4	4	3	4	5
205	5	5	5	5	5	5	4	4	5	5	5	5	5	5	5	5	5	5	4	5	5	5	5	5	5	5	5	5	5	5	5	5	5
206	5	5	5	5	5	5	4	4	4	4	4	4	4	4	4	5	5	5	3	4	4	4	4	4	4	4	3	4	4	4	4	4	4
207	1	2	2	3	3	4	3	2	2	3	2	4	2	4	3	3	5	5	2	3	5	2	2	2	2	2	3	2	4	2	5	5	4
208	1	1	2	5	5	5	1	1	1	4	2	1	1	4	1	5	1	2	1	1	2	1	1	1	1	2	4	1	2	1	1	4	1
209	3	5	4	3	3	3	3	3	2	2	3	2	3	3	2	4	3	3	3	3	4	4	4	3	3	4	3	5	5	5	4	4	4
210	3	2	2	2	2	1	2	1	1	1	1	2	1	1	2	3	1	1	1	1	1	1	1	1	1	1	2	3	3	3	3	4	2
211	4	4	5	4	5	4	4	4	4	4	3	4	4	4	5	4	3	5	5	4	4	5	4	4	4	3	4	4	4	4	5	4	3
212	3	4	4	2	4	3	4	2	3	2	4	2	2	3	4	4	3	3	2	3	1	2	2	2	1	2	1	1	1	2	1	4	4
213	4	4	5	5	4	5	4	5	5	4	4	5	5	5	5	5	5	5	5	5	5	5	5	3	5	5	5	4	5	5	4	5	4
214	5	4	4	4	4	4	3	3	3	3	2	5	3	3	5	4	5	5	5	5	4	4	1	2	3	3	3	5	5	5	4	5	5
215	5	4	5	5	4	5	4	3	5	4	4	5	4	4	3	5	4	5	4	5	4	5	4	5	3	4	3	4	5	5	4	5	5
216	4	5	4	5	5	4	3	4	4	4	5	5	4	5	5	4	4	5	4	5	5	4	4	5	4	4	3	4	4	4	4	5	5
217	4	5	5	4	4	4	4	3	3	2	3	4	3	5	5	5	3	3	3	3	4	4	4	4	5	4	4	4	5	3	4	5	5
218	5	4	3	2	3	3	3	3	2	1	3	3	3	5	3	4	2	2	3	4	3	2	2	3	2	3	1	4	3	3	5	4	3
219	4	4	5	4	4	4	4	3	4	4	3	4	4	4	4	4	3	5	4	4	5	4	5	4	4	5	4	5	4	4	4	5	4
220	5	5	4	4	5	5	4	3	3	4	3	5	4	4	5	5	4	5	5	5	5	5	4	4	4	5	4	5	5	4	3	5	5
221	4	4	5	4	3	4	3	3	4	3	3	3	4	5	5	4	3	2	4	4	2	3	2	4	4	5	4	5	4	5	5	5	3
222	4	3	4	4	5	2	4	2	4	3	5	3	4	4	5	4	3	2	4	1	4	5	3	3	2	5	5	4	4	3	4	5	4
223	4	5	5	5	5	5	4	4	5	4	3	4	4	5	5	5	4	5	5	5	5	5	5	4	5	5	4	5	5	5	4	5	5
224	4	3	5	3	4	4	3	2	2	3	2	4	4	4	3	4	2	4	2	4	3	2	4	2	4	3	4	4	4	3	4	5	3
225	4	5	5	4	4	4	4	5	4	5	4	5	5	5	5	5	4	5	5	5	5	4	4	4	4	5	5	4	4	4	4	4	4
226	4	5	4	3	3	4	3	3	4	3	3	4	3	5	4	4	4	5	3	5	3	2	2	2	2	3	4	4	4	4	4	5	4
227	4	4	4	5	4	3	5	5	5	4	4	3	5	4	4	5	4	5	4	5	5	4	5	4	4	3	3	4	4	4	3	4	3

228	4	5	4	4	4	3	5	4	4	4	3	4	3	4	3	4	5	5	4	4	5	4	3	3	4	4	3	3	4	5	3	4	4
229	4	4	5	3	2	3	4	2	5	4	5	4	3	5	5	5	3	5	4	4	5	4	4	4	4	5	4	5	5	5	5	5	5
230	4	4	4	4	3	3	4	3	4	5	4	4	3	3	4	5	3	5	2	4	4	4	3	3	3	4	4	4	3	4	5	3	4
231	4	3	4	4	4	5	3	4	5	3	2	5	4	5	5	4	5	5	5	5	5	4	3	3	3	5	4	4	4	5	4	3	4
232	4	4	4	4	4	4	3	4	4	3	3	5	4	4	4	4	4	4	5	5	5	4	3	3	3	5	3	4	4	4	4	4	4
233	5	5	5	4	5	4	5	4	4	4	4	5	5	5	5	5	4	5	3	5	5	5	5	5	4	4	5	5	5	5	5	5	4
234	4	4	5	5	5	4	5	5	5	4	4	4	4	5	5	4	5	4	4	5	55	5	5	5	5	5	5	5	5	5	5	5	5
235	5	5	5	4	4	5	5	3	4	3	3	5	5	4	5	5	5	5	3	4	5	5	5	3	4	5	4	3	3	4	5	5	5
236	4	5	5	5	3	3	2	2	3	1	1	3	2	2	4	5	5	5	2	1	5	4	5	1	1	1	1	4	3	3	4	5	4
237	5	5	5	5	5	5	5	4	5	4	4	5	4	5	5	5	4	5	3	5	5	5	4	5	4	3	5	5	5	5	5	5	5
238	5	5	5	5	5	5	3	4	4	4	3	4	4	4	4	5	5	5	5	4	5	5	3	3	2	2	2	5	5	5	5	5	5
239	4	4	4	3	5	4	4	4	5	3	4	4	4	5	5	5	5	4	5	5	5	5	5	5	4	4	5	5	5	4	4	4	3
240	4	3	3	4	3	2	2	3	2	2	4	3	2	2	3	4	3	3	3	2	2	3	4	5	3	2	3	3	3	3	3	4	3
241	5	5	5	5	4	4	4	5	5	5	5	5	4	5	5	5	5	5	5	5	5	5	5	5	5	5	5	5	5	5	5	5	5
242	5	5	5	5	5	5	5	5	5	5	5	5	5	5	5	5	5	5	5	5	5	5	5	5	5	5	5	5	5	5	5	5	5
243	5	5	5	5	5	5	5	5	5	5	4	5	5	4	5	5	5	4	4	5	5	5	5	4	4	4	3	5	5	5	4	4	4
244	5	5	5	5	5	5	4	4	4	5	4	4	5	5	5	5	4	4	4	5	5	5	4	4	4	5	3	5	5	5	4	4	4
245	5	4	5	4	4	3	5	3	4	4	3	3	4	4	5	5	2	5	4	5	5	5	5	4	3	5	5	4	5	4	3	4	4
246	4	4	3	3	3	4	3	4	3	3	4	4	5	5	4	4	4	5	4	5	5	4	3	4	4	4	4	5	5	4	3	4	3
247	5	5	5	5	4	4	5	5	5	5	5	5	4	5	5	5	5	5	5	4	5	5	5	5	3	3	5	5	5	5	5	5	5
248	5	5	5	5	5	5	5	5	5	4	5	5	5	5	5	5	4	5	5	5	5	5	5	5	5	5	5	5	5	5	5	5	5
249	3	4	5	5	5	5	4	4	4	4	5	4	4	4	5	5	5	5	4	5	4	5	5	5	3	4	5	4	4	5	4	5	5
250	4	3	4	4	3	3	4	4	3	3	3	4	3	4	3	4	3	4	3	3	3	4	3	3	3	4	3	3	4	4	3	4	4
251	5	4	4	4	5	4	5	4	5	5	4	4	5	5	5	5	5	5	5	4	5	4	5	4	5	4	5	5	4	5	5	5	5
252	5	5	4	4	4	4	5	5	5	4	4	4	5	5	5	5	5	5	4	5	5	5	4	4	4	5	4	5	4	4	5	5	5
253	5	5	5	5	5	5	5	3	5	3	5	4	3	5	5	5	5	5	5	4	5	4	4	3	4	5	3	5	4	5	5	5	5
254	4	3	4	3	3	4	3	3	4	2	2	4	2	5	5	5	3	5	4	3	4	3	2	2	2	3	3	4	4	3	3	4	4
255	5	4	5	5	5	5	5	4	4	4	5	5	5	5	5	5	5	5	5	5	5	5	4	5	4	5	5	5	5	5	4	5	4
256	5	5	5	4	5	5	4	4	5	3	3	3	5	5	5	5	4	4	5	4	4	4	3	3	4	4	5	5	4	5	4	5	4
257	4	3	5	4	4	3	3	3	4	3	4	3	3	5	5	4	3	5	3	3	5	5	2	2	3	4	4	5	5	4	3	5	5
258	4	3	4	3	4	3	4	3	4	3	3	4	5	5	5	5	4	5	1	3	3	2	4	2	3	4	3	3	3	4	3	4	3
259	4	5	5	5	3	4	3	2	3	3	2	4	2	3	4	4	4	5	5	5	4	5	5	4	3	4	5	4	4	4	3	5	4
260	3	3	2	4	1	2	1	1	1	1	1	1	1	1	1	1	1	1	1	1	2	5	2	2	1	1	1	4	1	3	1	3	1
261	4	4	3	4	5	4	4	4	5	4	4	3	4	4	5	5	5	5	4	4	5	4	3	3	4	4	4	4	4	5	3	5	3
262	5	4	5	5	5	5	3	3	4	2	2	5	4	3	5	5	5	5	4	5	5	4	5	5	3	4	4	5	5	5	4	5	5
263	4	3	4	5	5	4	5	5	4	5	3	4	4	4	5	5	4	5	4	5	4	5	3	4	5	5	5	5	4	5	4	5	4
264	5	5	4	4	5	4	4	4	4	4	3	4	3	4	4	4	5	5	4	5	5	3	4	3	3	4	4	5	4	4	4	5	3
265	5	4	4	4	5	4	5	3	3	3	4	4	5	5	5	4	4	4	5	3	4	4	4	4	4	5	5	4	5	5	5	5	5
266	5	4	5	4	5	4	4	5	4	5	5	5	5	4	5	4	5	4	5	4	5	5	4	4	4	5	5	4	5	4	4	5	4
267	5	5	5	5	5	5	4	4	4	4	4	5	4	5	5	5	5	5	5	5	5	5	4	4	4	4	3	5	5	5	5	5	5
268	4	4	4	4	3	3	3	4	4	3	3	3	2	3	3	4	2	4	4	3	4	4	3	4	3	3	4	4	4	3	4	3	3
269	5	4	5	4	4	4	3	4	4	5	4	4	5	5	5	4	4	5	4	5	4	5	3	3	3	3	3	3	3	5	4	4	3
270	5	5	5	5	4	5	4	4	4	4	3	4	4	5	5	5	4	5	4	5	4	4	4	3	3	5	4	3	3	5	4	5	3
271	4	3	5	4	5	3	4	3	4	4	2	4	3	4	5	5	5	5	4	5	5	5	4	4	3	4	3	4	4	4	3	5	5
272	4	4	4	5	5	3	3	3	4	3	2	5	3	4	5	4	2	2	3	4	4	4	2	1	3	3	3	4	3	4	4	5	4
273	4	5	4	4	4	4	4	4	4	4	4	4	4	3	5	5	4	4	4	5	5	4	4	4	4	5	4	5	5	5	5	5	5
274	5	4	5	4	4	3	5	4	4	5	4	5	5	5	5	5	4	5	4	5	5	5	5	5	5	5	5	5	5	5	5	5	4
275	4	4	4	5	4	3	4	4	4	3	3	4	4	5	4	5	4	4	3	5	4	5	4	5	4	5	4	2	4	5	3	5	5
276	4	4	5	5	3	5	3	4	5	2	4	3	4	5	5	3	4	5	5	5	5	4	3	2	2	5	3	5	4	4	3	5	5
277	5	5	4	4	5	3	5	4	5	4	3	5	4	5	4	4	4	5	4	5	4	5	4	3	3	4	3	3	4	5	4	3	5
278	4	4	5	4	5	5	5	4	5	4	4	5	4	5	5	4	4	5	5	5	4	4	4	4	5	5	4	4	4	4	4	5	5
279	4	4	5	5	4	3	5	4	5	4	3	5	4	4	4	5	5	5	1	4	4	5	3	3	3	3	3	2	5	4	4	5	5
280	4	4	5	5	3	3	5	4	5	3	1	5	2	4	5	5	4	5	1	2	4	5	3	2	3	3	3	2	5	4	4	4	4
281	5	3	5	5	5	4	4	3	5	4	4	5	5	5	5	5	5	5	5	5	5	5	5	5	4	5	3	5	5	5	5	5	5
282	5	4	5	5	4	4	4	4	5	5	4	4	5	5	5	5	5	5	3	5	5	5	4	3	4	4	5	5	5	5	4	5	5
283	5	5	5	5	4	4	4	3	4	3	3	3	4	4	4	5	3	4	3	4	3	4	3	2	3	3	3	3	4	4	4	4	4
284	4	3	3	3	2	3	3	3	3	2	2	3	2	2	3	3	4	3	3	4	3	3	3	2	2	3	2	3	3	3	3	4	3
285	5	5	5	4	5	5	3	4	5	5	4	5	5	5	5	5	5	5	4	5	5	5	5	5	4	5	5	5	5	5	4	5	4
286	2	2	3	2	1	1	1	2	2	1	1	1	1	2	1	3	1	5	1	5	4	2	3	2	4	3	1	1	4	3	1	5	3
287	4	5	4	5	5	4	5	4	5	4	4	4	5	5	5	5	5	5	4	5	5	5	4	5	4	5	4	4	5	5	4	5	5
288	4	5	5	5	5	5	4	5	5	3	4	5	5	5	5	4	4	5	4	4	3	3	5	5	5	5	3	5	3	4	5	5	5
289	4	3	5	4	4	4	4	4	4	2	4	4	4	4	5	5	5	4	4	3	4	4	4	3	4	4	3	4	4	5	3	5	4
290	4	5	5	4	4	4	4	3	4	3	5	4	3	4	4	5	4	4	3	4	4	4	4	3	4	4	4	4	4	4	4	5	4
291	5	4	5	4	5	4	5	4	5	4	4	5	5	5	5	5	4	5	5	5	4	5	5	5	4	5	5	5	5	5	5	5	5
292	5	4	4	4	5	5	4	4	4	4	4	5	5	5	5	5	5	5	4	5	4	5	5	5	4	5	4	5	5	5	5	5	5
293	5	5	5	5	5	5	5	5	5	5	5	5	5	5	5	5	5	5	5	5	5	5	5	5	5	5	5	5	5	5	5	5	5
294	5	5	4	5	5	5	5	4	5	4	5	5	4	5	5	5	4	5	4	5	4	4	5	5	4	4	5	5	5	5	5	5	5
295	5	5	5	5	5	5	5	5	5	5	5	5	5	4	5	5	5	4	4	5	5	5	3	3	3	5	3	5	5	5	4	5	5
296	3	2	4	4	4	5	5	4	3	5	4	5	3	3	5	5	4	4	4	2	4	3	2	2	4	2	2	4	3	5	4	5	3
297	5	5	4	3	3	4	3	4	4	4	3	4	3	4	4	4	3	5	5	4	5	5	4	5	5	5	5	4	4	4	4	5	5
298	5	3	4	5	5	4	3	2	3	3	3	3	4	5	5	4	3	5	3	2	3	5	4	5	4	3	3	5	3	4	4	5	4
299	4	3	4	3	3	2	3	3	3	3	4	3	3	2	3	2	3	3	2	3	3	3	3	3	3	3	2	3	3	4	2	2	2
300	2	2	3	4	4	4	3	3	3	3	2	2	1	3	3	4	3	3	2	3	4	3	2	1	2	3	3	4	4	4	3	4	4
301	4	4	5	3	4	3	5	5	4	3	3	4	5	4	4	4	5	4	5	4	5	5	5	1	5	5	5	5	5	4	4	5	4
302	5	5	4	4	4	5	4	3	4	5	5	4	3	3	5	5	5	5	2	5	5	3	1	4	4	5	4	4	5	5	3	5	4
303	4	4	5	5	5	4	4	4	4	5	4	4	5	5	5	5	4	4	4	5	5	5	4	4	3	4	3	4	4	4	4	4	4

304	5	5	5	5	4	3	4	5	4	4	4	5	4	5	5	5	4	5	5	5	5	5	5	5	5	5	5	5	5	5	4	5	5
305	5	5	5	4	5	5	4	5	5	5	4	5	4	5	5	5	4	5	4	4	5	4	4	4	4	4	5	5	4	5	4	5	4
306	1	1	1	1	2	2	1	1	1	1	1	2	1	2	1	3	2	3	3	4	3	1	1	3	4	2	1	4	1	1	4	4	3
307	5	5	5	5	5	5	5	4	4	3	3	5	5	5	5	5	4	5	4	4	5	5	5	3	4	5	3	5	5	5	5	5	5
308	5	4	5	5	5	5	4	4	5	5	3	5	5	5	5	5	5	4	4	5	4	5	5	4	3	4	4	5	5	4	5	5	5
309	4	4	4	4	5	5	4	3	4	5	4	4	4	4	5	4	5	5	4	4	5	5	4	3	5	5	4	5	4	5	4	5	5
310	5	5	5	2	3	4	3	2	3	1	4	4	3	3	4	4	3	5	3	4	3	5	5	5	4	5	4	3	5	5	3	5	2
311	5	5	5	5	5	5	4	5	5	5	5	5	4	5	5	5	5	5	4	5	5	5	5	5	3	5	3	4	5	5	4	4	4
312	5	4	4	5	4	3	4	3	4	3	4	3	5	5	5	4	4	5	4	3	4	4	3	4	3	4	4	4	4	5	4	4	4
313	5	5	4	4	5	4	4	5	4	3	5	4	5	4	4	5	4	5	3	2	4	4	4	3	3	3	3	4	4	4	4	4	3
314	4	4	3	4	4	3	4	2	2	4	2	2	3	4	4	2	4	4	3	3	3	2	3	2	2	3	3	4	4	3	3	5	3
315	4	3	5	5	4	5	4	5	5	3	5	4	5	5	4	5	4	5	4	4	5	5	5	4	5	5	5	4	5	5	4	5	5
316	4	3	4	3	3	3	3	4	4	3	4	2	4	3	5	4	3	4	4	3	3	4	3	2	4	4	3	4	4	3	4	5	4
317	5	5	5	5	4	5	4	5	5	5	4	5	5	5	5	5	4	5	5	5	5	5	5	5	4	5	5	5	5	5	5	5	5
318	3	3	4	3	4	3	3	3	4	3	3	3	4	3	4	3	3	4	3	3	4	3	3	4	3	4	3	3	3	3	3	3	3
319	4	4	4	5	4	5	4	4	4	4	4	4	4	4	4	5	5	5	4	5	4	5	5	4	4	4	4	5	4	5	4	4	4
320	4	4	5	5	5	4	4	2	4	2	4	4	3	3	4	5	3	5	5	5	4	5	2	4	5	4	4	5	5	4	4	5	5
321	5	5	5	5	5	5	5	4	5	5	5	5	5	5	5	4	5	5	5	5	5	5	5	5	5	5	5	5	5	5	5	5	5
322	4	3	4	3	4	4	4	4	3	4	4	4	4	4	2	4	3	4	3	4	4	3	2	3	1	2	3	3	4	3	4	4	4
323	5	5	5	5	5	4	4	5	5	4	4	5	5	5	5	5	5	5	1	5	5	5	5	5	5	5	5	5	5	5	5	5	5
324	5	5	5	5	5	5	4	5	5	4	4	5	5	5	4	5	4	5	4	5	5	4	5	5	5	4	5	5	5	4	4	5	5
325	5	4	5	5	5	5	5	5	5	3	4	5	5	5	5	5	5	5	5	5	5	5	5	5	5	5	5	5	5	5	3	2	3
326	5	4	5	5	5	5	5	5	5	3	4	5	5	5	5	5	5	5	5	5	5	5	5	5	5	5	5	5	5	4	3	2	2
327	5	5	5	4	4	4	3	3	4	5	5	5	5	4	5	5	5	5	4	5	4	4	4	5	5	5	5	5	4	5	4	5	4
328	5	4	5	4	4	4	4	4	4	4	3	4	4	5	5	4	4	5	4	5	5	5	4	4	4	4	4	4	4	5	4	5	4
329	5	5	5	5	5	4	5	4	4	3	3	5	5	5	5	5	5	5	5	5	5	5	5	5	5	5	5	5	4	4	5	5	4
330	5	5	4	4	5	5	4	4	5	4	3	4	4	4	4	5	5	5	3	5	5	5	5	5	3	4	5	5	5	4	4	5	5
331	4	5	4	5	5	4	4	5	4	4	4	4	5	5	4	4	4	4	5	5	4	5	5	5	5	5	5	4	4	5	5	5	5
332	5	5	5	5	4	4	5	5	5	4	4	5	5	4	5	5	5	5	5	5	5	4	4	4	5	5	5	4	4	5	5	4	4
333	5	4	5	5	5	5	4	5	5	5	4	5	4	4	5	5	5	4	5	5	5	5	5	4	5	5	5	5	5	5	5	5	5
334	5	5	4	4	4	4	5	5	5	5	4	5	5	5	5	5	5	5	5	5	5	5	4	4	4	5	4	5	5	5	5	5	5
335	5	4	5	5	4	3	4	3	3	5	5	4	4	4	5	5	4	5	5	5	5	4	5	5	2	4	4	5	5	5	4	5	3
336	4	3	4	4	5	5	3	3	4	2	4	3	3	4	5	5	4	5	5	4	5	4	4	4	4	5	3	5	4	4	3	5	3
337	5	5	5	5	5	5	4	5	5	5	5	4	5	4	5	4	5	5	5	4	5	4	5	5	4	5	3	5	5	5	5	5	5
338	5	4	3	2	4	4	3	2	3	2	4	4	2	2	3	4	4	5	5	5	4	2	3	3	1	2	2	3	3	4	2	4	4
339	4	5	5	5	4	5	4	4	5	5	5	4	5	5	5	5	5	5	5	5	5	5	5	5	5	5	5	5	5	5	5	5	5
340	5	5	5	5	5	5	4	4	5	5	5	5	5	5	5	5	5	5	5	5	5	5	5	5	5	5	5	5	5	5	5	5	5
341	5	4	5	4	4	3	3	4	5	3	2	5	5	5	4	5	5	5	5	5	5	5	4	4	4	4	4	5	5	4	4	5	4
342	3	3	4	5	5	4	3	5	5	5	1	4	5	5	5	5	5	5	4	5	5	2	2	5	4	4	5	4	5	5	4	5	5
343	4	5	5	4	4	4	4	4	4	4	4	4	3	5	5	5	5	5	3	5	4	3	4	4	5	4	4	3	3	3	4	4	4
344	4	5	5	4	5	5	4	4	5	5	5	5	3	4	5	5	4	5	3	5	4	5	5	5	5	5	4	4	4	4	4	5	4
