# Supplementary material for: Determinants of instructional leadership: A survey of public secondary schools
Source: PLoS One. 2026 Aug 14;21(8):e0355674. doi: 10.1371/journal.pone.0355674 (PMC13475869; doi:10.1371/journal.pone.0355674)
Supplement: Supplementary file 2 [file pone.0355674.s003.pdf]

PLOS ONE

Determinants of instructional leadership: A survey of of public secondary schools

--Manuscript Draft--

**Manuscript Number:**	PONE-D-25-14755
**Article Type:**	Research Article
**Full Title:**	Determinants of instructional leadership: A survey of of public secondary schools
**Short Title:**	Determinants of instructional leadership
**Corresponding Author:**	Suman Pande, MPhil<br>Kathmandu University School of Education<br>Kathmandu, Bagmati (Province 3) NEPAL
**Keywords:**	Instructional leadership; school; Head teacher; student; Academic Achievement
**Abstract:**	Instructional leadership is the leadership role associated with teaching and learning that academic heads must take on in order to demonstrate higher levels of student accomplishment. This study explores the factors influencing instructional leadership in Kathmandu, Nepal. Hallinger and Murphy (1985) model of instructional leadership is taken as the fundamental basis of this study. This model is subjected to Classical Delphi to develop a scale for this study. Exploratory factor analysis is conducted in the data to extract the factors. Three factors are formed and named as planning, managing and supervising instructional program; motivating and developing competence of teachers and students; and involving parents. The factors identified are supported by existing research. This research follows a post-positivist paradigm relying on statistical analysis of numerical data. The findings are valuable for school head teachers, schools' other stakeholders, concerned government officials of educational sector, and international researchers in instructional leadership.
**Order of Authors:**	Suman Pande, MPhil
	Laxman Acharya, PhD
	Prakash C Bhattarai, PhD
**Additional Information:**	
**Question**	**Response**
**Financial Disclosure**<br><br>Enter a financial disclosure statement that describes the sources of funding for the work included in this submission. Review the submission guidelines for detailed requirements. View published research articles from *PLOS ONE* for specific examples.<br><br>This statement is required for submission and **will appear in the published article** if the submission is accepted. Please make sure it is accurate.	The author(s) received no specific funding for this work.

Powered by Editorial Manager® and ProduXion Manager® from Aries Systems Corporation

**Funded studies**<br>Enter a statement with the following details:<br>• Initials of the authors who received each award<br>• Grant numbers awarded to each author<br>• The full name of each funder<br>• URL of each funder website<br>• Did the sponsors or funders play any role in the study design, data collection and analysis, decision to publish, or preparation of the manuscript?<br><br>Did you receive funding for this work?	
**Competing Interests**<br><br>Use the instructions below to enter a competing interest statement for this submission. On behalf of all authors, disclose any competing interests that could be perceived to bias this work—acknowledging all financial support and any other relevant financial or non-financial competing interests.<br><br>This statement is required for submission and **will appear in the published article** if the submission is accepted. Please make sure it is accurate and that any funding sources listed in your Funding Information later in the submission form are also declared in your Financial Disclosure statement.<br><br>View published research articles from *PLOS ONE* for specific examples.	The authors have declared that no competing interests exist.

Powered by Editorial Manager® and ProduXion Manager® from Aries Systems Corporation

**NO authors have competing interests**<br><br>Enter: *The authors have declared that no competing interests exist.*<br><br>**Authors with competing interests**<br><br>Enter competing interest details beginning with this statement:<br><br>*I have read the journal's policy and the authors of this manuscript have the following competing interests: [insert competing interests here]*<br><br>* typeset	
**Ethics Statement**<br><br>Enter an ethics statement for this submission. This statement is required if the study involved:<br><br>• Human participants<br>• Human specimens or tissue<br>• Vertebrate animals or cephalopods<br>• Vertebrate embryos or tissues<br>• Field research<br><br>Write "N/A" if the submission does not require an ethics statement.<br><br>General guidance is provided below. Consult the submission guidelines for detailed instructions. **Make sure that all information entered here is included in the Methods section of the manuscript.**	Ethical standards were maintained throughout the research process, including during data collection. Prior to initiating the study, ethical approval was obtained from the Research Committee of Kathmandu University, School of Education (Approval No. 181028-1). Recruitment period for pilot study was 24- 07- 2019 to 24- 08- 2019. Similarly, the recruitment period began in 25- 08- 2019 and concluded on 05- 02- 2020 for main study. Informed consent was secured from all participants. Head teachers, who served as respondents, were initially contacted by phone to request their time and contribution, while consent from the teacher respondents was obtained during school visits. A questionnaire with cover letter was distributed to each respondent. Some respondents provided data on the same day, while others scheduled a later time. Thus, the data was gathered in accordance with established ethical standards.

Powered by Editorial Manager® and ProduXion Manager® from Aries Systems Corporation

**Format for specific study types**<br><br>**Human Subject Research (involving human participants and/or tissue)**<br>• Give the name of the institutional review board or ethics committee that approved the study<br>• Include the approval number and/or a statement indicating approval of this research<br>• Indicate the form of consent obtained (written/oral) or the reason that consent was not obtained (e.g. the data were analyzed anonymously)<br><br>**Animal Research (involving vertebrate animals, embryos or tissues)**<br>• Provide the name of the Institutional Animal Care and Use Committee (IACUC) or other relevant ethics board that reviewed the study protocol, and indicate whether they approved this research or granted a formal waiver of ethical approval<br>• Include an approval number if one was obtained<br>• If the study involved *non-human primates*, add *additional details* about animal welfare and steps taken to ameliorate suffering<br>• If anesthesia, euthanasia, or any kind of animal sacrifice is part of the study, include briefly which substances and/or methods were applied<br><br>**Field Research**<br><br>Include the following details if this study involves the collection of plant, animal, or other materials from a natural setting:<br>• Field permit number<br>• Name of the institution or relevant body that granted permission	
**Data Availability**<br><br>Authors are required to make all data underlying the findings described fully available, without restriction, and from the time of publication. PLOS allows rare exceptions to address legal and ethical concerns. See the PLOS Data Policy and FAQ for detailed information.	Yes - all data are fully available without restriction

Powered by Editorial Manager® and ProduXion Manager® from Aries Systems Corporation

A Data Availability Statement describing where the data can be found is required at submission. Your answers to this question constitute the Data Availability Statement and **will be published in the article**, if accepted.<br><br>**Important:** Stating 'data available on request from the author' is not sufficient. If your data are only available upon request, select 'No' for the first question and explain your exceptional situation in the text box.<br><br>Do the authors confirm that all data underlying the findings described in their manuscript are fully available without restriction?	
**Describe where the data may be found in full sentences. If you are copying our sample text, replace any instances of XXX with the appropriate details.**<br><br>• If the data are **held or will be held in a public repository**, include URLs, accession numbers or DOIs. If this information will only be available after acceptance, indicate this by ticking the box below. For example: *All XXX files are available from the XXX database (accession number(s) XXX, XXX.).*<br>• If the data are all contained **within the manuscript and/or Supporting Information files**, enter the following: *All relevant data are within the manuscript and its Supporting Information files.*<br>• If neither of these applies but you are able to provide **details of access elsewhere**, with or without limitations, please do so. For example:<br><br>*Data cannot be shared publicly because of [XXX]. Data are available from the XXX Institutional Data Access / Ethics Committee (contact via XXX) for researchers who meet the criteria for access to confidential data.*<br><br>*The data underlying the results presented in the study are available from (include the name of the third party*	Data is with the authors and can be provided if necessary

and contact information or URL).<br>• This text is appropriate if the data are owned by a third party and authors do not have permission to share the data.<br><br>* typeset	
Additional data availability information:	Tick here if the URLs/accession numbers/DOIs will be available only after acceptance of the manuscript for publication so that we can ensure their inclusion before publication.; Tick here if your circumstances are not covered by the questions above and you need the journal's help to make your data available.

Manuscript

Click here to access/download;Manuscript;A paper on IL Anonymous.docx

Determinants of instructional leadership: A survey of public secondary schools

Suman Pande[1*], Laxman Acharya[2]¶, Prakash C Bhattarai[3]¶

[1]Department of Development Education, Kathmandu University, School of Education, Hattiban, Lalitpur, Nepal

[2]Department of Development Education, Kathmandu University, School of Education, Hattiban, Lalitpur, Nepal

[3]Department of Development Education, Kathmandu University, School of Education, Hattiban, Lalitpur, Nepal

*Corresponding author

E-mail: suman_devpdh2023@kusoed.edu.np (SP)

¶These authors contributed equally to this work

Acknowledgements: The authors express their gratitude to all those head teachers and teachers of public secondary schools of Kathmandu valley who provided data for this study. The authors would also like to acknowledge Kathmandu University faculty members who directly or indirectly helped to do this research.

Abstract

Instructional leadership is the leadership role associated with teaching and learning that academic heads must take on in order to demonstrate higher levels of student accomplishment. This study explores the factors influencing instructional leadership in Kathmandu, Nepal. Hallinger and Murphy (1985) model of instructional leadership is taken as the fundamental basis of this study. This model is subjected to Classical Delphi to develop a scale for this study. Exploratory factor analysis is conducted in the data to extract the factors. Three factors are formed and named as planning, managing and supervising instructional program; motivating and developing competence of teachers and students; and involving parents. The factors identified are supported by existing research. This research follows a post-positivist paradigm relying on statistical analysis of numerical data. The findings are valuable for school head teachers, schools' other stakeholders, concerned government officials of educational sector, and international researchers in instructional leadership.

Keywords

Instructional leadership, School, Head teacher, Student, Academic achievement

Introduction

The function of instructional leadership is vital in schools as it vastly influences the academic achievement of students [1, 2, 3]. In the schools of Nepal, the headteacher is only the academic leader who plays the role of instructional leader. In this regard, the head teachers are considered to be accountable for students' academic achievement. In Nepal, the policies for strengthening the role of the head teacher as an effective leader were enacted in Education Rule 2002 [4]. However, some instructional leadership roles were also defined, but they were grouped with administrative roles and not specifically recognized as instructional leadership responsibilities. The other policies formulated for the development of school education are Three Year Interim Plan (2007/08 -2009/10) [5] and other sequential Three-Year Interim Plans, School Sector Reform Plan (SSRP) (2009/10-2015/16) [6] School Sector Development Plan (SSDP) (2016/17- 2022/23) [7], National Education Policy, 2076 B.S. [8], School Education Sector Plan (SESP) (2022/23- 2031/32) [9]. In these policies, the policymakers have considered the leadership of the head teacher as a way to enhance students' academic progress. However, the instructional leadership role as the duty of head teacher has been mentioned clearly only in SSDP (2016/17- 2022/23) [7]. This demonstrates that the Ministry of Education within the Nepal Government has consistently worked to enhance Nepal's educational system. Still, the instructional leadership concept was introduced in the policy document only in 2016.

In spite of several efforts from the Nepal government to improve school education, satisfactory results have not been achieved by the public schools of Nepal for a long time. A review of the SLC exam results of Nepal from 1994 to 2013 showed that the average pass percentage of the SLC exam was only 45.50% over those 20 years' period [10]. After replacing the SLC with SEE and reforming the existing assessment system by changing the numerical grading system to a letter through the Education Act 2016, the result has become even worse.The SEE results of recent years have shown a continuous downfall in public schools [11]. The result of SEE, which is evaluated in the letter grading system, is not satisfactory in Nepal [12]. The study of SEE result data showed that only 45 % of students were over the satisfactory level in the academic year 2018 and 2019, however it reached to 78% in the academic year 2020 due to use of student scores from schools rather than center-based exams in the framework of Covid-19 [13]. In 2021 also, the SEE pass percentage increased as the board issued grade sheets based on internal evaluations conducted by individual schools in response to the pandemic [14]. In the

subsequent years, the results reverted to a similar trend, with only 47.86% of students passing the SEE exam in 2024 [15].

Unsatisfactory school performance over the years is evident from the SLC (School Leaving Certificate) results before the Education Act 2016, and SEE (Secondary Education Examination) results after the Education Act 2016 [16, 12, 17, 11, 18-22]. The unsatisfactory performance of public schools in Nepal is a blatant indication of the head teachers' inadequate effort in the teaching and learning process in these schools. What could be the cause of this failure? To determine the cause of any occurrence, it is necessary to understand the factors influencing the outcome. ~~In this regard, there seems an urgent need to know the factors affecting head teachers' role in teaching and learning, which is called the instructional leadership role. Although there is a large amount of literature on instructional leadership, a study to find the factors that affect the instructional leadership of head teachers in the local context of Nepal has not been found so far in the literature. Therefore, it seemed imperative to research the factors that influence the instructional leadership of head teachers. So, the research question developed for this study is, what factors affect head teachers' instructional leadership. Identifying the factors that influence instructional leadership would help head teachers identify where they are falling short and where they need to advance. This research is believed to be particularly helpful in helping head teachers build their professional abilities as effective instructional leaders. This study can improve the students' achievement in the classroom and supplement to what is already known about instructional leadership.~~

Reviewing the Literature

Researchers attempted to examine school effectiveness or program improvement in the late 1970s. They first identified the effective schools and then analyzed what made them effective. They noticed that a skillful head teacher was a key factor in accounting for successful change or school improvement [23]. This effective school research base assigned the role of instructional leader to the head teachers to develop their skills because it was found that a head teacher would seem to be capable of fulfilling the head teacher’s functions of managing the facility, controlling the operation, taking charge of community relations and overseeing the student progress only when he or she also becomes responsible for instructional leadership [24]. As a result, instructional leadership appeared as an essential construct for making schools effective, but it was still unclear what the actual instructional leadership roles are.

Instructional leadership is a term formed by combining "instruction" and "leadership." There have been multiple efforts to define instruction, but four key dimensions of instruction remain consistent: setting learning objectives, choosing teaching methods, selecting reinforcement activities, and evaluating outcomes [25]. Researchers hold varying views on ledership as well. Some have defined leadership as how a leader leads; others have described it as the job done through people [25]. Leadership includes followers acting toward goals that represent the values of the leaders and the followers [26]. Such uncertainty surrounding the concept of leadership has made the combined construct of “instructional leadership” difficult to define and clarify. The early instructional leadership models tended to focus on the leadership portion of the construct, and they attempted to define it in terms of general leadership theory. Those models focused on personality/ leadership style, behavioral/ situational or contextual components of the head teacher [24].

In this manner, the combined construct of "instructional leadership" has been defined in various ways. Instructional leadership means providing the necessary resources, possessing knowledge and skill in curriculum and instructional matters, being a skilled communicator and being a visionary who is out and around creating a visible presence [26]. Instructional leadership is direct assistance to teachers in their day-to-day activities, development of collaborative groups among staff, design and procurement of effective staff development, curriculum development, and the use of action research [27]. These definitions emphasized not only improving the instructional leadership role of the head teacher through the monitoring of instructional activities but also focused on the managerial task of the head teacher within it. Later scholars of instructional leadership began linking it to activities aimed at enhancing teaching and learning, leading to a slight shift in how instructional leadership was defined. Here are some examples. The instructional leadership begins when principals talk about being in the classroom, visit with students about their academic pursuits, and prioritise instruction [28]. Instructional leadership is important for effective practices in curriculum, instruction, and assessment and the ability to work with teachers on the day-to-day problems related to these topics [29]. The latter two perspectives on instructional leadership place a strong emphasis on teaching and learning activities. In this way the concept of instructional leadership evolved through various research developments, ultimately establishing that leadership roles connected to teaching and learning within the school fall under the domain of instructional leadership [30, 31, 2, 32].

The instructional leadership notion has dominated the educational literature since the creation of instructional leadership models, and there is ample proof of its effectiveness regarding teacher and student results [33, 34]. The notable models of instructional leadership have been proposed by various researchers [e.g., 35-41]. They have offered a variety of indications of instructional leadership, but the theoretical underpinnings are often the same [40, 41]. Researchers most extensively use the Hallinger and Murphy (1985) model of instructional leadership to do research on instructional leadership. The widespread acceptance of this model is primarily due to Hallinger and Murphy's (1985) core concept, which provides head teachers with valuable insights into all aspects of leadership related to teaching and learning in schools. The main theme of this model is to develop and communicate school goals, coordinate and supervise the school curriculum, monitor and evaluate student progress, encourage teachers and students, and maintain high visibility and a hands-on approach [23, 44]. Their model is in the form of a scale known as PIMRS (Principal/ Head Teacher Instructional Management Rating Scale). This PIMRS has been utilized in at least 130 PhD dissertations over almost three decades [45].

Several academics have mentioned their view on the characteristics of an instructional leader. The instructional leaders perform at high levels in four areas: a) resource provider, b) instructional resource, c) communicator, and d) visible presence in the school [35]. The instructional leaders emphasize on: a) goal establishment curriculum development, b) frequent observations, c) program evaluation, and d) support for teachers [41]. The instructional leader perform seven crucial functions,which are: a) establishing and implementing instructional goals; b) being available to staff; c) creating a school culture and climate conducive to learning; d) communicating the vision and mission of the school to students, staff, and parents; e) setting high expectations for the staff; f) developing teacher leaders; g) maintaining positive attitudes toward student, staff, and parents [39]. There are four tenets of an instructional leader: a) focus on developing, directing, and supervising the curriculum and instruction in the classroom; b) interact with their teachers to improve teaching and learning c) goal-oriented; d) establish high expectations and standards for teachers and students [38]. The effective instructional leader plays five important roles, which are: a) establishing goals and expectations; b) strategic resourcing; c) planning, coordinating, and evaluating teaching and the curriculum; d) promoting and participating in teacher learning and development; and e) ensuring an orderly and supportive environment [40]. There are six characteristics of effective instructional leaders: a) identify and

communicate the mission and vision of the school; b) maintain a school environment that is conducive to learning; c) promote the professional development of staff; d) monitor teachers' instruction; e) maintain a positive school climate and f) initiate organizational change [36]. From these scholars' view it can be inferred that an instructional leader's job is improving instruction to increase student achievement. Hence, an instructional leader is a person who seeks to ensure effective teaching and learning in the school by meeting all the requirements necessary to enhance students' academic performance.

Theoretical Underpinning

Among the models of instructional leadership proposed by various researchers devoted to the study of instructional leadership, Hallinger and Murphy's (1985) model of instructional leadership was used as a theoretical guide for this research because, this model has attained top priority and is more dominated in the literature of instructional leadership. This model of instructional leadership consists of three factors: defining the school's mission, managing the instructional program, and promoting a positive learning climate. There are two functions under the factor defining the school's mission, which are framing the school goals and communicating the school goals, and three functions under the factor managing the instructional program, which are supervising and evaluating instruction, coordinating curriculum, and monitoring students' progress, and five functions under the factor promoting a positive learning climate which are protecting instructional time; providing incentives for teachers; providing incentives for learning; promoting professional development of teachers; and maintaining high visibility. There are five items under each of these ten functions, forming fifty variables altogether.

These fifty items were studied thoroughly, and classical Delphi was conducted, followed by three round meetings with experienced head teachers and teachers to get their concrete ideas on it. Based on their opinion, some items, for example, (i) draw upon the results of class wise testing when making curriculum decisions, (ii) participate actively in the review of curricular materials, (iii) acknowledge teachers' exceptional performance by writing memos for their personal files, (iv) recognize superior students' achievement or improvement by seeing in the office the students with their work, (v) review student work products when evaluating classroom instruction were unselected from this PIMRS. Some items, for example, (i) head teacher announces mandatory presence of parents during terminal report card distribution, (ii) head teacher meets the parents of poor-performing students and shares with them what roles the

school and parents may together take for their improvements, (iii) head teacher learns students' family problems and talks to their parents to resolve them, (iv) head teacher is transparent about every financial detail of the school to all stakeholders were added in new PIMRS.

Some items were changed to best suit in Nepali schools, such as (i) monitoring the classroom curriculum to see that it covers the school's curricular objectives was changed to the head teacher checking teachers' log books regularly to see if they are going in accordance with the syllabus, (ii) limit interruption of instructional time by public address announcements was changed to head teacher tries his best to ensure uninterrupted instruction time, (iv) lead or attend teacher in- service activities concerned with instruction was changed to head teacher seeks to provide training to teachers during vacations or so as not to interrupt daily classes, (v) conduct informal observations in classrooms regularly was changed to head teacher regularly monitors activities of teachers and students while they are in class. In this way, finally a new PIMRS was developed for this research. This PIMRS consists of 11 factors and 33 items.

~~Research~~ Methods

As the study intends to find out the determining factors of instructional leadership, it is the study of a *cause*-and-effect relationship. It is because those factors can be found out by studying the causes that influence instructional leadership. Therefore, it follows the post-positivistic deterministic philosophy of *causes* determine *effects*. The objective reality of instructional leadership exists out there, and that reality can be found out through careful study and measurement. The research design is quantitative, and ~~research~~ methods include the preparation of questionnaires, data collection, data analysis, and interpretation. A well-structured questionnaire was prepared using the new PIMRS. The new PIMRS with 11 factors and 33 items asked the respondents to rate their view on a 5-point Likert scale with the descriptors; 1= almost never, 2= seldom, 3= sometimes, 4= frequently, and 5= almost always at which the head teacher was engaged in those particular activities of the school. The data was collected using a survey design, an appropriate way to get quantitative or numerical descriptions of population trends, attitudes, or opinions by surveying a sample of that population [46].

The population of this study includes all public secondary schools in Kathmandu Valley. Kathmandu Valley was chosen for the study because it has the greatest concentration of public secondary schools compared to any other region in the nation. In total, 303 public secondary schools in the Kathmandu Valley made up the population of this study and 172 schools made up

the sample size. 172 schools were chosen at random from a pool of 303 to visit to gather data. Data was collected from one teacher and the head teacher each from 172 schools to see the difference in head teachers' and teachers' ratings. One teacher from each school was chosen by the lottery process.

Ethical standards were maintained throughout the research process, including during data collection. Prior to initiating the study, ethical approval was obtained from the Research Committee of Kathmandu University, School of Education (Approval No. 181028-1). Recruitment period for pilot study was 24- 07- 2019 to 24-.08-2019. Similarly, the recruitment period began in 25- 08- 2019 and concluded on 05- 02- 2020 for main study. Informed consent was secured from all participants. Head teachers, who served as respondents, were initially contacted by phone to request their time and contribution, while consent from the teacher respondents was obtained during school visits. A questionnaire with cover letter was distributed to each respondent. Some respondents provided data on the same day, while others scheduled a later time. Thus, the data was gathered in accordance with established ethical standards.

The pilot testing of the questionnaire was done with the help of 30 head teachers and teachers from the schools apart from the randomly selected 172 schools. A default sample size of 30 is advised for questionnaire pilot testing [47]. The majority of the items' mean scores on the questionnaire's pilot test were determined to be more than four times the standard deviation. A low standard deviation indicates that the majority of the numbers are extremely close to the average, demonstrating the normal distribution. This ensured the reliability of the questionnaire [48]. The data was collected from 172 public schools of Kathmandu Valley with the help of this questionnaire, and the data were subjected to factor analysis to determine the factors influencing instructional leadership.

All the conditions required for factor analysis were checked. Factor analysis on Likert scale data is valued [49]. The criteria were met as a 5-point Likert- scale was used in this study. A lower number of categories cannot provide more possibilities and cannot help to obtain more reliable responses; on the other hand, a large range of scale impacts respondents' responses and reduces their chances of providing the proper answer on a specific item [50]. Therefore, I created a questionnaire using a 5-point Likert scale. The ratio of sample size-to-variable should be three to six [51]. Since the sample size is 172 and the number of variables is 39, this is within the

recommended range of 3:1 to 6:1. One of the most common situations is the retention of factors with Eigenvalues greater than one [52, 53]. In this study, all factors had Eigenvalues greater than 1, hence, this criterion was also met. This study also met the requirement of item loading .40 to .50 [54] as the item loading was .50.

Another condition is that the average commonalities or extraction value of items is more than 0.5 [54]. The average extraction value for items was .593. Therefore, this requirement was also fulfilled. Another criterion is Kaiser-Meyer sampling adequacy. If the KMO measure is greater than .50, it is safe to assume that the factor ability exists in the dataset [52, 55]. The Kaiser-Meyer sampling adequacy score was .896. So, this condition was also deemed met. A further requirement is that a factor must have at least three components to be considered genuine [56, 54]. This criterion was also fulfilled because there were three to fourteen items in each of the three instructional leadership factors. All the conditions for factor analysis were met, and three factors of instructional leadership were obtained. The Cronbach's alpha value of instructional leadership factors ranged between .75 to .92. The literature suggests that an alpha value of .75 to .95 is adequate [57]. Therefore, that was acceptable. Thus, the reliability of factor analysis was assessed.

Result

The factor analysis yielded 25 items under three factors. Fourteen of these twenty-five variables fall under factor one, eight under factor two, and three under factor three.

Factor One: Planning, managing and supervising instructional program

All the items of factor one are shown below in the rotated component matrix of this factor.

Table 1

Rotated Component Matrix of Factor One of IL

Components	1	2	3
1. Head teacher analyses students' performance of the last year while planning for the new academic session	.610		
2. Head teacher specifies school’s whole year educational plan in the presence of all concerned people	.652		
3. Head teacher seeks to incorporate teachers’ inputs in planning during his formal or informal meetings	.588		
4. Head teacher shares academic plans with everyone involved and discusses how those plans may be effectively implemented	.651		
5. Head teacher displays school’s whole year plan on the notice boards and communicates them to students during assembly	.639		
6. Head teacher reports educational planning of school to the local government and Province/Local level education offices	.604		
7. Head teacher regularly monitors activities of teachers and students while they are in class	.634		
8. Head teacher makes sure that students’ every class work/home work is completed timely and all notes are marked by assigned teachers at least a month prior to terminal examinations	.695		
9. Head teacher seeks to find out teachers’ areas of improvements in instructional practices	.711		
10. Head teacher gets every subject department head to implement the syllabus of all subjects in each class	.653		
11. Head teacher checks teachers’ log books regularly to see if they are going in accordance with the syllabus	.630		
12. Head teacher reviews whether curriculum goal is achieved through students’ terminal examination results, their discipline and overall change indicators	.664		
13. Head teacher regularly discusses with subject teachers regarding each student’s progress	.530		
6.2 Head teacher instructs teachers to ensure no student has bunked classes	.528		

These fourteen variables are connected to five distinct instructional leadership roles: planning and controlling school educational activities, overseeing and evaluating instruction, coordinating the curriculum and other educational objectives, keeping track of students' progress, and utilizing instructional time. These specific instructional leadership functions of the head teacher, except a last one, the head teacher directs teachers to make sure no student has skipped class, are similar to the items in Hallinger and Murphy's (1985) framework of IL falling under their two factors, defining the school mission, and managing instructional program [58-61]. Therefore, by including the meaning of those three leadership functions, namely defining the school mission, managing the instructional program, and ensuring no student has bunked the class, this factor was appropriately named as planning, managing, and supervising instructional program.

The factor planning, managing and supervising instructional program is about making an educational plan for the school, coordinating the curriculum, sharing plans, observing instructors to ensure they are practicing good teaching techniques, communicating with teachers to learn about their students' development, and making attempts to improve. There were two factors: framing and communicating school goals and supervising and evaluating instruction for these particular instructional leadership activities in Hallinger and Murphy's (1985) model [37]. They mentioned, it is about setting and expressing goals, working with staff members and teachers, and managing the instructional process and curriculum. It involves establishing the school's goals, reminding teachers of the purposes and mission, and informing teachers that teachers need to plan their classes properly [62]. A school lacks any standards to assess its success if it has no plan for improving the educational process. This factor is concerned with choosing the primary goals of the school as well as coordinating and managing curriculum and instruction [63]. Planning, managing, and supervising instructional program entails about articulating and expressing the school's goals, coordinating the curriculum, and monitoring and controlling the program by supervising and evaluating instruction [64].

Factor Two: Motivating and developing competence of teachers and students

All the items of factor two are shown below in the rotated component matrix of this factor.

Table 2

Rotated Component Matrix of Factor Two of IL

Components	1	2	3
1. Head teacher publicly praises teachers' superior performance but meets them in private for correction		.608	
2. Head teacher tries to manage opportunity for sincere teachers to work in a higher position as a reward for their good work		.668	
3. Head teacher provides letter of appreciation or honor certificates to teachers for their contribution		.725	
4. Head teacher seeks to provide training to teachers during vacations or so as not to interrupt daily classes		.735	
5. Head teacher actively supports teachers to use those learnt skills in the classrooms		.695	
6. Head teacher sometimes provides opportunities for observation or educational tour for teachers		.719	
7. Head teacher publicly honors students for their excellent performance or discipline		.489	
8. Head teacher learns students' family problems and talks to their parents to resolve them		.549	

Factor two consists of eight variables which explain the role of an instructional leader in motivating and supporting teachers and students for their academic growth. These eight variables serve similar functions to the variables in the Hallinger and Murphy's (1985) Framework of instructional leadership placed under the functions providing incentives for teachers, providing incentives for learning, and promoting professional development under the factor creating a positive school climate [58-61]. Since the obtained eight variables of this factor share the same aim of inspiring teachers and students for their development, a suitable name, 'motivating and developing the competence of teachers and students' was given to this factor.

The factor motivating and developing the competence of teachers and students is about encouraging teachers and students to develop their abilities and academic excellence. Praising and rewarding teachers and students for their good deeds, assisting teachers with their

professional growth, and supporting students in improving their self-confidence and academic focus are the key roles of an instructional leader, which are crucial in achieving academic success in the school. Recognizing and rewarding teachers' and students' accomplishments is important to develop interest in them [62]. These instructional leadership roles correspond to two functions in Hallinger and Murphy's (1985) instructional leadership model: promoting professional development, and offering incentives for learning. This factor focuses on inspiring and fostering teachers' and students' competence [64]. The overall focus is on helping both teachers and students get better.

Factor Three: Involving Parents

All the items of factor three are shown below in the rotated component matrix of this factor.

Table 3

Rotated Component Matrix of Factor Three of IL

Components			
	1	2	3
1. Head teacher calls class wise parents’ meeting and discusses student’s progress			.553
2. Head teacher announces mandatory presence of parents during terminal report card distribution			.839
3. Head teacher meets the parents of poor performing students and shares with them what roles the school and parents may together take for their improvements.			.653

The factor three of instructional leadership consists of three items. These items are linked to the head teachers' policy to get parents/guardians involved in knowing about their children's condition and taking the appropriate action if necessary. In accordance with the meaning of these variables, a suitable name involving parents was given to this factor.

The core idea summarized in this factor is to enhance parent-school communication for students' academic development. The parents/guardians are also made accountable along with the school through this strategy. Parental role is found to have given more importance to students'

academic progress in the Nepali context. Although there are studies [65, 66] that have shown a positive relationship between parental involvement and students' academic achievement in the international context as well, but the parent's involvement is not mentioned as a factor in the researchers' instructional leadership models whoever have proposed such model.

Discussion

The role of head teachers in engaging parents as one of the factors influencing head teachers' instructional leadership in the context of Nepal is a noteworthy finding because parental contribution to students' progress as one of the factors of instructional leadership has not been mentioned yet in any of the proposed instructional leadership models. Regular communication between head teachers and parents encourages parents to take more responsibility for their children's growth and to pay more attention to their educational needs. Increasing parental and community involvement in the educational process results in stronger relationships between school leaders and the community [67]. There is need of developing relational behavior for school administrators, including interaction with parents [68]. Furthermore, regular communication with parents assists head teachers in avoiding unwarranted complaints, as parents stay informed about their children's progress and collaborate with the school to encourage their children's development.

An Indonesian study, showed a positive correlation between parental participation and academic achievement in school [69]. He found that students with active parental involvement had higher test scores and grade point averages. Many studies have shown that parental participation positively influences students' educational performance. A favorable and modest impact of parental involvement on students' academic attainment was found in a meta-analysis of 37 research published between 2000 and 2013 in kindergarten, primary, and secondary schools [70]. However, some researchers found no relationship between parental involvement and students' accomplishments. A study conducted in Canada showed no significant results for the influence of parental participation on students' academic progress [71]. A study conducted in the United States of America involving 450 students from kindergarten to fourth grade, showed that the students who scored higher academically had lower parental involvement in school. Such findings imply that the country's cultural and societal institutions and practices may determine whether or not parental participation influences students' academic achievement [72].

Conclusion

The study concludes that there are three factors determining instructional leadership. The first factor, planning, managing and supervising instructional programs, explains the head teacher's role in collaborating with teachers to ensure that the school has a clear mission and that mission concentrates on students' academic achievement. The second factor, motivating and developing the competence of teachers and students describes the head teacher's activities to boost teachers' and students' interest in their line of work. The third factor, involving parents mentions the roles of parents/ guardians in association with school for students' academic progress. The study's key finding is that the parents'/ guardians' involvement in students' progress is seen as an essential factor in the schools of Nepal which is different from Hallinger and Murphy's (1985) model of instructional leadership. In Hallinger and Murphy's (1985) instructional leadership model, the parents'/guardians' involvement is not explicitly recognized as a factor in student success. Instead, the model includes only one related item, contacting parents to communicate improved or exemplary student performance or contributions, which falls under the factor "providing incentives for learning". This suggests that Nepal's unique cultural and societal framework places greater emphasis on the role of parents/ guardians in students' academic achievement.

Implications of the Study

The results of this study can help head teachers stay informed in their role as instructional leaders by providing recommendations for enhancing teaching and learning in schools**.** The finding can also assist policymakers and bureaucrats in formulating and implementing necessary policies to enhance head teachers' instructional leadership. Additionally, it can support local authorities in improving schools, as the responsibility for school development is entrusted to local bodies in Nepal's federal democratic system. In this way, the result of this study can be very useful for making schools effective and attaining quality education. Further, this study has added new knowledge to the construct of instructional leadership in the local Nepali context by identifying the factors that influence it, which may be of interest to international researchers of this field.

Acknowledgements The authors express their gratitude to all those head teachers and teachers of public secondary schools of Kathmandu valley who provided data for this study. The authors would also like to acknowledge Kathmandu University faculty members who directly or indirectly helped to do this research.

Funding

The authors have not got financial support for the research, authorship, and/or publication of this article.

Competing interests

The authors declare that they have no conflict of interest.

Ethical approval

The authors declare that they follow ethical standards

References

1. Bhujel, C. B. (2021).
   The role of principal in improvement of school performance: A qualitative study in community school of Nepal.
   Artech J. Art Social Sci., 3, 11-22.
2. Hallinger, P., & Murphy, J. (2012).
   Running on empty? Finding the time and capacity to lead learning.
   NASSP Bulletin, 97(1), 5-21.
3. Lamsal, B. (2022).
   Instructional leadership behavior among principals of two schools in Lalitpur, Nepal.
   TVET Journal, 1(16), 50 59.
4. Nepal Law Commission. (2002). *Education regulation 2002*.
   www.lawcommission.gov.np/en/ documents/2015/08/education-rules-2059-2002.pdf.
5. Nepal Planning Commission. (2007).
   Three-year interim plan (2007/08- 2009/10).
   Government of Nepal.
6. Ministry of Education. (2009).
   School sector reform plan, Nepal, 2009/10- 2015/16.
7. Ministry of Education. (2016).
   School sector development plan, Nepal, 2016/17- 2012/23.
8. Ministry of Education. (2019).
   National education policy 2076 (2019/ 020).
9. Ministry of Education, Science and Technology (2022).
   School Education Sector Plan, (2022/023-2031/032).
10. Bhattarai, Y. B, (2014).
    The school leaving certificate (SLC) examination of Nepal: Exploring negative consequences. [Master dissertation].
    Carleton University
11. Dixit, S. (2019). Seeing through SLC results.
    Setopati. https://en.setopati.com/view/149213

12. Chapagain, Y. (2021).
School student academic performance in Nepal: An analysis using the School Education Exam (SEE) results.
International Journal on Studies in Education, 3(1), 22-36.
13. Adhikari, P. (2020, August 8).
Abuse of rights in evaluation.
Gorkhapatra Daily.
https://old.gorkhapatraonline.com/epaper/view?paper=gorkhapatra&today=2020-08-26.
14. Sherma, A.B. (2023, August 13)
Did the SEE result genuinely improve this year?
Republica
https://myrepublica.nagariknetwork.com/news/did-the-see-result-genuinely-improve-this-year
15. Dhakal, M. (2024, June 28)
SEE results out: Over 52% students non- graded.
The Rising Nepal.
https://risingnepaldaily.com/news/44994
16. Budhathoki, C. B., Khatri, H.B., Shrestha, D. Rana, B. K., Sigdel, T.P., Panta, K., & Thapa, S.W. (2014).
Status of SLC dropouts and identifying ways to engaging students in co-curricular activities (Ed. David Hayes).
British Council.
17. Danai, R.P. (2021). Head teachers' instructional leadership at community schools.
AMC Journal, *2*(1), 55-73.
18. Koirala, A. (2015). Debate on public and private schools in Nepal.
International Journal of Social Sciences and Management, *2*(1), 3-8.
19. Pandey, A. (2015, June 18). Poor SLC results.
The Himalaya Times. https://thehimalayatimes.com/opinion/poor-slc-results/
20. Parajuli, D. R. & Das, T. (2013).
Performance of community schools in Nepal: A macro level analysis.
International Journal of Science and Technology Research, 2(7), 148-154.

21. Regmi, K.D. (2017).
World Bank in Nepal's education: Three decades of neoliberal reform.
Globalization, Societies and Education, 15(2), 188-201.
22. Thapa, A. (2015).
Public and private school performance in Nepal: An analysis using the SLC examination.
Education Economics, 23(1), 47-62.
23. Hallinger, P. (2003).
Leading educational change: Reflections on the practice of instructional and transformational leadership.
Cambridge Journal of Education, 33(3), 329- 351.
24. Uchtman, P. A. (1992).
Beyond the myths of instructional leadership: A study of teacher attitudes regarding the implementation of an organizational model of instructional leadership [Doctoral dissertation].
University of Kansas.
25. Irwin, C. C. (1985).
What research tells the teacher about instructional leadership.
https://eric.ed.gov/?id=ED257173
26. Smith, F., & Andrews, R. L. (1989).
Instructional leadership: How principals make a difference. [Report No. EA 021500], Association for Supervision and Curriculum Development.
27. Glickman, C. D., Gordon, S. P., & Ross-Gordon, J. M. (1995).
Supervision of instruction: A developmental approach (3rd ed.).
Allyn & Bacon.
28. Supovitz, J. A. (2000).
Manages less, leads more.
Principal Leadership, 1(3), 14-19.
29. Elmore, R. (2000).
Building a structure for school leadership.
Albert Shanker Institute.

30. Bush, T. (2007).
Educational leadership and management: Theory, policy, and practice.
South African Journal of Education, 27(3), 391-406.
31. Bush, T., & Glover, D. (2009).
Managing, teaching and learning (Concept paper).
Matthew Goniwe School of Leadership and Governance, South Africa.
32. Day, C., Gu, Q., & Sammons, P. (2016).
The impact of leadership on student outcomes: How successful school leaders use transformational and instructional strategies to make a difference.
Educational Administration, 52(2), 221– 258.
33. Hallinger, P., & Heck, R. H. (1998).
Exploring the principal's contribution to school effectiveness: 1980-1995.
School Effectiveness & School Improvement, 9(2), 157-191.
34. Ylimaki, R. M. (2007).
Instructional leadership in challenging US schools.
International Studies in Educational Administration, 35(3), 11-19.
35. Andrews, R., & Soder, R. (1987).
Principal instructional leadership and school achievement.
Educational Leadership, 44, 9-11.
36. Gulcan, M. (2012).
Research on instructional leadership competencies of school principals.
Education, 132, 625-635.
37. Hallinger, P., & Murphy J. (1985).
Assessing the instructional leadership behavior of principals.
Elementary School Journal, 86(2), 217-248.
38. Leithwood, K. (1994).
Leadership for school restructuring.
Educational Administration Quarterly, 30(4), 498-518.
39. McEwan, E. K. (1998).
Seven steps to effective instructional leadership.
Corwin Press.

40. Robinson, V. M. J., Lloyd, C. A., & Rowe, K. J. (2008).
The impact of leadership on student outcomes: An analysis of the differential effects of leadership types.
Educational Administration Quarterly, 44(5), 635–674.
41. Sheppard, B. (1996).
Exploring the transformational nature of instructional leadership.
Alberta Journal of Educational Research, 42(4), 325-344.
42. Burch, P. (2007).
The professionalization of instructional leadership in the United States: Competing values and current tensions.
Journal of Education Policy, 22(2), 195-214.
43. Reitzug, U. C. (2008).
Conceptualizing instructional leadership: The voices of principals.
Education and Urban Society, 40(6), 694-714.
44. Ovando, M., & Ramirez, A. (2007).
Principals' instructional leadership within a teacher performance appraisal system: Enhancing students' academic success.
Journal of Personnel Evaluation in Education, 20 (1-2), 85-110.
45. Hallinger, P. (2011).
A review of three decades of doctoral studies using the principal instructional management rating scale: A lens on methodological progress in educational leadership.
Educational Administration Quarterly, 47(2), 271–306.
46. Cresswell, J.W. (2012).
Educational research: Planning, conducting and evaluating quantitative and qualitative research (4th ed.).
Pearson.
47. Perneger T.V., Courvoisier, D.S., Hudelson, P.M., & Ageron, A. G. (2014).
Sample size for pre-tests of questionnaire.
Quality of Life Research, 1-6.

48. Kate, M. (2017).
What does a high standard deviation mean in real term?
Socratic.
https://socratic.org/questions/what-does-a-high-low-standard-deviation-mean-in-real-terms
49. Zaman, N., Bibi, Z., Sheikh, S. R., & Raziq, A. (2020).
Manualizing factor analysis of likert scale.
Journal of Management Sciences 7(2):56-67.
50. Tarka, P. (2015).
Likert scale and change in range of response categories. vs. the factors extraction in EFA model.
Acta Universitatis Lodziensis Folia Economica, 1(311), 27-37.
51. Cattell, R. B. (1978).
The scientific use of factor analysis.
Plenum.
52. Field, A. (2009).
Discovering statistics using SPSS (3rd ed.).
Sage.
53. Maskey, R., Fei, J., & Nguyen, H.O. (2018).
Use of exploratory factor analysis in maritime research.
The Asian Journal of Shipping and Logistics, 34, 91-111.
54. Tabachnick, B. G., & Fidell, L. S. (2007).
Using multivariate statistics (5th ed.).
Allyn & Bacon.
55. Zulkepli, M., Sipan, I., & Jibril, J. D. (2017).
An exploratory factor analysis and reliability analysis for green affordable housing criteria instrument.
An International Journal of Real Estate Studies, 11(4), 9-21.
56. Kline, P. (1994).
An essay guide to factor analysis.
Routledge

57. Tavacol, M., & Dennic, R. (2011).
Making sense of cronbach's alpha.
International Journal of Medical Education 2, 53-55.
58. Alhosani, H.M.A. (2015).
Principals' instructional leadership practices and their relationship to teachers' instructional practices in Sarjah schools. [Master dissertation].
United Arab Emirates University.
59. Lyons, B. J. (2010).
Principal instructional leadership behavior as perceived by teachers and principals at New York State recognized and non-recognized schools. [Doctoral dissertation].
Seton Hall University.
60. Peariso, J. F. (2011).
A study of principals' instructional leadership behaviours and beliefs of good pedagogical practice among effective California high schools serving socioeconomically disadvantaged and English learners. [Doctoral dissertation].
Liberty University.
61. Vilakaji, N.S. (2016).
Exploring principals' instructional leadership practices in primary schools: A case study of two underperforming township primary schools in Ekurhuleni South district, Gauteng province. [Master dissertation].
University of Witwatersrand.
62. Krug, S.E. (1992).
Instructional leadership: A constructivist perspective.
Educational Administration Quarterly, 28(3), 430-443.
63. Hallainger, P. (2005).
Instructional leadership and the school principal: A passing fancy that refuses to fade away.
Leadership and Policy in Schools, 4, 1-20.
64. Ghavifekr, S., Radwan, O., & Velarde J. M. (2019).
Teachers' perceptions of principal's instructional leadership roles and practices.
Journal Pendidikan Malaysia 44(2), 72- 83. http://dx.doi.org/ 10.17576/JPEN-2019-44.02-08.

65. Dahie, M. A., Mohamed, A.A., & Mohamed, R. A. (2018).
The role of parental involvement in student academic achievement: Empirical study from secondary schools in Mogadishu-Somalia.
International Research Journal of Human Resources and Social Sciences, *5*(7), 1-24.
66. Sapungan, G. M., & Sapungam, R. M. (2014).
Parental involvement in child's education: Importnce, barriers and benefits.
Asian Journal of Management Sciences and Education 3(2), 42-48.
67. Stronge, J. H., Richard, H. B., & Catano, N. (2008).
Qualities of effective principals.
Association for Supervision and Curriculum Development.
68. Northouse, P. G. (2015).
Introduction to leadership (3rd ed.).
Sage.
69. Utami, A.Y. (2022).
The role of parental involvement in student academic outcomes.
Journal of Education Review Provision, 2 (1), 17-21.
70. Castro, M., Exposito-Casas, E., Lopez-Martin, E., Lizasoain, L., Navarro-Asencio, E., Gaviria, J.L. (2015).
Parental involvement on student academic achievement: A meta-analysis.
Educational Research Review, 14, 33-46.
71. Epping, K.A. (2018).
The impact of parental involvement on student's academic achievement, parental well-being and parent-teacher relationships. [Master Dissertation].
University of Calgary.
72. Avnet, M., Makara, D., Larwin, K. H., & Erickson, M. (2019).
The impact of parental involvement and education on academic achievement in elementary school.
International Journal of Evaluation and Research in Education, 8 (3), 476-483.

Click here to access/download
Supporting Information
IL_Factors and items.docx
