# Supplementary material for: Determinants of instructional leadership: A survey of public secondary schools
Source: PLoS One. 2026 Aug 14;21(8):e0355674. doi: 10.1371/journal.pone.0355674 (PMC13475869; doi:10.1371/journal.pone.0355674)
Supplement: Supplementary file 3 [file pone.0355674.s006.pdf]

PLOS ONE

Determinants of instructional leadership: A survey of of public secondary schools

--Manuscript Draft--

**Manuscript Number:**	PONE-D-25-14755R1
**Article Type:**	Research Article
**Full Title:**	Determinants of instructional leadership: A survey of of public secondary schools
**Short Title:**	Determinants of instructional leadership
**Corresponding Author:**	Suman Pande, MPhil<br>Kathmandu University School of Education<br>Kathmandu, Bagmati (Province 3) NEPAL
**Keywords:**	Instructional leadership; school; Head teacher; Student; academic achievement; Factor
**Abstract:**	Instructional leadership is centered on teaching and learning that academic leaders undertake to promote higher levels of student learning. Given its importance, this study examines the factors influencing instructional leadership in Kathmandu, Nepal. The framework for the study is grounded in Hallinger and Murphy's (1985) model of instructional leadership, which was employed as the conceptual basis. Using the Classical Delphi method, the model was adapted to develop a measurement scale, while the study was situated within a post-positivist paradigm, assuming that the reality of instructional leadership exists independently. Exploratory factor analysis was then conducted to extract the underlying factors. The findings reveal three major factors influencing instructional leadership: (a) planning, managing, and supervising instructional programs; (b) motivating and developing the competence of teachers and students; and (c) involving parents in the learning process. The inclusion of parental involvement emerged as a novel dimension in the context of Nepal. These results provide meaningful insights for school headteachers, other educational stakeholders, policymakers in the government sector, and international researchers interested in instructional leadership.
**Order of Authors:**	Suman Pande, MPhil
	Laxman Acharya, PhD
	Prakash C Bhattarai, PhD
**Response to Reviewers:**	Comment Matrix<br>Comment by: Reviewer 1<br>S.No.Section where revision has been indicatedThe section presented before revision was advisedRevision suggestedThe section after revision was made according to the reviewers' comments<br>1.Literature ReviewResearchers attempted to examine school effectiveness or program improvement in the late 1970s. They first identified the effective schools and then analyzed what made them effective. They noticed that a skillful head teacher was a key factor in accounting for successful change or school improvement [23]. This effective school research base assigned the role of instructional leader to the head teachers to develop their skills because it was found that a head teacher would seem to be capable of fulfilling the head teacher's functions of managing the facility, controlling the operation, taking charge of community relations and overseeing the student progress only when he or she also becomes responsible for instructional leadership [24]. As a result, instructional leadership appeared as an essential construct for making schools effective, but it was still unclear what the actual instructional leadership roles are.<br>Instructional leadership is a term formed by combining "instruction" and "leadership." There have been multiple efforts to define instruction, but four key dimensions of instruction remain consistent: setting learning objectives, choosing teaching methods, selecting reinforcement activities, and evaluating outcomes [25]. Researchers hold varying views on ledership as well. Some have defined leadership as how a leader leads; others have described it as the job done through people [25]. Leadership includes followers acting toward goals that represent the values of the leaders and the followers [26]. Such uncertainty surrounding the concept of leadership has made the

Powered by Editorial Manager® and ProduXion Manager® from Aries Systems Corporation

combined construct of “instructional leadership” difficult to define and clarify. The early instructional leadership models tended to focus on the leadership portion of the construct, and they attempted to define it in terms of general leadership theory. Those models focused on personality/ leadership style, behavioral/ situational or contextual components of the head teacher [24].

In this manner, the combined construct of "instructional leadership" has been defined in various ways. Instructional leadership means providing the necessary resources, possessing knowledge and skill in curriculum and instructional matters, being a skilled communicator and being a visionary who is out and around creating a visible presence [26]. Instructional leadership is direct assistance to teachers in their day-to-day activities, development of collaborative groups among staff, design and procurement of effective staff development, curriculum development, and the use of action research [27]. These definitions emphasized not only improving the instructional leadership role of the head teacher through the monitoring of instructional activities but also focused on the managerial task of the head teacher within it. Later scholars of instructional leadership began linking it to activities aimed at enhancing teaching and learning, leading to a slight shift in how instructional leadership was defined. Here are some examples. The instructional leadership begins when principals talk about being in the classroom, visit with students about their academic pursuits, and prioritise instruction [28]. Instructional leadership is important for effective practices in curriculum, instruction, and assessment and the ability to work with teachers on the day-to-day problems related to these topics [29]. The latter two perspectives on instructional leadership place a strong emphasis on teaching and learning activities. In this way the concept of instructional leadership evolved through various research developments, ultimately establishing that leadership roles connected to teaching and learning within the school fall under the domain of instructional leadership [30, 31, 2, 32].

The instructional leadership notion has dominated the educational literature since the creation of instructional leadership models, and there is ample proof of its effectiveness regarding teacher and student results [33, 34]. The notable models of instructional leadership have been proposed by various researchers [e.g., 35, 36, 37, 38, 39, 40, 41]. They have offered a variety of indications of instructional leadership, but the theoretical underpinnings are often the same [40, 41]. Researchers most extensively use the Hallinger and Murphy (1985) model of instructional leadership to do research on instructional leadership. The widespread acceptance of this model is primarily due to Hallinger and Murphy's (1985) core concept, which provides head teachers with valuable insights into all aspects of leadership related to teaching and learning in schools. The main theme of this model is to develop and communicate school goals, coordinate and supervise the school curriculum, monitor and evaluate student progress, encourage teachers and students, and maintain high visibility and a hands-on approach [23, 44]. Their model is in the form of a scale known as PIMRS (Principal/ Head Teacher Instructional Management Rating Scale). This PIMRS has been utilized in at least 130 PhD dissertations over almost three decades [45].

Several academics have mentioned their view on the characteristics of an instructional leader. The instructional leaders perform at high levels in four areas: a) resource provider, b) instructional resource, c) communicator, and d) visible presence in the school [35]. The instructional leaders emphasize on: a) goal establishment curriculum development, b) frequent observations, c) program evaluation, and d) support for teachers [41]. The instructional leader perform seven crucial functions,which are: a) establishing and implementing instructional goals; b) being available to staff; c) creating a school culture and climate conducive to learning; d) communicating the vision and mission of the school to students, staff, and parents; e) setting high expectations for the staff; f) developing teacher leaders; g) maintaining positive attitudes toward student, staff, and parents [39]. There are four tenets of an instructional leader: a) focus on developing, directing, and supervising the curriculum and instruction in the classroom; b) interact with their teachers to improve teaching and learning c) goal-oriented; d) establish high expectations and standards for teachers and students [38]. The effective instructional leader plays five important roles, which are: a) establishing goals and expectations; b) strategic resourcing; c) planning, coordinating, and evaluating teaching and the curriculum; d) promoting and participating in teacher learning and development; and e) ensuring an orderly and supportive environment [40]. There are six characteristics of effective instructional leaders: a) identify and communicate the mission and vision of the school; b) maintain a school environment that is conducive to learning; c) promote the professional development of staff; d) monitor teachers’ instruction; e) maintain a positive school

Powered by Editorial Manager® and ProduXion Manager® from Aries Systems Corporation

climate and f) initiate organizational change [36]. From these scholars' view it can be inferred that an instructional leader's job is improving instruction to increase student achievement. Hence, an instructional leader is a person who seeks to ensure effective teaching and learning in the school by meeting all the requirements necessary to enhance students' academic performance.

Among the models of instructional leadership proposed by various researchers devoted to the study of instructional leadership, Hallinger and Murphy's (1985) model of instructional leadership was used as a theoretical guide for this research because, this model has attained top priority and is more dominated in the literature of instructional leadership. This model of instructional leadership consists of three factors: defining the school's mission, managing the instructional program, and promoting a positive learning climate. There are two functions under the factor defining the school's mission, which are framing the school goals and communicating the school goals, and three functions under the factor managing the instructional program, which are supervising and evaluating instruction, coordinating curriculum, and monitoring students' progress, and five functions under the factor promoting a positive learning climate which are protecting instructional time; providing incentives for teachers; providing incentives for learning; promoting professional development of teachers; and maintaining high visibility. There are five items under each of these ten functions, forming fifty variables altogether.

These fifty items were studied thoroughly, and classical Delphi was conducted, followed by three round meetings with experienced head teachers and teachers to get their concrete ideas on it. Based on their opinion, some items, for example, (i) draw upon the results of class wise testing when making curriculum decisions, (ii) participate actively in the review of curricular materials, (iii) acknowledge teachers' exceptional performance by writing memos for their personal files, (iv) recognize superior students' achievement or improvement by seeing in the office the students with their work, (v) review student work products when evaluating classroom instruction were unselected from this PIMRS. Some items, for example, (i) head teacher announces mandatory presence of parents during terminal report card distribution, (ii) head teacher meets the parents of poor-performing students and shares with them what roles the school and parents may together take for their improvements, (iii) head teacher learns students' family problems and talks to their parents to resolve them, (iv) head teacher is transparent about every financial detail of the school to all stakeholders were added in new PIMRS.

Some items were changed to best suit in Nepali schools, such as (i) monitoring the classroom curriculum to see that it covers the school's curricular objectives was changed to the head teacher checking teachers' log books regularly to see if they are going in accordance with the syllabus, (ii) limit interruption of instructional time by public address announcements was changed to head teacher tries his best to ensure uninterrupted instruction time, (iv) lead or attend teacher in- service activities concerned with instruction was changed to head teacher seeks to provide training to teachers during vacations or so as not to interrupt daily classes, (v) conduct informal observations in classrooms regularly was changed to head teacher regularly monitors activities of teachers and students while they are in class. In this way, finally a new PIMRS was developed for this research. This PIMRS consists of 11 factors and 33 items.

The manuscript is generally intelligible and written in standard English, though there are occasional grammatical errors and redundancies, particularly in the literature review and policy background sections Researchers began examining school effectiveness and program improvement in the late 1970s. They first identified effective schools and then analyzed what made them successful, noting that a skillful head teacher was a key factor in driving school improvement [23]. It was also emphasized that instructional leadership plays a pivotal role in fostering school improvement, promoting better academic outcomes both directly, by supporting teachers, and indirectly, by improving conditions for teaching and learning. Successful school leadership involves improving conditions for teaching and learning, redesigning and enriching the curriculum, redesigning the rolesw and responsibilities, enhancing teacher quality, building relationships inside the school and community, enhancing teaching and learning and building relationship outside the school community [24].

Powered by Editorial Manager® and ProduXion Manager® from Aries Systems Corporation

Instructional leadership is a construct derived form the combination of the concepts of "instruction" and "leadership." There have been multiple efforts to define instruction, but four key dimensions of instruction remain consistent: setting learning objectives, choosing teaching methods, selecting reinforcement activities, and evaluating outcomes [25]. Researchers hold varying views on ledership as well. Some have defined leadership as how a leader leads; others have described it as the job done through people [25]. Leadership includes followers acting toward goals that represent the values of the leaders and the followers [26]. Such uncertainty surrounding the concept of leadership has made the combined construct of "instructional leadership" difficult to define and clarify. The early instructional leadership models tended to focus on the leadership portion of the construct, and they attempted to define it in terms of general leadership theory. Those models focused on personality/leadership style, behavioral/situational, or contextual components of the head teacher [24].

In this manner, the combined construct of "instructional leadership" has been defined in various ways. Instructional leadership means providing the necessary resources, possessing knowledge and skill in curriculum and instructional matters, being a skilled communicator, and being a visionary who is out and around creating a visible presence [26]. Instructional leadership is direct assistance to teachers in their day-to-day activities, the development of collaborative groups among staff, the design and procurement of effective staff development, curriculum development, and the use of action research [27]. These definitions emphasized not only improving the instructional leadership role of the head teacher through the monitoring of instructional activities but also focused on the managerial tasks of the head teacher within it. Later scholars of instructional leadership began linking it to activities aimed at enhancing teaching and learning, leading to a slight shift in how instructional leadership was defined. Here are some examples: instructional leadership begins when principals talk about being in the classroom, visit with students about their academic pursuits, and prioritize instruction [28]. Instructional leadership is important for effective practices in curriculum, instruction, and assessment, and for the ability to work with teachers on the day-to-day problems related to these topics [29]. The latter two perspectives on instructional leadership place a strong emphasis on teaching and learning activities. In this way, the concept of instructional leadership evolved through various research developments, ultimately establishing that leadership roles connected to teaching and learning within the school fall under the domain of instructional leadership [30, 31, 2, 32].

The instructional leadership notion has dominated the educational literature since the creation of instructional leadership models, and there is ample proof of its effectiveness regarding teacher and student results [33, 34]. The notable models of instructional leadership have been proposed by various researchers [e.g.,35, 36, 37, 38, 39, 40, 41]. They have offered a variety of indications of instructional leadership, but the theoretical underpinnings are often the same [40, 41]. Researchers most extensively use the Hallinger and Murphy (1985) model of instructional leadership to conduct research on instructional leadership. The widespread acceptance of this model is primarily due to Hallinger and Murphy's (1985) core concept, which provides head teachers with valuable insights into all aspects of leadership related to teaching and learning in schools. The main theme of this model is to develop and communicate school goals, coordinate and supervise the school curriculum, monitor and evaluate student progress, encourage teachers and students, and maintain high visibility and a hands-on approach [23, 44]. Their model is in the form of a scale known as PIMRS (Principal/Head Teacher Instructional Management Rating Scale). This PIMRS has been utilized in at least 130 PhD dissertations over almost three decades [45].

Several academics have mentioned their views on the characteristics of an instructional leader. I Instructional leaders perform at high levels in four areas: a) resource provider, b) instructional resource, c) communicator, and d) visible presence in the school [35]. The instructional leaders emphasize: a) goal establishment and curriculum development, b) frequent observations, c) program evaluation, and d) support for teachers [41]. Instructional leaders perform seven crucial functions,which are: a) establishing and implementing instructional goals; b) being available to staff; c) creating a school culture and climate conducive to learning; d) communicating the vision and mission of the school to students, staff, and parents; e) setting high expectations for the staff; f) developing teacher leaders; g) maintaining positive attitudes toward student, staff, and parents [39]. There are four tenets of an instructional leader: a) focus on developing, directing, and supervising the curriculum and instruction in the classroom; b) interacting with teachers to improve teaching and learning c) being goal-oriented; d) establish high expectations and standards for

Powered by Editorial Manager® and ProduXion Manager® from Aries Systems Corporation

teachers and students [38]. The effective instructional leader plays five important roles, which are: a) establishing goals and expectations; b) strategic resourcing; c) planning, coordinating, and evaluating teaching and the curriculum; d) promoting and participating in teacher learning and development; and e) ensuring an orderly and supportive environment [40]. There are six characteristics of effective instructional leaders: a) identifying and communicating the mission and vision of the school; b) maintain a school environment that is conducive to learning; c) promote the professional development of staff; d) monitor teachers' instruction; e) maintain a positive school climate and f) initiate organizational change [36]. From these scholars' views, it can be inferred that an instructional leader's job is improving instruction to increase student achievement. Hence, an instructional leader is a person who seeks to ensure effective teaching and learning in the school by meeting all the requirements necessary to enhance students' academic performance.

Among the models of instructional leadership proposed by various researchers devoted to the study of instructional leadership, Hallinger and Murphy's (1985) model of instructional leadership was used as a theoretical guide for this research because this model has attained top priority and is more dominat in the literature on instructional leadership. This model of instructional leadership consists of three factors: defining the school's mission, managing the instructional program, and promoting a positive learning climate. There are two functions under the factor defining the school's mission, which are framing the school goals and communicating the school goals; three functions under the factor managing the instructional program, which are supervising and evaluating instruction, coordinating curriculum, and monitoring students' progress; and five functions under the factor promoting a positive learning climate which are protecting instructional time, providing incentives for teachers, providing incentives for learning, promoting professional development of teachers, and maintaining high visibility. There are five items under each of these ten functions, forming fifty variables altogether.

These fifty items were studied thoroughly, and a classical Delphi was conducted, followed by three rounds of meetings with experienced head teachers and teachers to get their concrete ideas on it. Based on their opinions, some items- for example, (i) draw upon the results of class-wise testing when making curriculum decisions, (ii) participate actively in the review of curricular materials, (iii) acknowledge teachers' exceptional performance by writing memos for their personal files, (iv) recognize superior students' achievement or improvement by seeing in the office the students with their work, (v) review student work products when evaluating classroom instruction- were deselected from this PIMRS. Some items- for example, (i) head teacher announces mandatory presence of parents during terminal report card distribution, (ii) head teacher meets the parents of poor-performing students and shares with them what roles the school and parents may together take for their improvements, (iii) head teacher learns students' family problems and talks to their parents to resolve them, (iv) the head teacher is transparent about every financial detail of the school to all stakeholders- were added to the in new PIMRS.

Some items were changed to best suit Nepali schools, such as (i) monitoring the classroom curriculum to see that it covers the school's curricular objectives was changed to the head teacher checking teachers' log books regularly to see if they are going in accordance with the syllabus, (ii) limiting interruption of instructional time by public address announcements was changed to head teacher trying his best to ensure uninterrupted instructional time, (iii) leading or attending teacher in- service activities concerned with instruction was changed to head teacher seeking to provide training to teachers during vacations so as not to interrupt daily classes, (v) conducting informal observations in classrooms regularly was changed to head teacher regularly monitoring the activities of teachers and students while they are in class. In this way, a new PIMRS was finally developed for this research. This PIMRS consists of 11 factors and 33 items.

Comment by: Reviewer 1

2.   Result   The factor analysis yielded 25 items under three factors. Fourteen of these twenty-five variables fall under factor one, eight under factor two, and three under factor three.

Factor One: Planning, managing and supervising instructional program

All the items of factor one are shown below in the rotated component matrix of this factor.

Powered by Editorial Manager® and ProduXion Manager® from Aries Systems Corporation

Table 1
Rotated Component Matrix of Factor One of IL

These fourteen variables are connected to five distinct instructional leadership roles: planning and controlling school educational activities, overseeing and evaluating instruction, coordinating the curriculum and other educational objectives, keeping track of students' progress, and utilizing instructional time. These specific instructional leadership functions of the head teacher, except a last one, the head teacher directs teachers to make sure no student has skipped class, are similar to the items in Hallinger and Murphy's (1985) framework of IL falling under their two factors, defining the school mission, and managing instructional program [58-61]. Therefore, by including the meaning of those three leadership functions, namely defining the school mission, managing the instructional program, and ensuring no student has bunked the class, this factor was appropriately named as planning, managing, and supervising instructional program.

The factor planning, managing and supervising instructional program is about making an educational plan for the school, coordinating the curriculum, sharing plans, observing instructors to ensure they are practicing good teaching techniques, communicating with teachers to learn about their students' development, and making attempts to improve. There were two factors: framing and communicating school goals and supervising and evaluating instruction for these particular instructional leadership activities in Hallinger and Murphy's (1985) model [37]. They mentioned, it is about setting and expressing goals, working with staff members and teachers, and managing the instructional process and curriculum. It involves establishing the school's goals, reminding teachers of the purposes and mission, and informing teachers that teachers need to plan their classes properly [62]. A school lacks any standards to assess its success if it has no plan for improving the educational process. This factor is concerned with choosing the primary goals of the school as well as coordinating and managing curriculum and instruction [63]. Planning, managing, and supervising instructional program entails about articulating and expressing the school's goals, coordinating the curriculum, and monitoring and controlling the program by supervising and evaluating instruction [64].

Factor Two: Motivating and developing competence of teachers and students

All the items of factor two are shown below in the rotated component matrix of this factor.

Table 2
Rotated Component Matrix of Factor Two of IL

Factor two consists of eight variables which explain the role of an instructional leader in motivating and supporting teachers and students for their academic growth. These eight variables serve similar functions to the variables in the Hallinger and Murphy's (1985) Framework of instructional leadership placed under the functions providing incentives for teachers, providing incentives for learning, and promoting professional development under the factor creating a positive school climate [58-61]. Since the obtained eight variables of this factor share the same aim of inspiring teachers and students for their development, a suitable name, 'motivating and developing the competence of teachers and students' was given to this factor.

The factor motivating and developing the competence of teachers and students is about encouraging teachers and students to develop their abilities and academic excellence. Praising and rewarding teachers and students for their good deeds, assisting teachers with their professional growth, and supporting students in improving their self-confidence and academic focus are the key roles of an instructional leader, which are crucial in achieving academic success in the school. Recognizing and rewarding teachers' and students' accomplishments is important to develop interest in them [62]. These instructional leadership roles correspond to two functions in Hallinger and Murphy's (1985) instructional leadership model: promoting professional development, and offering incentives for learning. This factor focuses on inspiring and fostering teachers' and students' competence [64]. The overall focus is on helping both teachers and students get better.

Factor Three: Involving Parents

All the items of factor three are shown below in the rotated component matrix of this factor.

Powered by Editorial Manager® and ProduXion Manager® from Aries Systems Corporation

Table 3
Rotated Component Matrix of Factor Three of IL
The factor three of instructional leadership consists of three items. These items are linked to the head teachers' policy to get parents/guardians involved in knowing about their children's condition and taking the appropriate action if necessary. In accordance with the meaning of these variables, a suitable name involving parents was given to this factor.
The core idea summarized in the variables of this factor is to enhance parent/guardian-school communication for students' academic development. The parents/guardians are also made accountable along with the school through this strategy. Parental role is found to have given more importance to students' academic progress in the Nepali context. Although there are studies [65, 66] that have shown a positive relationship between parental involvement and students' academic achievement in the international context as well, but the parent's involvement is not mentioned as a factor in the researchers' instructional leadership models whoever have proposed such model.

The structure of the results section could be improved with clearer subheadings, and some sentences would benefit from greater clarity and conciseness. Terms such as "post-positivist paradigm" should be defined for broader accessibility.The factor analysis yielded three instructional leadership factors. Fourteen items loaded on the first factor, eight on the second, and three on the third.
Factor One: Planning, managing and supervising instructional program
All the items of factor one are shown below in the rotated component matrix of this factor.
Table 1
Rotated Component Matrix of Factor One of IL

Explanation of Variables in Factor One
These fourteen variables are connected to five distinct instructional leadership roles: planning and controlling school educational activities, overseeing and evaluating instruction, coordinating the curriculum and other educational objectives, keeping track of students' progress, and utilizing instructional time. These specific instructional leadership functions of the head teacher, except the head teacher directs teachers to make sure no student has skipped class, are similar to the items in Hallinger and Murphy's (1985) framework of IL falling under their two factors, defining the school mission, and managing instructional program [58, 59, 60, 61]. Therefore, by including the meaning of those three leadership functions, namely defining the school mission, managing the instructional program, and ensuring no student has bunked the class, this factor was appropriately named as planning, managing, and supervising the instructional program. All the variables of the factor planning, managing, and supervising instructional program show that it is about making an educational plan for the school, coordinating the curriculum, sharing plans, observing instructors to ensure they are practicing good teaching techniques, communicating with teachers to learn about their students' development, and making attempts to improve. There were two factors: framing and communicating school goals, and supervising and evaluating instruction for these particular instructional leadership activities in Hallinger and Murphy's (1985) model [37]. They mentioned, it is about setting and expressing goals, working with staff members and teachers, and managing the instructional process and curriculum. According to [62] this involves establishing the school's goals, reminding teachers of its purposes and mission, and informing teachers that teachers need to plan their classes properly. A school lacks any standards to assess its success without a plan to improve the educational process. As stated by [63] this factor relates to selecting the schools' primary goals as well as coordinating and managing curriculum and instruction. Similarly, [64] notes that planning, managing, and supervising instructional programs entails articulating and expressing the school's goals, coordinating the curriculum, and monitoring and controlling the program by supervising and evaluating instruction.
This factor also resonates with the conceptualization of instructional leadership by [65], which is grounded in head teachers' core practices and was developed to explain how

Powered by Editorial Manager® and ProduXion Manager® from Aries Systems Corporation

Norwegian school leaders build their instructional capacity. Their multidimensional model consisting of goal orientation, collaboration with teachers, professional development, observation, and supervision is similar to the variables of the factor planning, managing and supervising instructional program identified in this study. Both undersore head teachers' active role in shaping and monitoring instructional processes as a foundation for improving teaching and learning.

This finding can also be compared with those [66], where coordinating the curriculum and providing professional learning opportunities to teachers emerged as statistically significant predictors of teachers' professional development in the context of Nigeria. Coordinating the curriculum is parallel to the variable "the head teacher gets every subject department heads to implement the syllabus of all subjects in each class" within the factor of planning, managing and supervising instructional program. This reinforces the importance of this variable for developing teaching and learning ability. The next predictor, providing professional learning opportunities to teachers, corroborates the variables of the second factor, motivating and developing competence of teachers and students developed in this study.

Factor Two: Motivating and developing competence of teachers and students

All the items of factor two are shown below in the rotated component matrix of this factor.

Table 2

Rotated Component Matrix of Factor Two of IL

Explanation of Variables in Factor Two

Factor two consists of eight variables which explain the role of an instructional leader in motivating and supporting teachers and students for their academic growth. These eight variables serve similar functions to the variables in the Hallinger and Murphy's (1985) Framework of instructional leadership placed under the functions providing incentives for teachers, providing incentives for learning, and promoting professional development under the factor creating a positive school climate [58, 59, 60, 61]. Since the obtained eight variables of this factor share the same aim of inspiring teachers and students for their development, a suitable name, ‘motivating and developing competence of teachers and students’ was given to this factor.

All the variables of the factor motivating and developing the competence of teachers and students show that it is about encouraging teachers and students to develop their abilities and academic excellence. Praising and rewarding teachers and students for their good deeds, assisting teachers with their professional growth, and supporting students in improving their self-confidence and academic focus are the key roles of an instructional leader, which are crucial in achieving academic success in the school. As stated by [62], recognizing and rewarding teachers' and students' accomplishments is important for fostering their interest and engagement . These instructional leadership roles align withtwo functions in Hallinger and Murphy's (1985) instructional leadership model, promoting professional development, and providing incentives for learning. According to [64], this factor emphasizes on inspiring and enhancing the competence of both teachers and students . The overall focus is on helping both teachers and students get better.

The variables within this factor, such as offering training to teachers during vacations, supporting the classroom application of acquired skills, and facilitating educational tours corroborate the finding of [66] who found that providing professional learning opportunities to teachers supports their professional development. In contrast to the finding of [66] who did not find providing incentives for teachers to be a significant predictor of teachers' professional development, the present study suggests that motivational strategies such as public praise, appreciation letters, honor certificates, and rewarding sincere teachers with opportunities for career development are integral to strengthening teachers' competence. Additionally, the inclusion of student focused practices such as honoring excellent performance and addressing family problems reflects a broader and more relational approach for both teachers' and learners' holistic development.

Factor Three: Involving Parents

All the items of factor three are shown below in the rotated component matrix of this factor.

Table 3

Rotated Component Matrix of Factor Three of IL

Explanation of Variables in Factor Three

The factor three of instructional leadership consists of three items. These items are

Powered by Editorial Manager® and ProduXion Manager® from Aries Systems Corporation

linked to the head teachers' policy to get parents/guardians involved in knowing about their children's condition and taking the appropriate action if necessary. In accordance with the meaning of these variables, a suitable name involving parents was given to this factor. The core idea summarized in the variables of this factor is to enhance parent/guardian-school communication for students' academic development. The parents/guardians are also made accountable along with the school through this strategy. Parental role is found to be more important to students' academic progress in the Nepali context. There are studies that have shown importance of the parent-school relationship. For example, some studies [67 , 68] have shown a positive relationship between parental involvement and students' academic achievement, [69] reported that the increasing parental and community involvement in the educational process results in stronger relationships between school leaders and the community. Similarly, [70] emphasized the need for developing relational behavior for school administrators, including interaction with parents. A recent research [71] conducted in Hong Kong, and Macao also demonstrated that leadership can sinfluence student achievement through Parental Academic Commitment (PAC). This reinforces the idea that parent involvement is not just a peripheral activity but a leadership-mediated pathway to better student outcomes.

Comment by: Reviewer 1
3.Abstract & Research MethodsThis research follows a post-positivist paradigm relying on statistical analysis of numerical data.

Terms such as "post-positivist paradigm" should be defined for broader accessibility. Overall, the manuscript communicates its findings effectively, but a thorough language and style revision is recommended to address minor issues and improve readability.The objective reality of instructional leadership exists out there, and that reality can be found through careful study and measurement. Although the reality of instructional leadership exists independently, it can only be understood through data and evidence. Therefore, this study relies on statistical analysis of numerical data, a method known as quantitative research.
Language and style revision is also done

Comment by: Reviewer 1
4.Regarding data availabilityRegarding data availability, the manuscript states that data are with the authors and can be provided if necessary. However, this does not fully comply with PLOS ONE's data policy, which requires that all data underlying the findings be made publicly available without restriction. The authors should deposit the anonymized dataset in a public repository or provide it as supplementary information to ensure compliance with journal requirements.Data will be submitted along with the manuscript and other required files

Comment by: Reviewer 2
5.Regarding output files of factor analysisThe authors should provide the outputs of the

Powered by Editorial Manager® and ProduXion Manager® from Aries Systems Corporation

factor analysis, which was completed using the Statistical Package for Social Sciences 29.0. Based on the information provided regarding the factor loadings of the questionnaire items, reviewers need to verify the factor loadings outputs to ensure the data analysis's accuracy.The output files of factor analysis will be attached

Comment

by: Reviewer 3

6.AbstractInstructional leadership is the leadership role associated with teaching and learning that academic heads must take on in order to demonstrate higher levels of student accomplishment. This study explores the factors influencing instructional leadership in Kathmandu, Nepal. Hallinger and Murphy (1985) model of instructional leadership is taken as the fundamental basis of this study. This model is subjected to Classical Delphi to develop a scale for this study. Exploratory factor analysis is conducted in the data to extract the factors. Three factors are formed and named as planning, managing and supervising instructional program; motivating and developing competence of teachers and students; and involving parents. The factors identified are supported by existing research. This research follows a post-positivist paradigm relying on statistical analysis of numerical data. The findings are valuable for school head teachers, schools' other stakeholders, concerned government officials of educational sector, and international researchers in instructional leadership.

Keywords

Instructional leadership, School, Head teacher, Student, Academic achievement

The abstract and keywords do not provide adequate information about the article. Although the objective and topic addressed in the manuscript are mentioned, the research methodology, main results, and conclusions found in the study are missing.Instructional leadership is centered on teaching and learning that academic leaders undertake to promote higher levels of student learning. Given its importance, this study examines the factors influencing instructional leadership in Kathmandu, Nepal. The framework for the study is grounded in Hallinger and Murphy's (1985) model of instructional leadership, which was employed as the conceptual basis. Using the Classical Delphi method, the model was adapted to develop a measurement scale, while the study was situated within a post-positivist paradigm, assuming that the reality of instructional leadership exists independently. Exploratory factor analysis was then conducted to extract the underlying factors. The findings reveal three major factors influencing instructional leadership: (a) planning, managing, and supervising instructional programs; (b) motivating and developing the competence of teachers and students; and (c) involving parents in the instructional process. The inclusion of parental involvement emerged as a novel dimension in the context of Nepal. These results provide meaningful insights for school headteachers, other educational stakeholders, policymakers in the government sector, and international researchers interested in instructional leadership.

Keywords

Instructional leadership, School, Head teacher, Student, Academic achievement, Factors

Comment

by: Reviewer 3

7.ReferenceCheck whether all authors cited in the article are included in the manuscript's references, and vice versa.Checked

Comment

by: Reviewer 3

8.

IntroductionUnsatisfactory school performance over the years is evident from the SLC (School Leaving Certificate) results before the Education Act 2016, and SEE (Secondary Education Examination) results after the Education Act 2016 [16, 12, 17, 11, 18-22]. The unsatisfactory performance of public schools in Nepal is a blatant indication of the head teachers' inadequate effort in the teaching and learning process in these schools. What could be the cause of this failure? To determine the cause of any occurrence, it is necessary to understand the factors influencing the outcome. In this regard, there seems an urgent need to know the factors affecting head teachers' role in teaching and learning, which is called the instructional leadership role. Although there is a large amount of literature on instructional leadership, a study to find the factors that affect the instructional leadership of head teachers in the local context of Nepal has not been found so far in the literature. Therefore, it seemed imperative to research the factors that influence the instructional leadership of head teachers. So,

the research question developed for this study is, what factors affect head teachers' instructional leadership. Identifying the factors that influence instructional leadership would help head teachers identify where they are falling short and where they need to advance. This research is believed to be particularly helpful in helping head teachers build their professional abilities as effective instructional leaders. This study can improve the students' achievement in the classroom and supplement to what is already known about instructional leadership.5. The article has issues with clarity and coherence in its language, which need to be revised. The Introduction doesn't clarify the exact objective of the research (it differs from the objective stated in the Abstract) and the central research question. Please review this.Unsatisfactory school performance over the years is evident from the SLC (School Leaving Certificate) results before the Education Act 2016, and SEE (Secondary Education Examination) results after the Education Act 2016 [16, 12, 17, 11, 18, 19, 20, 21,22]. The unsatisfactory performance of public schools in Nepal is a blatant indication of the head teachers' inadequate effort in the teaching and learning process in these schools, a responsibility that falls under their instructional leadership role.. In this regard, there is an urgent need to identify the influencing factors of head teachers' instructional leadership. Although there are a number of literature on instructional leadership, a study to find the factors that affect the instructional leadership of head teachers in the local context of Nepal has not been found so far in the literature. Therefore, it was deemed imperative to identify the factors affecting head teachers' instructional leadership. So, the research question developed for this study is, what are the factors that affect head teachers' instructional leadership. Identifying the factors affecting head teachers' instructional leaderahip would help head teachers recognize areas where they are falling short and where they need to improve. This research is expected to support head teachers developing their professional abilities as effective instructional leaders, which, in turn, can enhance students' achievement in schools. Furthermore, the finding of this study can contribute to the existing body of knowlwdge on instructional leadership.

Comment by:

Reviewer 3

9.MethodologyAs the study intends to find out the determining factors of instructional leadership, it is the study of a cause-and-effect relationship. It is because those factors can be found out by studying the causes that influence instructional leadership. Therefore, it follows the post-positivistic deterministic philosophy of causes determine effects. The objective reality of instructional leadership exists out there, and that reality can be found out through careful study and measurement. The research design is quantitative, and research methods include the preparation of questionnaires, data collection, data analysis, and interpretation. A well-structured questionnaire was prepared using the new PIMRS. The new PIMRS with 11 factors and 33 items asked the respondents to rate their view on a 5-point Likert scale with the descriptors; 1= almost never, 2= seldom, 3= sometimes, 4= frequently, and 5= almost always at which the head teacher was engaged in those particular activities of the school. The data was collected using a survey design, an appropriate way to get quantitative or numerical descriptions of population trends, attitudes, or opinions by surveying a sample of that population [46].

The population of this study includes all public secondary schools in Kathmandu Valley. Kathmandu Valley was chosen for the study because it has the greatest concentration of public secondary schools compared to any other region in the nation. In total, 303 public secondary schools in the Kathmandu Valley made up the population of this study and 172 schools made up the sample size. 172 schools were chosen at random from a pool of 303 to visit to gather data. Data was collected from one teacher and the head teacher each from 172 schools to see the difference in head teachers' and teachers' ratings. One teacher from each school was chosen by the lottery process.

Regarding the methodology, it's necessary to clarify how the questionnaires were designed and administered to the research participants, and how the data analysis and interpretation were performed.As the study intends to find out the determining factors of instructional leadership, it is the study of a cause-and-effect relationship. It is because those factors can be found out by studying the causes that influence instructional leadership. Therefore, it follows the post-positivistic deterministic philosophy of causes determine effects. The objective reality of instructional leadership exists out there, and that reality can be found out through careful study and

<table>
<tr><td></td><td>measurement. The research design is quantitative, and research methods include the preparation of questionnaires, data collection, data analysis, and interpretation. Classical Delphi was conducted on Hallinger and Murphy's (1985) instructional leadership model, known as Principal/Head teacher Instructional Management Rating Scale (PIMRS) to develop a PIMRS suitable in the context of Nepal. A well-structured questionnaire was prepared using the new PIMRS. The questionnaire developed from new PIMRS with 11 factors and 33 items asked the respondents to rate their view on a 5-point Likert scale with the descriptors; 1= almost never, 2= seldom, 3= sometimes, 4= frequently, and 5= almost always at which the head teacher was engaged in those particular activities of the school. The data was collected using a survey design, an appropriate way to get quantitative or numerical descriptions of population trends, attitudes, or opinions by surveying a sample of that population [46]. Questionaire was administered to respondents during visits to th...</td></tr>
<tr><td>Additional Information:</td><td></td></tr>
<tr><td>Question</td><td>Response</td></tr>
<tr><td>Financial Disclosure

Enter a financial disclosure statement that describes the sources of funding for the work included in this submission. Review the submission guidelines for detailed requirements. View published research articles from PLOS ONE for specific examples.

This statement is required for submission and will appear in the published article if the submission is accepted. Please make sure it is accurate.

Funded studies
Enter a statement with the following details:
• Initials of the authors who received each award
• Grant numbers awarded to each author
• The full name of each funder
• URL of each funder website
• Did the sponsors or funders play any role in the study design, data collection and analysis, decision to publish, or preparation of the manuscript?

Did you receive funding for this work?</td><td>The author(s) received no specific funding for this work.</td></tr>
<tr><td>Competing Interests

Use the instructions below to enter a competing interest statement for this submission. On behalf of all authors, disclose any competing interests that could be perceived to bias this</td><td>The authors have declared that no competing interests exist.</td></tr>
</table>

work—acknowledging all financial support and any other relevant financial or non-financial competing interests.<br><br>This statement is required for submission and **will appear in the published article** if the submission is accepted. Please make sure it is accurate and that any funding sources listed in your Funding Information later in the submission form are also declared in your Financial Disclosure statement.<br><br>View published research articles from *PLOS ONE* for specific examples.<br><br>**NO authors have competing interests**<br><br>Enter: *The authors have declared that no competing interests exist.*<br><br>**Authors with competing interests**<br><br>Enter competing interest details beginning with this statement:<br><br>*I have read the journal's policy and the authors of this manuscript have the following competing interests: [insert competing interests here]*<br><br>* typeset	
**Ethics Statement**<br><br>Enter an ethics statement for this submission. This statement is required if the study involved:<br><br>• Human participants<br>• Human specimens or tissue<br>• Vertebrate animals or cephalopods<br>• Vertebrate embryos or tissues<br>• Field research<br><br>Write "N/A" if the submission does not require an ethics statement.	Ethical standards were maintained throughout the research process, including during data collection. Prior to initiating the study, ethical approval was obtained from the Research Committee of Kathmandu University, School of Education (Approval No. 181028-1). Recruitment period for pilot study was 24- 07- 2019 to 24- 08- 2019. Similarly, the recruitment period began in 25- 08- 2019 and concluded on 05- 02- 2020 for main study. Informed consent was secured from all participants. Head teachers, who served as respondents, were initially contacted by phone to request their time and contribution, while consent from the teacher respondents was obtained during school visits. A questionnaire with cover letter was distributed to each respondent. Some respondents provided data on the same day, while others scheduled a later time. Thus, the data was gathered in accordance with established ethical standards.

Powered by Editorial Manager® and ProduXion Manager® from Aries Systems Corporation

General guidance is provided below. Consult the submission guidelines for detailed instructions. **Make sure that all information entered here is included in the Methods section of the manuscript.**<br><br>**Format for specific study types**<br><br>**Human Subject Research (involving human participants and/or tissue)**<br>• Give the name of the institutional review board or ethics committee that approved the study<br>• Include the approval number and/or a statement indicating approval of this research<br>• Indicate the form of consent obtained (written/oral) or the reason that consent was not obtained (e.g. the data were analyzed anonymously)<br><br>**Animal Research (involving vertebrate animals, embryos or tissues)**<br>• Provide the name of the Institutional Animal Care and Use Committee (IACUC) or other relevant ethics board that reviewed the study protocol, and indicate whether they approved this research or granted a formal waiver of ethical approval<br>• Include an approval number if one was obtained<br>• If the study involved *non-human primates*, add *additional details* about animal welfare and steps taken to ameliorate suffering<br>• If anesthesia, euthanasia, or any kind of animal sacrifice is part of the study, include briefly which substances and/or methods were applied<br><br>**Field Research**<br><br>Include the following details if this study involves the collection of plant, animal, or other materials from a natural setting:<br>• Field permit number<br>• Name of the institution or relevant body that granted permission	
**Data Availability**<br><br>Authors are required to make all data underlying the findings described fully	Yes - all data are fully available without restriction

available, without restriction, and from the time of publication. PLOS allows rare exceptions to address legal and ethical concerns. See the PLOS Data Policy and FAQ for detailed information.<br><br>A Data Availability Statement describing where the data can be found is required at submission. Your answers to this question constitute the Data Availability Statement and **will be published in the article**, if accepted.<br><br>**Important:** Stating 'data available on request from the author' is not sufficient. If your data are only available upon request, select 'No' for the first question and explain your exceptional situation in the text box.<br><br>Do the authors confirm that all data underlying the findings described in their manuscript are fully available without restriction?	
**Describe where the data may be found in full sentences. If you are copying our sample text, replace any instances of XXX with the appropriate details.**<br><br>• If the data are **held or will be held in a public repository**, include URLs, accession numbers or DOIs. If this information will only be available after acceptance, indicate this by ticking the box below. For example: *All XXX files are available from the XXX database (accession number(s) XXX, XXX.*).<br>• If the data are all contained **within the manuscript and/or Supporting Information files**, enter the following: *All relevant data are within the manuscript and its Supporting Information files.*<br>• If neither of these applies but you are able to provide **details of access elsewhere**, with or without limitations, please do so. For example:<br><br>*Data cannot be shared publicly because of [XXX]. Data are available from the XXX Institutional Data Access / Ethics*	Data underlying the result presented in the study have been uploaded with this article as supplementary information.

Committee (contact via XXX) for researchers who meet the criteria for access to confidential data.<br><br>*The data underlying the results presented in the study are available from (include the name of the third party and contact information or URL).*<br>• This text is appropriate if the data are owned by a third party and authors do not have permission to share the data.<br><br>* typeset	
Additional data availability information:	Tick here if the URLs/accession numbers/DOIs will be available only after acceptance of the manuscript for publication so that we can ensure their inclusion before publication.; Tick here if your circumstances are not covered by the questions above and you need the journal's help to make your data available.

Manuscript Click here to access/download;Manuscript;CleanCopy_IL.docx

Determinants of instructional leadership: A survey of public secondary schools

Suman Pande[1*], Laxman Acharya[2]¶, Prakash C Bhattarai[3]¶

[1]Department of Development Education, Kathmandu University, School of Education, Hattiban, Lalitpur, Nepal

[2]Department of Development Education, Kathmandu University, School of Education, Hattiban, Lalitpur, Nepal

[3]Department of Development Education, Kathmandu University, School of Education, Hattiban, Lalitpur, Nepal

*Corresponding author

E-mail: suman_devpdh2023@kusoed.edu.np (SP)

¶ Conceptualization: Prakash C. Bhattarai
Formal analysis: Suman Pande
Methodology: Suman Pande, Prakash C. Bhattarai, Laxman Acharya
Supervision: Laxman Acharya
Writing – original draft: Suman Pande
Writing – review & editing: Suman Pande, Prakash C. Bhattarai, Laxman Acharya

Acknowledgements: The authors express their gratitude to all those head teachers and teachers of public secondary schools of Kathmandu valley who provided data for this study. The authors would also like to acknowledge Kathmandu University faculty members who directly or indirectly helped to do this research.

Abstract

Instructional leadership is centered on teaching and learning that academic leaders undertake to promote higher levels of student learning. Given its importance, this study examines the factors influencing instructional leadership in Kathmandu, Nepal. The framework for the study is grounded in Hallinger and Murphy's (1985) model of instructional leadership, which was employed as the conceptual basis. Using the Classical Delphi method, the model was adapted to develop a measurement scale, while the study was situated within a post-positivist paradigm, assuming that the reality of instructional leadership exists independently. Exploratory factor analysis was then conducted to extract the underlying factors. The findings reveal three major factors influencing instructional leadership: (a) planning, managing, and supervising instructional programs; (b) motivating and developing the competence of teachers and students; and (c) involving parents in the learning process. The inclusion of parental involvement emerged as a novel dimension in the context of Nepal. These results provide meaningful insights for school headteachers, other educational stakeholders, policymakers in the government sector, and international researchers interested in instructional leadership.

Keywords

Instructional leadership, School, Head teacher, Student, Academic achievement, Factors

Introduction

The function of instructional leadership is vital in schools as it vastly influences the academic achievement of students [1, 2, 3]. In the schools of Nepal, the headteacher is only the academic leader who plays the role of instructional leader. In this regard, the head teachers are considered to be accountable for students' academic achievement. In Nepal, the policies for strengthening the role of the head teacher as an effective leader were enacted in the Education Rule 2002 [4]. However, some instructional leadership roles were also defined, but they were grouped with administrative roles and not specifically recognized as instructional leadership responsibilities. The other policies formulated for the development of school education are Three Year Interim Plan (2007/08 -2009/10) [5] and other sequential Three-Year Interim Plans, School Sector Reform Plan (SSRP) (2009/10-2015/16) [6] School Sector Development Plan (SSDP) (2016/17- 2022/23) [7], National Education Policy, 2076 B.S. [8], School Education Sector Plan

(SESP) (2022/23- 2031/32) [9]. In these policies, the policymakers have considered the leadership of the head teacher as a way to enhance students' academic progress. However, the instructional leadership role as the duty of the head teacher has been mentioned clearly only in SSDP (2016/17- 2022/23) [7]. This demonstrates that the Ministry of Education within the Nepal Government has consistently worked to enhance Nepal's educational system. Still, the instructional leadership concept was introduced in the policy document only in 2016.

In spite of several efforts from the Nepal government to improve school education, satisfactory results have not been achieved by the public schools of Nepal for a long time. A review of the SLC exam results of Nepal from 1994 to 2013 showed that the average pass percentage of the SLC exam was only 45.50% over those 20 years [10]. After replacing the SLC with SEE and reforming the existing assessment system by changing the numerical grading system to a letter system through the Education Act 2016, the result has become even worse. The SEE results of recent years have shown a continuous downfall in public schools [11]. The result of SEE, which is evaluated in the letter grading system, is not satisfactory in Nepal [12]. The study of SEE result data showed that only 45 % of students were above the satisfactory level in the academic year 2018 and 2019; however, it reached 78% in the academic year 2020 due to the use of student scores from schools rather than center-based exams in the framework of Covid-19 [13]. In 2021, the SEE pass percentage increased as the board issued grade sheets based on internal evaluations conducted by individual schools in response to the pandemic [14]. In the subsequent years, the results reverted to a similar trend, with only 47.86% of students passing the SEE exam in 2024 [15].

Unsatisfactory school performance over the years is evident from the SLC (School Leaving Certificate) results before the Education Act 2016, and SEE (Secondary Education Examination) results after the Education Act 2016 [16, 12, 17, 11, 18, 19, 20, 21,22]. The unsatisfactory performance of public schools in Nepal is a blatant indication of the head teachers' inadequate effort in the teaching and learning process in these schools, a responsibility that falls under their instructional leadership role. In this regard, there is an urgent need to identify the influencing factors of head teachers' instructional leadership. Although there is some literature on instructional leadership, no study has been found that examines the factors influencing the instructional leadership of headteachers in the local context of Nepal. Therefore, it was deemed imperative to identify the factors influencing headteachers' instructional leadership. Accordingly,

the research question guiding this study is: What are the factors that affect headteachers' instructional leadership? Identifying these factors helps headteachers recognize areas where they may be falling short and where improvement is needed. This research is expected to support headteachers in developing their professional capacities as effective instructional leaders, which, in turn, can enhance students' achievement in schools. Furthermore, the findings of this study can contribute to the existing body of knowledge on instructional leadership.

Reviewing the Literature

Researchers began examining school effectiveness and program improvement in the late 1970s. They first identified effective schools and then analyzed what made them successful, noting that a skillful head teacher was a key factor in driving school improvement [23]. It was emphasized that instructional leadership plays a pivotal role in fostering school improvement, promoting better academic outcomes both directly, by supporting teachers, and indirectly, by improving conditions for teaching and learning. Successful school leadership also involves enriching the curriculum, redefining the roles and responsibilities, enhancing teacher quality, and building strong relationships within the school and with the wider community [24].

Instructional leadership is a construct derived from the combination of the concepts of "instruction" and "leadership." There have been multiple efforts to define instruction, but four key dimensions of instruction remain consistent: setting learning objectives, choosing teaching methods, selecting reinforcement activities, and evaluating outcomes [25]. Researchers hold varying views on leadership as well. Some have defined leadership as how a leader leads; others have described it as the job done through people [25]. Leadership includes followers acting toward goals that represent the values of the leaders and the followers [26]. Such uncertainty surrounding the concept of leadership has made the combined construct of "instructional leadership" difficult to define and clarify. The early instructional leadership models tended to focus on the leadership portion of the construct, and they attempted to define it in terms of general leadership theory. Those models focused on personality/leadership style, behavioral/situational, or contextual components of the head teacher [24].

In this manner, the combined construct of "instructional leadership" has been defined in various ways. Instructional leadership means providing the necessary resources, possessing knowledge and skill in curriculum and instructional matters, being a skilled communicator, and being a visionary who is out and around creating a visible presence [26]. Instructional leadership

is direct assistance to teachers in their day-to-day activities, the development of collaborative groups among staff, the design and procurement of effective staff development, curriculum development, and the use of action research [27]. These definitions emphasized not only improving the instructional leadership role of the head teacher through the monitoring of instructional activities, but also focused on the managerial tasks of the head teacher within it. Later scholars of instructional leadership began linking it to activities aimed at enhancing teaching and learning, leading to a slight shift in how instructional leadership was defined. Here are some examples: instructional leadership begins when principals talk about being in the classroom, visit with students about their academic pursuits, and prioritize instruction [28]. Instructional leadership is important for effective practices in curriculum, instruction, and assessment, and for the ability to work with teachers on the day-to-day problems related to these topics [29]. The latter two perspectives on instructional leadership place a strong emphasis on teaching and learning activities. In this way, the concept of instructional leadership evolved through various research developments, ultimately establishing that leadership roles connected to teaching and learning within the school fall under the domain of instructional leadership [30, 31, 2, 32].

The instructional leadership notion has dominated the educational literature since the creation of instructional leadership models, and there is ample proof of its effectiveness regarding teacher and student results [33, 34]. The notable models of instructional leadership have been proposed by various researchers [e.g.,35, 36, 37, 38, 39, 40, 41]. They have offered a variety of indications of instructional leadership, but the theoretical underpinnings are often the same [40, 41]. Researchers most extensively use the Hallinger and Murphy (1985) model of instructional leadership to research instructional leadership. The widespread acceptance of this model is primarily due to Hallinger and Murphy's (1985) core concept, which provides head teachers with valuable insights into all aspects of leadership related to teaching and learning in schools. The main theme of this model is to develop and communicate school goals, coordinate and supervise the school curriculum, monitor and evaluate student progress, encourage teachers and students, and maintain high visibility and a hands-on approach [23, 44]. Their model is in the form of a scale known as PIMRS (Principal/Head Teacher Instructional Management Rating Scale). This PIMRS has been utilized in at least 130 PhD dissertations over almost three decades [45].

Several academics have mentioned their views on the characteristics of an instructional leader. Instructional leaders perform at high levels in four areas: a) resource provider, b) instructional resource, c) communicator, and d) visible presence in the school [35]. The instructional leaders emphasize: a) goal establishment and curriculum development, b) frequent observations, c) program evaluation, and d) support for teachers [41]. Instructional leaders perform seven crucial functions, which are: a) establishing and implementing instructional goals; b) being available to staff; c) creating a school culture and climate conducive to learning; d) communicating the vision and mission of the school to students, staff, and parents; e) setting high expectations for the staff; f) developing teacher leaders; g) maintaining positive attitudes toward student, staff, and parents [39]. There are four tenets of an instructional leader: a) focus on developing, directing, and supervising the curriculum and instruction in the classroom; b) interacting with teachers to improve teaching and learning c) being goal-oriented; d) establishing high expectations and standards for teachers and students [38]. The effective instructional leader plays five important roles, which are: a) establishing goals and expectations; b) strategic resourcing; c) planning, coordinating, and evaluating teaching and the curriculum; d) promoting and participating in teacher learning and development; and e) ensuring an orderly and supportive environment [40]. There are six characteristics of effective instructional leaders: a) identifying and communicating the mission and vision of the school; b) maintaining a school environment that is conducive to learning; c) promoting the professional development of staff; d) monitoring teachers' instruction; e) maintaining a positive school climate; and f) initiating organizational change [36]. From these scholars' views, it can be inferred that an instructional leader's job is to improve instruction to increase student achievement. Hence, an instructional leader is a person who seeks to ensure effective teaching and learning in the school by meeting all the requirements necessary to enhance students' academic performance.

Among the models of instructional leadership proposed by various researchers devoted to the study of instructional leadership, Hallinger and Murphy's (1985) model of instructional leadership was used as a theoretical guide for this research because this model has attained top priority and is more dominant in the literature on instructional leadership. This model of instructional leadership consists of three factors: defining the school's mission, managing the instructional program, and promoting a positive learning climate. There are two functions under the factor defining the school's mission, which are framing the school goals and communicating

the school goals; three functions under the factor managing the instructional program, which are supervising and evaluating instruction, coordinating curriculum, and monitoring students' progress; and five functions under the factor promoting a positive learning climate which are protecting instructional time, providing incentives for teachers, providing incentives for learning, promoting professional development of teachers, and maintaining high visibility. There are five items under each of these ten functions, forming fifty variables altogether.

These fifty items were studied thoroughly, and a classical Delphi was conducted, followed by three rounds of meetings with experienced head teachers and teachers to get their concrete ideas on them. Based on their opinions, some items- for example, (i) draw upon the results of class-wise testing when making curriculum decisions, (ii) participate actively in the review of curricular materials, (iii) acknowledge teachers' exceptional performance by writing memos for their personal files, (iv) recognize superior students' achievement or improvement by seeing in the office the students with their work, (v) review student work products when evaluating classroom instruction- were deselected from this PIMRS. Some items- for example, (i) head teacher announces mandatory presence of parents during terminal report card distribution, (ii) head teacher meets the parents of poor-performing students and shares with them what roles the school and parents may together take for their improvements, (iii) head teacher learns students' family problems and talks to their parents to resolve them, (iv) the head teacher is transparent about every financial detail of the school to all stakeholders- were added to the in new PIMRS.

Some items were changed to best suit Nepali schools, such as (i) monitoring the classroom curriculum to see that it covers the school's curricular objectives was changed to the head teacher checking teachers' log books regularly to see if they are going in accordance with the syllabus, (ii) limiting interruption of instructional time by public address announcements was changed to head teacher trying his best to ensure uninterrupted instructional time, (iii) leading or attending teacher in- service activities concerned with instruction was changed to head teacher seeking to provide training to teachers during vacations so as not to interrupt daily classes, (v) conducting informal observations in classrooms regularly was changed to head teacher regularly monitoring the activities of teachers and students while they are in class. In this way, a new PIMRS was finally developed for this research. This PIMRS consists of 11 factors and 33 items.

Research Methods

As the study intends to find out the determining factors of instructional leadership, it is the study of a *cause*-and-effect relationship. It is because those factors can be found out by studying the causes that influence instructional leadership. Therefore, it follows the post-positivistic deterministic philosophy of *causes* determine *effects*. The objective reality of instructional leadership exists out there, and that reality can be found through careful study and measurement. Although the reality of instructional leadership exists independently, it can only be understood through data and evidence. Therefore, this study relies on statistical analysis of numerical data, a method known as quantitative research. The research design is quantitative, and research methods include the preparation of questionnaires, data collection, data analysis, and interpretation. Classical Delphi was conducted on Hallinger and Murphy's (1985) instructional leadership model, known as Principal/Head teacher Instructional Management Rating Scale (PIMRS) to develop a PIMRS suitable in the context of Nepal. A well-structured questionnaire was prepared using the new PIMRS. The questionnaire developed from the new PIMRS with 11 factors and 33 items asked the respondents to rate their view on a 5-point Likert scale with the descriptors: 1= almost never, 2= seldom, 3= sometimes, 4= frequently, and 5= almost always, on which the head teacher was engaged in those particular activities of the school. The data was collected using a survey design, an appropriate way to get quantitative or numerical descriptions of population trends, attitudes, or opinions by surveying a sample of that population [46]. A questionnaire was administered to respondents during visits to their schools.

The population of this study includes all public secondary schools in Kathmandu Valley. Kathmandu Valley was chosen for the study because it has the greatest concentration of public secondary schools compared to any other region in the nation. In total, 303 public secondary schools in the Kathmandu Valley made up the population of this study, and 172 schools made up the sample size. 172 schools were chosen at random from a pool of 303 to visit to gather data. Data (see S1_dataset in the Appendix) was collected from one teacher and the head teacher, each from 172 schools, to see the difference in head teachers' and teachers' ratings. One teacher from each school was chosen by the lottery process. Factor analysis was conducted on the obtained data to identify the factors affecting head teachers' instructional leadership (see

S2_output@FactorAnalysis in the Appendix). The findings were analyzed and interpreted in the light of the relevant literature.

Ethical standards were maintained throughout the research process, including during data collection. Before initiating the study, ethical approval was obtained from the Research Committee of Kathmandu University, School of Education (Approval No. 181028-1). The recruitment period for the pilot study was 24-07-2019 to 24-08-2019. Similarly, the recruitment period began on 25-08-2019 and concluded on 05-02-2020 for the main study. Informed consent was secured from all participants. Head teachers, who served as respondents, were initially contacted by phone to request their time and contribution, while consent from the teacher respondents was obtained during school visits. A questionnaire with a cover letter was distributed to each respondent. Some respondents provided data on the same day, while others scheduled a later time. Thus, the data was gathered in accordance with established ethical standards.

The pilot testing of the questionnaire was done with the help of 30 head teachers and teachers from the schools, apart from the randomly selected 172 schools. A default sample size of 30 is advised for questionnaire pilot testing [47]. The majority of the items' mean scores on the questionnaire's pilot test were determined to be more than four times the standard deviation. A low standard deviation indicates that the majority of the numbers are extremely close to the average, demonstrating the normal distribution. This ensured the reliability of the questionnaire [48]. The data were collected from 172 public schools of Kathmandu Valley with the help of this questionnaire, and the data were subjected to factor analysis to determine the factors influencing instructional leadership.

All the conditions required for factor analysis were checked. Factor analysis on Likert scale data is valued [49]. The criteria were met as a 5-point Likert scale was used in this study. A lower number of categories cannot provide more possibilities and cannot help to obtain more reliable responses; on the other hand, a large range of scale impacts respondents' responses and reduces their chances of providing the proper answer on a specific item [50]. Therefore, I created a questionnaire using a 5-point Likert scale. The ratio of sample size to variable should be three to six [51]. Since the sample size is 172 and the number of variables is 39, this is within the recommended range of 3:1 to 6:1. One of the most common situations is the retention of factors with Eigenvalues greater than one [52, 53]. In this study, all factors had Eigenvalues greater than

1; hence, this criterion was also met. This study also met the requirement of item loading .40 to .50 [54] as the item loading was .50.

Another condition is that the average commonalities or extraction value of items is more than 0.5 [54]. The average extraction value for items was .593. Therefore, this requirement was also fulfilled. Another criterion is Kaiser-Meyer sampling adequacy. If the KMO measure is greater than .50, it is safe to assume that the factor ability exists in the dataset [52, 55]. The Kaiser-Meyer sampling adequacy score was .896. So, this condition was also deemed met. A further requirement is that a factor must have at least three components to be considered genuine [56, 54]. This criterion was also fulfilled because there were three to fourteen items in each of the three instructional leadership factors. All the conditions for factor analysis were met, and three factors of instructional leadership were obtained. The Cronbach's alpha value of instructional leadership factors ranged between .75 to .92. The literature suggests that an alpha value of .75 to .95 is adequate [57]. Therefore, that was acceptable. Thus, the reliability of factor analysis was assessed.

Result

The factor analysis yielded three instructional leadership factors. Fourteen items loaded on the first factor, eight on the second, and three on the third.

Factor One: Planning, managing, and supervising instructional program

All the items of factor one are shown below in the rotated component matrix of this factor.

Table 1
Rotated Component Matrix of Factor One of IL

SN	Items	Factors		
		1	2	3
1.	The head teacher analyses students' performance from the last year while planning for the new academic session.	.610		
2.	The head teacher specifies the school's whole year educational plan in the presence of all concerned people.	.652		
3.	The head teacher seeks to incorporate teachers' inputs in planning during his formal or informal meetings.	.588		
4.	The head teacher shares academic plans with everyone involved and discusses how those plans may be effectively implemented.	.651		
5.	The head teacher displays the school's whole year plan on the notice boards and communicates it to students during assembly.	.639		
6.	The head teacher reports educational planning of the school to the local			

government and the Province/Local level education offices.	.604
7. The head teacher regularly monitors the activities of teachers and students while they are in class.	.634
8. The head teacher makes sure that students' every class work/homework is completed timely and all notes are marked by assigned teachers at least a month before terminal examinations.	.695
9. The head teacher seeks to find out the teachers' areas of improvement in instructional practices.	.711
10. The head teacher gets every subject department head to implement the syllabus of all subjects in each class.	.653
11. The head teacher checks teachers' log books regularly to see if they are going in accordance with the syllabus.	.630
12. The Head teacher reviews whether the curriculum goal is achieved through students' terminal examination results, their discipline, and overall change indicators	.664
13. The head teacher regularly discusses with subject teachers regarding each student's progress	.530
14. The head teacher instructs teachers to ensure no student has bunked classes	.528

Explanation of Variables in Factor One

These fourteen variables are connected to five distinct instructional leadership roles: planning and controlling school educational activities, overseeing and evaluating instruction, coordinating the curriculum and other educational objectives, keeping track of students' progress, and utilizing instructional time. These specific instructional leadership functions of the head teacher, except the head teacher directs teachers to make sure no student has skipped class, are similar to the items in Hallinger and Murphy's (1985) framework of IL falling under their two factors, defining the school mission, and managing instructional program [58, 59, 60, 61]. Therefore, by including the meaning of those three leadership functions, namely defining the school mission, managing the instructional program, and ensuring no student has bunked the class, this factor was appropriately named as planning, managing, and supervising the instructional program. All the variables of the factor planning, managing, and supervising instructional program show that it is about making an educational plan for the school, coordinating the curriculum, sharing plans, observing instructors to ensure they are practicing good teaching techniques, communicating with teachers to learn about their students' development, and making attempts to improve. There were two factors: framing and communicating school goals, and supervising and evaluating instruction for these particular instructional leadership activities in Hallinger and Murphy's (1985) model [37]. They

mentioned, it is about setting and expressing goals, working with staff members and teachers, and managing the instructional process and curriculum. According to [62] this involves establishing the school's goals, reminding teachers of its purposes and mission, and informing teachers that teachers need to plan their classes properly. A school lacks any standards to assess its success without a plan to improve the educational process. As stated by [63] this factor relates to selecting the schools' primary goals as well as coordinating and managing curriculum and instruction. Similarly, [64] notes that planning, managing, and supervising instructional programs entails articulating and expressing the school's goals, coordinating the curriculum, and monitoring and controlling the program by supervising and evaluating instruction.

This factor also resonates with the conceptualization of instructional leadership by [65], which is grounded in head teachers' core practices and was developed to explain how Norwegian school leaders build their instructional capacity. Their multidimensional model, consisting of goal orientation, collaboration with teachers, professional development, observation, and supervision, is similar to the variables of the factor planning, managing, and supervising instructional program identified in this study. Both underscore head teachers' active role in shaping and monitoring instructional processes as a foundation for improving teaching and learning.

This finding can also be compared with those [66], where coordinating the curriculum and providing professional learning opportunities to teachers emerged as statistically significant predictors of teachers' professional development in the context of Nigeria. Coordinating the curriculum is parallel to the variable "the head teacher gets every subject department heads to implement the syllabus of all subjects in each class" within the factor of planning, managing, and supervising instructional program. This reinforces the importance of this variable for developing teaching and learning ability. The next predictor, providing professional learning opportunities to teachers, corroborates the variables of the second factor, motivating and developing the competence of teachers and students developed in this study.

Factor Two: Motivating and developing the competence of teachers and students

All the items of factor two are shown below in the rotated component matrix of this factor.

Table 2

Rotated Component Matrix of Factor Two of IL

SN	Items	Factors		
		1	2	[illegible]
1.	The head teacher publicly praises teachers' superior performance, but meets them in private for correction.		.608	
2.	The head teacher tries to manage opportunities for sincere teachers to work in a higher position as a reward for their good work.		.668	
3.	The head teacher provides a letter of appreciation or honor certificates to teachers for their contribution.		.725	
4.	The head teacher seeks to provide training to teachers during vacations or so as not to interrupt daily classes.		.735	
5.	The head teacher actively supports teachers to use those learnt skills in the classrooms.		.695	
6.	The head teacher sometimes provides opportunities for observation or an educational tour for teachers.		.719	
7.	The head teacher publicly honors students for their excellent performance or discipline.		.489	
8.	The head teacher learns about students' family problems and talks to their parents to resolve them.		.549	

Explanation of Variables in Factor Two

Factor two consists of eight variables, which explain the role of an instructional leader in motivating and supporting teachers and students for their academic growth. These eight variables serve similar functions to the variables in Hallinger and Murphy's (1985) Framework of instructional leadership, placed under the functions providing incentives for teachers, providing incentives for learning, and promoting professional development under the factor creating a positive school climate [58, 59, 60, 61]. Since the eight variables obtained from this factor share the same aim of inspiring teachers and students for their development, a suitable name, 'motivating and developing competence of teachers and students', was given to this factor.

All the variables of the factor motivating and developing the competence of teachers and students show that it is about encouraging teachers and students to develop their abilities and academic excellence. Praising and rewarding teachers and students for their good deeds, assisting teachers with their professional growth, and supporting students in improving their self-confidence and academic focus are the key roles of an instructional leader, which are crucial in achieving academic success in the school. As stated by [62], recognizing and rewarding teachers' and students' accomplishments is important for fostering their interest and engagement.

These instructional leadership roles align with two functions in Hallinger and Murphy's (1985) instructional leadership model, promoting professional development and providing incentives for learning. According to [64], this factor emphasizes inspiring and enhancing the competence of both teachers and students. The overall focus is on helping both teachers and students get better.

The variables within this factor, such as offering training to teachers during vacations, supporting the classroom application of acquired skills, and facilitating educational tours, corroborate the finding of [66], who found that providing professional learning opportunities to teachers supports their professional development. In contrast to the finding of [66], who did not find providing incentives for teachers to be a significant predictor of teachers' professional development, the present study suggests that motivational strategies such as public praise, appreciation letters, honor certificates, and rewarding sincere teachers with opportunities for career development are integral to strengthening teachers' competence. Additionally, the inclusion of student-focused practices such as honoring excellent performance and addressing family problems reflects a broader and more relational approach for both teachers' and learners' holistic development.

Factor Three: Involving Parents

All the items of factor three are shown below in the rotated component matrix of this factor.

Table 3

Rotated Component Matrix of Factor Three of IL

SN	Items	Factors		
		1	2	3
1.	The head teacher calls class class-wise parents’ meeting and discusses students’ progress.			.553
2.	The head teacher announces the mandatory presence of parents during the terminal report card distribution.			.839
3.	The head teacher meets the parents of poor performing students and shares with them what roles the school and parents may together take for their improvement.			.653

Explanation of Variables in Factor Three

The factor three of instructional leadership consists of three items. These items are linked to the head teachers' policy to get parents/guardians involved in knowing about their children's condition and taking the appropriate action if necessary. In accordance with the meaning of these variables, a suitable name involving parents was given to this factor. The core idea summarized in the variables of this factor is to enhance parent/guardian-school communication for students' academic development. The parents/guardians are also made accountable, along with the school, through this strategy. Parental role is found to be more important to students' academic progress in the Nepali context. Some studies have shown the importance of the parent-school relationship. For example, some studies [67, 68] have shown a positive relationship between parental involvement and students' academic achievement, [69] reported that the increasing parental and community involvement in the educational process results in stronger relationships between school leaders and the community. Similarly, [70] emphasized the need for developing relational behavior for school administrators, including interaction with parents. Recent research [71] conducted in Hong Kong, and Macao also demonstrated that leadership can influence student achievement through Parental Academic Commitment (PAC). This reinforces the idea that parent involvement is not just a peripheral activity but a leadership-mediated pathway to better student outcomes.

Discussion

The meaning of the variables under three different factors, the planning, managing, and supervising instructional program**;** motivating and developing competence of teachers and students; and involving parents, are closely aligned with some international findings on factors influencing leadership that contribute to improving teaching and learning in the schools. For example, these three factors identified in this study are parallel to the leadership pathways proposed by [72] in their study conducted in Chicago, USA. The planning, managing, and supervising instructional program aligns with the ‘learning climate’ pathway in which head teachers establish conditions necessary to maintain a strong instructional focus. Motivating and developing the competence of teachers and students reflects the ‘professional capacity’ pathway, emphasizing the development of teachers' skills, professional quality, and capacity to work together. Finally, involving parents directly corresponds to the ‘parent-community ties’ pathway, which strengthens collaboration between parents and school to enhance student outcomes.

Again, [72] concluded that leadership affects students' achievement primarily by improving the learning climate, which further supports the planning, managing, and supervising instructional program as a factor for enhancing students' academic outcomes through improved teaching and learning.

It also seems informative to compare the findings of the present study with the research on head teachers' competencies by [73] in Vietnam because instructional leadership is one of the various competencies of head teachers. Three of the eight competency domains identified in the Vietnam study, the ‘instructional program management’, ‘staff development’, and ‘engagement with families’, closely align with the three factors found in the present study. This suggests that the three dimensions developed in the present study are not only central to teaching and learning improvement in Nepal but are also recognized internationally as essential aspects of effective school leadership. A meta-analysis [74] covering 24 studies and 9,178 teachers revealed that the head teachers' instructional leadership is moderately related to teacher self-efficacy, which results in enhanced student achievement. This finding aligns with the result of the present study, where motivating and developing the competence of teachers and students emerged as one of the factors of instructional leadership. A systematic review of 349 Asian studies by [75] revealed that the PIMR (Principal/head teacher Instructional Management Rating Scale) dominates the literature focusing on managing instructional programs and promoting a positive learning climate. In this regard, the first two factors of the present study parallel those dimensions.

"Involving parents" is a new factor developed in this study. The role of head teachers in involving parents as one of the factors of head teachers' instructional leadership in the context of Nepal is a noteworthy finding. Although studies have shown that parental involvement is crucial for students' achievement, none of the established instructional leadership frameworks explicitly recognize it as a distinct factor of instructional leadership.

An Indonesian study showed a positive correlation between parental participation and academic achievement in school [76]. He found that students with active parental involvement had higher test scores and grade point averages. Many studies have shown that parental participation positively influences students' educational performance. A favorable and modest impact of parental involvement on students' academic attainment was found in a meta-analysis of 37 studies published between 2000 and 2013 in kindergarten, primary, and secondary schools [77]. The mediation test results of [78] revealed a partially mediating role of parental

involvement in the relationship between socioeconomic status and students' academic achievement, as well as a strong mediating effect between family environment and academic achievement in rural China. A meta-analytic study with a random effects approach by [79] across Scopus-indexed publications showed a significant positive correlation between parental involvement and student achievement.

While substantial research supports the positive impact of parental involvement on student achievement, some studies indicate that parental involvement is not always beneficial. A study conducted in Canada showed no significant results for the influence of parental participation on students' academic progress [80]. A study conducted in the United States of America involving 450 students from kindergarten to fourth grade showed that the students who scored higher academically had lower parental involvement in school [81]. Such findings imply that the country's and locality's cultural and societal institutions and practices may determine whether or not parental participation influences students' academic achievement.

Conclusion

In response to the research question, what factors affect head teachers' instructional leadership, the study identified three factors. The first factor, planning, managing, and supervising instructional programs, explains the head teacher's role in collaborating with teachers to ensure that the school has a clear mission and that the mission concentrates on students' academic achievement. The second factor, motivating and developing the competence of teachers and students, describes the head teacher's activities to boost teachers' and students' interest in their line of work. The third factor, involving parents, mentions the roles of parents/ guardians in association with the school for students' academic progress. The study's key finding is that the parents'/ guardians' involvement in students' progress is seen as an essential factor in the schools of Nepal, which is different from Hallinger and Murphy's (1985) model of instructional leadership. In Hallinger and Murphy's (1985) instructional leadership model, the parents'/guardians' involvement is not explicitly recognized as a factor in student success. Instead, the model includes only one related item, contacting parents to communicate improved or exemplary student performance or contributions, which falls under the factor ‘providing incentives for learning’. This suggests that Nepal's unique cultural and societal framework places greater emphasis on the role of parents/ guardians in students' academic achievement. By identifying these factors, this study contributes to the field of instructional leadership and

underscores specific requirements for effective teaching and learning in the public schools of Nepal.

Implications of the Study

The results of this study can help head teachers stay informed in their role as instructional leaders by providing recommendations for enhancing teaching and learning in schools**.** The finding can also assist policymakers and bureaucrats in formulating and implementing necessary policies to enhance head teachers' instructional leadership. Additionally, it can support local authorities in improving schools, as the responsibility for school development is entrusted to local bodies in Nepal's federal democratic system. In this way, the result of this study can be very useful for making schools effective and attaining quality education. Further, this study has added new knowledge to the construct of instructional leadership in the local Nepali context by identifying the factors that influence it, which may be of interest to international researchers of this field.

Acknowledgements The authors express their gratitude to all those head teachers and teachers of public secondary schools of Kathmandu valley who provided data for this study. The authors would also like to acknowledge Kathmandu University faculty members who directly or indirectly helped to do this research.

Funding

The authors have not received any financial support for the research, authorship, and/or publication of this article.

Competing interests

The authors declare that they have no conflict of interest.

Ethical approval

The authors declare that they follow ethical standards

References

1. Bhujel, C. B. (2021). The role of principal in improvement of school performance: A qualitative study in community school of Nepal. *Artech J. Art Social Sci., 3,* 11-22. https://doi.org/10.32861/rje.71.1.10

2. Hallinger, P., & Murphy, J. (2012). Running on empty? Finding the time and capacity to lead learning. *NASSP Bulletin*, *97*(1), 5-21. https://doi.org/10.1177/0192636512469288

3. Lamsal, B. (2022). Instructional leadership behavior among principals of two schools in Lalitpur, Nepal. *TVET Journal*, *1*(16), 50 59. https://doi.org/10.3126/tvet.v1i16.45186

4. Nepal Law Commission. (2002). *Education regulation 2002*. https://lawcommission.gov.np/content/12987/12987-education-regulation-2059/

5. National Planning Commission. (2007). *Three-year interim plan (2007/08-2009/10)*. Government of Nepal. https://www.yumpu.com/en/document/view/6747910/three-year-interim-plan-2007-08-2009-10

6. Ministry of Education. (2009). *School sector reform plan (2009/10-2015/16)*. Government of Nepal. https://www.globalpartnership.org/node/document/download?file=sites/default/files/SSRP.pdf

7. Ministry of Education. (2016). *School sector development plan. 2016/17-2022/23*. Government of Nepal. https://planipolis.iiep.unesco.org/sites/default/files/ressources/nepal_ssdp_final_document_oct_2016_0.pdf

8. CollegeNP. (2019, December 12). *National education policy of Nepal 2076 / 2019*. CollegeNP. https://www.collegenp.com/news/national-education-policy-2076

9. Ministry of Education, Science and Technology (2022). *School education sector plan, (2022/023-2031/032)*. Government of Nepal. https://planipolis.iiep.unesco.org/sites/default/files/ressources/nepal_school_education_sector_plan_2022-23-2031-32_.pdf

10. Bhattarai, Y. B, (2014). *The school leaving certificate (SLC) examination of Nepal: Exploring negative consequences*. [Master dissertation]. Carleton University. https://carleton.scholaris.ca/server/api/core/bitstreams/070a3183-e3ab-4a47-9b6e-19e6a2b28cf1/content

11. Setopati. (2019, July 8). *Seeing through SEE results*. Setopati. https://en.setopati.com/view/149213

12. Chapagain, Y. (2021). School student academic performance in Nepal: An analysis using the School Education Exam (SEE) results. *International Journal on Studies in Education*, *3*(1), 22-36. https://doi.org/10.46328/ijonse.34

13. Adhikari, P. (2020, August 8). Abuse of rights in evaluation. *Gorkhapatra Daily*. https://old.gorkhapatraonline.com/epaper/view?paper=gorkhapatra&today=2020-08-26.

14. Sherma, A.B. (2023, August 13). Did the SEE result genuinely improve this year? *Republica*.https://myrepublica.nagariknetwork.com/news/did-the-see-result-genuinely-improve-this-year

15. Dhakal, M. (2024, June 28).SEE results out: Over 52% students non- graded. *The Raisin Nepal*. https://risingnepaldaily.com/news/44994

16. Budhathoki, C. B., Khatri, H.B., Shrestha, D. Rana, B. K., Sigdel, T.P., Panta, K., & Thapa, S.W. (2014). *Status of SLC dropouts and identifying ways to engaging students in co-curricular activities: Final report*. Vertex Consult Pvt. Ltd. https://doe.gov.np/assets/uploads/files/0f853b187d239aa7a083672837d7636a.pdf

17. Danai, R.P. (2021). Head teachers' instructional leadership at community schools. *AMC Journal, 2*(1), 55-73. https://doi.org/10.3126/amcj.v2i1.35788

18. Koirala, A. (2015). Debate on public and private schools in Nepal. *International Journal of Social Sciences and Management, 2*(1), 3-8. https://doi.org/10.3126/ijssm.v2i1.11882

19. Pandey, A. (2015, June 18). Poor SLC results. *The Himalaya Times.* https://thehimalayantimes.com/opinion/poor-slc-results/

20. Parajuli, D. R. & Das, T. (2013). Performance of community schools in Nepal: A macro level analysis. *International Journal of Science and Technology Research, 2*(7), 148-154. https://www.ijstr.org/final-print/july2013/Performance-Of-Community-Schools-In-Nepal-A-Macro-Level-Analysis.pdf

21. Regmi, K.D. (2017). World Bank in Nepal's education: Three decades of neoliberal reform.

Globalization, Societies and Education, 15(2), 188-201.
https://doi.org/10.1080/14767724.2016.1169517

22. Thapa, A. (2015).Public and private school performance in Nepal: An analysis using the SLC examination. *Education Economics, 23*(1), 47-62.
https://doi.org/10.1080/09645292.2012.738809

23. Hallinger, P. (2003). Leading educational change: Reflections on the practice of instructional and transformational leadership. *Cambridge Journal of Education, 33*(3), 329- 351.
https://doi.org/10.1080/0305764032000122005

24. Day, C., Sammons, P., & Gorgen, K. (2020). Successful school leadership (ERIC Document No. ED614324). Education Development Trust. https://files.eric.ed.gov/fulltext/ED614324.pdf

25. Irwin, C. C. (1985). *What research tells the teacher about instructional leadership* (ED257173). ERIC. https://eric.ed.gov/?id=ED257173

26. Smith, F., & Andrews, R. L. (1989). *Instructional leadership: How principals make a difference* (ED314826). ERIC. Association for Supervision and Curriculum Development, Alexandria, VA. https://eric.ed.gov/?id=ED314826

27. Glickman, C. D., Gordon, S. P., & Ross-Gordon, J. M. (1995). *Supervision of instruction: A developmental approach* (3rd ed.). Allyn & Bacon.

28. Supovitz, J. A. (2000). Manages less, leads more (EJ616311). ERIC. *Principal Leadership, 1*(3), 14-19. https://eric.ed.gov/?id=EJ616311

29. Elmore, R. F. (2000). Building a new structure for school leadership (ERIC Doc. No. ED546618). Albert Shanker Institute. https://files.eric.ed.gov/fulltext/ED546618.pdf

30. Bush, T. (2007). Educational leadership and management: Theory, policy, and practice. *South African Journal of Education, 27*(3), 391-406. https://files.eric.ed.gov/fulltext/EJ1150205.pdf

31. Department of Education. (2008). *Managing teaching and learning: A module of the Advanced Certificate: Education (School Management and Leadership)*. Department of Education, South Africa. https://www.oerafrica.org/system/files/2024-01/managingteachingandlearning_0.pdf

32. Day, C., Gu, Q., & Sammons, P. (2016). The impact of leadership on student outcomes: How successful school leaders use transformational and instructional strategies to make a difference. *Educational Administration, 52*(2), 221– 258. https://journals.sagepub.com/doi/10.1177/0013161X15616863

33. Hallinger, P., & Heck, R. H. (1998). Exploring the principal's contribution to school effectiveness: 1980-1995. *School Effectiveness & School Improvement, 9*(2), 157-191. https://doi.org/10.1080/0924345980090203

34. Gaston, V. A. (2024). Instructional leadership of school heads and its impact on teachers' professional development. Instabright International Journal of Multidisciplinary Research, 5(1), 1-32. https://doi.org/10.52877/instabright.005.01.0202

35. Andrews, R., & Soder, R. (1987). Principal instructional leadership and school achievement (EJ353780). ERIC. *Educational Leadership*, *44*(6), 9-11. https://eric.ed.gov/?id=EJ353780

36. Gulcan, M. (2012). Research on instructional leadership competencies of school principals (EJ991116). ERIC. *Education, 132*(3), 625-635. https://eric.ed.gov/?id=EJ991116

37. Hallinger, P., & Murphy J. (1985). Assessing the instructional management behavior of principals. *Elementary School Journal*, 86(2), 217-248. https://doi.org/10.1086/461445

38. Leithwood, K. (1994). Leadership for school restructuring. *Educational Administration Quarterly, 30*(4), 498-518. https://doi.org/10.1177/0013161X94030004006

39. McEwan-Adkins, E. K. (2003). Seven steps to effective instructional leadership (2nd ed.). Corwin Press.

40. Robinson, V. M. J., Lloyd, C. A., & Rowe, K. J. (2008). The impact of leadership on student outcomes: An analysis of the differential effects of leadership types. *Educational Administration Quarterly, 44*(5), 635–674. https://doi.org/10.1177/0013161X08321509

41. Sheppard, B. (1996). Exploring the transformational nature of instructional leadership. *Alberta Journal of Educational Research, 42*(4), 325-344. https://www.researchgate.net/publication/234599554_Exploring_the_Transformational_Nature_of_Instructional_Leadership

42. Burch, P. (2007). The professionalization of instructional leadership in the United States: Competing values and current tensions. *Journal of Education Policy, 22*(2), 195-214. https://doi.org/10.1080/02680930601158943

43. Reitzug, U. C. (2008). Conceptualizing instructional leadership: The voices of principals (EJ806421). ERIC. *Education and Urban Society, 40*(6), 694-714.

44. Ovando, M., & Ramirez, A. (2007). Principals' instructional leadership within a teacher performance appraisal system: Enhancing students' academic success. *Journal of Personnel Evaluation in Education, 20* (1-2), 85-110. https://doi.org/10.1007/s11092-007-9048-1

45. Hallinger, P. (2011). A review of three decades of doctoral studies using the principal instructional management rating scale: A lens on methodological progress in educational leadership. *Educational Administration Quarterly, 47*(2), 271–306. https://doi.org/10.1177/0013161X10383412

46. Cresswell, J.W. (2012). *Educational research: Planning, conducting and evaluating quantitative and qualitative research* (4th ed.). Pearson.

47. Perneger T.V., Courvoisier, D.S., Hudelson, P.M., & Ageron, A. G. (2014). Sample size for pre-tests of questionnaire. *Quality of Life Research,* 24(1),147-51. https://doi.org/10.1007/s11136-014-0752-2

48. Frost, J. (2021). Standard deviation: Definition, examples, and analysis. Statistics By Jim. https://statisticsbyjim.com/basics/standard-deviation.

49. Zaman, N., Bibi, Z., Sheikh, S. R., & Raziq, A. (2020). Manualizing factor analysis of likert scale. *Journal of Management Sciences 7*(2), 56-67. https://www.researchgate.net/publication/345746851_Manualizing_Factor_Analysis_of_Likert_Scale_Data

50. Tarka, P. (2015). Likert scale and change in range of response categories. vs. the factors extraction in EFA model. *Acta Universitatis Lodziensis Folia Economica,311*(1), 27-37. https://www.researchgate.net/publication/285639462_Likert_Scale_and_Change_in_Range_of_Response_Categories_vs_the_Factors_Extraction_in_EFA_Model

51. Cattell, R. B. (1978). *The scientific use of factor analysis in behavioral and life sciences* (1st ed.). Plenum.

52. Field, A. (2009). *Discovering statistics using SPSS* (3rd ed.). Sage.

53. Maskey, R., Fei, J., & Nguyen, H.O. (2018). Use of exploratory factor analysis in maritime research. *The Asian Journal of Shipping and Logistics, 34*(2), 91-111. https://doi.org/10.1016/j.ajsl.2018.06.006

54. Tabachnick, B. G., & Fidell, L. S. (2007). *Using multivariate statistics* (5th ed.). Allyn & Bacon.

55. Zulkepli, M. B., Sipan, I., & Jibril, J. D. (2017). *An exploratory factor analysis and reliability analysis for green affordable housing criteria instrument*. Semantic Scholar. https://api.semanticscholar.org/CorpusID:201895136

56. Kline, P. (1994). An essay guide to factor analysis (1st ed.). Routledge

57. Tavacol, M., & Dennic, R. (2011). Making sense of cronbach's alpha. *International Journal of Medical Education 2,* 53-55. https://pmc.ncbi.nlm.nih.gov/articles/PMC4205511/

58. Alhosani, H.M.A. (2015). *Principals' instructional leadership practices and their relationship to teachers' instructional practices in Sarjah schools*. [Master's dissertation]. United Arab Emirates University. https://scholarworks.uaeu.ac.ae/all_theses/22/

59. Lyons, B. J. (2010). *Principal instructional leadership behavior as perceived by teachers and principals at New York State recognized and non-recognized schools*. Doctoral dissertation]. Seton Hall University. https://scholarship.shu.edu/dissertations/1401/

60. Peariso, J. F. (2011). *A study of principals' instructional leadership behaviours and beliefs of good pedagogical practice among effective California high schools serving socioeconomically*

disadvantaged and English learners. [Doctoral dissertation]. Liberty University. https://eric.ed.gov/?id=ED529189

61. Vilakaji, N.S. (2016). *Exploring principals' instructional leadership practices in primary schools: A case study of two underperforming township primary schools in Ekurhuleni South district, Gauteng province*. [Master dissertation]. University of Witwatersrand, Johannesburg. https://wiredspace.wits.ac.za/server/api/core/bitstreams/1b835bc1-3192-4ac8-898a-c474f5bcc38c/content

62. Krug, S.E. (1992). Instructional leadership: A constructivist perspective. *Educational Administration Quarterly, 28*(3), 430-443. https://doi.org/10.1177/0013161X92028003012

63. Hallainger, P. (2005). Instructional leadership and the school principal: A passing fancy that refuses to fade away. *Leadership and Policy in Schools, 4*(3), 1-20. http://doi.org/10.1080/15700760500244793

64. Ghavifekr, S., Radwan, O., & Velarde J. M. (2019). Teachers' perceptions of principal's instructional leadership roles and practices. *Journal Pendidikan Malaysia 44*(2), 72- 83. http://dx.doi.org/ 10.17576/JPEN-2019-44.02-08.

65. Jenssen, M. M. F., & Paulsen, J. M. (2022). Combining capacity for instructional leadership with individual core practices in the Norwegian policy context. *Educational Management Administration & Leadership, 52*(2), 475-492. https://doi.org/10.1177/17411432221084155

66. He, P., Guo, F., & Abazie, G. A. (2024). School principals' instructional leadership as a predictor of teacher's professional development. *Asian-Pacific Journal of Second and Foreign Language Education, 9*, Article 63. http://doi.org/10.1186/s40862-024-00290-0

67. Dahie, M. A., Mohamed, A.A., & Mohamed, R. A. (2018). The role of parental involvement in student academic achievement: Empirical study from secondary schools in Mogadishu-Somalia. *International Research Journal of Human Resources and Social Sciences, 5*(7), 1-24. https://www.researchgate.net/publication/326957190_THE_ROLE_OF_PARENTAL_INVOLVEMENT_IN_STUDENT_ACADEMIC_ACHIEVEMENT_EMPIRICAL_STUDY_FROM_SECONDARY_SCHOOLS_IN_MOGADISHU-SOMALIA

68. Sapungan, G. M., & Sapungam, R. M. (2014). Parental involvement in child’s education: Importnce, barriers and benefits. *Asian Journal of Management Sciences and Education 3*(2), 42-48. https://www.researchgate.net/publication/283539737_Parental_Involvement_in_Child's_Education_Importance_Barriers_and_Benefits

69. Stronge, J. H., Richard, H. B., & Catano, N. (2008). *Qualities of effective principals* (ED509122). ERIC. Association for Supervision and Curriculum Development. https://eric.ed.gov/?id=ED509122

70. Northouse, P. G. (2015). *Introduction to leadership: Concepts and practice* (3rd ed.). Sage.

71. Zhang, S., & Wu, H. (2025). School leadership, parental involvement, and student achievement: A comparative analysis of principal and teacher perspectives. *Education Sciences*, *15*(6), Article 767. https://doi.org/10.3390/educsci15060767

72. Sebastian, J., & Allensworth, E. (2012). The influence of principal leadership on classroom instruction and student learning: A study of mediated pathways to learning. Educational Administration Quarterly, 48(4), 626–663. https://doi.org/10.1177/0013161X11436273

73. Le, L. K., Tran, T., Le, H. T. T., & Le, T. T. T. (2020). A study on factors affecting school principals’ competencies in Vietnam’s mountainous provinces. *Management in Education, 35*(4), 174-181. https;//doi.org/10.1177/0892020620918254

74. Alanoglu, M. (2022). The role of instructional leadership in increasing teacher self-efficacy: A meta-analytic review. *Asia Pacific Education Review, 23*, 233-244. https;//doi.org/10.1007/s12564-021-09726-5

75. Hallinger, P., Liu, S., & Aung, P. N. (2025). A systematic state-of-the-art review of Asian research on principal instructional leadership, 1987-2024. *Education Sciences, 15*(7), Article 817. https;//doi.org/10.3390/educsci15070817

76. Utami, A.Y. (2022). The role of parental involvement in student academic outcomes. *Journal of Education Review Provision, 2* (1), 17-21. https;//doi.org/10.55885/jerp.v2i1.156

77. Castro, M., Exposito-Casas, E., Lopez-Martin, E., Lizasoain, L., Navarro-Asencio, E., & Gaviria, J.L. (2015). Parental involvement on student academic achievement: A meta-analysis. *Educational Research Review*, 14, 33-46. https://doi.org/10.1016/j.edurev.2015.01.002

78. Gu, X., Hassan, N. C., & Sulaiman, T. (2024). The relationship between family factors and academic achievement of junior high school students in rural China: Mediation effect of parental involvement. Behavioral Sciences, 14(3), Article 221. https://doi.org/10.3390/bs14030221

79. Sujarwo, S., & Herwin, H. (2023). Parental involvement and student achievement: A meta-analysis of publications in the scopus database. *International Journal of Instruction, 16*(2), 107-124. https://doi.org/10.29333/iji.2023.1627a

80. Epping, K.A. (2018). *The impact of parental involvement on student's academic achievement, parental well-being and parent-teacher relationships*. [Master Dissertation]. University of Calgary, Alberta. https://ucalgary.scholaris.ca/server/api/core/bitstreams/b5387ad0-d588-4c55-b2aa-4db26388fb7b/content

81. Avnet, M., Makara, D., Larwin, K. H., & Erickson, M. (2019). The impact of parental involvement and education on academic achievement in elementary school. *International Journal of Evaluation and Research in Education, 8*(3), 476-483. https://doi.org/10.11591/ijere.v8i3.20249

Click here to access/download
Supporting Information
S2_output@FactorAnalysis.pdf

Click here to access/download
Supporting Information
S1_dataset.pdf

Click here to access/download
Supporting Information
S2_output@FactorAnalysis.xlsx

Revised Manuscript with Track Changes

Determinants of instructional leadership: A survey of public secondary schools

Suman Pande[1*], Laxman Acharya[2]¶, Prakash C Bhattarai[3]¶

[1]Department of Development Education, Kathmandu University, School of Education, Hattiban, Lalitpur, Nepal

[2]Department of Development Education, Kathmandu University, School of Education, Hattiban, Lalitpur, Nepal

[3]Department of Development Education, Kathmandu University, School of Education, Hattiban, Lalitpur, Nepal

*Corresponding author

E-mail: suman_devpdh2023@kusoed.edu.np (SP)

¶ Conceptualization: Prakash C. Bhattarai
Formal analysis: Suman Pande
Methodology: Suman Pande, Prakash C. Bhattarai, Laxman Acharya
Supervision: Laxman Acharya
Writing – original draft: Suman Pande
Writing – review & editing: Suman Pande, Prakash C. Bhattarai, Laxman Acharya
~~These authors contributed equally to this work~~

Acknowledgements: The authors express their gratitude to all those head teachers and teachers of public secondary schools of Kathmandu valley who provided data for this study. The authors would also like to acknowledge Kathmandu University faculty members who directly or indirectly helped to do this research.

Abstract

Instructional leadership is centered on teaching and learning that academic leaders undertake to promote higher levels of student learning. Given its importance, this study examines the factors influencing instructional leadership in Kathmandu, Nepal. The framework for the study is grounded in Hallinger and Murphy's (1985) model of instructional leadership, which was employed as the conceptual basis. Using the Classical Delphi method, the model was adapted to develop a measurement scale, while the study was situated within a post-positivist paradigm, assuming that the reality of instructional leadership exists independently. Exploratory factor analysis was then conducted to extract the underlying factors. The findings reveal three major factors influencing instructional leadership: (a) planning, managing, and supervising instructional programs; (b) motivating and developing the competence of teachers and students; and (c) involving parents in the ~~instructional~~learning process. The inclusion of parental involvement emerged as a novel dimension in the context of Nepal. These results provide meaningful insights for school headteachers, other educational stakeholders, policymakers in the government sector, and international researchers interested in instructional leadership.

~~Instructional leadership is the leadership role associated with teaching and learning that academic heads must take on in order to demonstrate higher levels of student accomplishment. This study explores the factors influencing instructional leadership in Kathmandu, Nepal. Hallinger and Murphy (1985) model of instructional leadership is taken as the fundamental basis of this study. This model is subjected to Classical Delphi to develop a scale for this study. This model is subjected to Classical Delphi to develop a scale for this study. Exploratory factor analysis is conducted in the data to extract the factors. Three factors are formed and named as planning, managing and supervising instructional program; motivating and developing competence of teachers and students; and involving parents. The factors identified are supported by existing research. This research follows a post-positivist paradigm, which assumes that although the~~

Formatted: Line spacing: 1.5 lines

Formatted: Font color: Red

Formatted: Font color: Red

reality of instructional leadership exists independently, it can only be understood through data and evidence. Therefore, the study relies relying on statistical analysis of numerical data data, a method known as quantitative research. The study found that planning, managing and supervising instructional program; motivating and developing competence of teachers and students; and involving parents are the influencing factors of instructional leadership. The identified factors are supported by existing research. Among these, involving parents is a new concept developed in the context of Nepal. These e findings are s are valuable for school head teachers, schools' other stakeholders, concerned government officials of educational sector, and international researchers in instructional leadership.

Formatted: Font color: Red

Formatted: Font color: Red

Formatted: Font color: Red

Formatted: Font color: Red

Keywords

Instructional leadership, School, Head teacher, Student, Academic achievement, Factors

Introduction

The function of instructional leadership is vital in schools as it vastly influences the academic achievement of students [1, 2, 3]. In the schools of Nepal, the headteacher is only the academic leader who plays the role of instructional leader. In this regard, the head teachers are considered to be accountable for students' academic achievement. In Nepal, the policies for strengthening the role of the head teacher as an effective leader were enacted in the Education Rule 2002 [4]. However, some instructional leadership roles were also defined, but they were grouped with administrative roles and not specifically recognized as instructional leadership responsibilities. The other policies formulated for the development of school education are Three Year Interim Plan (2007/08 -2009/10) [5] and other sequential Three-Year Interim Plans, School Sector Reform Plan (SSRP) (2009/10-2015/16) [6] School Sector Development Plan (SSDP) (2016/17- 2022/23) [7], National Education Policy, 2076 B.S. [8], School Education Sector Plan (SESP) (2022/23- 2031/32) [9]. In these policies, the policymakers have considered the leadership of the head teacher as a way to enhance students' academic progress. However, the instructional leadership role as the duty of the head teacher has been mentioned clearly only in SSDP (2016/17- 2022/23) [7]. This demonstrates that the Ministry of Education within the Nepal Government has consistently worked to enhance Nepal's educational system. Still, the instructional leadership concept was introduced in the policy document only in 2016.

In spite of several efforts from the Nepal government to improve school education, satisfactory results have not been achieved by the public schools of Nepal for a long time. A review of the SLC exam results of Nepal from 1994 to 2013 showed that the average pass percentage of the SLC exam was only 45.50% over th~~ose 20 years'~~ose 20 years ~~period~~ [10]. After replacing the SLC with SEE and reforming the existing assessment system by changing the numerical grading system to a letter system through the Education Act 2016, the result has become even worse. The SEE results of recent years have shown a continuous downfall in public schools [11]. The result of SEE, which is evaluated in the letter grading system, is not satisfactory in Nepal [12]. The study of SEE result data showed that only 45 % of students were ~~over~~ above the satisfactory level in the academic year 2018 and 2019~~,~~; however, it reached ~~to~~ 78% in the academic year 2020 due to the use of student scores from schools rather than center-based exams in the framework of Covid-19 [13]. In 2021 ~~also~~, the SEE pass percentage increased as the board issued grade sheets based on internal evaluations conducted by individual

Formatted: Font: Times New Roman, 12 pt

Formatted: Normal

Formatted: Font: (Default) Times New Roman, 12 pt

schools in response to the pandemic [14]. In the subsequent years, the results reverted to a similar trend, with only 47.86% of students passing the SEE exam in 2024 [15]. Unsatisfactory school performance over the years is evident from the SLC (School Leaving Certificate) results before the Education Act 2016, and SEE (Secondary Education Examination) results after the Education Act 2016 [16, 12, 17, 11, 18, 19, 20, 21, ~~,~~22]. The unsatisfactory performance of public schools in Nepal is a blatant indication of the head teachers' inadequate effort in the teaching and learning process in these schools, a responsibility that falls under their instructional leadership role. ~~What could be the cause of this failure? To determine the cause of any occurrence, it is necessary to understand the factors influencing the outcome.~~ In this regard, there is ~~seems~~ an urgent need to identify ~~know~~ the influencing factors of ~~affecting~~ head teachers' ~~role in teaching and learning, which is called the~~ instructional leadership ~~role~~. Although there is some literature on instructional leadership, no study has been found that examines the factors influencing the instructional leadership of headteachers in the local context of Nepal. ~~Although there~~ ~~are~~ ~~is a large amount~~ ~~number~~ ~~of literature on instructional leadership, a study to find the factors that affect the instructional leadership of head teachers in the local context of Nepal has not been found so far in the literature.~~ Therefore, it was deemed imperative to identify the factors influencing headteachers' instructional leadership. Accordingly, the research question guiding this study is: What are the factors that affect headteachers' instructional leadership? Identifying these factors helps headteachers recognize areas where they may be falling short and where improvement is needed. This research is expected to support headteachers in developing their professional capacities as effective instructional leaders, which, in turn, can enhance students' achievement in schools. Furthermore, the findings of this study can contribute to the existing body of knowledge on instructional leadership. ~~Therefore, it~~ ~~was deemed~~ ~~seemed imperative to identify the factors affecting~~ ~~research the factors that influence the~~ ~~head teachers'~~ ~~instructional leadership of head teachers. So, the research question developed for this study is, what~~ ~~are the factors~~ ~~that affect~~ ~~affect head teachers' instructional leadership. Identifying the~~ ~~e factors that influence instructional leadership would help~~ ~~affecting~~ ~~head teachers'~~ ~~instructional leaderahip would help head teachers~~ ~~identify~~ ~~recognize areas~~ ~~where they are falling short and where they need to advance~~ ~~improve~~. ~~This research is believed~~ ~~expected to~~ ~~support~~ ~~be particularly helpful in helping~~ ~~head teachers build~~ ~~developing~~ ~~their professional abilities as effective instructional leaders~~, ~~which, in turn, can enhance~~. ~~This study can improve the students' achievement in the~~

Formatted: Space After: 0 pt, Line spacing: 1.5 lines

schools. classroom and Furthermore, the finding of this study can contribute to the existing body of knowlwdge on instructional leadership supplement to what is already known about instructional leadership.

Reviewing the Literature

Researchers attempted to examine school effectiveness or program improvement in the late 1970s. They first identified the effective schools and then analyzed what made them effective. They noticed that a skillful head teacher was a key factor in accounting for successful change or school improvement [23]. This effective school research base assigned the role of instructional leader to the head teachers to develop their skills because it was found that a head teacher would seem to be capable of fulfilling the head teacher's functions of managing the facility, controlling the operation, taking charge of community relations and overseeing the student progress only when he or she also becomes responsible for instructional leadership [24]. Researchers began examining school effectiveness and program improvement in the late 1970s. They first identified effective schools and then analyzed what made them successful, noting that a skillful head teacher was a key factor in driving school improvement [23]. It was also emphasized that instructional leadership plays a pivotal role in fostering school improvement, promoting better academic outcomes both directly, by supporting teachers, and indirectly, by improving conditions for teaching and learning. Successful school leadership involves improving conditions for teaching and learning, redesigning and enriching the curriculum, redesigning the roles and responsibilities, enhancing teacher quality, building relationships inside the school and community, enhancing teaching and learning and building relationship outside the school community[24]. As a result, instructional leadership appeared as an essential construct for making schools effective, but it was still unclear what the actual instructional leadership roles are.

Instructional leadership is a term construct derived form romed the by combination ing of the concepts of "instruction" and "leadership." There have been multiple efforts to define instruction, but four key dimensions of instruction remain consistent: setting learning objectives, choosing teaching methods, selecting reinforcement activities, and evaluating outcomes [25]. Researchers hold varying views on leadership as well. Some have defined leadership as how a

Formatted: Font: Times New Roman, 12 pt, Bold

Formatted: Space After: 0 pt, Line spacing: 1.5 lines

Formatted: Font: (Default) Times New Roman, 12 pt, Font color: Red

Formatted: Indent: First line: 0"

Formatted: Font color: Red

Formatted: Font: (Default) Times New Roman, 12 pt, Font color: Red

Formatted: Font color: Red

Formatted: Font: (Default) Times New Roman, 12 pt, Font color: Red

Formatted: Font color: Red

Formatted: Font: (Default) Times New Roman, 12 pt, Font color: Red

Formatted: Font color: Red

Formatted: Font: (Default) Times New Roman, 12 pt, Font color: Red

Formatted: Font color: Red

Formatted: Font color: Red

Formatted: Font: (Default) Times New Roman, 12 pt, Font color: Red

leader leads; others have described it as the job done through people [25]. Leadership includes followers acting toward goals that represent the values of the leaders and the followers [26]. Such uncertainty surrounding the concept of leadership has made the combined construct of “instructional leadership” difficult to define and clarify. The early instructional leadership models tended to focus on the leadership portion of the construct, and they attempted to define it in terms of general leadership theory. Those models focused on personality/~~-~~leadership style, behavioral/~~-~~situational, or contextual components of the head teacher [24].

In this manner, the combined construct of "instructional leadership" has been defined in various ways. Instructional leadership means providing the necessary resources, possessing knowledge and skill in curriculum and instructional matters, being a skilled communicator, and being a visionary who is out and around creating a visible presence [26]. Instructional leadership is direct assistance to teachers in their day-to-day activities, the development of collaborative groups among staff, the design and procurement of effective staff development, curriculum development, and the use of action research [27]. These definitions emphasized not only improving the instructional leadership role of the head teacher through the monitoring of instructional activities, but also focused on the managerial tasks of the head teacher within it. Later scholars of instructional leadership began linking it to activities aimed at enhancing teaching and learning, leading to a slight shift in how instructional leadership was defined. Here are some examples: ~~. The~~ instructional leadership begins when principals talk about being in the classroom, visit with students about their academic pursuits, and prioritize ~~se~~ instruction [28]. Instructional leadership is important for effective practices in curriculum, instruction, and assessment, and~~,~~ for the ability to work with teachers on the day-to-day problems related to these topics [29]. The latter two perspectives on instructional leadership place a strong emphasis on teaching and learning activities. In this way, the concept of instructional leadership evolved through various research developments, ultimately establishing that leadership roles connected to teaching and learning within the school fall under the domain of instructional leadership [30, 31, 2, 32].

The instructional leadership notion has dominated the educational literature since the creation of instructional leadership models, and there is ample proof of its effectiveness regarding teacher and student ~~results~~performance [33, 34]. The notable models of instructional leadership have been proposed by various researchers [e.g.,~~-~~35, 36, 37, 38, 39, 40, ~~-~~41]. They

Formatted: Font color: Red

have offered a variety of indications of instructional leadership, but the theoretical underpinnings are often the same [40, 41]. Researchers most extensively use the Hallinger and Murphy (1985) model of instructional leadership to ~~conduct do research on~~research instructional leadership. The widespread acceptance of this model is primarily due to Hallinger and Murphy's (1985) core concept, which provides head teachers with valuable insights into all aspects of leadership related to teaching and learning in schools. The main theme of this model is to develop and communicate school goals, coordinate and supervise the school curriculum, monitor and evaluate student progress, encourage teachers~~-~~ and students, and maintain high visibility and a hands-on approach [23, 44]. Their model is in the form of a scale known as PIMRS (Principal/~~-~~Head Teacher Instructional Management Rating Scale). This PIMRS has been utilized in at least 130 PhD dissertations over almost three decades [45].

Several academics have mentioned their views on the characteristics of an instructional leader. ~~The iI~~ Instructional leaders perform at high levels in four areas: a) resource provider, b) instructional resource, c) communicator, and d) visible presence in the school [35]. The instructional leaders emphasize~~ on~~: a) goal establishment and curriculum development, b) frequent observations, c) program evaluation, and d) support for teachers [41]. ~~The i~~ Instructional leaders perform seven crucial functions, which are: a) establishing and implementing instructional goals; b) being available to staff; c) creating a school culture and climate conducive to learning; d) communicating the vision and mission of the school to students, staff, and parents; e) setting high expectations for the staff; f) developing teacher leaders; g) maintaining positive attitudes toward student, staff, and parents [39]. There are four tenets of an instructional leader: a) focus on developing, directing, and supervising the curriculum and instruction in the classroom; b) interacting with ~~their~~ teachers to improve teaching and learning c) being goal-oriented; d) establishing high expectations and standards for teachers and students [38]. The effective instructional leader plays five important roles, which are: a) establishing goals and expectations; b) strategic resourcing; c) planning, coordinating, and evaluating teaching and the curriculum; d) promoting and participating in teacher learning and development; and e) ensuring an orderly and supportive environment [40]. There are six characteristics of effective instructional leaders: a) identifying and communicating~~ e~~ the mission and vision of the school; b) maintaining a school environment that is conducive to learning; c) ~~promote~~ promoting the professional development of staff; d) monitoring teachers' instruction; e) maintaining a positive

Formatted: Font color: Red

Formatted: Font color: Red

Formatted: Font color: Red

Formatted: Font color: Red

Formatted: Font color: Red

school climate; and f) ~~initiate~~ initiating organizational change [36]. From these scholars' views, it can be inferred that an instructional leader's job is ~~improving~~ to improve instruction to increase student achievement. Hence, an instructional leader is a person who seeks to ensure effective teaching and learning in the school by meeting all the requirements necessary to enhance students' academic performance.

~~Theoretical Underpinning~~

Among the models of instructional leadership proposed by various researchers devoted to the study of instructional leadership, Hallinger and Murphy's (1985) model of instructional leadership was used as a theoretical guide for this research because~~,~~ this model has attained top priority and is more domina~~t~~nt~~ed~~ in the literature on ~~of~~ instructional leadership. This model of instructional leadership consists of three factors: defining the school's mission, managing the instructional program, and promoting a positive learning climate. There are two functions under the factor defining the school's mission, which are framing the school goals and communicating the school goals; ~~, and~~ three functions under the factor managing the instructional program, which are supervising and evaluating instruction, coordinating curriculum, and monitoring students' progress; ~~,~~ and five functions under the factor promoting a positive learning climate which are protecting instructional time, ~~;~~ providing incentives for teachers, ~~;~~ providing incentives for learning, ~~;~~ promoting professional development of teachers, ~~;~~ and maintaining high visibility. There are five items under each of these ten functions, forming fifty variables altogether.

These fifty items were studied thoroughly, and a classical Delphi was conducted, followed by three rounds of meetings with experienced head teachers and teachers to get their concrete ideas on ~~it~~them. Based on their opinions, some items- ~~,~~ for example, (i) draw upon the results of class-wise testing when making curriculum decisions, (ii) participate actively in the review of curricular materials, (iii) acknowledge teachers' exceptional performance by writing memos for their personal files, (iv) recognize superior students' achievement or improvement by seeing in the office the students with their work, (v) review student work products when evaluating classroom instruction- -were ~~un~~ deselected from this PIMRS. Some items- ~~,~~ for example, (i) head teacher announces mandatory presence of parents during terminal report card distribution, (ii) head teacher meets the parents of poor-performing students and shares with them what roles the school and parents may together take for their improvements, (iii) head teacher learns students' family problems and talks to their parents to resolve them, (iv) the head

Formatted: Font color: Red
Formatted: Font color: Red
Formatted: Font color: Red
Formatted: Font color: Red
Formatted: Font color: Red
Formatted: Font color: Red
Formatted: Font color: Red
Formatted: Font color: Red
Formatted: Font color: Red
Formatted: Font color: Red
Formatted: Font color: Red
Formatted: Font color: Red
Formatted: Font color: Red
Formatted: Font color: Red
Formatted: Font color: Red
Formatted: Font color: Red

teacher is transparent about every financial detail of the school to all stakeholders~~-~~ -were added to the in new PIMRS.

Some items were changed to best suit ~~in~~ Nepali schools, such as (i) monitoring the classroom curriculum to see that it covers the school's curricular objectives was changed to the head teacher checking teachers' log books regularly to see if they are going in accordance with the syllabus, (ii) limiting interruption of instructional time by public address announcements was changed to head teacher trying ~~ies~~ his best to ensure uninterrupted instructional time, (iii) ~~(iv)~~ leading or attending teacher in- service activities concerned with instruction was changed to head teacher seeking ~~s~~ to provide training to teachers during vacations ~~or~~ so as not to interrupt daily classes, (v) conducting informal observations in classrooms regularly was changed to head teacher regularly monitoring ~~s~~ the activities of teachers and students while they are in class. In this way, ~~finally~~ a new PIMRS was finally developed for this research. This PIMRS consists of 11 factors and 33 items.

Research Methods

As the study intends to find out the determining factors of instructional leadership, it is the study of a *cause*-and-effect relationship. It is because those factors can be found out by studying the causes that influence instructional leadership. Therefore, it follows the post-positivistic deterministic philosophy of *causes* determine *effects*. The objective reality of instructional leadership exists out there, and that reality can be found ~~out~~ through careful study and measurement. Although the reality of instructional leadership exists independently, it can only be understood through data and evidence. Therefore, this study relies on statistical analysis of numerical data, a method known as quantitative research. The research ~~design is quantitative, and research~~ methods include the preparation of questionnaires, data collection, data analysis, and interpretation. Classical Delphi was conducted on Hallinger and Murphy's (1985) instructional leadership model, known as Principal/Head teacher Instructional Management Rating Scale (PIMRS) to develop a PIMRS suitable in the context of Nepal. A well-structured questionnaire was prepared using the new PIMRS. The questionnaire developed from the new PIMRS with 11 factors and 33 items asked the respondents to rate their view on a 5-point Likert scale with the descriptors~~;~~ : 1= almost never, 2= seldom, 3= sometimes, 4= frequently, and 5= almost always, ~~at~~ on which the head teacher was engaged in those particular activities of the school. The data was collected using a survey design, an appropriate way to get quantitative or

Formatted: Font color: Red

Formatted: Font color: Red

Formatted: Font color: Red

Formatted: Font color: Red

Formatted: Font color: Red

Formatted: Font color: Red

Formatted: Font color: Red

Formatted: Font color: Red

Formatted: Font color: Red

Formatted: Indent: First line: 0", Space After: 10 pt

Formatted: Font: 12 pt

Formatted: Font: 12 pt

Formatted: Font: 12 pt, Font color: Red

Formatted: Font color: Red

Formatted: Font color: Red

numerical descriptions of population trends, attitudes, or opinions by surveying a sample of that population [46]. ~~Questio~~A questionnaire was administered to respondents during visits to their schools.

The population of this study includes all public secondary schools in Kathmandu Valley. Kathmandu Valley was chosen for the study because it has the greatest concentration of public secondary schools compared to any other region in the nation. In total, 303 public secondary schools in the Kathmandu Valley made up the population of this study, and 172 schools made up the sample size. 172 schools were chosen at random from a pool of 303 to visit to gather data. Data was collected from one teacher and the head teacher, each from 172 schools, to see the difference in head teachers' and teachers' ratings. One teacher from each school was chosen by the lottery process. Factor analysis was conducted on the obtained data to identify the factors affecting head teachers' instructional leadership. The findings were analysed and interpreted in the light of the relevant literature.

Ethical standards were maintained throughout the research process, including during data collection. ~~Prior to~~Before initiating the study, ethical approval was obtained from the Research Committee of Kathmandu University, School of Education (Approval No. 181028-1). ~~Recruitment~~ The recruitment period for the pilot study was 24-07-2019 to 24-08-2019. Similarly, the recruitment period began ~~in~~ on 25-08-2019 and concluded on 05-02-2020 for the main study. Informed consent was secured from all participants. Head teachers, who served as respondents, were initially contacted by phone to request their time and contribution, while consent from the teacher respondents was obtained during school visits. A questionnaire with a cover letter was distributed to each respondent. Some respondents provided data on the same day, while others scheduled a later time. Thus, the data was gathered in accordance with established ethical standards.

The pilot testing of the questionnaire was done with the help of 30 head teachers and teachers from the schools, apart from the randomly selected 172 schools. A default sample size of 30 is advised for questionnaire pilot testing [47]. The majority of the items' mean scores on the questionnaire's pilot test were determined to be more than four times the standard deviation. A low standard deviation indicates that the majority of the numbers are extremely close to the average, demonstrating the normal distribution. This ensured the reliability of the questionnaire [48]. The data ~~was~~ were collected from 172 public schools of Kathmandu Valley with the help of

Formatted: Font color: Red

Formatted: Font color: Red

Formatted: Font color: Red

Formatted: Space Before: 0 pt, After: 0 pt

Formatted: Font: (Default) Times New Roman, 12 pt

this questionnaire, and the data were subjected to factor analysis to determine the factors influencing instructional leadership.

All the conditions required for factor analysis were checked. Factor analysis on Likert scale data is valued [49]. The criteria were met as a 5-point Likert- scale was used in this study. A lower number of categories cannot provide more possibilities and cannot help to obtain more reliable responses; on the other hand, a large range of scale impacts respondents' responses and reduces their chances of providing the proper answer on a specific item [50]. Therefore, I created a questionnaire using a 5-point Likert scale. The ratio of sample size ~~to~~ to variable should be three to six [51]. Since the sample size is 172 and the number of variables is 39, this is within the recommended range of 3:1 to 6:1. One of the most common situations is the retention of factors with Eigenvalues greater than one [52, 53]. In this study, all factors had Eigenvalues greater than 1~~,~~; hence, this criterion was also met. This study also met the requirement of item loading .40 to .50 [54] as the item loading was .50.

Formatted: Font: (Default) Times New Roman

Another condition is that the average commonalities or extraction value of items is more than 0.5 [54]. The average extraction value for items was .593. Therefore, this requirement was also fulfilled. Another criterion is Kaiser-Meyer sampling adequacy. If the KMO measure is greater than .50, it is safe to assume that the factor ability exists in the dataset [52, 55]. The Kaiser-Meyer sampling adequacy score was .896. So, this condition was also deemed met. A further requirement is that a factor must have at least three components to be considered genuine [56, 54]. This criterion was also fulfilled because there were three to fourteen items in each of the three instructional leadership factors. All the conditions for factor analysis were met, and three factors of instructional leadership were obtained. The Cronbach's alpha value of instructional leadership factors ranged between .75 to .92. The literature suggests that an alpha value of .75 to .95 is adequate [57]. Therefore, that was acceptable. Thus, the reliability of factor analysis was assessed.

Result

~~The factor analysis yielded 25 items under three factors. Fourteen of these twenty-five variables fall under factor one, eight under factor two, and three under factor three.~~ The factor analysis yielded three instructional leadership factors. Fourteen items loaded on the first factor, eight on the second, and three on the third.

Factor One: Planning, managing, and supervising instructional program

All the items of factor one are shown below in the rotated component matrix of this factor.

Table 1

Rotated Component Matrix of Factor One of IL

~~Components~~

~~1~~ ~~2~~ ~~3~~

SN Items	Factors 1	2	3
1. ~~H~~The head teacher analyses students' performance ~~of~~from the last year while planning for the new academic session.	.610		
2. ~~H~~The head teacher specifies the school's whole year educational plan in the presence of all concerned people.	.652		
3. ~~H~~The head teacher seeks to incorporate teachers' inputs in planning during his formal or informal meetings.	.588		
4. ~~H~~The head teacher shares academic plans with everyone involved and discusses how those plans may be effectively implemented.	.651		
5. ~~H~~The head teacher displays the school's whole year plan on the notice boards and communicates ~~them~~it to students during assembly.	.639		
6. ~~H~~The head teacher reports educational planning of the school to the local government and the Province/Local level education offices.	.604		
7. ~~H~~The head teacher regularly monitors the activities of teachers and students while they are in class.	.634		
8. ~~H~~The head teacher makes sure that students' every class work/home-work is completed timely and all notes are marked by assigned teachers at least a month ~~prior to~~before terminal examinations.	.695		
9. ~~H~~The head teacher seeks to find out the teachers' areas of improvements in instructional practices.	.711		
10. ~~H~~The head teacher gets every subject department head to implement the syllabus of all subjects in each class.	.653		
11. ~~H~~The head teacher checks teachers' log books regularly to see if they are going in accordance with the syllabus.	.630		
12. The Head teacher reviews whether the curriculum goal is achieved through students' terminal examination results, their discipline, and overall change indicators	.664		
~~H~~The head teacher regularly discusses with subject teachers regarding each student's	.530		
13. progress	.528		
14.~~6.2~~ The ~~H~~head teacher instructs teachers to ensure no student has bunked classes			

Formatted: Line spacing: single

Formatted: Line spacing: single, Position: Horizontal: Left, Relative to: Column, Vertical: In line, Relative to: Margin, Horizontal: 0", Wrap Around

Formatted Table

Formatted: Line spacing: single, Position: Horizontal: Left, Relative to: Column, Vertical: In line, Relative to: Margin, Horizontal: 0", Wrap Around

Formatted: Left

Formatted: Centered

Formatted: Line spacing: single, Position: Horizontal: Left, Relative to: Column, Vertical: In line, Relative to: Margin, Horizontal: 0", Wrap Around

Formatted: Line spacing: single, Position: Horizontal: Left, Relative to: Column, Vertical: In line, Relative to: Margin, Horizontal: 0", Wrap Around

Formatted: Line spacing: single, Position: Horizontal: Left, Relative to: Column, Vertical: In line, Relative to: Margin, Horizontal: 0", Wrap Around

Formatted: Line spacing: single, Position: Horizontal: Left, Relative to: Column, Vertical: In line, Relative to: Margin, Horizontal: 0", Wrap Around

Formatted: Line spacing: single, Position: Horizontal: Left, Relative to: Column, Vertical: In line, Relative to: Margin, Horizontal: 0", Wrap Around

Formatted: Line spacing: single, Position: Horizontal: Left, Relative to: Column, Vertical: In line, Relative to: Margin, Horizontal: 0", Wrap Around

Formatted: Line spacing: single, Position: Horizontal: Left, Relative to: Column, Vertical: In line, Relative to: Margin, Horizontal: 0", Wrap Around

Formatted: Line spacing: single, Position: Horizontal: Left, Relative to: Column, Vertical: In line, Relative to: Margin, Horizontal: 0", Wrap Around

Formatted: Line spacing: single, Position: Horizontal: Left, Relative to: Column, Vertical: In line, Relative to: Margin, Horizontal: 0", Wrap Around

Formatted ...

Formatted ...

Formatted ...

Formatted ...

Formatted: Font: (Default) Times New Roman, 12 pt

Formatted ...

Formatted ...

Components	1	2	3
1. Head teacher analyses students' performance of the last year while planning for the new academic session	.610		
2. Head teacher specifies school's whole year educational plan in the presence of all concerned people	.652		
3. Head teacher seeks to incorporate teachers' inputs in planning during his formal or informal meetings	.588		
4. Head teacher shares academic plans with everyone involved and discusses how those plans may be effectively implemented	.651		
5. Head teacher displays school's whole year plan on the notice boards and communicates them to students during assembly	.639		
6. Head teacher reports educational planning of school to the local government and Province/Local level education offices	.604		
7. Head teacher regularly monitors activities of teachers and students while they are in class	.634		
8. Head teacher makes sure that students' every class work/home work is completed timely and all notes are marked by assigned teachers at least a month prior to terminal examinations	.695		
9. Head teacher seeks to find out teachers' areas of improvements in instructional practices	.711		
10. Head teacher gets every subject department head to implement the syllabus of all subjects in each class	.653		
11. Head teacher checks teachers' log books regularly to see if they are going in accordance with the syllabus	.630		
12. Head teacher reviews whether curriculum goal is achieved through students' terminal examination results, their discipline and overall change indicators	.664		
13. Head teacher regularly discusses with subject teachers regarding each student's progress	.530		
6.2 Head teacher instructs teachers to ensure no student has bunked classes	.528		

Explanation of Variables in Factor One

These fourteen variables are connected to five distinct instructional leadership roles: planning and controlling school educational activities, overseeing and evaluating instruction, coordinating the curriculum and other educational objectives, keeping track of students' progress, and utilizing instructional time. These specific instructional leadership functions of the head teacher, except ~~a last one,~~ the head teacher directs teachers to make sure no student has skipped class, are similar to the items in Hallinger and Murphy's (1985) framework of IL falling under their two factors, defining the school mission, and managing instructional program [58, 59, 60, -61]. Therefore, by including the meaning of those three leadership functions, namely defining the school mission, managing the instructional program, and ensuring no student has bunked the class, this factor was appropriately named as planning, managing, and supervising the instructional program.

All ~~T~~ the variables of the factor planning, managing, and supervising instructional program show that it is about making an educational plan for the school, coordinating the curriculum, sharing plans, observing instructors to ensure they are practicing good teaching techniques, communicating with teachers to learn about their students' development, and making attempts to improve. There were two factors: framing and communicating school goals, and supervising and evaluating instruction for these particular instructional leadership activities in Hallinger and Murphy's (1985) model [37]. They mentioned, it is about setting and expressing goals, working with staff members and teachers, and managing the instructional process and curriculum. According to [62] ~~It~~ this involves establishing the school's goals, reminding teachers of ~~the~~ its purposes and mission, and informing teachers that teachers need to plan their classes properly ~~[62]~~. A school lacks any standards to assess its success ~~if it has no~~ without a plan ~~for~~ to improve theing the educational process. As stated by [63] ~~T~~ this factor relates to selecting the schools' primary goals ~~is concerned with choosing the primary goals of the school~~ as well as coordinating and managing curriculum and instruction ~~[63].~~ Similarly, [64] notes that ~~P~~ planning, managing, and supervising instructional programs entails ~~about~~ articulating and expressing the school's goals, coordinating the curriculum, and monitoring and controlling the program by supervising and evaluating instruction ~~[64]~~.

Formatted: Font: Bold

Formatted: Indent: First line: 0"

Formatted: Right: 0", Adjust space between Latin and Asian text, Adjust space between Asian text and numbers

Formatted: Font color: Red

Formatted: Font: (Default) Times New Roman, 12 pt, Font color: Red

Formatted: Normal, Right: 0.04", Don't adjust space between Latin and Asian text, Don't adjust space between Asian text and numbers

Formatted: Font: (Default) Times New Roman, 12 pt

Formatted: Font: (Default) Times New Roman, 12 pt

Formatted: Font: (Default) Times New Roman, 12 pt, Font color: Blue

Formatted: Font: (Default) Times New Roman, 12 pt

Formatted: Font: (Default) Times New Roman, 12 pt

Formatted: Font: (Default) Times New Roman, 12 pt, Font color: Blue

Formatted: Font: (Default) Times New Roman, 12 pt

Formatted: Font: (Default) Times New Roman, 12 pt

Formatted: Font: (Default) Times New Roman, 12 pt, Font color: Blue

Formatted: Font: (Default) Times New Roman, 12 pt

Formatted: Font: (Default) Times New Roman, 12 pt

This factor also resonates with the conceptualization of instructional leadership by [65], which is grounded in head teachers' core practices and was developed to explain how Norwegian school leaders build their instructional capacity. Their multidimensional model, consisting of goal orientation, collaboration with teachers, professional development, observation, and supervision, is similar to the variables of the factor planning, managing, and supervising instructional program identified in this study. Both undersore head teachers' active role in shaping and monitoring instructional processes as a foundation for improving teaching and learning.

This finding can also be compared with those [66], where coordinating the curriculum and providing professional learning opportunities to teachers emerged as statistically significant predictors of teachers' professional development in the context of Nigeria. Coordinating the curriculum is parallel to the variable "the head teacher gets every subject department heads to implement the syllabus of all subjects in each class" within the factor of planning, managing, and supervising instructional program. This reinforces the importance of this variable for developing teaching and learning ability. The next predictor, providing professional learning opportunities to teachers, corroborates the variables of the second factor, motivating and developing the competence of teachers and students developed in this study.

Factor Two: Motivating and developing the competence of teachers and students

All the items of factor two are shown below in the rotated component matrix of this factor.

Table 2

Rotated Component Matrix of Factor Two of IL

Components

1 2 3

SN	Items	Factors 1	2	3
1.	Head The head teacher publicly praises teachers' superior performance, but meets them in private for correction.	.608		
1.	Head teacher tries to manage opportunity for sincere teachers to work in a higher position as a reward for their good work	.668		

Formatted: Font color: Red
Formatted: Font color: Red
Formatted: Space Before: 0 pt, After: 0 pt
Formatted: Font color: Light Blue
Formatted: Font color: Red
Formatted: Font color: Red
Formatted: Font color: Red
Formatted: Font color: Red
Formatted: Font: (Default) Times New Roman, 12 pt, Font color: Red
Formatted: Normal
Formatted: Font: (Default) Times New Roman, 12 pt, Font color: Light Blue
Formatted: Font: (Default) Times New Roman, 12 pt, Font color: Red
Formatted: Font: (Default) Times New Roman, 12 pt, Font color: Red
Formatted: Font: (Default) Times New Roman, 12 pt, Font color: Red
Formatted ...
Formatted ...
Formatted ...
Formatted ...
Formatted ...
Formatted ...
Formatted ...
Formatted Table
Formatted ...
Formatted ...
Formatted: Space After: 0 pt, Line spacing: single
Formatted: Centered
Formatted: Centered
Formatted: Indent: Left: -0.25", Line spacing: single
Formatted: Centered, Line spacing: single
Formatted: Font: (Default) Times New Roman, 12 pt
Formatted ...
Formatted: Line spacing: single
Formatted: Indent: Left: 0", Line spacing: single
Formatted ...

	Head teacher provides letter of appreciation or honor certificates to teachers for their contribution	.725
2.	The head teacher tries to manage opportunities for sincere teachers to work in a higher position as a reward for their good work.	668
3.	The head teacher provides a letter of appreciation or honor certificates to teachers for their contribution.	.725
5. .	Head teacher seeks to provide training to teachers during vacations or so as not to interrupt daily classes.	.735
7. .	Head The head teacher actively supports teachers to use those learnt skills in the classrooms.	.695
9. .	7. Head The head teacher sometimes provides opportunities for observation or an educational tour for teachers.	.719
11. .	8. Head The head teacher publicly honors students for their excellent performance or discipline.	.489
13. .	9. Head The head teacher learns about students' family problems and talks to their parents to resolve them.	.549

Explanation of Variables in Factor Two

Factor two consists of eight variables, which explain the role of an instructional leader in motivating and supporting teachers and students for their academic growth. These eight variables serve similar functions to the variables in the Hallinger and Murphy's (1985) Framework of instructional leadership, placed under the functions providing incentives for teachers, providing incentives for learning, and promoting professional development under the factor creating a positive school climate [58, 59, 60, -61]. Since the obtained eight variableseight variables obtained of from this factor share the same aim of inspiring teachers and students for their development, a suitable name, 'motivating and developing the competence of teachers and students', was given to this factor.

T All- the variables of the factor motivating and developing the the competence of teachers and students show that it is about encouraging teachers and students to develop their abilities and academic excellence. Praising and rewarding teachers and students for their good deeds, assisting teachers with their professional growth, and supporting students in improving their self-confidence and academic focus are the key roles of an instructional leader, which are crucial in achieving academic success in the school. As stated by [62], R recognizing and rewarding teachers' and students' accomplishments is important for fostering their interest and engagement to develop interest in them [62]. These instructional leadership roles correspond to

Formatted: Centered, No bullets or numbering
Formatted: Centered
Formatted: Centered, No bullets or numbering
Formatted: Centered
Formatted: Centered, No bullets or numbering
Formatted Table
Formatted: Indent: Left: -0.25", Line spacing: single
Formatted: Centered, Line spacing: single
Formatted: Line spacing: single
Formatted: Indent: Left: 0", Line spacing: single
Formatted: Centered, No bullets or numbering
Formatted: Indent: Left: -0.25", Line spacing: single
Formatted: Centered, Line spacing: single
Formatted: Line spacing: single
Formatted: Indent: Left: 0", Line spacing: single
Formatted: Centered, No bullets or numbering
Formatted: Indent: Left: -0.25", Line spacing: single
Formatted: Centered, Line spacing: single
Formatted: Line spacing: single
Formatted: Indent: Left: 0", Line spacing: single
Formatted: Centered, No bullets or numbering
Formatted: Indent: Left: -0.25", Line spacing: single
Formatted: Centered, Line spacing: single
Formatted: Line spacing: single
Formatted: Indent: Left: 0", Line spacing: single
Formatted: Centered, No bullets or numbering
Formatted: Indent: Left: -0.25", Line spacing: single
Formatted: Centered, Line spacing: single
Formatted: Line spacing: single
Formatted: Indent: Left: 0", Line spacing: single
Formatted: Font: Bold
Formatted: Font: Bold
Formatted: Indent: First line: 0"
Formatted: Font: (Default) Times New Roman, 12 pt
Formatted: Font color: Blue

align with ~~-~~two functions in Hallinger and Murphy's (1985) instructional leadership model, ~~:~~ promoting professional development~~,~~ and ~~offering~~ providing incentives for learning. According to [64], ~~T~~ this factor ~~focuses~~ emphasizes ~~on~~ inspiring and ~~fostering~~ enhancing the competence of both teachers~~'~~ and students ~~' competence [64]~~. The overall focus is on helping both teachers and students get better.

The variables within this factor, such as offering training to teachers during vacations, supporting the classroom application of acquired skills, and facilitating educational tours, corroborate the finding of [66], who found that providing professional learning opportunities to teachers supports their professional development. In contrast to the finding of [66], who did not find providing incentives for teachers to be a significant predictor of teachers' professional development, the present study suggests that motivational strategies such as public praise, appreciation letters, honor certificates, and rewarding sincere teachers with opportunities for career development are integral to strengthening teachers' competence. Additionally, the inclusion of student~~-~~-focused practices such as honoring excellent performance and addressing family problems reflects a broader and more relational approach for both teachers' and learners' holistic development.

Factor Three: Involving Parents

All the items of factor three are shown below in the rotated component matrix of this factor.

Table 3

Rotated Component Matrix of Factor Three of IL

~~Components~~

~~12 3~~

Items	Factors		
	1	2	3
1. ~~H~~The head teacher calls class class-wise parents' meeting and discusses student~~'s~~s' progress.			.553
2. The h~~H~~ead teacher announces the mandatory presence of parents during the terminal report card distribution.			.839
3. ~~H~~The head teacher meets the parents of poor performing students and shares with them what roles the school and parents may together take for their			

Formatted: Font color: Blue
Formatted: Font color: Red
Formatted: Space Before: 0 pt, After: 0 pt
Formatted: Font color: Red
Formatted: Font color: Light Blue
Formatted: Font color: Red
Formatted: Font color: Red
Formatted: Font color: Red
Formatted: Font color: Red
Formatted: Font color: Light Blue
Formatted: Font color: Red
Formatted: Font color: Red
Formatted: Space After: 10 pt, Line spacing: Multiple 1.15 li
Formatted: Space After: 0 pt, Line spacing: single
Formatted Table
Formatted: Left, Space After: 0 pt, Line spacing: single
Formatted: Space After: 0 pt, Line spacing: single
Formatted: Centered
Formatted: Line spacing: single
Formatted: Space Before: 0 pt, Line spacing: single
Formatted: Line spacing: single
Formatted: Space Before: 0 pt, Line spacing: single
Formatted: Space Before: 0 pt, Line spacing: single
Formatted: Line spacing: single
Formatted: Space Before: 0 pt, Line spacing: single

improvements.	.653

Table 3

Rotated Component Matrix of Factor Three of IL

Explanation of Variables in Factor Three

The factor three of instructional leadership consists of three items. These items are linked to the

Components	1	2	3
1. Head teacher calls class wise parents' meeting and discusses student's progress			.553
2. Head teacher announces mandatory presence of parents during terminal report card distribution			.839
3. Head teacher meets the parents of poor performing students and shares with them what roles the school and parents may together take for their improvements.			.653

head teachers' policy to get parents/guardians involved in knowing about their children's condition and taking the appropriate action if necessary. In accordance with the meaning of these variables, a suitable name involving parents was given to this factor.

The core idea summarized in the variables of this factor is to enhance parent/guardian-school communication for students' academic development. The parents/guardians are also made accountable, along with the school, through this strategy. Parental role is found to be have given more importantnt nce to students' academic progress in the Nepali context. There are studies thatSome studies have shown the importance of the parent-school relationship. For example,

Formatted: Line spacing: single
Formatted: Font: Not Bold
Formatted: Space After: 10 pt, Line spacing: Multiple 1.15 li
Formatted: Font: Not Italic
Formatted: Space After: 10 pt, Line spacing: Multiple 1.15 li, Tab stops: Not at 0.31" + 0.38" + 0.44"
Formatted: Space After: 0 pt, Line spacing: 1.5 lines
Formatted: Tab stops: Not at 0.31" + 0.38" + 0.44"
Formatted: Indent: First line: 0", Tab stops: Not at 0"
Formatted: Indent: Left: 0", First line: 0", Right: 0", Line spacing: Multiple 1.15 li, Widow/Orphan control, Adjust space between Latin and Asian text, Adjust space between Asian text and numbers
Formatted: Line spacing: Multiple 1.15 li, Widow/Orphan control, Adjust space between Latin and Asian text, Adjust space between Asian text and numbers
Formatted: Left, Right: 0", Line spacing: Multiple 1.15 li, Widow/Orphan control, Adjust space between Latin and Asian text, Adjust space between Asian text and numbers
Formatted: Normal, Line spacing: single, No bullets or numbering, Widow/Orphan control, Adjust space between Latin and Asian text, Adjust space between Asian text and numbers
Formatted ...
Formatted ...
Formatted ...
Formatted ...
Formatted ...
Formatted ...
Formatted ...
Formatted ...
Formatted: Font: Times New Roman, 12 pt
Formatted: Normal, Line spacing: 1.5 lines
Formatted: Font: Times New Roman, 12 pt
Formatted: Font: Times New Roman, 12 pt
Formatted: Font: Times New Roman, 12 pt
Formatted: Font: Times New Roman, 12 pt

~~Although there are~~ some studies ~~studies~~ [67~~5~~, 68~~6~~] ~~that have~~ have shown~~n~~ a positive relationship between parental involvement and students' academic achievement, [69] reported that the increasing parental and community involvement in the educational process results in stronger relationships between school leaders and the community. Similarly, [70] emphasized the need for developing relational behavior for school administrators, including interaction with parents. ~~, in the international context as well, but the parent's involvement is not mentioned as a factor in the researchers' instructional leadership models whoever have proposed such model.~~ A recent research [71] conducted in Hong Kong, and Macao also demonstrated that leadership can sinfluence student achievement through Parental Academic Commitment (PAC). This reinforces the idea that parent involvement is not just a peripheral activity but a leadership-mediated pathway to better student outcomes.

Discussion

The meaning of the variables under three different factors, the planning, managing, and supervising instructional program**;** motivating and developing competence of teachers and students; and involving parents, are closely aligned with some international findings on factors influencing leadership that contribute to improving teaching and learning in the schools. For example, these three factors identified in this study are parallel to the leadership pathways proposed by [72] in their study conducted in Chicago, USA. The planning, managing, and supervising instructional program aligns with the '~~"~~learning climate'~~"~~ pathway in which head teachers establish conditions necessary to maintain a strong instructional focus. Motivating and developing the competence of teachers and students reflects the '~~"~~professional capacity'~~"~~ pathway, emphasizing the development of teachers' skills, professional quality, and capacity to work together. Finally, involving parents directly corresponds to the '~~"~~parent-community ties'~~"~~ pathway, which strengthens collaboration between parents and school to enhance student outcomes. Again, [72] concluded that leadership affects students' achievement primarily by improving the learning climate, which further supports the planning, managing, and supervising instructional program as a factor for enhancing students' academic outcomes through improved teaching and learning.

It also seems informative to compare the findings of the present study with the research on head teachers' competencies by [73] in Vietnam because instructional leadership is one of the

Formatted: Font: Times New Roman, 12 pt, Font color: Light Blue
Formatted: Font: Times New Roman, 12 pt
Formatted: Font: Times New Roman, 12 pt
Formatted: Font: Times New Roman, 12 pt, Font color: Red
Formatted: Font: Times New Roman, 12 pt
Formatted: Font: Times New Roman, 12 pt, Font color: Light Blue
Formatted: Font: Times New Roman, 12 pt
Formatted: Font: Times New Roman, 12 pt, Font color: Red
Formatted: Font color: Red
Formatted: Adjust space between Latin and Asian text, Adjust space between Asian text and numbers, Tab stops: 0", Left
Formatted: Font: Not Bold
Formatted: Font: Not Bold
Formatted: Font: Bold
Formatted: Font color: Light Blue
Formatted: Font color: Auto
Formatted: Font color: Auto
Formatted: Font: Not Italic
Formatted: Font: Not Bold
Formatted: Font: Not Italic
Formatted: Font: Not Italic
Formatted: Font: Not Bold
Formatted: Font: Not Italic
Formatted: Font: Not Bold
Formatted: Font color: Auto
Formatted: Font color: Light Blue
Formatted: Font color: Auto
Formatted: Font color: Auto
Formatted: Font color: Auto
Formatted: Font color: Auto
Formatted: Font color: Auto
Formatted: Font color: Light Blue
Formatted: Font color: Auto

various competencies of head teachers. Three of the eight competency domains identified in the Vietnam study, the ‘instructional program management’, ‘staff development’, and ‘engagement with families’, closely align with the three factors found in the present study. This suggests that the three dimensions developed in the present study are not only central to teaching and learning improvement in Nepal but are also recognized internationally as essential aspects of effective school leadership. A meta-analysis [74] covering 24 studies and 9,178 teachers revealed that the head teachers' instructional leadership is moderately related to teacher self-efficacy, which results in enhanced student achievement. This finding aligns with the result of the present study, where motivating and developing the competence of teachers and students emerged as one of the factors of instructional leadership. A systematic review of 349 Asian studies by [75] revealed that the PIMR (Principal/head teacher Instructional Management Rating Scale) dominates the literature focusing on managing instructional programs and promoting a positive learning climate. In this regard, the first two factors of the present study parallel those dimensions.

"Involving parents" is a new factor developed in this study. The role of head teachers in involving parents as one of the factors of head teachers' instructional leadership in the context of Nepal is a noteworthy finding. Although studies have shown that parental involvement is crucial for students' achievement, none of the established instructional leadership frameworks explicitly recognize it as a distinct factor of instructional leadership.

The role of head teachers in engaging parents as one of the factors influencing head teachers' instructional leadership in the context of Nepal is a noteworthy finding because parental contribution to students' progress as one of the factors of instructional leadership has not been mentioned yet in any of the proposed instructional leadership models. Regular communication between head teachers and parents encourages parents to take more responsibility for their children's growth and to pay more attention to their educational needs. Increasing parental and community involvement in the educational process results in stronger relationships between school leaders and the community [67]. There is need of developing relational behavior for school administrators, including interaction with parents [68]. Furthermore, regular communication with parents assists head teachers in avoiding unwarranted

Formatted: Font color: Light Blue

Formatted: Font color: Red

Formatted: Font color: Light Blue

Formatted: Font color: Red

Formatted: Font color: Auto

Formatted: Font color: Auto

Formatted: Space Before: 0 pt, After: 0 pt

Formatted: Font color: Red

complaints, as parents stay informed about their children's progress and collaborate with the school to encourage their children's development.

An Indonesian study, showed a positive correlation between parental participation and academic achievement in school [76 69]. He found that students with active parental involvement had higher test scores and grade point averages. Many studies have shown that parental participation positively influences students' educational performance. A favorable and modest impact of parental involvement on students' academic attainment was found in a meta-analysis of 37 research studies published between 2000 and 2013 in kindergarten, primary, and secondary schools [770]. The mediation test results of [78] revealed a partially mediating role of parental involvement in the relationship between socioeconomic status and students' academic achievement, as well as a strong mediating effect between family environment and academic achievement in rural China. A meta-analytic study with a random effects approach by [79] across Scopus-indexed publications showed a significant positive correlation between parental involvement and student achievement.

Formatted: Space Before: 0 pt, After: 0 pt

Formatted: Font color: Light Blue

Formatted: Font color: Light Blue

While substantial research supports the positive impact of parental involvement on student achievement, some studies indicate that parental involvement is not always beneficial. However, some researchers found no relationship between parental involvement and students' accomplishments. A study conducted in Canada showed no significant results for the influence of parental participation on students' academic progress [80]. A study conducted in the United States of America involving 450 students from kindergarten to fourth grade, showed that the students who scored higher academically had lower parental involvement in school [81]. Such findings imply that the country's and locality's cultural and societal institutions and practices may determine whether or not parental participation influences students' academic achievement [72].

Formatted: Font color: Light Blue

Formatted: Font color: Light Blue

Conclusion

The study concludes that there are three factors determining instructional leadership In response to the research question, what factors affect head teachers' instructional leadership, the study identified three factors. The first factor, planning, managing, and supervising instructional

programs, explains the head teacher's role in collaborating with teachers to ensure that the school has a clear mission and that the mission concentrates on students' academic achievement. The second factor, motivating and developing the ~~the~~ competence of teachers and students, describes the head teacher's activities to boost teachers' and students' interest in their line of work. The third factor, involving parents, mentions the roles of parents/ guardians in association with the school for students' academic progress. The study's key finding is that the parents'/ guardians' involvement in students' progress is seen as an essential factor in the schools of Nepal, which is different from Hallinger and Murphy's (1985) model of instructional leadership. In Hallinger and Murphy's (1985) instructional leadership model, the parents'/guardians' involvement is not explicitly recognized as a factor in student success. Instead, the model includes only one related item, contacting parents to communicate improved or exemplary student performance or contributions, which falls under the factor ‘"providing incentives for learning’". This suggests that Nepal's unique cultural and societal framework places greater emphasis on the role of parents/ guardians in students' academic achievement. By identifying these factors, this study contributes to the field of instructional leadership and underscores specific requirements for effective teaching and learning in the public schools of Nepal.

Implications of the Study

The results of this study can help head teachers stay informed in their role as instructional leaders by providing recommendations for enhancing teaching and learning in schools**.** The finding can also assist policymakers and bureaucrats in formulating and implementing necessary policies to enhance head teachers' instructional leadership. Additionally, it can support local authorities in improving schools, as the responsibility for school development is entrusted to local bodies in Nepal's federal democratic system. In this way, the result of this study can be very useful for making schools effective and attaining quality education. Further, this study has added new knowledge to the construct of instructional leadership in the local Nepali context by identifying the factors that influence it, which may be of interest to international researchers of this field.

Formatted: Font: (Default) Times New Roman, 12 pt

Acknowledgements The authors express their gratitude to all those head teachers and teachers of public secondary schools of Kathmandu valley who provided data for this study. The authors would also like to acknowledge Kathmandu University faculty members who directly or indirectly helped to do this research.

Funding

The authors have not got financial support for the research, authorship, and/or publication of this article.

Competing interests

The authors declare that they have no conflict of interest.

Formatted: Font: 12 pt

Ethical approval

The authors declare that they follow ethical standards

References

1. –Bhujel, C. B. (2021).

The role of principal in improvement of school performance: A qualitative study in community school of Nepal.

——*Artech J. Art Social Sci.*, *3*, 11-22. https://doi.org/10.32861/rje.71.1.10

2. –Hallinger, P., & Murphy, J. (2012).

——Running on empty? Finding the time and capacity to lead learning.

- —*NASSP Bulletin*, *97*(1), 5-21. https://doi.org/10.1177/0192636512469288

3. –Lamsal, B. (2022).

Instructional leadership behavior among principals of two schools in Lalitpur, Nepal.

TVET Journal, *1*(16), 50 59. https://doi.org/10.3126/tvet.v1i16.45186

4. Nepal Law Commission. (2002). *Education regulation 2002*.

https://lawcommission.gov.np/content/12987/12987-education-regulation-2059/

~~4.~~ 5. ~~www.lawcommission.gov.np/en/ documents/2015/08/education-rules-2059-2002.pdf.~~

~~5.~~ Nationa~~epa~~l Planning Commission. (2007).

Three-year interim plan (2007/08--2009/10).

Government of Nepal. https://www.yumpu.com/en/document/view/6747910/three-year-interim-plan-2007-08-2009-10

~~6.~~ 6. Ministry of Education. (2009).

School sector reform plan ~~, Nepal, (~~*2009/10--2015/16*). Government of Nepal.

https://www.globalpartnership.org/node/document/download?file=sites/default/files/SSRP.pdf

~~7.~~ 7. Ministry of Education. (2016).

Formatted: Indent: Left: 0"

Formatted: Font: (Default) Times New Roman, 12 pt

Formatted: Normal, Line spacing: 1.5 lines

Formatted: Indent: Left: 0.25"

Formatted: Normal, Line spacing: 1.5 lines

Formatted: Font: (Default) Times New Roman, 12 pt

Formatted: Normal, Indent: Left: 0"

Formatted: Line spacing: 1.5 lines

Formatted: Font: (Default) Times New Roman, 12 pt

Formatted: Normal, Indent: Left: 0"

Formatted: Font: (Default) Times New Roman, 12 pt

Formatted: Normal, Line spacing: 1.5 lines

Formatted: Font: (Default) Times New Roman, 12 pt

Formatted: Normal, No bullets or numbering

Formatted: Font: (Default) Times New Roman, 12 pt, Italic

Formatted: Normal, No bullets or numbering, Tab stops: Not at 0.06"

Formatted: Normal, Indent: Left: 0", Tab stops: Not at 0.06"

Formatted: Font: (Default) Times New Roman, 12 pt

Formatted: Normal, No bullets or numbering

Formatted: Font: (Default) Times New Roman, 12 pt

Formatted: Normal, Line spacing: 1.5 lines

Formatted: Normal, No bullets or numbering

Formatted: Font: (Default) Times New Roman, 12 pt

School sector development plan~~,~~. ~~Nepal,~~ *2016/17–20*2~~1~~*2/23*. Government of Nepal. https://planipolis.iiep.unesco.org/sites/default/files/ressources/nepal_ssdp_final_document_oct_2016_0.pdf

8. CollegeNP. (2019, December 12). *National education policy of Nepal 2076 / 2019.* CollegeNP. https://www.collegenp.com/news/national-education-policy-2076

~~8.~~ 9. ~~Ministry of Education. (2019).~~

~~*National education policy 2076 (2019/ 020).*~~

~~9.~~ Ministry of Education, Science and Technology (2022).

School ~~*e*~~*Education* ~~*s*~~*Sector* ~~*p*~~*Plan, (2022/023-2031/032)*. Government of Nepal. https://planipolis.iiep.unesco.org/sites/default/files/ressources/nepal_school_education_sector_plan_2022-23-2031-32_.pdf

10.

~~10.~~ Bhattarai, Y. B, (2014).

The school leaving certificate (SLC) examination of Nepal: Exploring negative consequences. [Master dissertation].

Carleton University. https://carleton.scholaris.ca/server/api/core/bitstreams/070a3183-e3ab-4a47-9b6e-19e6a2b28cf1/content

11. Setopati. (2019, July 8). *Seeing through SEE results*. Setopati. https://en.setopati.com/view/149213

12. Chapagain, Y. (2021). School student academic performance in Nepal: An analysis using the School Education Exam (SEE) results. *International Journal on Studies in Education, 3*(1), 22-36. https://doi.org/10.46328/ijonse.34

~~11. Dixit, S. (2019). Seeing through SLC results.~~

~~Setopati. https://en.setopati.com/view/149213~~

Formatted: Font: (Default) Times New Roman, 12 pt
Formatted: Normal, Indent: Left: 0"
Formatted: Font: Italic
Formatted: Line spacing: 1.5 lines
Formatted: Font: Italic
Formatted: Font: Italic
Formatted: Font: (Default) Times New Roman, 12 pt
Formatted: Normal, No bullets or numbering
Formatted: Font: (Default) Times New Roman, 12 pt, Italic
Formatted: Normal, Indent: Left: 0"
Formatted: Font: (Default) Times New Roman, 12 pt
Formatted: Normal, No bullets or numbering, Tab stops: Not at 0.06"
Formatted: Font: (Default) Times New Roman, 12 pt
Formatted: Normal, Line spacing: 1.5 lines
Formatted: Font: (Default) Times New Roman, 12 pt, Italic
Formatted: Font: (Default) Times New Roman, 12 pt
Formatted: Font: (Default) Times New Roman, 12 pt
Formatted: Numbered + Level: 1 + Numbering Style: 1, 2, 3, … + Start at: 1 + Alignment: Left + Aligned at: 0.01" + Indent at: 0.26"
Formatted: Line spacing: 1.5 lines
Formatted: Normal, Line spacing: 1.5 lines, No bullets or numbering
Formatted: Font: (Default) Times New Roman, 12 pt
Formatted: Font: Italic
Formatted: Font: (Default) Times New Roman, 12 pt
Formatted: Normal (Web), Indent: Left: 0"
Formatted: Font: Italic
Formatted: Normal (Web), No bullets or numbering
Formatted: Font: Not Italic, Italian (Italy)
Formatted: Normal (Web), Indent: Left: 0"
Formatted: Italian (Italy)
Formatted: Font: Not Italic, Italian (Italy)

12. Chapagain, Y. (2021).

School student academic performance in Nepal: An analysis using the School Education Exam (SEE) results.

International Journal on Studies in Education, 3(1), 22-36.

13. 13. Adhikari, P. (2020, August 8).
Abuse of rights in evaluation.

Gorkhapatra Daily.
https://old.gorkhapatraonline.com/epaper/view?paper=gorkhapatra&today=2020-08-26.

14. Sherma, A.B. (2023, August 13).
Did the SEE result genuinely improve this year? *Republica.*
Republica
14. https://myrepublica.nagariknetwork.com/news/did-the-see-result-genuinely-improve-this-year
15. Dhakal, M. (2024, June 28).
-SEE results out: Over 52% students non- graded. *The Raisin Nepal.*
The Rising Nepal.
15. https://risingnepaldaily.com/news/44994
16. Budhathoki, C. B., Khatri, H.B., Shrestha, D. Rana, B. K., Sigdel, T.P., Panta, K., & Thapa, S.W. (2014).
16. *Status of SLC dropouts and identifying ways to engaging students in co-curricular activities: Final report*. Vertex Consult Pvt. Ltd.
https://doe.gov.np/assets/uploads/files/0f853b187d239aa7a083672837d7636a.pdf

17. (Ed. David Hayes).
British Council.

17. Danai, R.P. (2021). Head teachers' instructional leadership at community schools.

Formatted: Italian (Italy)

Formatted: Normal (Web), No bullets or numbering

Formatted: Normal (Web), Indent: Left: 0"

Formatted: Font: Italic

Formatted: Normal, Indent: Left: 0.06", No bullets or numbering

Formatted: Font: (Default) Times New Roman, 12 pt

Formatted: Normal, Indent: Left: 0", Tab stops: Not at 0.06"

Formatted: Font: (Default) Times New Roman, 12 pt

Formatted: Numbered + Level: 1 + Numbering Style: 1, 2, 3, … + Start at: 1 + Alignment: Left + Aligned at: 0.01" + Indent at: 0.26"

Formatted: Font: (Default) Times New Roman, 12 pt

Formatted: Space After: 10 pt, Numbered + Level: 1 + Numbering Style: 1, 2, 3, … + Start at: 1 + Alignment: Left + Aligned at: 0.01" + Indent at: 0.26", Tab stops: Not at 0.06"

Formatted: Font: Italic

Formatted: Font: (Default) Times New Roman, 12 pt

Formatted: Numbered + Level: 1 + Numbering Style: 1, 2, 3, … + Start at: 1 + Alignment: Left + Aligned at: 0.01" + Indent at: 0.26"

Formatted: Font: Italic

Formatted: Font: Italic

Formatted: Line spacing: 1.5 lines, Numbered + Level: 1 + Numbering Style: 1, 2, 3, … + Start at: 1 + Alignment: Left + Aligned at: 0.01" + Indent at: 0.26"

Formatted: Font: Italic

Formatted: Normal, Indent: Left: 0.01"

Formatted: Font: (Default) Times New Roman, 12 pt

Formatted: Normal, Indent: Left: 0.01", No bullets or numbering

AMC Journal, *2*(1), 55-73. https://doi.org/10.3126/amcj.v2i1.35788

18.

~~18.~~ Koirala, A. (2015). Debate on public and private schools in Nepal. *International Journal of S*

~~*International Journal of S*~~*ocial Sciences and Management*, *2*(1), 3-8. https://doi.org/10.3126/ijssm.v2i1.11882

19.

~~19.~~ Pandey, A. (2015, June 18). Poor SLC results.

The Himalaya Times. https://thehimalayantimes.com/opinion/poor-slc-results/~~https://thehimalayatimes.com/opinion/poor-slc-results/~~

~~20.~~ 20. Parajuli, D. R. & Das, T. (2013).

Performance of community schools in Nepal: A macro level analysis.

International Journal of Science and Technology Research, *2*(7), 148-154. https://www.ijstr.org/final-print/july2013/Performance-Of-Community-Schools-In-Nepal-A-Macro-Level-Analysis.pdf

21.

~~21.~~ Regmi, K.D. (2017).

World Bank in Nepal's education: Three decades of neoliberal reform.

Globalization, Societies and Education, *15*(2), 188-201. https://doi.org/10.1080/14767724.2016.1169517

~~22.~~ 22. Thapa, A. (2015).

Public and private school performance in Nepal: An analysis using the SLC examination.

Education Economics, *23*(1), 47-62. https://doi.org/10.1080/09645292.2012.738809

~~23.~~ 23. Hallinger, P. (2003).

Formatted: Line spacing: 1.5 lines
Formatted: Normal, Indent: Left: 0.01"
Formatted: Font: (Default) Times New Roman, 12 pt
Formatted: Normal, No bullets or numbering
Formatted: Font: Italic
Formatted: Font: (Default) Times New Roman, 12 pt, Italic
Formatted: Font: (Default) Times New Roman, 12 pt
Formatted: Normal, Line spacing: 1.5 lines
Formatted: Font: (Default) Times New Roman, 12 pt
Formatted: Numbered + Level: 1 + Numbering Style: 1, 2, 3, … + Start at: 1 + Alignment: Left + Aligned at: 0.01" + Indent at: 0.26"
Formatted: Normal (Web), Indent: Left: 0"
Formatted: Normal, Indent: Left: -0.25", Numbered + Level: 1 + Numbering Style: 1, 2, 3, … + Start at: 1 + Alignment: Left + Aligned at: 0.01" + Indent at: 0.26"
Formatted: Font: (Default) Times New Roman, 12 pt
Formatted: Normal, Indent: Left: 0"
Formatted: Font: (Default) Times New Roman, 12 pt
Formatted: Normal, Line spacing: 1.5 lines
Formatted: Normal, Indent: Left: 0"
Formatted: Font: (Default) Times New Roman, 12 pt
Formatted: Normal, No bullets or numbering
Formatted: Font: (Default) Times New Roman, 12 pt
Formatted: Normal, Indent: Left: 0"
Formatted: Font: (Default) Times New Roman, 12 pt
Formatted: Space Before: 0 pt, After: 0 pt, Line spacing: 1.5 lines
Formatted: Normal, No bullets or numbering
Formatted: Font: (Default) Times New Roman, 12 pt
Formatted: Normal, Indent: Left: 0"
Formatted: Font: (Default) Times New Roman, 12 pt
Formatted: Normal, Line spacing: 1.5 lines
Formatted: Normal, No bullets or numbering
Formatted: Font: (Default) Times New Roman, 12 pt

Leading educational change: Reflections on the practice of instructional and transformational leadership.

Cambridge Journal of Education, *33*(3), 329- 351. https://doi.org/10.1080/0305764032000122005

24. 24.Uchtman, P. A. (1992).

Beyond the myths of instructional leadership: A study of teacher attitudes regarding the implementation of an organizational model of instructional leadership [Doctoral dissertation].

University of Kansas. Day, C., Sammons, P., & Gorgen, K. (2020). Successful school leadership (ERIC Document No. ED614324). Education Development Trust. https://files.eric.ed.gov/fulltext/ED614324.pdf

25. 25. Irwin, C. C. (1985).

What research tells the teacher about instructional leadership . (ED257173). ERIC. https://eric.ed.gov/?id=ED257173

26. 26. Smith, F., & Andrews, R. L. (1989).

Instructional leadership: How principals make a difference (ED314826). ERIC. . [Report No. EA 021500], Association for Supervision and Curriculum Development, Alexandria, VA. https://eric.ed.gov/?id=ED314826

27. .

27. Glickman, C. D., Gordon, S. P., & Ross-Gordon, J. M. (1995). *Supervision of instruction: A developmental approach* (3rd ed.). Allyn & Bacon.

28. 28. Supovitz, J. A. (2000).

Manages less, leads more (EJ616311). ERIC. .

Principal Leadership, *1*(3), 14-19. https://eric.ed.gov/?id=EJ616311

Formatted: Normal, Indent: Left: 0"

Formatted: Font: (Default) Times New Roman, 12 pt

Formatted: Normal, Line spacing: 1.5 lines

Formatted: Normal, No bullets or numbering

Formatted: Font: (Default) Times New Roman, 12 pt

Formatted: Font: (Default) Times New Roman, 12 pt, Italic

Formatted: Normal, Indent: Left: 0"

Formatted: Font: (Default) Times New Roman, 12 pt

Formatted: Line spacing: 1.5 lines

Formatted: Font: (Default) Times New Roman, 12 pt

Formatted: Normal, Indent: Left: 0"

Formatted: Normal, Indent: Left: 0.01", No bullets or numbering

Formatted: Font: (Default) Times New Roman, 12 pt

Formatted: Normal, Indent: Left: 0"

Formatted: Font: (Default) Times New Roman, 12 pt

Formatted: Normal, No bullets or numbering

Formatted: Font: (Default) Times New Roman, 12 pt

Formatted: Normal, Line spacing: 1.5 lines

Formatted: Normal, Indent: Left: 0"

Formatted: Font: (Default) Times New Roman, 12 pt

Formatted: Normal, Right: 0", No bullets or numbering, Tab stops: 2.27", Left

Formatted: Font: (Default) Times New Roman, 12 pt, Italic

Formatted: Font: (Default) Times New Roman, 12 pt

Formatted: Normal, Indent: Left: -0.25", Numbered + Level: 1 + Numbering Style: 1, 2, 3, … + Start at: 23 + Alignment: Left + Aligned at: 0.01" + Indent at: 0.26"

Formatted: Font: (Default) Times New Roman, 12 pt

Formatted: Normal, Indent: Left: 0"

Formatted: Font: Italic

Formatted: Line spacing: 1.5 lines

Formatted: Font: (Default) Times New Roman, 12 pt

Formatted: Normal, Indent: Left: 0"

29. 29. Elmore, R. (2000). Elmore, R. F. (2000). Building a new structure for school leadership (ERIC Doc. No. ED546618). Albert Shanker Institute. https://files.eric.ed.gov/fulltext/ED546618.pdf

30. *Building a structure for school leadership.*

Albert Shanker Institute.

30. Bush, T. (2007).

Educational leadership and management: Theory, policy, and practice.

South African Journal of Education, 27(3), 391-406. https://files.eric.ed.gov/fulltext/EJ1150205.pdf

31.

31. Bush, T., & Glover, D. (2009).

Managing, teaching and learning (Concept paper).

Matthew Goniwe School of Leadership and Governance, South Africa. Bush, T., & Glover, D. (2009). Managing teaching and learning: A concept paper. Johannesburg, South Africa: Matthew Goniwe School of Leadership and Governance (MGSLG).

32. 32. Day, C., Gu, Q., & Sammons, P. (2016).

The impact of leadership on student outcomes: How successful school leaders use transformational and instructional strategies to make a difference.

Educational Administration, 52(2), 221– 258. https://journals.sagepub.com/doi/10.1177/0013161X15616863

33. 33. Hallinger, P., & Heck, R. H. (1998).

Exploring the principal's contribution to school effectiveness: 1980-1995.

Formatted: Normal (Web), No bullets or numbering

Formatted: Font: Not Italic

Formatted: Font: (Default) Times New Roman, 12 pt, Italic

Formatted: Font: (Default) Times New Roman, 12 pt

Formatted: Normal, Indent: Left: 0.01"

Formatted: Normal, Indent: Left: 0"

Formatted: Normal, No bullets or numbering, Tab stops: Not at 1"

Formatted: Normal, Indent: Left: 0", Tab stops: Not at 1"

Formatted: Normal, Line spacing: 1.5 lines

Formatted: Font: (Default) Times New Roman, 12 pt

Formatted: Normal, Indent: Left: 0"

Formatted: Font: (Default) Times New Roman, 12 pt, Kern at 12 pt

Formatted: Normal (Web), No bullets or numbering, Tab stops: Not at 0.56" + 0.63" + 1.44" + 1.81" + 2" + 2.5" + 3.25"

Formatted: Line spacing: 1.5 lines

Formatted: Font: (Default) Times New Roman, 12 pt

Formatted: Normal, Indent: Left: 0", Tab stops: Not at 0.56" + 0.63" + 1.44" + 1.81" + 2" + 2.5" + 3.25"

Formatted: Normal, No bullets or numbering

Formatted: Font: (Default) Times New Roman, 12 pt

Formatted: Normal, Indent: Left: 0"

Formatted: Font: (Default) Times New Roman, 12 pt

Formatted: Normal, No bullets or numbering

Formatted: Font: (Default) Times New Roman, 12 pt

Formatted: Normal, Indent: Left: 0"

School Effectiveness & School Improvement, *9*(2), 157-191.
https://doi.org/10.1080/0924345980090203

34.

~~34. Ylimaki, R. M. (2007).~~

~~Instructional leadership in challenging US schools.~~

~~*International Studies in Educational Administration, 35*(3), 11-19.~~

Gaston, V. A. (2024). Instructional leadership of school heads and its impact on teachers' professional development. Instabright International Journal of Multidisciplinary Research, 5(1), 1-32. https://doi.org/10.52877/instabright.005.01.0202

~~35.~~ 35. Andrews, R., & Soder, R. (1987).

Principal instructional leadership and school achievement (EJ353780). ERIC.~~.~~

Educational Leadership, *44*(6), 9-11. https://eric.ed.gov/?id=EJ353780

36.

~~36.~~ Gulcan, M. (2012).

Research on instructional leadership competencies of school principals (EJ991116). ERIC.~~.~~

Education, *132*(3), 625-635. https://eric.ed.gov/?id=EJ991116

~~37.~~ 37. Hallinger, P., & Murphy J. (1985).

Assessing the instructional ~~leadership~~management behavior of principals.

Elementary School Journal, 86(2), 217-248. https://doi.org/10.1086/461445

38.

~~38.~~ Leithwood, K. (1994).

Leadership for school restructuring. *Educational Administration Quarterly*, *30*(4), 498-518.
https://doi.org/10.1177/0013161X94030004006

Formatted: Font: (Default) Times New Roman, 12 pt

Formatted: Normal, Line spacing: 1.5 lines

Formatted: Font: (Default) Times New Roman, 12 pt

Formatted: Normal, Indent: Left: 0"

Formatted: Normal, No bullets or numbering, Tab stops: Not at 0.81" + 0.88" + 1"

Formatted: Normal, Indent: Left: 0", Tab stops: Not at 0.81" + 0.88" + 1"

Formatted: Normal, Space Before: 0 pt, After: 0 pt, Line spacing: 1.5 lines, No bullets or numbering

Formatted: Normal, No bullets or numbering

Formatted: Font: (Default) Times New Roman, 12 pt

Formatted: Normal, Indent: Left: 0"

Formatted: Font: (Default) Times New Roman, 12 pt

Formatted: Font: (Default) Times New Roman, 12 pt

Formatted: Normal, Line spacing: 1.5 lines

Formatted: Font: (Default) Times New Roman, 12 pt, Not Italic

Formatted: Font: (Default) Times New Roman, 12 pt

Formatted: Normal, Indent: Left: 0"

Formatted: Font: (Default) Times New Roman, 12 pt

Formatted: Normal, Right: 0", No bullets or numbering

Formatted: Normal, Indent: Left: 0", Right: 0"

Formatted: Line spacing: 1.5 lines

Formatted: Font: Italic

Formatted: Font: (Default) Times New Roman, 12 pt

Formatted: Normal, Indent: Left: 0"

Formatted: Numbered + Level: 1 + Numbering Style: 1, 2, 3, … + Start at: 23 + Alignment: Left + Aligned at: 0.01" + Indent at: 0.26"

Formatted: Line spacing: 1.5 lines

Formatted: Font: (Default) Times New Roman, 12 pt

Formatted: Indent: Left: 0.26", No bullets or numbering

Formatted: Line spacing: 1.5 lines

39.

Educational Administration Quarterly, 30(4), 498-518.

39. McEwan, E. K. (1998). McEwan-Adkins, E. K. (2003). Seven steps to effective instructional leadership (2nd ed.). Corwin Press.

40. Seven steps to effective instructional leadership.

Corwin Press.

40. Robinson, V. M. J., Lloyd, C. A., & Rowe, K. J. (2008).

The impact of leadership on student outcomes: An analysis of the differential effects of leadership types.

Educational Administration Quarterly, *44*(5), 635–674. https://doi.org/10.1177/0013161X08321509

41.

41. Sheppard, B. (1996).

Exploring the transformational nature of instructional leadership. *Alberta Journal of Educational Research*, *42*(4), 325-344. https://www.researchgate.net/publication/234599554_Exploring_the_Transformational_Nature_of_Instructional_Leadership

Alberta Journal of Educational Research, 42(4), 325-344.

42. 42. Burch, P. (2007).

The professionalization of instructional leadership in the United States: Competing values and current tensions. *Journal of Education Policy*, *22*(2), 195-214. https://doi.org/10.1080/02680930601158943

Journal of Education Policy, 22(2), 195-214.

43. 43. Reitzug, U. C. (2008).

Formatted: Font: (Default) Times New Roman, 12 pt

Formatted: Normal (Web), No bullets or numbering

Formatted: Font: Not Italic

Formatted: Normal, Indent: Left: 0"

Formatted: Font: (Default) Times New Roman, 12 pt

Formatted: Normal, No bullets or numbering

Formatted: Font: (Default) Times New Roman, 12 pt

Formatted: Normal, Indent: Left: 0"

Formatted: Font: (Default) Times New Roman, 12 pt

Formatted: Normal, Line spacing: 1.5 lines

Formatted: Font: (Default) Times New Roman, 12 pt

Formatted: Normal, Indent: Left: 0"

Formatted: Normal, No bullets or numbering

Formatted: Normal (Web), Indent: Left: 0"

Formatted: Font: (Default) Times New Roman, 12 pt

Formatted: Normal, No bullets or numbering

Formatted: Normal, Line spacing: 1.5 lines

Formatted: Font: (Default) Times New Roman, 12 pt

Formatted: Normal, Indent: Left: 0"

Formatted: Normal, Indent: Left: 0.01", No bullets or numbering

Formatted: Font: (Default) Times New Roman, 12 pt

Conceptualizing instructional leadership: The voices of principals (EJ806421). ERIC. *Education and Urban Society, 40*(6), 694-714.

44.

~~*Education and Urban Society, 40*(6), 694-714.~~

~~44.~~ Ovando, M., & Ramirez, A. (2007).

Principals' instructional leadership within a teacher performance appraisal system: Enhancing students' academic success. *Journal of Personnel Evaluation in Education, 20* (1-2), 85-110. https://doi.org/10.1007/s11092-007-9048-1

45.

~~*Journal of Personnel Evaluation in Education, 20* (1-2), 85-110.~~

~~45.~~ Hallinger, P. (2011).

A review of three decades of doctoral studies using the principal instructional management rating scale: A lens on methodological progress in educational leadership. *Educational Administration Quarterly, 47*(2), 271–306. https://doi.org/10.1177/0013161X10383412

46.

~~*Educational Administration Quarterly, 47*(2), 271–306.~~

Cresswell, J.W. (2012). *Educational research: Planning, conducting and evaluating quantitative and qualitative research* (4th ed.). Pearson.

47. ~~*Educational research: Planning, conducting and evaluating quantitative and qualitative research* (4th ed.).~~

~~Pearson.~~

~~46.~~ Perneger T.V., Courvoisier, D.S., Hudelson, P.M., & Ageron, A. G. (2014).

Sample size for pre-tests of questionnaire.

Quality of Life Research, 24(1),147-51. https://doi.org/10.1007/s11136-014-0752-2

Formatted: Font: (Default) Times New Roman, 12 pt

Formatted: Normal (Web), Indent: Left: 0"

Formatted: Font: Italic

Formatted: Normal (Web), No bullets or numbering, Pattern: Clear

Formatted: Line spacing: 1.5 lines

Formatted: Normal (Web), Indent: Left: 0", Pattern: Clear

Formatted: Font: (Default) Times New Roman, 12 pt

Formatted: Normal, No bullets or numbering, Pattern: Clear (White)

Formatted: Line spacing: 1.5 lines

Formatted: Font: Not Italic

Formatted: Normal, Indent: Left: 0", Pattern: Clear (White)

Formatted: Font: (Default) Times New Roman, 12 pt

Formatted: Font: (Default) Times New Roman, 12 pt, Italic

Formatted: Normal, Indent: Left: 0"

Formatted: Font: (Default) Times New Roman, 12 pt

Formatted: Normal, No bullets or numbering, Tab stops: Not at 0.06" + 0.13" + 0.19" + 0.44" + 0.5" + 0.81" + 1" + 1.25"

Formatted: Font: Not Italic

Formatted: Normal, Indent: Left: 0"

Formatted: Font: (Default) Times New Roman, 12 pt, Italic

Formatted: Font: (Default) Times New Roman, 12 pt

Formatted: Normal, No bullets or numbering, Tab stops: 0.06", Left + 0.13", Left + 0.19", Left + 0.44", Left + 0.5", Left + 0.81", Left + 1", Left + 1.25", Left

Formatted: Font: (Default) Times New Roman, 12 pt

Formatted: Font: (Default) Times New Roman, 12 pt, Italic

Formatted: Normal, Line spacing: 1.5 lines

Formatted: Font: (Default) Times New Roman, 12 pt

~~1 6.~~

48. Frost, J. (2021). Standard deviation: Definition, examples, and analysis. Statistics By Jim. https://statisticsbyjim.com/basics/standard-deviation.

49.

~~47. Kate, M. (2017).~~

~~What does a high standard deviation mean in real term?~~

~~*Socratic.*~~

~~https://socratic.org/questions/what-does-a-high-low-standard-deviation-mean-in-real-terms~~

~~48.~~ Zaman, N., Bibi, Z., Sheikh, S. R., & Raziq, A. (2020).

Manualizing factor analysis of likert scale.

Journal of Management Sciences 7(2), ~~:~~56-67. https://www.researchgate.net/publication/345746851_Manualizing_Factor_Analysis_of_Likert_Scale_Data

~~49.~~ 50. Tarka, P. (2015).
Likert scale and change in range of response categories. vs. the factors extraction in EFA model.
Acta Universitatis Lodziensis Folia Economica, ~~*1(*~~*311*(1), 27-37.
https://www.researchgate.net/publication/285639462_Likert_Scale_and_Change_in_Range_of_Response_Categories_vs_the_Factors_Extraction_in_EFA_Model

~~50.~~ 51. Cattell, R. B. (1978).
The scientific use of factor analysis in behavioral and life sciences~~.~~ (1st ed.).

Formatted: Font: (Default) Times New Roman, 12 pt, Italic
Formatted: Font: (Default) Times New Roman, 12 pt
Formatted: Normal, Indent: Left: 0"
Formatted: Normal, Indent: Left: 0.01", Line spacing: 1.5 lines, No bullets or numbering
Formatted: Font: (Default) Times New Roman, 12 pt
Formatted: Font: (Default) Times New Roman, 12 pt
Formatted: Font: (Default) Times New Roman, 12 pt
Formatted: Normal, Indent: Left: 0"
Formatted: Normal, No bullets or numbering, Adjust space between Latin and Asian text, Adjust space between Asian text and numbers
Formatted: Normal, Indent: Left: 0", Adjust space between Latin and Asian text, Adjust space between Asian text and numbers
Formatted: Italian (Italy)
Formatted: Normal, No bullets or numbering, Tab stops: Not at 0.81" + 0.88" + 1"
Formatted: Font: (Default) Times New Roman, 12 pt
Formatted: Normal, Indent: Left: 0", Tab stops: Not at 0.81" + 0.88" + 1"
Formatted: Line spacing: 1.5 lines
Formatted: Font: (Default) Times New Roman, 12 pt
Formatted: Normal, Indent: Left: 0", Tab stops: Not at 0.81" + 0.88" + 1"
Formatted: Normal, No bullets or numbering
Formatted: Font: (Default) Times New Roman, 12 pt
Formatted: Normal, Indent: Left: 0"
Formatted: Normal, Line spacing: 1.5 lines, Pattern: Clear (White)
Formatted: Font: (Default) Times New Roman, 12 pt, Italic
Formatted: Font: (Default) Times New Roman, 12 pt
Formatted: Indent: Left: -0.25", Numbered + Level: 1 + Numbering Style: 1, 2, 3, … + Start at: 23 + Alignment: Left + Aligned at: 0.01" + Indent at: 0.26"

Plenum.

~~51.~~ 52. Field, A. (2009).

Discovering statistics using SPSS (3rd ed.).

Sage.

~~52.~~ 53. Maskey, R., Fei, J., & Nguyen, H.O. (2018).

Use of exploratory factor analysis in maritime research. *The Asian Journal of Shipping and Logistics*, *34*(2), 91-111. https://doi.org/10.1016/j.ajsl.2018.06.006

54.

~~*The Asian Journal of Shipping and Logistics, 34*, 91-111.~~

~~53.~~ Tabachnick, B. G., & Fidell, L. S. (2007).

Using multivariate statistics (5th ed.).

Allyn & Bacon.

~~54.~~ 55. ~~Zulkepli, M., Sipan, I., & Jibril, J. D. (2017).~~

~~An exploratory factor analysis and reliability analysis for green affordable housing criteria instrument.~~

~~*An International Journal of Real Estate Studies, 11*(4), 9-21.~~Zulkepli, M. B., Sipan, I., & Jibril, J. D. (2017). *An exploratory factor analysis and reliability analysis for green affordable housing criteria instrument*. Semantic Scholar. https://api.semanticscholar.org/CorpusID:201895136

56.

~~55.~~ Kline, P. (1994).

An essay guide to factor analysis (1st ed.).

Routledge

57.

~~56.~~ Tavacol, M., & Dennic, R. (2011).

Formatted: Indent: Left: 0"

Formatted: Font: (Default) Times New Roman, 12 pt

Formatted: Normal, No bullets or numbering

Formatted: Font: (Default) Times New Roman, 12 pt, Italic

Formatted: Font: (Default) Times New Roman, 12 pt

Formatted: Normal, Indent: Left: 0"

Formatted: Normal, No bullets or numbering

Formatted: Line spacing: 1.5 lines

Formatted: Normal, Indent: Left: 0"

Formatted: Font: (Default) Times New Roman, 12 pt

Formatted: Normal, No bullets or numbering

Formatted: Font: (Default) Times New Roman, 12 pt, Italic

Formatted: Normal, Indent: Left: 0"

Formatted: Font: (Default) Times New Roman, 12 pt

Formatted: Font: (Default) Times New Roman, 12 pt

Formatted: Normal, No bullets or numbering

Formatted: Normal, Indent: Left: 0"

Formatted: Line spacing: 1.5 lines

Formatted: Font: Italic

Formatted: Normal, Indent: Left: 0"

Formatted: Normal, No bullets or numbering, Tab stops: Not at 0.81"

Formatted: Font: (Default) Times New Roman, 12 pt

Formatted: Normal, Indent: Left: 0", Tab stops: Not at 0.81"

Formatted: Normal, Indent: Left: 0"

Formatted: Normal, No bullets or numbering, Pattern: Clear

Making sense of cronbach's alpha.

International Journal of Medical Education 2, 53-55.
https://pmc.ncbi.nlm.nih.gov/articles/PMC4205511/

58.

~~57.~~ Alhosani, H.M.A. (2015).

Principals' instructional leadership practices and their relationship to teachers' instructional practices in Sarjah schools, [Master's dissertation].

United Arab Emirates University. https://scholarworks.uaeu.ac.ae/all_theses/22/

~~58.~~ 59. Lyons, B. J. (2010).

Principal instructional leadership behavior as perceived by teachers and principals at New York State recognized and non-recognized schools. [Doctoral dissertation].

Seton Hall University. https://scholarship.shu.edu/dissertations/1401/

60.

~~59.~~ Peariso, J. F. (2011).

A study of principals' instructional leadership behaviours and beliefs of good pedagogical practice among effective California high schools serving socioeconomically disadvantaged and English learners. [Doctoral dissertation]. Liberty University. https://eric.ed.gov/?id=ED529189

61.

~~Liberty University.~~

~~60.~~ Vilakaji, N.S. (2016).

Exploring principals' instructional leadership practices in primary schools: A case study of two underperforming township primary schools in Ekurhuleni South district, Gauteng province. [Master dissertation].

Formatted: Font: (Default) Times New Roman, 12 pt
Formatted: Line spacing: 1.5 lines
Formatted: Font: (Default) Times New Roman, 12 pt
Formatted: Normal, Indent: Left: 0"
Formatted: Normal, No bullets or numbering
Formatted: Font: (Default) Times New Roman, 12 pt, Italic, Kern at 12 pt
Formatted: Normal, Indent: Left: 0"
Formatted: Font: (Default) Times New Roman, 12 pt, Kern at 12 pt
Formatted: Font: (Default) Times New Roman, 12 pt
Formatted: Normal, Line spacing: 1.5 lines
Formatted: Font: (Default) Times New Roman, 12 pt
Formatted: Normal, No bullets or numbering
Formatted: Font: (Default) Times New Roman, 12 pt
Formatted: Font: (Default) Times New Roman, 12 pt, Italic
Formatted: Normal, Indent: Left: 0", Tab stops: Not at 0.06" + 0.13" + 0.19" + 1.32"
Formatted: Font: (Default) Times New Roman, 12 pt
Formatted: Space Before: 0 pt, Line spacing: 1.5 lines
Formatted: Normal, Indent: Left: 0", Tab stops: Not at 0.06" + 0.13" + 0.19" + 1.32"
Formatted: Font: (Default) Times New Roman, 12 pt
Formatted: Normal, No bullets or numbering, Tab stops: Not at 0.81"
Formatted: Font: (Default) Times New Roman, 12 pt, Italic
Formatted: Normal, Line spacing: 1.5 lines
Formatted: Font: (Default) Times New Roman, 12 pt
Formatted: Font: (Default) Times New Roman, 12 pt
Formatted: Normal, Indent: Left: 0"
Formatted: Font: (Default) Times New Roman, 12 pt
Formatted: Normal, Indent: Left: 0", Tab stops: Not at 0.81"
Formatted: Normal, No bullets or numbering, Tab stops: Not at 0.81" + 0.88" + 1"
Formatted ...
Formatted ...
Formatted ...

University of Witwatersrand, Johannesburg. https://wiredspace.wits.ac.za/server/api/core/bitstreams/1b835bc1-3192-4ac8-898a-c474f5bcc38c/content

61. 62. Krug, S.E. (1992).

Instructional leadership: A constructivist perspective.

Educational Administration Quarterly, *28*(3), 430-443. https://doi.org/10.1177/0013161X92028003012

63.

62. Hallainger, P. (2005).

Instructional leadership and the school principal: A passing fancy that refuses to fade away. *Leadership and Policy in Schools*, *4*(3), 1-20. http://doi.org/10.1080/15700760500244793

63. 64. Ghavifekr, S., Radwan, O., & Velarde J. M. (2019). Teachers' perceptions of principal's instructional leadership roles and practices. *Journal Pendidikan Malaysia 44*(2), 72- 83. http://dx.doi.org/ 10.17576/JPEN-2019-44.02-08.

65. *Journal Pendidikan Malaysia 44*(2), 72- 83. http://dx.doi.org/ 10.17576/JPEN-2019-44.02-08.

Jenssen, M. M. F., & Paulsen, J. M. (2022). Combining capacity for instructional leadership with individual core practices in the Norwegian policy context. *Educational Management Administration & Leadership*, *52*(2), 475-492. https://doi.org/10.1177/17411432221084155

66. He, P., Guo, F., & Abazie, G. A. (2024). School principals' instructional leadership as a predictor of teacher's professional development. *Asian-Pacific Journal of Second and Foreign Language Education*, *9*, Article 63. http://doi.org/10.1186/s40862-024-00290-0

64. 67. Dahie, M. A., Mohamed, A.A., & Mohamed, R. A. (2018).

Formatted: Line spacing: 1.5 lines
Formatted: Font: (Default) Times New Roman, 12 pt
Formatted: Font: (Default) Times New Roman, 12 pt
Formatted: Normal, No bullets or numbering
Formatted: Normal, Indent: Left: 0"
Formatted: Font: (Default) Times New Roman, 12 pt, Italic
Formatted: Font: (Default) Times New Roman, 12 pt
Formatted: Normal, Line spacing: 1.5 lines
Formatted: Normal, Indent: Left: 0"
Formatted: Font: (Default) Times New Roman, 12 pt
Formatted: Normal, No bullets or numbering
Formatted: Normal, Line spacing: 1.5 lines
Formatted: Font: (Default) Times New Roman, 12 pt, Not Italic
Formatted: Font: (Default) Times New Roman, 12 pt
Formatted: Font: (Default) Times New Roman, 12 pt
Formatted: Normal, Indent: Left: 0"
Formatted ...
Formatted: German (Germany)
Formatted: Font: (Default) Times New Roman, 12 pt
Formatted ...
Formatted: Normal, Indent: Left: 0.01"
Formatted: Font: Not Italic, Font color: Red
Formatted: Normal, Indent: Left: 0"
Formatted: Font color: Red
Formatted ...
Formatted: Font color: Red, German (Germany)
Formatted: Line spacing: 1.5 lines
Formatted: Font color: Red
Formatted: Font: Italic, Font color: Red
Formatted: Font color: Red
Formatted: Font color: Red
Formatted: Font: Italic, Font color: Red
Formatted: Font color: Red
Formatted: Font color: Red
Formatted: Normal, No bullets or numbering
Formatted: Font: (Default) Times New Roman, 12 pt

The role of parental involvement in student academic achievement: Empirical study from secondary schools in Mogadishu-Somalia.

International Research Journal of Human Resources and Social Sciences, 5(7), 1-24. https://www.researchgate.net/publication/326957190_THE_ROLE_OF_PARENTAL_INVOLVEMENT_IN_STUDENT_ACADEMIC_ACHIEVEMENT_EMPIRICAL_STUDY_FROM_SECONDARY_SCHOOLS_IN_MOGADISHU-SOMALIA

~~65.~~ 68. Sapungan, G. M., & Sapungam, R. M. (2014).

Parental involvement in child's education: Importnce, barriers and benefits. *Asian Journal of Management Sciences and Education 3*(2), 42-48. https://www.researchgate.net/publication/283539737_Parental_Involvement_in_Child's_Education_Importance_Barriers_and_Benefits

69.

~~*Asian Journal of Management Sciences and Education 3*(2), 42-48.~~

~~66.~~ Stronge, J. H., Richard, H. B., & Catano, N. (2008).

Qualities of effective principals (ED509122). ERIC. Association for Supervision and Curriculum Development. https://eric.ed.gov/?id=ED509122

70.

~~Association for Supervision and Curriculum Development.~~

~~67.~~ Northouse, P. G. (2015).

Introduction to leadership: Concepts and practice (3rd ed.). Sage.

71. Zhang, S., & Wu, H. (2025). School leadership, parental involvement, and student achievement: A comparative analysis of principal and teacher perspectives. *Education Sciences, 15*(6), Article 767. https://doi.org/10.3390/educsci15060767

72. Sebastian, J., & Allensworth, E. (2012). The influence of principal leadership on classroom instruction and student learning: A study of mediated pathways to learning. Educational Administration Quarterly, 48(4), 626–663. https://doi.org/10.1177/0013161X11436273

Formatted: Normal, Indent: Left: 0"

Formatted: Space Before: 0 pt, After: 0 pt, Line spacing: 1.5 lines, Tab stops: 0.06", Left + 0.13", Left + 0.19", Left + 0.44", Left + 0.5", Left + 0.81", Left + 1", Left + 1.25", Left

Formatted: Font: (Default) Times New Roman, 12 pt

Formatted: Normal, Indent: Left: 0"

Formatted: Normal, No bullets or numbering

Formatted: Italian (Italy)

Formatted: Line spacing: 1.5 lines

Formatted: Normal, Indent: Left: 0"

Formatted: Font: (Default) Times New Roman, 12 pt

Formatted: Font: (Default) Times New Roman, 12 pt

Formatted: Normal, No bullets or numbering, Don't adjust space between Latin and Asian text, Don't adjust space between Asian text and numbers, Tab stops: Not at 0.06" + 0.19" + 0.63" + 0.75" + 0.88"

Formatted: Font: (Default) Times New Roman, 12 pt, Italic

Formatted: Font: (Default) Times New Roman, 12 pt

Formatted: Font: Bold

Formatted: Font: (Default) Times New Roman, 12 pt

Formatted: Normal, Indent: Left: 0"

Formatted: Normal, No bullets or numbering, Adjust space between Latin and Asian text, Adjust space between Asian text and numbers, Tab stops: 0.06", Left + 0.19", Left + 0.75", Left + 0.88", Left + Not at 3"

Formatted: Font: (Default) Times New Roman, 12 pt, Italic

Formatted: Font: (Default) Times New Roman, 12 pt

Formatted: Line spacing: 1.5 lines

Formatted: Font color: Red

Formatted: Font color: Red

Formatted: Font color: Red

Formatted: Font color: Red

Formatted: Normal, Indent: Left: 0.01", No bullets or numbering

Formatted: Font: (Default) Times New Roman, 12 pt, Font color: Red

73. Le, L. K., Tran, T., Le, H. T. T., & Le, T. T. T. (2020). A study on factors affecting school principals' competencies in Vietnam's mountainous provinces. *Management in Education*, *35*(4), 174-181. https;//doi.org/10.1177/0892020620918254

74. Alanoglu, M. (2022). The role of instructional leadership in increasing teacher self-efficacy: A meta-analytic review. *Asia Pacific Education Review*, *23*, 233-244. https;//doi.org/10.1007/s12564-021-09726-5

75. Hallinger, P., Liu, S., & Aung, P. N. (2025). A systematic state-of-the-art review of Asian research on principal instructional leadership, 1987-2024. *Education Sciences*, *15*(7), Article 817. https;//doi.org/10.3390/educsci15070817

~~68.~~ 76. Utami, A.Y. (2022).
The role of parental involvement in student academic outcomes.
Journal of Education Review Provision, *2* (1), 17-21. https;//doi.org/10.55885/jerp.v2i1.156

~~69.~~ 77. Castro, M., Exposito-Casas, E., Lopez-Martin, E., Lizasoain, L., Navarro-Asencio, E., & Gaviria, J.L. (2015).
Parental involvement on student academic achievement: A meta-analysis.
Educational Research Review, 14, 33-46. https://doi.org/10.1016/j.edurev.2015.01.002

78. Gu, X., Hassan, N. C., & Sulaiman, T. (2024). The relationship between family factors and academic achievement of junior high school students in rural China: Mediation effect of parental involvement. Behavioral Sciences, 14(3), Article 221. https://doi.org/10.3390/bs14030221

79. Sujarwo, S., & Herwin, H. (2023). Parental involvement and student achievement: A meta-analysis of publications in the scopus database. *International Journal of Instruction*, *16*(2), 107-124. https://doi.org/10.29333/iji.2023.1627a

~~70.~~ 80. Epping, K.A. (2018).

Formatted: Font color: Red, Italian (Italy)
Formatted: Line spacing: 1.5 lines
Formatted: Font color: Red
Formatted: Font: Italic, Font color: Red
Formatted: Font color: Red
Formatted: Font color: Red
Formatted: Font color: Red, German (Germany)
Formatted: Font color: Red
Formatted: Font color: Red
Formatted: Normal, No bullets or numbering
Formatted: Font: (Default) Times New Roman, 12 pt, Kern at 12 pt
Formatted: Normal, Indent: Left: 0"
Formatted: Font: (Default) Times New Roman, 12 pt, Italic
Formatted: Normal, Line spacing: 1.5 lines, Tab stops: 0.81", Left + 0.88", Left + 1", Left
Formatted: Font: (Default) Times New Roman, 12 pt
Formatted: Font: (Default) Times New Roman, 12 pt, Kern at 12 pt
Formatted: Normal, Indent: Left: 0"
Formatted: No bullets or numbering
Formatted: Portuguese (Portugal)
Formatted: Indent: Left: 0"
Formatted: Space Before: 0 pt, Line spacing: 1.5 lines
Formatted: Indent: Left: 0"
Formatted: Normal (Web), Space Before: 0 pt, After: 0 pt, No bullets or numbering
Formatted: Font color: Red
Formatted: Font color: Red
Formatted: Line spacing: 1.5 lines
Formatted: Font color: Red
Formatted: Font color: Red
Formatted: Normal, No bullets or numbering
Formatted: Font: (Default) Times New Roman, 12 pt

The impact of parental involvement on student's academic achievement, parental well-being and parent-teacher relationships. [Master Dissertation]. University of Calgary, Alberta. https://ucalgary.scholaris.ca/server/api/core/bitstreams/b5387ad0-d588-4c55-b2aa-4db26388fb7b/content

81. University of Calgary.

71. Avnet, M., Makara, D., Larwin, K. H., & Erickson, M. (2019).

The impact of parental involvement and education on academic achievement in elementary school. *International Journal of Evaluation and Research in Education, 8*(3), 476-483. https://doi.org/10.11591/ijere.v8i3.20249

International Journal of Evaluation and Research in Education, 8 (3), 476-483.

Formatted: Font: Italic

Formatted: Normal (Web), Indent: Left: 0", Adjust space between Latin and Asian text, Adjust space between Asian text and numbers, Tab stops: Not at 0.13" + 1.85"

Formatted: Font: (Default) Times New Roman, 12 pt

Formatted: Normal, Indent: Left: 0"

Formatted: Normal, No bullets or numbering

Formatted: Kern at 12 pt

Formatted: Line spacing: 1.5 lines

Formatted: Font: (Default) Times New Roman, 12 pt, Kern at 12 pt

Formatted: Normal, Indent: Left: 0"

Dear Editor,

Thank you very much for your email and the constructive feedback provided on our manuscript. We truly appreciate the time and effort you and the reviewers have taken to review our work. The detailed comments were incredibly helpful in enhancing our understanding and improving the quality of the paper.

We have carefully addressed each of the suggestions and made substantial revisions to the manuscript. We believe that these revisions have significantly strengthened the paper.

In accordance waith the journal's data availability criteria, I have attached the dataset along with the manuscript and other supporting files.

We kindly request that you consider the revised manuscript for publication in your esteemed journal. We look forward to your feedback and hope for a favourable outcome.

Thank you once again for your valuable input and consideration. The comment matrix is as follows.

Best regards

Authors

Comment Matrix				
**Comment by: Reviewer 1**				
**S.No.**	**Section where revision has been**	**The section presented before revision was advised**	**Revision suggested**	**The section after revision was made according to the reviewers' comments**

	indicated			
1.	Literature Review	Researchers attempted to examine school effectiveness or program improvement in the late 1970s. They first identified the effective schools and then analyzed what made them effective. They noticed that a skillful head teacher was a key factor in accounting for successful change or school improvement [23]. This effective school research base assigned the role of instructional leader to the head teachers to develop their skills because it was found that a head teacher would seem to be capable of fulfilling the head teacher's functions of managing the facility, controlling the operation, taking charge of community relations and overseeing the student progress only when he or she also becomes responsible for instructional leadership [24]. As a result, instructional leadership appeared as an essential construct for making schools effective, but it was still unclear what the actual instructional leadership roles are.<br>Instructional leadership is a term formed by combining "instruction" and "leadership." There have been multiple efforts to define instruction, but four	The manuscript is generally intelligible and written in standard English, though there are occasional grammatical errors and redundancies, particularly in the literature review and policy background sections	Researchers began examining school effectiveness and program improvement in the late 1970s. They first identified effective schools and then analyzed what made them successful, noting that a skillful head teacher was a key factor in driving school improvement [23]. It was also emphasized that instructional leadership plays a pivotal role in fostering school improvement, promoting better academic outcomes both directly, by supporting teachers, and indirectly, by improving conditions for teaching and learning. Successful school leadership involves improving conditions for teaching and learning, redesigning and enriching the curriculum, redesigning the rolesw and responsibilities, enhancing teacher quality, building relationships inside the school and community, enhancing teaching and learning and building relationship outside the school community [24].

		key dimensions of instruction remain consistent: setting learning objectives, choosing teaching methods, selecting reinforcement activities, and evaluating outcomes [25]. Researchers hold varying views on ledership as well. Some have defined leadership as how a leader leads; others have described it as the job done through people [25]. Leadership includes followers acting toward goals that represent the values of the leaders and the followers [26]. Such uncertainty surrounding the concept of leadership has made the combined construct of “instructional leadership” difficult to define and clarify. The early instructional leadership models tended to focus on the leadership portion of the construct, and they attempted to define it in terms of general leadership theory. Those models focused on personality/ leadership style, behavioral/ situational or contextual components of the head teacher [24].<br>In this manner, the combined construct of "instructional leadership" has been defined in various ways. Instructional leadership means providing the necessary resources, possessing knowledge and skill		Instructional leadership is a construct derived form the combination of the concepts of "instruction" and "leadership." There have been multiple efforts to define instruction, but four key dimensions of instruction remain consistent: setting learning objectives, choosing teaching methods, selecting reinforcement activities, and evaluating outcomes [25]. Researchers hold varying views on ledership as well. Some have defined leadership as how a leader leads; others have described it as the job done through people [25]. Leadership includes followers acting toward goals that represent the values of the leaders and the followers [26]. Such uncertainty surrounding the concept of leadership has made the combined construct of “instructional leadership” difficult to define and clarify. The early instructional leadership models tended to focus on the leadership portion of the construct, and they attempted to define it in terms of general leadership theory. Those models focused on personality/leadership style, behavioral/situational, or contextual components of the head teacher [24].

		in curriculum and instructional matters, being a skilled communicator and being a visionary who is out and around creating a visible presence [26]. Instructional leadership is direct assistance to teachers in their day-to-day activities, development of collaborative groups among staff, design and procurement of effective staff development, curriculum development, and the use of action research [27]. These definitions emphasized not only improving the instructional leadership role of the head teacher through the monitoring of instructional activities but also focused on the managerial task of the head teacher within it. Later scholars of instructional leadership began linking it to activities aimed at enhancing teaching and learning, leading to a slight shift in how instructional leadership was defined. Here are some examples. The instructional leadership begins when principals talk about being in the classroom, visit with students about their academic pursuits, and prioritise instruction [28]. Instructional leadership is important for effective practices in curriculum, instruction, and assessment and the ability to work with teachers on the day-to-		In this manner, the combined construct of "instructional leadership" has been defined in various ways. Instructional leadership means providing the necessary resources, possessing knowledge and skill in curriculum and instructional matters, being a skilled communicator, and being a visionary who is out and around creating a visible presence [26]. Instructional leadership is direct assistance to teachers in their day-to-day activities, the development of collaborative groups among staff, the design and procurement of effective staff development, curriculum development, and the use of action research [27]. These definitions emphasized not only improving the instructional leadership role of the head teacher through the monitoring of instructional activities but also focused on the managerial tasks of the head teacher within it. Later scholars of instructional leadership began linking it to activities aimed at enhancing teaching and learning, leading to a slight shift in how instructional leadership was defined. Here are some examples: instructional leadership begins when principals talk about being in the classroom, visit with students

		day problems related to these topics [29]. The latter two perspectives on instructional leadership place a strong emphasis on teaching and learning activities. In this way the concept of instructional leadership evolved through various research developments, ultimately establishing that leadership roles connected to teaching and learning within the school fall under the domain of instructional leadership [30, 31, 2, 32].<br>The instructional leadership notion has dominated the educational literature since the creation of instructional leadership models, and there is ample proof of its effectiveness regarding teacher and student results [33, 34]. The notable models of instructional leadership have been proposed by various researchers [e.g., 35, 36, 37, 38, 39, 40, 41]. They have offered a variety of indications of instructional leadership, but the theoretical underpinnings are often the same [40, 41]. Researchers most extensively use the Hallinger and Murphy (1985) model of instructional leadership to do research on instructional leadership. The widespread acceptance of this model is primarily due		about their academic pursuits, and prioritize instruction [28]. Instructional leadership is important for effective practices in curriculum, instruction, and assessment, and for the ability to work with teachers on the day-to-day problems related to these topics [29]. The latter two perspectives on instructional leadership place a strong emphasis on teaching and learning activities. In this way, the concept of instructional leadership evolved through various research developments, ultimately establishing that leadership roles connected to teaching and learning within the school fall under the domain of instructional leadership [30, 31, 2, 32].<br>The instructional leadership notion has dominated the educational literature since the creation of instructional leadership models, and there is ample proof of its effectiveness regarding teacher and student results [33, 34]. The notable models of instructional leadership have been proposed by various researchers [e.g.,35, 36, 37, 38, 39, 40, 41]. They have offered a variety of indications of instructional leadership, but the theoretical underpinnings are often the same [40, 41].

		to Hallinger and Murphy's (1985) core concept, which provides head teachers with valuable insights into all aspects of leadership related to teaching and learning in schools. The main theme of this model is to develop and communicate school goals, coordinate and supervise the school curriculum, monitor and evaluate student progress, encourage teachers and students, and maintain high visibility and a hands-on approach [23, 44]. Their model is in the form of a scale known as PIMRS (Principal/ Head Teacher Instructional Management Rating Scale). This PIMRS has been utilized in at least 130 PhD dissertations over almost three decades [45].<br>Several academics have mentioned their view on the characteristics of an instructional leader. The instructional leaders perform at high levels in four areas: a) resource provider, b) instructional resource, c) communicator, and d) visible presence in the school [35]. The instructional leaders emphasize on: a) goal establishment curriculum development, b) frequent observations, c) program evaluation, and d) support for teachers [41]. The instructional leader perform seven crucial functions,which are: a)		Researchers most extensively use the Hallinger and Murphy (1985) model of instructional leadership to conduct research on instructional leadership. The widespread acceptance of this model is primarily due to Hallinger and Murphy's (1985) core concept, which provides head teachers with valuable insights into all aspects of leadership related to teaching and learning in schools. The main theme of this model is to develop and communicate school goals, coordinate and supervise the school curriculum, monitor and evaluate student progress, encourage teachers and students, and maintain high visibility and a hands-on approach [23, 44]. Their model is in the form of a scale known as PIMRS (Principal/Head Teacher Instructional Management Rating Scale). This PIMRS has been utilized in at least 130 PhD dissertations over almost three decades [45].<br>Several academics have mentioned their views on the characteristics of an instructional leader. I Instructional leaders perform at high levels in four areas: a) resource provider, b) instructional resource, c) communicator, and d) visible presence in the school [35]. The instructional leaders emphasize: a)

		establishing and implementing instructional goals; b) being available to staff; c) creating a school culture and climate conducive to learning; d) communicating the vision and mission of the school to students, staff, and parents; e) setting high expectations for the staff; f) developing teacher leaders; g) maintaining positive attitudes toward student, staff, and parents [39]. There are four tenets of an instructional leader: a) focus on developing, directing, and supervising the curriculum and instruction in the classroom; b) interact with their teachers to improve teaching and learning c) goal-oriented; d) establish high expectations and standards for teachers and students [38]. The effective instructional leader plays five important roles, which are: a) establishing goals and expectations; b) strategic resourcing; c) planning, coordinating, and evaluating teaching and the curriculum; d) promoting and participating in teacher learning and development; and e) ensuring an orderly and supportive environment [40]. There are six characteristics of effective instructional leaders: a) identify and communicate the mission and vision of the school; b) maintain a school environment that is		goal establishment and curriculum development, b) frequent observations, c) program evaluation, and d) support for teachers [41]. Instructional leaders perform seven crucial functions,which are: a) establishing and implementing instructional goals; b) being available to staff; c) creating a school culture and climate conducive to learning; d) communicating the vision and mission of the school to students, staff, and parents; e) setting high expectations for the staff; f) developing teacher leaders; g) maintaining positive attitudes toward student, staff, and parents [39]. There are four tenets of an instructional leader: a) focus on developing, directing, and supervising the curriculum and instruction in the classroom; b) interacting with teachers to improve teaching and learning c) being goal-oriented; d) establish high expectations and standards for teachers and students [38]. The effective instructional leader plays five important roles, which are: a) establishing goals and expectations; b) strategic resourcing; c) planning, coordinating, and evaluating teaching and the curriculum; d) promoting and participating in teacher learning and development; and e) ensuring an orderly

		conducive to learning; c) promote the professional development of staff; d) monitor teachers’ instruction; e) maintain a positive school climate and f) initiate organizational change [36]. From these scholars' view it can be inferred that an instructional leader's job is improving instruction to increase student achievement. Hence, an instructional leader is a person who seeks to ensure effective teaching and learning in the school by meeting all the requirements necessary to enhance students' academic performance.<br>Among the models of instructional leadership proposed by various researchers devoted to the study of instructional leadership, Hallinger and Murphy's (1985) model of instructional leadership was used as a theoretical guide for this research because, this model has attained top priority and is more dominated in the literature of instructional leadership. This model of instructional leadership consists of three factors: defining the school's mission, managing the instructional program, and promoting a positive learning climate. There are two functions under the factor defining the school's		and supportive environment [40]. There are six characteristics of effective instructional leaders: a) identifying and communicating the mission and vision of the school; b) maintain a school environment that is conducive to learning; c) promote the professional development of staff; d) monitor teachers’ instruction; e) maintain a positive school climate and f) initiate organizational change [36]. From these scholars' views, it can be inferred that an instructional leader's job is improving instruction to increase student achievement. Hence, an instructional leader is a person who seeks to ensure effective teaching and learning in the school by meeting all the requirements necessary to enhance students' academic performance.<br>Among the models of instructional leadership proposed by various researchers devoted to the study of instructional leadership, Hallinger and Murphy's (1985) model of instructional leadership was used as a theoretical guide for this research because this model has attained top priority and is more dominat in the literature on instructional leadership. This model of instructional leadership consists of three

		mission, which are framing the school goals and communicating the school goals, and three functions under the factor managing the instructional program, which are supervising and evaluating instruction, coordinating curriculum, and monitoring students' progress, and five functions under the factor promoting a positive learning climate which are protecting instructional time; providing incentives for teachers; providing incentives for learning; promoting professional development of teachers; and maintaining high visibility. There are five items under each of these ten functions, forming fifty variables altogether.<br>These fifty items were studied thoroughly, and classical Delphi was conducted, followed by three round meetings with experienced head teachers and teachers to get their concrete ideas on it. Based on their opinion, some items, for example, (i) draw upon the results of class wise testing when making curriculum decisions, (ii) participate actively in the review of curricular materials, (iii) acknowledge teachers' exceptional performance by writing memos for their personal files, (iv) recognize superior		factors: defining the school's mission, managing the instructional program, and promoting a positive learning climate. There are two functions under the factor defining the school's mission, which are framing the school goals and communicating the school goals; three functions under the factor managing the instructional program, which are supervising and evaluating instruction, coordinating curriculum, and monitoring students' progress; and five functions under the factor promoting a positive learning climate which are protecting instructional time, providing incentives for teachers, providing incentives for learning, promoting professional development of teachers, and maintaining high visibility. There are five items under each of these ten functions, forming fifty variables altogether.<br>These fifty items were studied thoroughly, and a classical Delphi was conducted, followed by three rounds of meetings with experienced head teachers and teachers to get their concrete ideas on it. Based on their opinions, some items- for example, (i) draw upon the results of class-wise testing when making curriculum decisions, (ii) participate actively

		students’ achievement or improvement by seeing in the office the students with their work, (v) review student work products when evaluating classroom instruction were unselected from this PIMRS. Some items, for example, (i) head teacher announces mandatory presence of parents during terminal report card distribution, (ii) head teacher meets the parents of poor-performing students and shares with them what roles the school and parents may together take for their improvements, (iii) head teacher learns students’ family problems and talks to their parents to resolve them, (iv) head teacher is transparent about every financial detail of the school to all stakeholders were added in new PIMRS.<br>Some items were changed to best suit in Nepali schools, such as (i) monitoring the classroom curriculum to see that it covers the school's curricular objectives was changed to the head teacher checking teachers' log books regularly to see if they are going in accordance with the syllabus, (ii) limit interruption of instructional time by public address announcements was changed to head teacher tries his best to ensure uninterrupted instruction time, (iv)		in the review of curricular materials, (iii) acknowledge teachers’ exceptional performance by writing memos for their personal files, (iv) recognize superior students’ achievement or improvement by seeing in the office the students with their work, (v) review student work products when evaluating classroom instruction- were deselected from this PIMRS. Some items- for example, (i) head teacher announces mandatory presence of parents during terminal report card distribution, (ii) head teacher meets the parents of poor-performing students and shares with them what roles the school and parents may together take for their improvements, (iii) head teacher learns students’ family problems and talks to their parents to resolve them, (iv) the head teacher is transparent about every financial detail of the school to all stakeholders- were added to the in new PIMRS.<br>Some items were changed to best suit Nepali schools, such as (i) monitoring the classroom curriculum to see that it covers the school's curricular objectives was changed to the head teacher checking teachers' log books regularly to see if they are going in accordance with the syllabus, (ii) limiting

		lead or attend teacher in- service activities concerned with instruction was changed to head teacher seeks to provide training to teachers during vacations or so as not to interrupt daily classes, (v) conduct informal observations in classrooms regularly was changed to head teacher regularly monitors activities of teachers and students while they are in class. In this way, finally a new PIMRS was developed for this research. This PIMRS consists of 11 factors and 33 items.		interruption of instructional time by public address announcements was changed to head teacher trying his best to ensure uninterrupted instructional time, (iii) leading or attending teacher in- service activities concerned with instruction was changed to head teacher seeking to provide training to teachers during vacations so as not to interrupt daily classes, (v) conducting informal observations in classrooms regularly was changed to head teacher regularly monitoring the activities of teachers and students while they are in class. In this way, a new PIMRS was finally developed for this research. This PIMRS consists of 11 factors and 33 items.
	**Comment by: Reviewer 1**			
2.	Result	The factor analysis yielded 25 items under three factors. Fourteen of these twenty-five variables fall under factor one, eight under factor two, and three under factor three. Factor One: Planning, managing and supervising instructional program All the items of factor one are shown below	The structure of the results section could be improved with clearer subheadings, and some sentences would benefit from	The factor analysis yielded three instructional leadership factors. Fourteen items loaded on the first factor, eight on the second, and three on the third. Factor One: Planning, managing and supervising instructional program All the items of factor one are shown below in the rotated component matrix of this factor.

		in the rotated component matrix of this factor.<br>**Table 1**<br>*Rotated Component Matrix of Factor One of IL*<br><br>These fourteen variables are connected to five distinct instructional leadership roles: planning and controlling school educational activities, overseeing and evaluating instruction, coordinating the curriculum and other educational objectives, keeping track of students' progress, and utilizing instructional time. These specific instructional leadership functions of the head teacher, except a last one, the head teacher directs teachers to make sure no student has skipped class, are similar to the items in Hallinger and Murphy's (1985) framework of IL falling under their two factors, defining the school mission, and managing instructional program [58-61]. Therefore, by including the meaning of those three leadership functions, namely defining the school mission, managing the instructional program, and ensuring no student has bunked the class, this factor was appropriately named as planning,	greater clarity and conciseness. Terms such as "post-positivist paradigm" should be defined for broader accessibility.	**Table 1**<br>*Rotated Component Matrix of Factor One of IL*<br><br>**Explanation of Variables in Factor One**<br>These fourteen variables are connected to five distinct instructional leadership roles: planning and controlling school educational activities, overseeing and evaluating instruction, coordinating the curriculum and other educational objectives, keeping track of students' progress, and utilizing instructional time. These specific instructional leadership functions of the head teacher, except the head teacher directs teachers to make sure no student has skipped class, are similar to the items in Hallinger and Murphy's (1985) framework of IL falling under their two factors, defining the school mission, and managing instructional program [58, 59, 60, 61]. Therefore, by including the meaning of those three leadership functions, namely defining the school mission, managing the instructional program, and

		managing, and supervising instructional program.<br><br>The factor planning, managing and supervising instructional program is about making an educational plan for the school, coordinating the curriculum, sharing plans, observing instructors to ensure they are practicing good teaching techniques, communicating with teachers to learn about their students' development, and making attempts to improve. There were two factors: framing and communicating school goals and supervising and evaluating instruction for these particular instructional leadership activities in Hallinger and Murphy's (1985) model [37]. They mentioned, it is about setting and expressing goals, working with staff members and teachers, and managing the instructional process and curriculum. It involves establishing the school's goals, reminding teachers of the purposes and mission, and informing teachers that teachers need to plan their classes properly [62]. A school lacks any standards to assess its success if it has no plan for improving the educational process. This factor is concerned with choosing the primary		ensuring no student has bunked the class, this factor was appropriately named as planning, managing, and supervising the instructional program. All the variables of the factor planning, managing, and supervising instructional program show that it is about making an educational plan for the school, coordinating the curriculum, sharing plans, observing instructors to ensure they are practicing good teaching techniques, communicating with teachers to learn about their students' development, and making attempts to improve. There were two factors: framing and communicating school goals, and supervising and evaluating instruction for these particular instructional leadership activities in Hallinger and Murphy's (1985) model [37]. They mentioned, it is about setting and expressing goals, working with staff members and teachers, and managing the instructional process and curriculum. According to [62] this

		goals of the school as well as coordinating and managing curriculum and instruction [63]. Planning, managing, and supervising instructional program entails about articulating and expressing the school's goals, coordinating the curriculum, and monitoring and controlling the program by supervising and evaluating instruction [64].<br><br>Factor Two: Motivating and developing competence of teachers and students<br>All the items of factor two are shown below in the rotated component matrix of this factor.<br><br>**Table 2**<br>*Rotated Component Matrix of Factor Two of IL*<br><br>Factor two consists of eight variables which explain the role of an instructional leader in motivating and supporting teachers and students for their academic growth. These eight variables serve similar functions to the variables in the Hallinger and Murphy's (1985) Framework of instructional leadership placed under the functions providing incentives for teachers, providing incentives for		involves establishing the school's goals, reminding teachers of its purposes and mission, and informing teachers that teachers need to plan their classes properly. A school lacks any standards to assess its success without a plan to improve the educational process. As stated by [63] this factor relates to selecting the schools' primary goals as well as coordinating and managing curriculum and instruction. Similarly, [64] notes that planning, managing, and supervising instructional programs entails articulating and expressing the school's goals, coordinating the curriculum, and monitoring and controlling the program by supervising and evaluating instruction.<br><br>This factor also resonates with the conceptualization of instructional leadership by [65], which is grounded in head teachers' core practices and was developed to explain how Norwegian school leaders build their instructional capacity. Their multidimensional model consisting of goal

		learning, and promoting professional development under the factor creating a positive school climate [58-61]. Since the obtained eight variables of this factor share the same aim of inspiring teachers and students for their development, a suitable name, 'motivating and developing the competence of teachers and students' was given to this factor.<br>The factor motivating and developing the competence of teachers and students is about encouraging teachers and students to develop their abilities and academic excellence. Praising and rewarding teachers and students for their good deeds, assisting teachers with their professional growth, and supporting students in improving their self-confidence and academic focus are the key roles of an instructional leader, which are crucial in achieving academic success in the school. Recognizing and rewarding teachers' and students' accomplishments is important to develop interest in them [62]. These instructional leadership roles correspond to two functions in Hallinger and Murphy's (1985) instructional leadership model: promoting professional development, and offering incentives for		orientation, collaboration with teachers, professional development, observation, and supervision is similar to the variables of the factor planning, managing and supervising instructional program identified in this study. Both undersore head teachers' active role in shaping and monitoring instructional processes as a foundation for improving teaching and learning.<br>This finding can also be compared with those [66], where coordinating the curriculum and providing professional learning opportunities to teachers emerged as statistically significant predictors of teachers' professional development in the context of Nigeria. Coordinating the curriculum is parallel to the variable "the head teacher gets every subject department heads to implement the syllabus of all subjects in each class" within the factor of planning, managing and supervising instructional program. This reinforces the importance of this variable for developing teaching and learning ability. The next predictor, providing professional learning opportunities to teachers, corroborates the variables of the second factor, motivating and developing

		learning. This factor focuses on inspiring and fostering teachers' and students' competence [64]. The overall focus is on helping both teachers and students get better.<br><br>Factor Three: Involving Parents<br><br>All the items of factor three are shown below in the rotated component matrix of this factor.<br><br>**Table 3**<br><br>*Rotated Component Matrix of Factor Three of IL*<br><br>The factor three of instructional leadership consists of three items. These items are linked to the head teachers' policy to get parents/guardians involved in knowing about their children's condition and taking the appropriate action if necessary. In accordance with the meaning of these variables, a suitable name involving parents was given to this factor.<br><br>The core idea summarized in the variables of this factor is to enhance parent/guardian-school communication for students' academic development. The parents/guardians are also made accountable along with the school through this strategy. Parental role is found to have given more importance to		competence of teachers and students developed in this study.<br><br>Factor Two: Motivating and developing competence of teachers and students<br><br>All the items of factor two are shown below in the rotated component matrix of this factor.<br><br>**Table 2**<br><br>*Rotated Component Matrix of Factor Two of IL*<br><br>**Explanation of Variables in Factor Two**<br><br>Factor two consists of eight variables which explain the role of an instructional leader in motivating and supporting teachers and students for their academic growth. These eight variables serve similar functions to the variables in the Hallinger and Murphy's (1985) Framework of instructional leadership placed under the functions providing incentives for teachers, providing incentives for learning, and promoting professional development under the factor creating a positive school climate [58, 59, 60, 61]. Since the obtained eight variables of this factor share the same aim of inspiring teachers and students for their development, a suitable name,

		students' academic progress in the Nepali context. Although there are studies [65, 66] that have shown a positive relationship between parental involvement and students' academic achievement in the international context as well, but the parent's involvement is not mentioned as a factor in the researchers' instructional leadership models whoever have proposed such model.		'motivating and developing competence of teachers and students' was given to this factor.<br>All the variables of the factor motivating and developing the competence of teachers and students show that it is about encouraging teachers and students to develop their abilities and academic excellence. Praising and rewarding teachers and students for their good deeds, assisting teachers with their professional growth, and supporting students in improving their self-confidence and academic focus are the key roles of an instructional leader, which are crucial in achieving academic success in the school. As stated by [62], recognizing and rewarding teachers' and students' accomplishments is important for fostering their interest and engagement . These instructional leadership roles align withtwo functions in Hallinger and Murphy's (1985) instructional leadership model, promoting professional development, and providing incentives for learning. According to [64], this factor emphasizes on inspiring and enhancing the competence of both teachers and students . The overall focus is on helping both teachers and students get better.

				The variables within this factor, such as offering training to teachers during vacations, supporting the classroom application of acquired skills, and facilitating educational tours corroborate the finding of [66] who found that providing professional learning opportunities to teachers supports their professional development. In contrast to the finding of [66] who did not find providing incentives for teachers to be a significant predictor of teachers' professional development, the present study suggests that motivational strategies such as public praise, appreciation letters, honor certificates, and rewarding sincere teachers with opportunities for career development are integral to strengthening teachers' competence. Additionally, the inclusion of student focused practices such as honoring excellent performance and addressing family problems reflects a broader and more relational approach for both teachers' and learners' holistic development.<br><br>Factor Three: Involving Parents<br>All the items of factor three are shown below in the rotated component matrix of this factor.

				**Table 3**<br>*Rotated Component Matrix of Factor Three of IL*<br>**Explanation of Variables in Factor Three**<br>The factor three of instructional leadership consists of three items. These items are linked to the head teachers' policy to get parents/guardians involved in knowing about their children's condition and taking the appropriate action if necessary. In accordance with the meaning of these variables, a suitable name involving parents was given to this factor. The core idea summarized in the variables of this factor is to enhance parent/guardian-school communication for students' academic development. The parents/guardians are also made accountable along with the school through this strategy. Parental role is found to be more important to students' academic progress in the Nepali context. There are studies that have shown importance of the parent-school relationship. For example, some studies [67 , 68] have shown a positive relationship between parental involvement and students' academic achievement, [69] reported that the increasing parental and community involvement in the

				educational process results in stronger relationships between school leaders and the community. Similarly, [70] emphasized the need for developing relational behavior for school administrators, including interaction with parents. A recent research [71] conducted in Hong Kong, and Macao also demonstrated that leadership can sinfluence student achievement through Parental Academic Commitment (PAC). This reinforces the idea that parent involvement is not just a peripheral activity but a *leadership-mediated pathway* to better student outcomes.
	**Comment by: Reviewer 1**			

3.	Abstract & Research Methods	This research follows a post-positivist paradigm relying on statistical analysis of numerical data.	Terms such as "post-positivist paradigm" should be defined for broader accessibility. Overall, the manuscript communicates its findings effectively, but a thorough language and style revision is recommended to address minor issues and improve readability.	The objective reality of instructional leadership exists out there, and that reality can be found through careful study and measurement. Although the reality of instructional leadership exists independently, it can only be understood through data and evidence. Therefore, this study relies on statistical analysis of numerical data, a method known as quantitative research.<br><br>Language and style revision is also done
**Comment by: Reviewer 1**				
4.	Regarding data availability		Regarding data availability, the manuscript states that data are with the authors and can	Data will be submitted along with the manuscript and other required files

			be provided if necessary. However, this does not fully comply with PLOS ONE's data policy, which requires that all data underlying the findings be made publicly available without restriction. The authors should deposit the anonymized dataset in a public repository or provide it as supplementary information to ensure compliance with journal requirements.	
**Comment by: Reviewer 2**				

5.	Regarding output files of factor analysis		The authors should provide the outputs of the factor analysis, which was completed using the Statistical Package for Social Sciences 29.0. Based on the information provided regarding the factor loadings of the questionnaire items, reviewers need to verify the factor loadings outputs to ensure the data analysis's accuracy.	The output files of factor analysis will be attached
**Comment by: Reviewer 3**				
6.	Abstract	Instructional leadership is the leadership role associated with teaching and learning that academic heads must take on in order to demonstrate higher	The abstract and keywords do not provide adequate	Instructional leadership is centered on teaching and learning that academic leaders undertake to promote higher levels of student learning. Given

		levels of student accomplishment. This study explores the factors influencing instructional leadership in Kathmandu, Nepal. Hallinger and Murphy (1985) model of instructional leadership is taken as the fundamental basis of this study. This model is subjected to Classical Delphi to develop a scale for this study. Exploratory factor analysis is conducted in the data to extract the factors. Three factors are formed and named as planning, managing and supervising instructional program; motivating and developing competence of teachers and students; and involving parents. The factors identified are supported by existing research. This research follows a post-positivist paradigm relying on statistical analysis of numerical data. The findings are valuable for school head teachers, schools' other stakeholders, concerned government officials of educational sector, and international researchers in instructional leadership.<br>**Keywords**<br>Instructional leadership, School, Head teacher, Student, Academic achievement	information about the article. Although the objective and topic addressed in the manuscript are mentioned, the research methodology, main results, and conclusions found in the study are missing.	its importance, this study examines the factors influencing instructional leadership in Kathmandu, Nepal. The framework for the study is grounded in Hallinger and Murphy’s (1985) model of instructional leadership, which was employed as the conceptual basis. Using the Classical Delphi method, the model was adapted to develop a measurement scale, while the study was situated within a post-positivist paradigm, assuming that the reality of instructional leadership exists independently. Exploratory factor analysis was then conducted to extract the underlying factors. The findings reveal three major factors influencing instructional leadership: (a) planning, managing, and supervising instructional programs; (b) motivating and developing the competence of teachers and students; and (c) involving parents in the instructional process. The inclusion of parental involvement emerged as a novel dimension in the context of Nepal. These results

				provide meaningful insights for school headteachers, other educational stakeholders, policymakers in the government sector, and international researchers interested in instructional leadership.<br><br>**Keywords**<br>Instructional leadership, School, Head teacher, Student, Academic achievement, Factors
**Comment by: Reviewer 3**				
7.	Reference		Check whether all authors cited in the article are included in the manuscript's references, and vice versa.	Checked
**Comment by: Reviewer 3**				
8.	Introduction	Unsatisfactory school performance over the years is evident from the SLC (School Leaving Certificate) results before the Education Act 2016, and SEE (Secondary Education Examination) results after the	5. The article has issues with clarity and coherence in its language, which	Unsatisfactory school performance over the years is evident from the SLC (School Leaving Certificate) results before the Education Act 2016, and SEE (Secondary Education Examination) results

		Education Act 2016 [16, 12, 17, 11, 18-22]. The unsatisfactory performance of public schools in Nepal is a blatant indication of the head teachers' inadequate effort in the teaching and learning process in these schools. What could be the cause of this failure? To determine the cause of any occurrence, it is necessary to understand the factors influencing the outcome. In this regard, there seems an urgent need to know the factors affecting head teachers' role in teaching and learning, which is called the instructional leadership role. Although there is a large amount of literature on instructional leadership, a study to find the factors that affect the instructional leadership of head teachers in the local context of Nepal has not been found so far in the literature. Therefore, it seemed imperative to research the factors that influence the instructional leadership of head teachers. So, the research question developed for this study is, what factors affect head teachers' instructional leadership. Identifying the factors that influence instructional leadership would help head teachers identify where they are falling short and where they need to advance. This research is believed	need to be revised. The Introduction doesn't clarify the exact objective of the research (it differs from the objective stated in the Abstract) and the central research question. Please review this.	after the Education Act 2016 [16, 12, 17, 11, 18, 19, 20, 21,22]. The unsatisfactory performance of public schools in Nepal is a blatant indication of the head teachers' inadequate effort in the teaching and learning process in these schools, a responsibility that falls under their instructional leadership role.. In this regard, there is an urgent need to identify the influencing factors of head teachers' instructional leadership. Although there are a number of literature on instructional leadership, a study to find the factors that affect the instructional leadership of head teachers in the local context of Nepal has not been found so far in the literature. Therefore, it was deemed imperative to identify the factors affecting head teachers' instructional leadership. So, the research question developed for this study is, what are the factors that affect head teachers' instructional leadership. Identifying the factors affecting head teachers' instructional leaderahip would help head teachers recognize areas where they are falling short and where they need to improve. This research is expected to support head teachers developing their professional abilities as effective instructional

		to be particularly helpful in helping head teachers build their professional abilities as effective instructional leaders. This study can improve the students' achievement in the classroom and supplement to what is already known about instructional leadership.		leaders, which, in turn, can enhance students' achievement in schools. Furthermore, the finding of this study can contribute to the existing body of knowlwdge on instructional leadership.
**Comment by: Reviewer 3**				
9.	Methodology	As the study intends to find out the determining factors of instructional leadership, it is the study of a *cause*-and-effect relationship. It is because those factors can be found out by studying the causes that influence instructional leadership. Therefore, it follows the post-positivistic deterministic philosophy of *causes* determine *effects*. The objective reality of instructional leadership exists out there, and that reality can be found out through careful study and measurement. The research design is quantitative, and research methods include the preparation of questionnaires, data collection, data analysis, and interpretation. A well-structured questionnaire was prepared using the new PIMRS. The new PIMRS with 11 factors and 33 items asked the respondents to rate their view on a 5-point Likert	Regarding the methodology, it's necessary to clarify how the questionnaires were designed and administered to the research participants, and how the data analysis and interpretation were performed.	As the study intends to find out the determining factors of instructional leadership, it is the study of a *cause*-and-effect relationship. It is because those factors can be found out by studying the causes that influence instructional leadership. Therefore, it follows the post-positivistic deterministic philosophy of *causes* determine *effects*. The objective reality of instructional leadership exists out there, and that reality can be found out through careful study and measurement. The research design is quantitative, and research methods include the preparation of questionnaires, data collection, data analysis, and interpretation. Classical Delphi was conducted on Hallinger and Murphy's (1985) instructional leadership model, known as Principal/Head teacher Instructional Management

		scale with the descriptors; 1= almost never, 2= seldom, 3= sometimes, 4= frequently, and 5= almost always at which the head teacher was engaged in those particular activities of the school. The data was collected using a survey design, an appropriate way to get quantitative or numerical descriptions of population trends, attitudes, or opinions by surveying a sample of that population [46].<br>The population of this study includes all public secondary schools in Kathmandu Valley. Kathmandu Valley was chosen for the study because it has the greatest concentration of public secondary schools compared to any other region in the nation. In total, 303 public secondary schools in the Kathmandu Valley made up the population of this study and 172 schools made up the sample size. 172 schools were chosen at random from a pool of 303 to visit to gather data. Data was collected from one teacher and the head teacher each from 172 schools to see the difference in head teachers' and teachers' ratings. One teacher from each school was chosen by the lottery process.		Rating Scale (PIMRS) to develop a PIMRS suitable in the context of Nepal. A well-structured questionnaire was prepared using the new PIMRS. The questionnaire developed from new PIMRS with 11 factors and 33 items asked the respondents to rate their view on a 5-point Likert scale with the descriptors; 1= almost never, 2= seldom, 3= sometimes, 4= frequently, and 5= almost always at which the head teacher was engaged in those particular activities of the school. The data was collected using a survey design, an appropriate way to get quantitative or numerical descriptions of population trends, attitudes, or opinions by surveying a sample of that population [46]. Questionaire was administered to respondents during visits to their schools.<br>The population of this study includes all public secondary schools in Kathmandu Valley. Kathmandu Valley was chosen for the study because it has the greatest concentration of public secondary schools compared to any other region in the nation. In total, 303 public secondary schools in the Kathmandu Valley made up the population of this study and 172

				schools made up the sample size. 172 schools were chosen at random from a pool of 303 to visit to gather data. Data was collected from one teacher and the head teacher each from 172 schools to see the difference in head teachers' and teachers' ratings. One teacher from each school was chosen by the lottery process. Factor analysis was conducted on the obtained data to identify the factors affecting head teachers' instructional leadership. The findings were analysed and interpreted in the light of the relevant literature.
**Comment by: Reviewer 3**				
10.	Conclusion	The study concludes that there are three factors determining instructional leadership. The first factor, planning, managing and supervising instructional programs, explains the head teacher's role in collaborating with teachers to ensure that the school has a clear mission and that mission concentrates on students' academic achievement. The second factor, motivating and developing the competence of teachers and students describes the	The conclusions lack clarity on whether the central research question was answered, whether the hypotheses were confirmed, and the relevance of the	In response to the research question, what factors affect head teachers' instructional leadership, the study identified three factors. The first factor, planning, managing and supervising instructional programs, explains the head teacher's role in collaborating with teachers to ensure that the school has a clear mission and that mission concentrates on students' academic achievement. The second factor, motivating and developing competence of teachers

		head teacher's activities to boost teachers' and students' interest in their line of work. The third factor, involving parents mentions the roles of parents/ guardians in association with school for students' academic progress. The study's key finding is that the parents'/ guardians' involvement in students' progress is seen as an essential factor in the schools of Nepal which is different from Hallinger and Murphy's (1985) model of instructional leadership. In Hallinger and Murphy's (1985) instructional leadership model, the parents'/guardians' involvement is not explicitly recognized as a factor in student success. Instead, the model includes only one related item, contacting parents to communicate improved or exemplary student performance or contributions, which falls under the factor "providing incentives for learning". This suggests that Nepal's unique cultural and societal framework places greater emphasis on the role of parents/ guardians in students' academic achievement.	research to the field of study.	and students describes the head teacher's activities to boost teachers' and students' interest in their line of work. The third factor, involving parents mentions the roles of parents/ guardians in association with school for students' academic progress. The study's key finding is that the parents'/ guardians' involvement in students' progress is seen as an essential factor in the schools of Nepal which is different from Hallinger and Murphy's (1985) model of instructional leadership. In Hallinger and Murphy's (1985) instructional leadership model, the parents'/guardians' involvement is not explicitly recognized as a factor in student success. Instead, the model includes only one related item, contacting parents to communicate improved or exemplary student performance or contributions, which falls under the factor "providing incentives for learning". This suggests that Nepal's unique cultural and societal framework places greater emphasis on the role of parents/ guardians in students' academic achievement. By identifying these factors this study contributes to the field of instructional leadership and underscores specific requirements for effective

				teaching and learning in the public schools of Nepal.
**Comment by: Reviewer 3**				
11.	Literature Review		There is a connection between the theme and the theoretical foundation. However, I don't understand why the author included two literature review topics (Literature Review and Theoretical Foundation), when they are the same thing. It's important to review this so that the topic discussed in this theoretical section	The heading "Theoretical Foundation" is removed

			isn't redundant and doesn't confuse the reader when reading the research	
**Comment by: Reviewer 3**				
12.	Discussion		There are some issues with the data analysis and the coherence of the argument.	We have strengthened the coherence of our argument by adding relevant literature that aligns with and supports the results obtained in this study.<br><br>In the track-change mode manuscript, and in the review matrix the text in red indicates the changes or additions<br>.
**Comment by: Reviewer 3**				
13.	Result	The factor analysis yielded 25 items under three factors. Fourteen of these twenty-five variables fall under factor one, eight under factor two, and three under factor three.	The following statement is confusing: "Factor analysis resulted in	The factor analysis yielded three instructional leadership factors. Fourteen items loaded on the first factor, eight on the second, and three on the third.

			25 items under three factors. Fourteen of these twenty-five items fall under factor one, eight under factor two, and three under factor three." Please explain better what this means and what you mean by it. Make this information clear to the reader, as well as how you arrived at this conclusion.	We arrived to this conclusion from the result of factor analysis
**Comment by: Reviewer 3**				

14.			Furthermore, it is important to review all the variables obtained.	We have carefully reviewed all the variables obtained in the analysis and ensured that thy are clearly defined and interpreted with support from relevant literature
**Comment by: Reviewer 3**				
15.	Discussion		Finally, the discussion of the collected data needs to be improved, in dialogue with the scientific literature in the field.	We strengthened the discussion section by incorporating relevant scientific literature
