# Supplementary material for: Determinants of instructional leadership: A survey of public secondary schools
Source: PLoS One. 2026 Aug 14;21(8):e0355674. doi: 10.1371/journal.pone.0355674 (PMC13475869; doi:10.1371/journal.pone.0355674)
Supplement: Supplementary file 4 [file pone.0355674.s007.pdf]

第一版。加利福尼亚州千橡市：贤者出版社。https://doi.org/10.4135/9781483349022
[10] 赖利 B.、布朗 M. 和弗劳尔 S. (2015)。巴布亚新几内亚的政治治理和服务提供：对当前和替代治理体系的战略审查。莫尔兹比港：巴布亚新几内亚国家研究所。取自 https://pngnri.org/images/Publications/DP_143.pdf
[11] STREINER, D.L.、NORMAN, G. 和 CAIRNEY, J. (2015)。健康测量量表：开发和使用的实用指南。第五版。纽约：牛津大学出版社。https://doi.org/10.1093/med/9780199685219.001.0001

Appendix A

Table 1. Demographic information on selected law and justice sector employees in relation to some variables (N = 37) (Coding of field survey data)

Variable	Description	Mean	%	SD	Tolerance	VIF
Sex	1 = male, female = 2	1.270	Male = 27, female = 10	0.450	0.790	1.266
Age	1 = 25-35, 2 = 36-46, 3 = 47-57, 4 = 58-65, 5 = not specified	1.882	25-35 = 40.5, 36-46 = 29.7, 47-57 = 13.5, 58-65 = 8.1, not specified = 8.1	0.977	0.099	10.104
Marital Status	1 = never married, 2 = married, 3 = divorced, 4 = de facto	2.108	Never married = 8.1, married = 83.8, divorced = 2.7, de facto = 5.4	0.774	0.753	1.328
Educational Qualifications	1 = Bachelor's degree, 2 = diploma, 3 = Grade 10, 4 = Grade 12, 5 = Master of Business Administration (MBA), 6 = not indicated	2.750	Bachelor's Degree=24.3, Diploma = 21.6, Grade 10 = 10.8, Grade 12 = 35.1, MBA = 5.4, not indicated = 2.7	1.339	0.722	1.385
Work Experience (Years)	1 = 1-10, 2 = 11-20, 3 = 21-30, 4 = 31-40.	2.054	1-10 = 43.2, 11-20 = 27.0, 21-30 = 10.8, 31-40 = 18.9	1.153	0.085	11.720
Rank	1 = chief inspector, 2 = chief sergeant, 3 = chief superintendent, 4 = constable, 5 = first inspector, 6 = senior constable, 7 = senior inspector, 8 = sergeant	5.405	Chief inspector = 2.7, chief sergeant = 2.7, chief superintendent = 2.7, constable = 43.2, first inspector = 13.5, senior constable = 5.4, senior inspector = 13.5, sergeant = 13.5	2.192	0.521	1.920
Place of Birth in PNG	1 = Highlands Region, 2 = Momase Region, 3 = New Guinea Island Region, 4 = Southern Region	1.812	Highlands Region = 59.5, Momase Region = 16.2, New Guinea Island Region = 8.1, Southern Region = 16.2	1.151	0.669	1.494

Appendix B

Questionnaire on Strategy for Improving Service Delivery in the PNG Law and Justice Sector Research Project [QSISDPNGLJSRP]

The National Security and International Relations Research Program (NSIRRP) at the prestigious Papua New Guinea National Research Institute (PNGNRI) is undertaking a national research project to improve service delivery in the law and justice sector of the PNG national economy. This aspect of the research project aims to "sequentially" explore strategies that are appropriate for ameliorating crimes in PNG and improving the ease of doing business.
Please note that the outcome of this research would enable the researchers in NSIRRP to provide research-informed policies to the stakeholders in PNG regarding crime reduction and ease of doing business.

Section A: Biographical Information

1. Please indicate your sex *(Please tick only one)*

1. Male □ 2. Female □

2. How old are you? *(Please indicate your age)*

……………………………………………………………………………………………

3. Please indicate your marital status *(Please tick only one)*
