# Supplementary material for: Determinants of instructional leadership: A survey of public secondary schools
Source: PLoS One. 2026 Aug 14;21(8):e0355674. doi: 10.1371/journal.pone.0355674 (PMC13475869; doi:10.1371/journal.pone.0355674)
Supplement: Supplementary file 5 [file pone.0355674.s002.docx]

Annex

Demographic information of the respondents

| **Variable** |  | **Description** |  | **Percentage** |  |  |
| --- | --- | --- | --- | --- | --- | --- |
| District |  | 1= District 1 |  | District 1: 61.9 % |  |  |
|  |  | 2= District 2 |  | District 2: 21.2 % |  |  |
|  |  | 3= District 3 |  | District 3: 16.9 % |  |  |
| Position in schools |  | 1= Head teacher |  | Head teacher: 50.0 % |  |  |
|  |  | 2= Teacher |  | Teacher: 50.0 % |  |  |
| Gender |  | 1= Male |  | Male: 78.8 % |  |  |
|  |  | 2= Female |  | Female: 21.2 % |  |  |
|  |  |  |  |  |  |  |
| Years of experience |  | 1= 1 yr. |  | 1 yr.: 9.9 % |  |  |
|  |  | 2= 2 to 4 yrs. |  | 2 to 4 yrs.: 19.5 % |  |  |
|  |  | 3= 5 to 9 yrs. |  | 5 to 9 yrs.: 21.5 % |  |  |
|  |  | 4= 10 to 14 yrs. |  | 10 to 14 yrs.: 16.8 % |  |  |
|  |  | 5= 15 yrs. and above |  | 15 yrs. and above: 32.3 % |  |  |
|  |  |  |  |  |  |  |
| Qualification |  | 1= SLC or PCL |  | SLC or PCL: 3.2 % |  |  |
|  |  | 2= Bachelor |  | Bachelor: 20.6 % |  |  |
|  |  | 3= Master |  | Master: 73.0 % |  |  |
|  |  | 4= MPhil or PhD |  | MPhil or PhD: 3.2 % |  |  |
