# Supplementary material for: Determinants of instructional leadership: A survey of public secondary schools
Source: PLoS One. 2026 Aug 14;21(8):e0355674. doi: 10.1371/journal.pone.0355674 (PMC13475869; doi:10.1371/journal.pone.0355674)
Supplement: Supplementary file 8 [file pone.0355674.s008.docx]

Dear Editor,

Thank you very much for your email and for the repeated rounds of constructive feedback on our manuscript. We sincerely appreciate the time, effort, and thoughtful guidance that you and the reviewers have invested in evaluating our work.

We have carefully addressed each of the comments and have made substantial revisions throughout the manuscript. The iterative process of responding to your feedback has significantly strengthened the quality and clarity of our paper.

We believe that the manuscript is now of publishable quality in your esteemed journal. However, we remain fully committed to making any further improvements that may still be needed.

Thank you once again for your valuable input and consideration. Please find the comment matrix attached below.

Warm regards,

Authors

| Comment Matrix | | | | |
| --- | --- | --- | --- | --- |
| **Comment by: Reviewer 2** | | | | |
| **S.No.** | **Section where revision has been indicated** | **The section presented before revision was advised** | **Revision suggested** | **The section after revision was made according to the reviewers' comments** |
| 1. | Literature Review | The researchers began examining school effectiveness and program improvement in the late 1970s. They first identified effective schools and then analyzed what made them successful, noting that a skillful head teacher was a key factor in driving school improvement [23]. It was emphasized that instructional leadership plays a pivotal role in fostering school improvement, promoting better academic outcomes both directly, by supporting teachers, and indirectly, by improving conditions for teaching and learning. Successful school leadership also involves enriching the curriculum, redefining the roles and responsibilities, enhancing teacher quality, and building strong relationships within the school and with the wider community [24].  Instructional leadership is a construct derived from the combination of the concepts of "instruction" and "leadership." There have been multiple efforts to define instruction, but four key dimensions of instruction remain consistent: setting learning objectives, choosing teaching methods, selecting reinforcement activities, and evaluating outcomes [25]. Researchers hold varying views on leadership as well. Some have defined leadership as how a leader leads; others have described it as the job done through people [25]. Leadership includes followers acting toward goals that represent the values of the leaders and the followers [26]. Such uncertainty surrounding the concept of leadership has made the combined construct of “instructional leadership” difficult to define and clarify. The early instructional leadership models tended to focus on the leadership portion of the construct, and they attempted to define it in terms of general leadership theory. Those models focused on personality/leadership style, behavioral/situational, or contextual components of the head teacher [24].  In this manner, the combined construct of "instructional leadership" has been defined in various ways. Instructional leadership means providing the necessary resources, possessing knowledge and skill in curriculum and instructional matters, being a skilled communicator, and being a visionary who is out and around creating a visible presence [26]. Instructional leadership is direct assistance to teachers in their day-to-day activities, the development of collaborative groups among staff, the design and procurement of effective staff development, curriculum development, and the use of action research [27]. These definitions emphasized not only improving the instructional leadership role of the head teacher through the monitoring of instructional activities, but also focused on the managerial tasks of the head teacher within it. Later scholars of instructional leadership began linking it to activities aimed at enhancing teaching and learning, leading to a slight shift in how instructional leadership was defined. Here are some examples: instructional leadership begins when principals talk about being in the classroom, visit with students about their academic pursuits, and prioritize instruction [28]. Instructional leadership is important for effective practices in curriculum, instruction, and assessment, and for the ability to work with teachers on the day-to-day problems related to these topics [29]. The latter two perspectives on instructional leadership place a strong emphasis on teaching and learning activities. In this way, the concept of instructional leadership evolved through various research developments, ultimately establishing that leadership roles connected to teaching and learning within the school fall under the domain of instructional leadership [30, 31, 2, 32].  The instructional leadership notion has dominated the educational literature since the creation of instructional leadership models, and there is ample proof of its effectiveness regarding teacher and student results [33, 34]. The notable models of instructional leadership have been proposed by various researchers [ e.g.,35, 36, 37, 38, 39, 40, 41]. They have offered a variety of indications of instructional leadership, but the theoretical underpinnings are often the same [40, 41]. Researchers most extensively use the Hallinger and Murphy (1985) model of instructional leadership to research instructional leadership. The widespread acceptance of this model is primarily due to Hallinger and Murphy's (1985) core concept, which provides head teachers with valuable insights into all aspects of leadership related to teaching and learning in schools. The main theme of this model is to develop and communicate school goals, coordinate and supervise the school curriculum, monitor and evaluate student progress, encourage teachers and students, and maintain high visibility and a hands-on approach [23, 44]. Their model is in the form of a scale known as PIMRS (Principal/Head Teacher Instructional Management Rating Scale). This PIMRS has been utilized in at least 130 PhD dissertations over almost three decades [45].  Several academics have mentioned their views on the characteristics of an instructional leader. Instructional leaders perform at high levels in four areas: a) resource provider, b) instructional resource, c) communicator, and d) visible presence in the school [35]. The instructional leaders emphasize: a) goal establishment and curriculum development, b) frequent observations, c) program evaluation, and d) support for teachers [41]. Instructional leaders perform seven crucial functions, which are: a) establishing and implementing instructional goals; b) being available to staff; c) creating a school culture and climate conducive to learning; d) communicating the vision and mission of the school to students, staff, and parents; e) setting high expectations for the staff; f) developing teacher leaders; g) maintaining positive attitudes toward student, staff, and parents [39]. There are four tenets of an instructional leader: a) focus on developing, directing, and supervising the curriculum and instruction in the classroom; b) interacting with teachers to improve teaching and learning c) being goal-oriented; d) establishing high expectations and standards for teachers and students [38]. The effective instructional leader plays five important roles, which are: a) establishing goals and expectations; b) strategic resourcing; c) planning, coordinating, and evaluating teaching and the curriculum; d) promoting and participating in teacher learning and development; and e) ensuring an orderly and supportive environment [40]. There are six characteristics of effective instructional leaders: a) identifying and communicating the mission and vision of the school; b) maintaining a school environment that is conducive to learning; c) promoting the professional development of staff; d) monitoring teachers’ instruction; e) maintaining a positive school climate; and f) initiating organizational change [36]. From these scholars' views, it can be inferred that an instructional leader's job is to improve instruction to increase student achievement. Hence, an instructional leader is a person who seeks to ensure effective teaching and learning in the school by meeting all the requirements necessary to enhance students' academic performance.  Among the models of instructional leadership proposed by various researchers devoted to the study of instructional leadership, Hallinger and Murphy's (1985) model of instructional leadership was used as a theoretical guide for this research because this model has attained top priority and is more dominant in the literature on instructional leadership. This model of instructional leadership consists of three factors: defining the school's mission, managing the instructional program, and promoting a positive learning climate. There are two functions under the factor defining the school's mission, which are framing the school goals and communicating the school goals; three functions under the factor managing the instructional program, which are supervising and evaluating instruction, coordinating curriculum, and monitoring students’ progress; and five functions under the factor promoting a positive learning climate which are protecting instructional time, providing incentives for teachers, providing incentives for learning, promoting professional development of teachers, and maintaining high visibility. There are five items under each of these ten functions, forming fifty variables altogether.  These fifty items were studied thoroughly, and a classical Delphi was conducted, followed by three rounds of meetings with experienced head teachers and teachers to get their concrete ideas on them. Based on their opinions, some items- for example, (i) draw upon the results of class-wise testing when making curriculum decisions, (ii) participate actively in the review of curricular materials, (iii) acknowledge teachers’ exceptional performance by writing memos for their personal files, (iv) recognize superior students’ achievement or improvement by seeing in the office the students with their work, (v) review student work products when evaluating classroom instruction- were deselected from this PIMRS. Some items- for example, (i) head teacher announces mandatory presence of parents during terminal report card distribution, (ii) head teacher meets the parents of poor-performing students and shares with them what roles the school and parents may together take for their improvements, (iii) head teacher learns students’ family problems and talks to their parents to resolve them, (iv) the head teacher is transparent about every financial detail of the school to all stakeholders- were added to the in new PIMRS.  Some items were changed to best suit Nepali schools, such as (i) monitoring the classroom curriculum to see that it covers the school's curricular objectives was changed to the head teacher checking teachers' log books regularly to see if they are going in accordance with the syllabus, (ii) limiting interruption of instructional time by public address announcements was changed to head teacher trying his best to ensure uninterrupted instructional time, (iii) leading or attending teacher in- service activities concerned with instruction was changed to head teacher seeking to provide training to teachers during vacations so as not to interrupt daily classes, (v) conducting informal observations in classrooms regularly was changed to head teacher regularly monitoring the activities of teachers and students while they are in class. In this way, a new PIMRS was finally developed for this research. This PIMRS consists of 11 factors and 33 items. | The literature review section must be strengthened with current literature on instructional leadership. Additionally, the definition or explanation of what constitutes should be reexamined, focusing on three countries or contexts before discussing what constitutes instructional leadership in Nepal. The citation "Hallinger, P., & Murphy J. (1985). Assessing the instructional management behavior of principals. Elementary School Journal, 86(2), 217-248. <https://doi.org/10.1086/461445>" is stale in its contribution to knowledge in the field of instructional leadership.  The authors should note that you cannot complete a research project on "Instructional Leadership", and leave out the contributions of Lees Shulman and other scholars in the United States. I strongly suggest the literature review in this manuscript should discuss the concept of instructional leadership in the United States. | **Reviewing the Literature:**  Instructional leadership is one of the leadership approaches that has gained steadily increasing importance within the overall role of school principals over the past six decades [23]. This growing emphasis arises from evidence demonstrating its substantial impact on student achievement. For instance, a meta-analysis by [24] found that the average effect of instructional leadership on student outcomes was three to four times greater than that of transformational leadership. A few years later, a study of elementary schools in the USA [25] yielded a similar result, showing that instructional leadership accounted for more variance in student outcomes than transformational leadership. Similarly, a study of Malaysian secondary schools by [26] revealed a positive correlation between the level of instructional leadership and students’ academic achievement, suggesting that the instructional leadership role is crucial in achieving better academic outcomes in schools.  Similarly, a meta-analysis conducted in 2017 that included 14 studies from the past decade [27] found a positive effect of instructional leadership on students’ academic outcomes, along with transformational leadership and distributed leadership. This study confirmed the argument that the more leaders are engaged in the business of teaching and learning with their students, the better the academic performance of students. Similarly, a quantitative study in Indonesia by [28] found that instructional leadership is effective in enhancing the quality of learning. In line with previous findings, a recent meta-analysis conducted in 2025 by [29] revealed that the relationships between principal leadership and student achievement have been more frequently investigated through supportive, instructional, distributed, and transformational leadership approaches (23.64%, 21.82%, and 20.00%), respectively. These findings suggest that the relationship between educational leadership and student achievement remains one of the most widely researched topics, with instructional leadership being a major type of leadership studied. Moreover, more than half of the studies included in the meta-analysis were conducted in the USA, indicating that such research is predominantly produced in that context. Further evidence of the prominence of instructional leadership research in the United States is demonstrated by the studies included in Hallinger’s [30] review, which identified 130 doctoral dissertations employing the Hallinger and Murphy instructional leadership model (PIMRS), produced by doctoral students from more than 85 American universities.  The concept of instructional leadership has been defined variously. According to [31], the pioneering scholars who initially formulated a clear understanding of instructional leadership, focusing on U.S. schools in the 1980s and 1990s, identified the principal’s role in aligning school goals, instructional practices, and student outcomes through both direct and indirect management activities. Their framework has been applied internationally. It involves defining the school’s mission, managing the instructional program, and promoting a favorable learning climate to enhance teaching and learning. This framework illustrates that instructional leaders influence classroom practices not only through direct supervision and feedback but also by developing and enforcing school-wide policies, expectations, and structures that guide teacher and student behavior toward common educational goals. However, the central concept of instructional leadership is the leadership role that focuses on teaching and learning activities with the goal of enhancing students’ academic achievement. Various educators have interpreted this concept in different ways according to their own perspectives. A Canadian researcher [32] portrayed instructional leadership as an active role in the classroom practice based on high levels of pedagogical expertise. Similarly, a researcher from Malaysia [26] defined it as leadership focused on teaching and learning activities, fostering interactions between teachers and students centered on the curriculum. Likewise, researchers from the UK [33] emphasized the importance of setting clear educational objectives, planning the curriculum, and evaluating teachers and their educational practices.  Among all, researchers from America have made significant contributions to the study of instructional leadership. They have provided various interpretations of how effective schools develop through instructional leadership. For example, the American educationist Lee Shulman, who observed schools in the late 1960s and early 1970s, argued that while general principles of effective teaching are important, they should not be treated as final measures, as those may not be true in particular and specific circumstances [34]. He emphasized that effective teaching requires teachers’ more specific professional knowledge, such as “wisdom of practices”, and “intersection of content and pedagogy”. Similarly, Grissom, Loeb, and Master, who examined the relationship between principals’ time spent in instructional leadership behaviors and student achievement gains in schools, asserted that the effect of instructional leadership does not depend on time spent engaging in instruction, but on the type and quality of those time investments [35]. Likewise, Hallinger and Heck, who reviewed research from 1980 to 1995 on the impact of principals on school effectiveness and student achievement, identified several pathways through which principals influence learning outcomes, including school goals, school structure, social network, people, and organizational culture [36].  Nepali researchers have also studied instructional leadership from various perspectives and provided their own interpretations. In Nepal, although the instructional leadership role of head teachers, has been incorporated in School Sector Development Plan (2016/17-2022/23) [7], the research on instructional leadership began to appear in the literature since 2020 only. This indicates that, in Nepal, researchers’ focus on this concept has primarily emerged since around 2020. In the context of Nepal, a principal of a public school is referred to as a head teacher. Danai defined instructional leadership as the behavior of head teachers that guides a school in educating all students to achieve high academic standards [37]. Lamsal conceptualized instructional leadership as the position within an educational institution that utilizes individual expertise and knowledge to ensure students’ academic performance [38]. Dahal described the head teacher’s role in coordinating instructors and parents with a clear focus on achieving their shared objectives together, as an instructional leadership role [39]. Drawing on the existing literature, we developed our understanding of instructional leadership as a process in which the head teacher provides support and guidance to the team by managing curriculum and instruction to improve students’ learning outcomes.  The “Australian Effective Schools Project,” which studied school effectiveness by viewing student achievement as one of several key outcomes, identified school leadership as a vital element of effective schools [40]. Based on the literature, it can be argued that among the various forms of leadership, instructional leadership holds even greater importance for effective schools. Instructional leadership promotes student outcomes by positively influencing the factors that affect the teaching-learning process. A recent empirical study conducted in Nigeria demonstrated that instructional leadership has a significant influence on teachers’ professional development and growth [41]. Similarly, a study on the effectiveness of instructional leadership in public secondary schools in Buea employing a non-experimental descriptive-correlation research design showed significant relationships between teachers’ perceptions of principals’ instructional leadership and key dimensions such as defining the school mission (r-cal=3.444, p<0.05), managing the instructional program (r-cal=3.321, p<0.05) and developing the school learning climate (r-cal=3.134, p<0.05) respectively [42]. These findings suggest that instructional leadership has a positive impact on the key factors influencing teaching and learning, thereby enhancing students’ learning achievement.  Instructional leadership is found to be influenced by context. In some countries, particularly those with developed educational systems, it has been more effectively practiced. In a paper, Hallinger illustrated the evolution of research on school leadership over the past three decades and cited the assertion of Edmonds, and Bossert along with his co-authors, that “strong instructional leadership from the principal” is a hallmark of effective urban elementary schools in the USA [43]. Similarly, a meta-analysis of 27 studies published between 1978 and 2006 revealed that the majority of instructional leadership research had been conducted in the USA, with two studies in Canada and one each in Australia, England, Hong Kong, Israel, the Netherlands, New Zealand, and Singapore [24]. This meta-analysis identified five core dimensions of instructional leadership: (i) establishing goals and expectations, (ii) resourcing strategically, (iii) planning, coordinating, and evaluating teaching and the curriculum, (iv) promoting and participating in teacher learning and development, and (v) ensuring an orderly and supportive environment. These findings demonstrate the effective implementation of instructional leadership practices within schools in these contexts. On the contrary, in a study incorporating systematic literature review and qualitative interviews across six sub-Saharan African countries, Nigeria, Sierra Leone, Sudan, Tanzania, Zambia, and Zimbabwe [44] reported that the concept of instructional leadership is weakly understood in these countries. There is a lack of contextualized definitions and a proper understanding of this concept, which is essential to connect it to wider school policies and practices in these settings. These studies illustrate that in developed countries, the concept of instructional leadership has been well contextualized and effectively implemented in practice. In contrast, in developing countries, the concept remains relatively new and has yet to be fully operationalized or grounded in local educational realities.  A contextual understanding of instructional leadership is necessary for its effective enactment because principals’ practices are shaped by their local context, including government policy, available resources, and culture. A systematic “state-of-the-art” research review, including 302 quantitative and mixed methods studies from Asia [45], found that a school’s cultural context moderated the effects of instructional leadership on teaching and learning. Therefore, contextual adaptation of the definition and model of instructional leadership is essential for its effective implementation. In the context of Nepal, a model of instructional leadership that aligns with the local context is crucial, although it has yet to be developed. Keeping this in mind, this study attempted to create an instructional leadership model by conducting a Delphi on Hallinger and Murphy’s (1985) model of instructional leadership. The classical Delphi technique was used to establish consensus among experienced panelists [46] by incorporating the influence of indigenous practices relevant to the Nepali context. |
|  | **Comment by: Reviewer 2** | | | |
| 2. | Research Methods | As the study intends to find out the determining factors of instructional leadership, it is the study of a *cause*-and-effect relationship. It is because those factors can be found out by studying the causes that influence instructional leadership. Therefore, it follows the post-positivistic deterministic philosophy of *causes* determine *effects*. The objective reality of instructional leadership exists out there, and that reality can be found through careful study and measurement. Although the reality of instructional leadership exists independently, it can only be understood through data and evidence. Therefore, this study relies on statistical analysis of numerical data, a method known as quantitative research. The research design is quantitative, and research methods include the preparation of questionnaires, data collection, data analysis, and interpretation. Classical Delphi was conducted on Hallinger and Murphy's (1985) instructional leadership model, known as Principal/Head teacher Instructional Management Rating Scale (PIMRS) to develop a PIMRS suitable in the context of Nepal. A well-structured questionnaire was prepared using the new PIMRS. The questionnaire developed from the new PIMRS with 11 factors and 33 items asked the respondents to rate their view on a 5-point Likert scale with the descriptors: 1= almost never, 2= seldom, 3= sometimes, 4= frequently, and 5= almost always, on which the head teacher was engaged in those particular activities of the school. The data was collected using a survey design, an appropriate way to get quantitative or numerical descriptions of population trends, attitudes, or opinions by surveying a sample of that population [46]. A questionnaire was administered to respondents during visits to their schools. | In the section on research methods, please contextualize this assertion within a research paradigm, i.e., "i.e. Therefore, it follows the postpositivistic deterministic philosophy of causes determine effects", colleagues! In this sentence, "The research design is quantitative, and research methods include the preparation of questionnaires, data collection, data analysis, and interpretation," please state the research design explicitly. What was the research architect's blueprint that you used in constructing the study reported in the research manuscript, colleagues? You may wish to refer to current literature on "Research Designs." | Research Methods:  This study aims to identify the factors that determine instructional leadership. In this context, factors act as causes which probably determine instructional leadership, the effects or outcomes [53]. The determining factors can be identified by examining the influences on instructional leadership. In this sense, the factors act as causes, and the way instructional leadership is affected is the result of those causes. Thus, it is the study of *cause-and-effect* relationships. Therefore, it follows the post-positivistic deterministic philosophy that *causes* determine *effects* or outcomes [54]. Post-positivists explore a single reality that is previously unknown [55]. They assume that reality exists independently, which can be approximated through careful observations and measurements of those variables [53]. Therefore, we made a claim for knowledge based on (i) a causal relationship exists between independent variables (factors) and dependent variable (instructional leadership), and (ii) detailed observation and measurement of variables is essential to obtain knowledge [53, 56]. Following these post-positivistic assumptions, this research used a quantitative research design. Quantitatively, it employed a cross-sectional survey design, serving as the architectural blueprint for the investigation.  The research design is quantitative, and the research methods include preparing questionnaires, collecting data, analyzing data, and interpreting the results. Classical Delphi was employed in Hallinger and Murphy’s (1985) instructional leadership model, known as the Principal/Head Teacher Instructional Management Rating Scale (PIMRS), to develop a PIMRS suitable for the context of Nepal. A well-structured questionnaire was prepared using the new PIMRS. The questionnaire developed from the new PIMRS with 11 factors and 33 items asked the respondents to rate their view on a 5-point Likert scale with the descriptors: 1= almost never, 2= seldom, 3= sometimes, 4= frequently, and 5= almost always, on which the head teacher was engaged in those particular activities of the school. The data were collected using a survey method, a technique that provides quantitative or numerical descriptions of population trends, attitudes, or opinions by surveying a sample of that population [57]. |
|  | **Comment by: Reviewer 2** | | | |
| 3. |  |  | Please consult the second attachment provided to format a table and report the necessary coding and demographic information of the selected respondents to enable check for technical consistencies of the information provided in methodology and results section of the research manuscript. |  |
