# Supplementary material for: Determinants of instructional leadership: A survey of public secondary schools
Source: PLoS One. 2026 Aug 14;21(8):e0355674. doi: 10.1371/journal.pone.0355674 (PMC13475869; doi:10.1371/journal.pone.0355674)
Supplement: Supplementary file 9 [file pone.0355674.s004.docx]

**Factors and items of instructional leadership finalized after conducting Delphi with experienced head teachers and teachers**

| Factors | Items |
| --- | --- |
| Setting schools’ annual academic plans  Sharing plans/ expectations | Head teacher analyses students’ last year’s performance while planning for the new academic session.  Head teacher specifies school’s whole year educational plan in the presence of all concerned people.  Head teacher uses formal or informal methods to secure teachers' input on meeting the schools' goal  Head teacher seeks to incorporate teacher’s inputs in planning during his or her formal or informal meetings. |
| Monitoring and supervision  Implementing curriculum    Monitoring students’ progress  Avoiding interruptions/promoting smooth teaching learning  Maintaining transparency  Motivating teachers    Promoting teachers’ professional development    Motivating students for learning      Involving parents | Head teacher shares academic plans with everyone involved and discusses how those plans may be effectively implemented.  Head teacher displays school’s whole year plan on the notice boards, school calendar and communicates them to students during assembly.  Head teacher reports school’s educational planning to the local government and District/Local level education offices.  Head teacher regularly monitors activities of teachers and students while they are in class.  Head teacher makes sure students’ every class work/home work is completed timely and all notes are marked by assigned teachers at least a month prior to terminal examinations.  Head teacher seeks to find out teachers’ areas to improvements in instruction practices and makes appropriate suggestions.  Head teacher gets every subject department heads to implement the syllabus of all subjects in each class.  Head teacher checks teachers’ log books regularly to see if they are in going in accordance with the syllabus.  Head teacher reviews whether the curriculum goal is achieved or not through students’ terminal examination results, their discipline and overall change indicators.  Head teacher regularly discusses with subject teachers about each student’s progress.  Head teacher conducts monthly meetings with all teachers to review the achievement versus goals.  Head teacher communicates whole school’s terminal results to teachers after each term.  Head teacher tries his best to ensure uninterrupted instruction time.  Head teacher instructs teachers to create a fear-free learning environment in class.  Head teacher visits classrooms sometimes to discuss with teacher and students on issues like admission, fee etc.  Head teacher is transparent about every financial detail of the school to all stakeholders.  Head teacher calls immediate teachers’ meeting to communicate notices received from higher authorities.  Head teacher publicly praises teacher’s superior performance but meets them in private for correction.  Head teacher tries to manage opportunity for sincere teachers to work in a higher position as a reward for their good work.  Head teacher provides letters of appreciation or honor certificates to teachers for their special contribution.  Head teacher seeks to provide trainings to teachers during vacations or so as not to interrupt daily classes.  Head teacher actively supports teachers to use those learnt skills in the classrooms.  Head teacher sometimes provides opportunities to teachers for observation or educational tours.  Head teacher publicly honors students for their excellent performance or discipline.  Head teacher learns students’ family problems and talks to their parents to resolve.  Head teacher instructs teachers to evaluate students’ performance regularly and help them for improvements.  Head teacher calls class-wise parents’ meeting and discusses about students’ progress.  Head teacher announces mandatory presence of parents during terminal report card distribution.  Head teacher meets the parents of poor-performing students and shares with them what roles the school and parents may together take for their improvements. |
|  |  |
