# Supplementary material for: Determinants of instructional leadership: A survey of public secondary schools
Source: PLoS One. 2026 Aug 14;21(8):e0355674. doi: 10.1371/journal.pone.0355674 (PMC13475869; doi:10.1371/journal.pone.0355674)
Supplement: Supplementary file 10 [file pone.0355674.s005.docx]

Dear Editor,

Thank you very much for your email and the constructive feedback provided on our manuscript. We truly appreciate the time and effort you and the reviewers have taken to review our work. The detailed comments were incredibly helpful in enhancing our understanding and improving the quality of the paper.

We have carefully addressed each of the suggestions and made substantial revisions to the manuscript. We believe that these revisions have significantly strengthened the paper.

In accordance waith the journal's data availability criteria, I have attached the dataset along with the manuscript and other supporting files.

We kindly request that you consider the revised manuscript for publication in your esteemed journal. We look forward to your feedback and hope for a favourable outcome.

Thank you once again for your valuable input and consideration. The comment matrix is as follows.

Best regards

Authors

| Comment Matrix | | | | |
| --- | --- | --- | --- | --- |
| **Comment by: Reviewer 1** | | | | |
| **S.No.** | **Section where revision has been indicated** | **The section presented before revision was advised** | **Revision suggested** | **The section after revision was made according to the reviewers' comments** |
| 1. | Literature Review | Researchers attempted to examine school effectiveness or program improvement in the late 1970s. They first identified the effective schools and then analyzed what made them effective. They noticed that a skillful head teacher was a key factor in accounting for successful change or school improvement [23]. This effective school research base assigned the role of instructional leader to the head teachers to develop their skills because it was found that a head teacher would seem to be capable of fulfilling the head teacher’s functions of managing the facility, controlling the operation, taking charge of community relations and overseeing the student progress only when he or she also becomes responsible for instructional leadership [24]. As a result, instructional leadership appeared as an essential construct for making schools effective, but it was still unclear what the actual instructional leadership roles are.  Instructional leadership is a term formed by combining "instruction" and "leadership." There have been multiple efforts to define instruction, but four key dimensions of instruction remain consistent: setting learning objectives, choosing teaching methods, selecting reinforcement activities, and evaluating outcomes [25]. Researchers hold varying views on ledership as well. Some have defined leadership as how a leader leads; others have described it as the job done through people [25]. Leadership includes followers acting toward goals that represent the values of the leaders and the followers [26]. Such uncertainty surrounding the concept of leadership has made the combined construct of “instructional leadership” difficult to define and clarify. The early instructional leadership models tended to focus on the leadership portion of the construct, and they attempted to define it in terms of general leadership theory. Those models focused on personality/ leadership style, behavioral/ situational or contextual components of the head teacher [24].  In this manner, the combined construct of "instructional leadership" has been defined in various ways. Instructional leadership means providing the necessary resources, possessing knowledge and skill in curriculum and instructional matters, being a skilled communicator and being a visionary who is out and around creating a visible presence [26]. Instructional leadership is direct assistance to teachers in their day-to-day activities, development of collaborative groups among staff, design and procurement of effective staff development, curriculum development, and the use of action research [27]. These definitions emphasized not only improving the instructional leadership role of the head teacher through the monitoring of instructional activities but also focused on the managerial task of the head teacher within it. Later scholars of instructional leadership began linking it to activities aimed at enhancing teaching and learning, leading to a slight shift in how instructional leadership was defined. Here are some examples. The instructional leadership begins when principals talk about being in the classroom, visit with students about their academic pursuits, and prioritise instruction [28]. Instructional leadership is important for effective practices in curriculum, instruction, and assessment and the ability to work with teachers on the day-to-day problems related to these topics [29]. The latter two perspectives on instructional leadership place a strong emphasis on teaching and learning activities. In this way the concept of instructional leadership evolved through various research developments, ultimately establishing that leadership roles connected to teaching and learning within the school fall under the domain of instructional leadership [30, 31, 2, 32].  The instructional leadership notion has dominated the educational literature since the creation of instructional leadership models, and there is ample proof of its effectiveness regarding teacher and student results [33, 34]. The notable models of instructional leadership have been proposed by various researchers [ e.g., 35, 36, 37, 38, 39, 40, 41]. They have offered a variety of indications of instructional leadership, but the theoretical underpinnings are often the same [40, 41]. Researchers most extensively use the Hallinger and Murphy (1985) model of instructional leadership to do research on instructional leadership. The widespread acceptance of this model is primarily due to Hallinger and Murphy's (1985) core concept, which provides head teachers with valuable insights into all aspects of leadership related to teaching and learning in schools. The main theme of this model is to develop and communicate school goals, coordinate and supervise the school curriculum, monitor and evaluate student progress, encourage teachers and students, and maintain high visibility and a hands-on approach [23, 44]. Their model is in the form of a scale known as PIMRS (Principal/ Head Teacher Instructional Management Rating Scale). This PIMRS has been utilized in at least 130 PhD dissertations over almost three decades [45].  Several academics have mentioned their view on the characteristics of an instructional leader. The instructional leaders perform at high levels in four areas: a) resource provider, b) instructional resource, c) communicator, and d) visible presence in the school [35]. The instructional leaders emphasize on: a) goal establishment curriculum development, b) frequent observations, c) program evaluation, and d) support for teachers [41]. The instructional leader perform seven crucial functions,which are: a) establishing and implementing instructional goals; b) being available to staff; c) creating a school culture and climate conducive to learning; d) communicating the vision and mission of the school to students, staff, and parents; e) setting high expectations for the staff; f) developing teacher leaders; g) maintaining positive attitudes toward student, staff, and parents [39]. There are four tenets of an instructional leader: a) focus on developing, directing, and supervising the curriculum and instruction in the classroom; b) interact with their teachers to improve teaching and learning c) goal-oriented; d) establish high expectations and standards for teachers and students [38]. The effective instructional leader plays five important roles, which are: a) establishing goals and expectations; b) strategic resourcing; c) planning, coordinating, and evaluating teaching and the curriculum; d) promoting and participating in teacher learning and development; and e) ensuring an orderly and supportive environment [40]. There are six characteristics of effective instructional leaders: a) identify and communicate the mission and vision of the school; b) maintain a school environment that is conducive to learning; c) promote the professional development of staff; d) monitor teachers’ instruction; e) maintain a positive school climate and f) initiate organizational change [36]. From these scholars' view it can be inferred that an instructional leader's job is improving instruction to increase student achievement. Hence, an instructional leader is a person who seeks to ensure effective teaching and learning in the school by meeting all the requirements necessary to enhance students' academic performance.  Among the models of instructional leadership proposed by various researchers devoted to the study of instructional leadership, Hallinger and Murphy's (1985) model of instructional leadership was used as a theoretical guide for this research because, this model has attained top priority and is more dominated in the literature of instructional leadership. This model of instructional leadership consists of three factors: defining the school's mission, managing the instructional program, and promoting a positive learning climate. There are two functions under the factor defining the school's mission, which are framing the school goals and communicating the school goals, and three functions under the factor managing the instructional program, which are supervising and evaluating instruction, coordinating curriculum, and monitoring students’ progress, and five functions under the factor promoting a positive learning climate which are protecting instructional time; providing incentives for teachers; providing incentives for learning; promoting professional development of teachers; and maintaining high visibility. There are five items under each of these ten functions, forming fifty variables altogether.  These fifty items were studied thoroughly, and classical Delphi was conducted, followed by three round meetings with experienced head teachers and teachers to get their concrete ideas on it. Based on their opinion, some items, for example, (i) draw upon the results of class wise testing when making curriculum decisions, (ii) participate actively in the review of curricular materials, (iii) acknowledge teachers’ exceptional performance by writing memos for their personal files, (iv) recognize superior students’ achievement or improvement by seeing in the office the students with their work, (v) review student work products when evaluating classroom instruction were unselected from this PIMRS. Some items, for example, (i) head teacher announces mandatory presence of parents during terminal report card distribution, (ii) head teacher meets the parents of poor-performing students and shares with them what roles the school and parents may together take for their improvements, (iii) head teacher learns students’ family problems and talks to their parents to resolve them, (iv) head teacher is transparent about every financial detail of the school to all stakeholders were added in new PIMRS.  Some items were changed to best suit in Nepali schools, such as (i) monitoring the classroom curriculum to see that it covers the school's curricular objectives was changed to the head teacher checking teachers' log books regularly to see if they are going in accordance with the syllabus, (ii) limit interruption of instructional time by public address announcements was changed to head teacher tries his best to ensure uninterrupted instruction time, (iv) lead or attend teacher in- service activities concerned with instruction was changed to head teacher seeks to provide training to teachers during vacations or so as not to interrupt daily classes, (v) conduct informal observations in classrooms regularly was changed to head teacher regularly monitors activities of teachers and students while they are in class. In this way, finally a new PIMRS was developed for this research. This PIMRS consists of 11 factors and 33 items. | The manuscript is generally intelligible and written in standard English, though there are occasional grammatical errors and redundancies, particularly in the literature review and policy background sections | Researchers began examining school effectiveness and program improvement in the late 1970s. They first identified effective schools and then analyzed what made them successful, noting that a skillful head teacher was a key factor in driving school improvement [23]. It was also emphasized that instructional leadership plays a pivotal role in fostering school improvement, promoting better academic outcomes both directly, by supporting teachers, and indirectly, by improving conditions for teaching and learning. Successful school leadership involves improving conditions for teaching and learning, redesigning and enriching the curriculum, redesigning the rolesw and responsibilities, enhancing teacher quality, building relationships inside the school and community, enhancing teaching and learning and building relationship outside the school community [24].  Instructional leadership is a construct derived form the combination of the concepts of "instruction" and "leadership." There have been multiple efforts to define instruction, but four key dimensions of instruction remain consistent: setting learning objectives, choosing teaching methods, selecting reinforcement activities, and evaluating outcomes [25]. Researchers hold varying views on ledership as well. Some have defined leadership as how a leader leads; others have described it as the job done through people [25]. Leadership includes followers acting toward goals that represent the values of the leaders and the followers [26]. Such uncertainty surrounding the concept of leadership has made the combined construct of “instructional leadership” difficult to define and clarify. The early instructional leadership models tended to focus on the leadership portion of the construct, and they attempted to define it in terms of general leadership theory. Those models focused on personality/leadership style, behavioral/situational, or contextual components of the head teacher [24].  In this manner, the combined construct of "instructional leadership" has been defined in various ways. Instructional leadership means providing the necessary resources, possessing knowledge and skill in curriculum and instructional matters, being a skilled communicator, and being a visionary who is out and around creating a visible presence [26]. Instructional leadership is direct assistance to teachers in their day-to-day activities, the development of collaborative groups among staff, the design and procurement of effective staff development, curriculum development, and the use of action research [27]. These definitions emphasized not only improving the instructional leadership role of the head teacher through the monitoring of instructional activities but also focused on the managerial tasks of the head teacher within it. Later scholars of instructional leadership began linking it to activities aimed at enhancing teaching and learning, leading to a slight shift in how instructional leadership was defined. Here are some examples: instructional leadership begins when principals talk about being in the classroom, visit with students about their academic pursuits, and prioritize instruction [28]. Instructional leadership is important for effective practices in curriculum, instruction, and assessment, and for the ability to work with teachers on the day-to-day problems related to these topics [29]. The latter two perspectives on instructional leadership place a strong emphasis on teaching and learning activities. In this way, the concept of instructional leadership evolved through various research developments, ultimately establishing that leadership roles connected to teaching and learning within the school fall under the domain of instructional leadership [30, 31, 2, 32].  The instructional leadership notion has dominated the educational literature since the creation of instructional leadership models, and there is ample proof of its effectiveness regarding teacher and student results [33, 34]. The notable models of instructional leadership have been proposed by various researchers [ e.g.,35, 36, 37, 38, 39, 40, 41]. They have offered a variety of indications of instructional leadership, but the theoretical underpinnings are often the same [40, 41]. Researchers most extensively use the Hallinger and Murphy (1985) model of instructional leadership to conduct research on instructional leadership. The widespread acceptance of this model is primarily due to Hallinger and Murphy's (1985) core concept, which provides head teachers with valuable insights into all aspects of leadership related to teaching and learning in schools. The main theme of this model is to develop and communicate school goals, coordinate and supervise the school curriculum, monitor and evaluate student progress, encourage teachers and students, and maintain high visibility and a hands-on approach [23, 44]. Their model is in the form of a scale known as PIMRS (Principal/Head Teacher Instructional Management Rating Scale). This PIMRS has been utilized in at least 130 PhD dissertations over almost three decades [45].  Several academics have mentioned their views on the characteristics of an instructional leader. I Instructional leaders perform at high levels in four areas: a) resource provider, b) instructional resource, c) communicator, and d) visible presence in the school [35]. The instructional leaders emphasize: a) goal establishment and curriculum development, b) frequent observations, c) program evaluation, and d) support for teachers [41]. Instructional leaders perform seven crucial functions,which are: a) establishing and implementing instructional goals; b) being available to staff; c) creating a school culture and climate conducive to learning; d) communicating the vision and mission of the school to students, staff, and parents; e) setting high expectations for the staff; f) developing teacher leaders; g) maintaining positive attitudes toward student, staff, and parents [39]. There are four tenets of an instructional leader: a) focus on developing, directing, and supervising the curriculum and instruction in the classroom; b) interacting with teachers to improve teaching and learning c) being goal-oriented; d) establish high expectations and standards for teachers and students [38]. The effective instructional leader plays five important roles, which are: a) establishing goals and expectations; b) strategic resourcing; c) planning, coordinating, and evaluating teaching and the curriculum; d) promoting and participating in teacher learning and development; and e) ensuring an orderly and supportive environment [40]. There are six characteristics of effective instructional leaders: a) identifying and communicating the mission and vision of the school; b) maintain a school environment that is conducive to learning; c) promote the professional development of staff; d) monitor teachers’ instruction; e) maintain a positive school climate and f) initiate organizational change [36]. From these scholars' views, it can be inferred that an instructional leader's job is improving instruction to increase student achievement. Hence, an instructional leader is a person who seeks to ensure effective teaching and learning in the school by meeting all the requirements necessary to enhance students' academic performance.  Among the models of instructional leadership proposed by various researchers devoted to the study of instructional leadership, Hallinger and Murphy's (1985) model of instructional leadership was used as a theoretical guide for this research because this model has attained top priority and is more dominat in the literature on instructional leadership. This model of instructional leadership consists of three factors: defining the school's mission, managing the instructional program, and promoting a positive learning climate. There are two functions under the factor defining the school's mission, which are framing the school goals and communicating the school goals; three functions under the factor managing the instructional program, which are supervising and evaluating instruction, coordinating curriculum, and monitoring students’ progress; and five functions under the factor promoting a positive learning climate which are protecting instructional time, providing incentives for teachers, providing incentives for learning, promoting professional development of teachers, and maintaining high visibility. There are five items under each of these ten functions, forming fifty variables altogether.  These fifty items were studied thoroughly, and a classical Delphi was conducted, followed by three rounds of meetings with experienced head teachers and teachers to get their concrete ideas on it. Based on their opinions, some items- for example, (i) draw upon the results of class-wise testing when making curriculum decisions, (ii) participate actively in the review of curricular materials, (iii) acknowledge teachers’ exceptional performance by writing memos for their personal files, (iv) recognize superior students’ achievement or improvement by seeing in the office the students with their work, (v) review student work products when evaluating classroom instruction- were deselected from this PIMRS. Some items- for example, (i) head teacher announces mandatory presence of parents during terminal report card distribution, (ii) head teacher meets the parents of poor-performing students and shares with them what roles the school and parents may together take for their improvements, (iii) head teacher learns students’ family problems and talks to their parents to resolve them, (iv) the head teacher is transparent about every financial detail of the school to all stakeholders- were added to the in new PIMRS.  Some items were changed to best suit Nepali schools, such as (i) monitoring the classroom curriculum to see that it covers the school's curricular objectives was changed to the head teacher checking teachers' log books regularly to see if they are going in accordance with the syllabus, (ii) limiting interruption of instructional time by public address announcements was changed to head teacher trying his best to ensure uninterrupted instructional time, (iii) leading or attending teacher in- service activities concerned with instruction was changed to head teacher seeking to provide training to teachers during vacations so as not to interrupt daily classes, (v) conducting informal observations in classrooms regularly was changed to head teacher regularly monitoring the activities of teachers and students while they are in class. In this way, a new PIMRS was finally developed for this research. This PIMRS consists of 11 factors and 33 items. |
|  | **Comment by: Reviewer 1** | | | |
| 2. | Result | The factor analysis yielded 25 items under three factors. Fourteen of these twenty-five variables fall under factor one, eight under factor two, and three under factor three.  Factor One: Planning, managing and supervising instructional program  All the items of factor one are shown below in the rotated component matrix of this factor.  **Table 1**  *Rotated Component Matrix of Factor One of IL*  These fourteen variables are connected to five distinct instructional leadership roles: planning and controlling school educational activities, overseeing and evaluating instruction, coordinating the curriculum and other educational objectives, keeping track of students' progress, and utilizing instructional time. These specific instructional leadership functions of the head teacher, except a last one, the head teacher directs teachers to make sure no student has skipped class, are similar to the items in Hallinger and Murphy's (1985) framework of IL falling under their two factors, defining the school mission, and managing instructional program [58-61]. Therefore, by including the meaning of those three leadership functions, namely defining the school mission, managing the instructional program, and ensuring no student has bunked the class, this factor was appropriately named as planning, managing, and supervising instructional program.  The factor planning, managing and supervising instructional program is about making an educational plan for the school, coordinating the curriculum, sharing plans, observing instructors to ensure they are practicing good teaching techniques, communicating with teachers to learn about their students’ development, and making attempts to improve. There were two factors: framing and communicating school goals and supervising and evaluating instruction for these particular instructional leadership activities in Hallinger and Murphy's (1985) model [37]. They mentioned, it is about setting and expressing goals, working with staff members and teachers, and managing the instructional process and curriculum. It involves establishing the school's goals, reminding teachers of the purposes and mission, and informing teachers that teachers need to plan their classes properly [62]. A school lacks any standards to assess its success if it has no plan for improving the educational process. This factor is concerned with choosing the primary goals of the school as well as coordinating and managing curriculum and instruction [63]. Planning, managing, and supervising instructional program entails about articulating and expressing the school's goals, coordinating the curriculum, and monitoring and controlling the program by supervising and evaluating instruction [64].  Factor Two: Motivating and developing competence of teachers and students  All the items of factor two are shown below in the rotated component matrix of this factor.  **Table 2**  *Rotated Component Matrix of Factor Two of IL*  Factor two consists of eight variables which explain the role of an instructional leader in motivating and supporting teachers and students for their academic growth. These eight variables serve similar functions to the variables in the Hallinger and Murphy's (1985) Framework of instructional leadership placed under the functions providing incentives for teachers, providing incentives for learning, and promoting professional development under the factor creating a positive school climate [58-61]. Since the obtained eight variables of this factor share the same aim of inspiring teachers and students for their development, a suitable name, ‘motivating and developing the competence of teachers and students’ was given to this factor.  The factor motivating and developing the competence of teachers and students is about encouraging teachers and students to develop their abilities and academic excellence. Praising and rewarding teachers and students for their good deeds, assisting teachers with their professional growth, and supporting students in improving their self-confidence and academic focus are the key roles of an instructional leader, which are crucial in achieving academic success in the school. Recognizing and rewarding teachers' and students' accomplishments is important to develop interest in them [62]. These instructional leadership roles correspond to two functions in Hallinger and Murphy's (1985) instructional leadership model: promoting professional development, and offering incentives for learning. This factor focuses on inspiring and fostering teachers' and students' competence [64]. The overall focus is on helping both teachers and students get better.  Factor Three: Involving Parents  All the items of factor three are shown below in the rotated component matrix of this factor.  **Table 3**  *Rotated Component Matrix of Factor Three of IL*  The factor three of instructional leadership consists of three items. These items are linked to the head teachers' policy to get parents/guardians involved in knowing about their children's condition and taking the appropriate action if necessary. In accordance with the meaning of these variables, a suitable name involving parents was given to this factor.  The core idea summarized in the variables of this factor is to enhance parent/guardian-school communication for students' academic development. The parents/guardians are also made accountable along with the school through this strategy. Parental role is found to have given more importance to students' academic progress in the Nepali context. Although there are studies [65, 66] that have shown a positive relationship between parental involvement and students' academic achievement in the international context as well, but the parent's involvement is not mentioned as a factor in the researchers' instructional leadership models whoever have proposed such model. | The structure of the results section could be improved with clearer subheadings, and some sentences would benefit from greater clarity and conciseness. Terms such as “post-positivist paradigm” should be defined for broader accessibility. | The factor analysis yielded three instructional leadership factors. Fourteen items loaded on the first factor, eight on the second, and three on the third.  Factor One: Planning, managing and supervising instructional program  All the items of factor one are shown below in the rotated component matrix of this factor.  **Table 1**  *Rotated Component Matrix of Factor One of IL*    **Explanation of Variables in Factor One**  These fourteen variables are connected to five distinct instructional leadership roles: planning and controlling school educational activities, overseeing and evaluating instruction, coordinating the curriculum and other educational objectives, keeping track of students' progress, and utilizing instructional time. These specific instructional leadership functions of the head teacher, except the head teacher directs teachers to make sure no student has skipped class, are similar to the items in Hallinger and Murphy's (1985) framework of IL falling under their two factors, defining the school mission, and managing instructional program [58, 59, 60, 61]. Therefore, by including the meaning of those three leadership functions, namely defining the school mission, managing the instructional program, and ensuring no student has bunked the class, this factor was appropriately named as planning, managing, and supervising the instructional program. All the variables of the factor planning, managing, and supervising instructional program show that it is about making an educational plan for the school, coordinating the curriculum, sharing plans, observing instructors to ensure they are practicing good teaching techniques, communicating with teachers to learn about their students’ development, and making attempts to improve. There were two factors: framing and communicating school goals, and supervising and evaluating instruction for these particular instructional leadership activities in Hallinger and Murphy's (1985) model [37]. They mentioned, it is about setting and expressing goals, working with staff members and teachers, and managing the instructional process and curriculum. According to [62] this involves establishing the school's goals, reminding teachers of its purposes and mission, and informing teachers that teachers need to plan their classes properly. A school lacks any standards to assess its success without a plan to improve the educational process. As stated by [63] this factor relates to selecting the schools' primary goals as well as coordinating and managing curriculum and instruction. Similarly, [64] notes that planning, managing, and supervising instructional programs entails articulating and expressing the school's goals, coordinating the curriculum, and monitoring and controlling the program by supervising and evaluating instruction.  This factor also resonates with the conceptualization of instructional leadership by [65], which is grounded in head teachers' core practices and was developed to explain how Norwegian school leaders build their instructional capacity. Their multidimensional model consisting of goal orientation, collaboration with teachers, professional development, observation, and supervision is similar to the variables of the factor planning, managing and supervising instructional program identified in this study. Both undersore head teachers' active role in shaping and monitoring instructional processes as a foundation for improving teaching and learning.  This finding can also be compared with those [ 66], where coordinating the curriculum and providing professional learning opportunities to teachers emerged as statistically significant predictors of teachers' professional development in the context of Nigeria. Coordinating the curriculum is parallel to the variable "the head teacher gets every subject department heads to implement the syllabus of all subjects in each class" within the factor of planning, managing and supervising instructional program. This reinforces the importance of this variable for developing teaching and learning ability. The next predictor, providing professional learning opportunities to teachers, corroborates the variables of the second factor, motivating and developing competence of teachers and students developed in this study.  Factor Two: Motivating and developing competence of teachers and students  All the items of factor two are shown below in the rotated component matrix of this factor.  **Table 2**  *Rotated Component Matrix of Factor Two of IL*  **Explanation of Variables in Factor Two**  Factor two consists of eight variables which explain the role of an instructional leader in motivating and supporting teachers and students for their academic growth. These eight variables serve similar functions to the variables in the Hallinger and Murphy's (1985) Framework of instructional leadership placed under the functions providing incentives for teachers, providing incentives for learning, and promoting professional development under the factor creating a positive school climate [58, 59, 60, 61]. Since the obtained eight variables of this factor share the same aim of inspiring teachers and students for their development, a suitable name, ‘motivating and developing competence of teachers and students’ was given to this factor.  All the variables of the factor motivating and developing the competence of teachers and students show that it is about encouraging teachers and students to develop their abilities and academic excellence. Praising and rewarding teachers and students for their good deeds, assisting teachers with their professional growth, and supporting students in improving their self-confidence and academic focus are the key roles of an instructional leader, which are crucial in achieving academic success in the school. As stated by [62], recognizing and rewarding teachers' and students' accomplishments is important for fostering their interest and engagement . These instructional leadership roles align withtwo functions in Hallinger and Murphy's (1985) instructional leadership model, promoting professional development, and providing incentives for learning. According to [64], this factor emphasizes on inspiring and enhancing the competence of both teachers and students . The overall focus is on helping both teachers and students get better.  The variables within this factor, such as offering training to teachers during vacations, supporting the classroom application of acquired skills, and facilitating educational tours corroborate the finding of [66] who found that providing professional learning opportunities to teachers supports their professional development. In contrast to the finding of [66] who did not find providing incentives for teachers to be a significant predictor of teachers' professional development, the present study suggests that motivational strategies such as public praise, appreciation letters, honor certificates, and rewarding sincere teachers with opportunities for career development are integral to strengthening teachers' competence. Additionally, the inclusion of student focused practices such as honoring excellent performance and addressing family problems reflects a broader and more relational approach for both teachers' and learners' holistic development.  Factor Three: Involving Parents  All the items of factor three are shown below in the rotated component matrix of this factor.  **Table 3**  *Rotated Component Matrix of Factor Three of IL*  **Explanation of Variables in Factor Three**  The factor three of instructional leadership consists of three items. These items are linked to the head teachers' policy to get parents/guardians involved in knowing about their children's condition and taking the appropriate action if necessary. In accordance with the meaning of these variables, a suitable name involving parents was given to this factor. The core idea summarized in the variables of this factor is to enhance parent/guardian-school communication for students' academic development. The parents/guardians are also made accountable along with the school through this strategy. Parental role is found to be more important to students' academic progress in the Nepali context. There are studies that have shown importance of the parent-school relationship. For example, some studies [67 , 68 ] have shown a positive relationship between parental involvement and students' academic achievement, [69] reported that the increasing parental and community involvement in the educational process results in stronger relationships between school leaders and the community. Similarly, [70] emphasized the need for developing relational behavior for school administrators, including interaction with parents. A recent research [71] conducted in Hong Kong, and Macao also demonstrated that leadership can sinfluence student achievement through **Parental Academic Commitment (PAC).** This reinforces the idea that parent involvement is not just a peripheral activity but a leadership-mediated pathway to better student outcomes. |
|  | **Comment by: Reviewer 1** | | | |
| 3. | Abstract & Research Methods | This research follows a post-positivist paradigm relying on statistical analysis of numerical data. | Terms such as “post-positivist paradigm” should be defined for broader accessibility. Overall, the manuscript communicates its findings effectively, but a thorough language and style revision is recommended to address minor issues and improve readability. | The objective reality of instructional leadership exists out there, and that reality can be found through careful study and measurement. Although the reality of instructional leadership exists independently, it can only be understood through data and evidence. Therefore, this study relies on statistical analysis of numerical data, a method known as quantitative research.  Language and style revision is also done |
| **Comment by: Reviewer 1** | | | | |
| 4. | Regarding data availability |  | Regarding data availability, the manuscript states that data are with the authors and can be provided if necessary. However, this does not fully comply with PLOS ONE’s data policy, which requires that all data underlying the findings be made publicly available without restriction. The authors should deposit the anonymized dataset in a public repository or provide it as supplementary information to ensure compliance with journal requirements. | Data will be submitted along with the manuscript and other required files |
| **Comment by: Reviewer 2** | | | | |
| 5. | Regarding output files of factor analysis |  | The authors should provide the outputs of the factor analysis, which was completed using the Statistical Package for Social Sciences 29.0. Based on the information provided regarding the factor loadings of the questionnaire items, reviewers need to verify the factor loadings outputs to ensure the data analysis's accuracy. | The output files of factor analysis will be attached |
| **Comment by: Reviewer 3** | | | | |
| 6. | Abstract | Instructional leadership is the leadership role associated with teaching and learning that academic heads must take on in order to demonstrate higher levels of student accomplishment. This study explores the factors influencing instructional leadership in Kathmandu, Nepal. Hallinger and Murphy (1985) model of instructional leadership is taken as the fundamental basis of this study. This model is subjected to Classical Delphi to develop a scale for this study. Exploratory factor analysis is conducted in the data to extract the factors. Three factors are formed and named as planning, managing and supervising instructional program; motivating and developing competence of teachers and students; and involving parents. The factors identified are supported by existing research. This research follows a post-positivist paradigm relying on statistical analysis of numerical data. The findings are valuable for school head teachers, schools' other stakeholders, concerned government officials of educational sector, and international researchers in instructional leadership.  **Keywords**  Instructional leadership, School, Head teacher, Student, Academic achievement | The abstract and keywords do not provide adequate information about the article. Although the objective and topic addressed in the manuscript are mentioned, the research methodology, main results, and conclusions found in the study are missing. | Instructional leadership is centered on teaching and learning that academic leaders undertake to promote higher levels of student learning. Given its importance, this study examines the factors influencing instructional leadership in Kathmandu, Nepal. The framework for the study is grounded in Hallinger and Murphy’s (1985) model of instructional leadership, which was employed as the conceptual basis. Using the Classical Delphi method, the model was adapted to develop a measurement scale, while the study was situated within a post-positivist paradigm, assuming that the reality of instructional leadership exists independently. Exploratory factor analysis was then conducted to extract the underlying factors. The findings reveal three major factors influencing instructional leadership: (a) planning, managing, and supervising instructional programs; (b) motivating and developing the competence of teachers and students; and (c) involving parents in the instructional process. The inclusion of parental involvement emerged as a novel dimension in the context of Nepal. These results provide meaningful insights for school headteachers, other educational stakeholders, policymakers in the government sector, and international researchers interested in instructional leadership.  **Keywords**  Instructional leadership, School, Head teacher, Student, Academic achievement, Factors |
| **Comment by: Reviewer 3** | | | | |
| 7. | Reference |  | Check whether all authors cited in the article are included in the manuscript's references, and vice versa. | Checked |
| **Comment by: Reviewer 3** | | | | |
| 8. | Introduction | Unsatisfactory school performance over the years is evident from the SLC (School Leaving Certificate) results before the Education Act 2016, and SEE (Secondary Education Examination) results after the Education Act 2016 [16, 12, 17, 11, 18-22]. The unsatisfactory performance of public schools in Nepal is a blatant indication of the head teachers' inadequate effort in the teaching and learning process in these schools. What could be the cause of this failure? To determine the cause of any occurrence, it is necessary to understand the factors influencing the outcome. In this regard, there seems an urgent need to know the factors affecting head teachers' role in teaching and learning, which is called the instructional leadership role. Although there is a large amount of literature on instructional leadership, a study to find the factors that affect the instructional leadership of head teachers in the local context of Nepal has not been found so far in the literature. Therefore, it seemed imperative to research the factors that influence the instructional leadership of head teachers. So, the research question developed for this study is, what factors affect head teachers' instructional leadership. Identifying the factors that influence instructional leadership would help head teachers identify where they are falling short and where they need to advance. This research is believed to be particularly helpful in helping head teachers build their professional abilities as effective instructional leaders. This study can improve the students' achievement in the classroom and supplement to what is already known about instructional leadership. | 5. The article has issues with clarity and coherence in its language, which need to be revised. The Introduction doesn't clarify the exact objective of the research (it differs from the objective stated in the Abstract) and the central research question. Please review this. | Unsatisfactory school performance over the years is evident from the SLC (School Leaving Certificate) results before the Education Act 2016, and SEE (Secondary Education Examination) results after the Education Act 2016 [16, 12, 17, 11, 18, 19, 20, 21,22]. The unsatisfactory performance of public schools in Nepal is a blatant indication of the head teachers' inadequate effort in the teaching and learning process in these schools, a responsibility that falls under their instructional leadership role.. In this regard, there is an urgent need to identify the influencing factors of head teachers' instructional leadership. Although there are a number of literature on instructional leadership, a study to find the factors that affect the instructional leadership of head teachers in the local context of Nepal has not been found so far in the literature. Therefore, it was deemed imperative to identify the factors affecting head teachers' instructional leadership. So, the research question developed for this study is, what are the factors that affect head teachers' instructional leadership. Identifying the factors affecting head teachers' instructional leaderahip would help head teachers recognize areas where they are falling short and where they need to improve. This research is expected to support head teachers developing their professional abilities as effective instructional leaders, which, in turn, can enhance students' achievement in schools. Furthermore, the finding of this study can contribute to the existing body of knowlwdge on instructional leadership. |
| **Comment by: Reviewer 3** | | | | |
| 9. | Methodology | As the study intends to find out the determining factors of instructional leadership, it is the study of a *cause*-and-effect relationship. It is because those factors can be found out by studying the causes that influence instructional leadership. Therefore, it follows the post-positivistic deterministic philosophy of *causes* determine *effects*. The objective reality of instructional leadership exists out there, and that reality can be found out through careful study and measurement. The research design is quantitative, and research methods include the preparation of questionnaires, data collection, data analysis, and interpretation. A well-structured questionnaire was prepared using the new PIMRS. The new PIMRS with 11 factors and 33 items asked the respondents to rate their view on a 5-point Likert scale with the descriptors; 1= almost never, 2= seldom, 3= sometimes, 4= frequently, and 5= almost always at which the head teacher was engaged in those particular activities of the school. The data was collected using a survey design, an appropriate way to get quantitative or numerical descriptions of population trends, attitudes, or opinions by surveying a sample of that population [46].  The population of this study includes all public secondary schools in Kathmandu Valley. Kathmandu Valley was chosen for the study because it has the greatest concentration of public secondary schools compared to any other region in the nation. In total, 303 public secondary schools in the Kathmandu Valley made up the population of this study and 172 schools made up the sample size. 172 schools were chosen at random from a pool of 303 to visit to gather data. Data was collected from one teacher and the head teacher each from 172 schools to see the difference in head teachers' and teachers' ratings. One teacher from each school was chosen by the lottery process. | Regarding the methodology, it's necessary to clarify how the questionnaires were designed and administered to the research participants, and how the data analysis and interpretation were performed. | As the study intends to find out the determining factors of instructional leadership, it is the study of a *cause*-and-effect relationship. It is because those factors can be found out by studying the causes that influence instructional leadership. Therefore, it follows the post-positivistic deterministic philosophy of *causes* determine *effects*. The objective reality of instructional leadership exists out there, and that reality can be found out through careful study and measurement. The research design is quantitative, and research methods include the preparation of questionnaires, data collection, data analysis, and interpretation. Classical Delphi was conducted on Hallinger and Murphy's (1985) instructional leadership model, known as Principal/Head teacher Instructional Management Rating Scale (PIMRS) to develop a PIMRS suitable in the context of Nepal. A well-structured questionnaire was prepared using the new PIMRS. The questionnaire developed from new PIMRS with 11 factors and 33 items asked the respondents to rate their view on a 5-point Likert scale with the descriptors; 1= almost never, 2= seldom, 3= sometimes, 4= frequently, and 5= almost always at which the head teacher was engaged in those particular activities of the school. The data was collected using a survey design, an appropriate way to get quantitative or numerical descriptions of population trends, attitudes, or opinions by surveying a sample of that population [46]. Questionaire was administered to respondents during visits to their schools.  The population of this study includes all public secondary schools in Kathmandu Valley. Kathmandu Valley was chosen for the study because it has the greatest concentration of public secondary schools compared to any other region in the nation. In total, 303 public secondary schools in the Kathmandu Valley made up the population of this study and 172 schools made up the sample size. 172 schools were chosen at random from a pool of 303 to visit to gather data. Data was collected from one teacher and the head teacher each from 172 schools to see the difference in head teachers' and teachers' ratings. One teacher from each school was chosen by the lottery process. Factor analysis was conducted on the obtained data to identify the factors affecting head teachers' instructional leadership. The findings were analysed and interpreted in the light of the relevant literature. |
| **Comment by: Reviewer 3** | | | | |
| 10. | Conclusion | The study concludes that there are three factors determining instructional leadership. The first factor, planning, managing and supervising instructional programs, explains the head teacher's role in collaborating with teachers to ensure that the school has a clear mission and that mission concentrates on students' academic achievement. The second factor, motivating and developing the competence of teachers and students describes the head teacher's activities to boost teachers' and students' interest in their line of work. The third factor, involving parents mentions the roles of parents/ guardians in association with school for students' academic progress. The study's key finding is that the parents'/ guardians' involvement in students' progress is seen as an essential factor in the schools of Nepal which is different from Hallinger and Murphy's (1985) model of instructional leadership. In Hallinger and Murphy's (1985) instructional leadership model, the parents'/guardians' involvement is not explicitly recognized as a factor in student success. Instead, the model includes only one related item, contacting parents to communicate improved or exemplary student performance or contributions, which falls under the factor "providing incentives for learning". This suggests that Nepal's unique cultural and societal framework places greater emphasis on the role of parents/ guardians in students' academic achievement. | The conclusions lack clarity on whether the central research question was answered, whether the hypotheses were confirmed, and the relevance of the research to the field of study. | In response to the research question, what factors affect head teachers' instructional leadership, the study identified three factors. The first factor, planning, managing and supervising instructional programs, explains the head teacher's role in collaborating with teachers to ensure that the school has a clear mission and that mission concentrates on students' academic achievement. The second factor, motivating and developing competence of teachers and students describes the head teacher's activities to boost teachers' and students' interest in their line of work. The third factor, involving parents mentions the roles of parents/ guardians in association with school for students' academic progress. The study's key finding is that the parents'/ guardians' involvement in students' progress is seen as an essential factor in the schools of Nepal which is different from Hallinger and Murphy's (1985) model of instructional leadership. In Hallinger and Murphy's (1985) instructional leadership model, the parents'/guardians' involvement is not explicitly recognized as a factor in student success. Instead, the model includes only one related item, contacting parents to communicate improved or exemplary student performance or contributions, which falls under the factor "providing incentives for learning". This suggests that Nepal's unique cultural and societal framework places greater emphasis on the role of parents/ guardians in students' academic achievement. By identifying these factors this study contributes to the field of instructional leadership and underscores specific requirements for effective teaching and learning in the public schools of Nepal. |
| **Comment by: Reviewer 3** | | | | |
| 11. | Literature Review |  | There is a connection between the theme and the theoretical foundation. However, I don't understand why the author included two literature review topics (Literature Review and Theoretical Foundation), when they are the same thing. It's important to review this so that the topic discussed in this theoretical section isn't redundant and doesn't confuse the reader when reading the research | The heading "Theoretical Foundation" is removed |
| **Comment by: Reviewer 3** | | | | |
| 12. | Discussion |  | There are some issues with the data analysis and the coherence of the argument. | We have strengthened the coherence of our argument by adding relevant literature that aligns with and supports the results obtained in this study.  In the track-change mode manuscript, and in the review matrix the text in red indicates the changes or additions  . |
| **Comment by: Reviewer 3** | | | | |
| 13. | Result | The factor analysis yielded 25 items under three factors. Fourteen of these twenty-five variables fall under factor one, eight under factor two, and three under factor three. | The following statement is confusing: "Factor analysis resulted in 25 items under three factors. Fourteen of these twenty-five items fall under factor one, eight under factor two, and three under factor three." Please explain better what this means and what you mean by it. Make this information clear to the reader, as well as how you arrived at this conclusion. | The factor analysis yielded three instructional leadership factors. Fourteen items loaded on the first factor, eight on the second, and three on the third.  We arrived to this conclusion from the result of factor analysis |
| **Comment by: Reviewer 3** | | | | |
| 14. |  |  | Furthermore, it is important to review all the variables obtained. | We have carefully reviewed all the variables obtained in the analysis and ensured that thy are clearly defined and interpreted with support from relevant literature |
| **Comment by: Reviewer 3** | | | | |
| 15. | Discussion |  | Finally, the discussion of the collected data needs to be improved, in dialogue with the scientific literature in the field. | We strengthened the discussion section by incorporating relevant scientific literature |
