# Supplementary material for: Determinants of instructional leadership: A survey of public secondary schools
Source: PLoS One. 2026 Aug 14;21(8):e0355674. doi: 10.1371/journal.pone.0355674 (PMC13475869; doi:10.1371/journal.pone.0355674)
Supplement: Supplementary file 11 [file pone.0355674.s009.docx]

**PLOS ONE**

**Determinants of instructional leadership: A survey of public secondary schools**

**Review Report**

**Recommendation: Minor Revision**

This study explores instructional leadership factors in Nepalese public secondary schools, adapting Hallinger & Murphy's model through Delphi and EFA. The identification of three factors planning/managing/supervising, motivating/developing competence, and involving parents, offers a culturally relevant contribution, particularly the emergence of parental involvement as a distinct factor**.**

However, several minor issues require attention:

- Add references within the last 5 years.
- Add future perspective of the Research.
- Tables missing: Tables 1–3 (rotated component matrices) must be included.
- Delphi clarity: Provide panel size, selection criteria, and consensus definition.
- Data availability: The statement "Data is with authors" does not meet PLOS ONE policy. Deposit data in a public repository or justify restrictions.
- Writing quality: Numerous grammatical errors and typos need correction (e.g., "of of" in title).
- Discussion: Briefly explain why parental involvement emerged as a factor in Nepal's cultural context.
- These revisions can be completed within 2–4 weeks. The manuscript has merit but requires these improvements before acceptance.
- **Recommendation:**
- **Minor Revision**
