# Supplementary material for: Determinants of instructional leadership: A survey of public secondary schools
Source: PLoS One. 2026 Aug 14;21(8):e0355674. doi: 10.1371/journal.pone.0355674 (PMC13475869; doi:10.1371/journal.pone.0355674)
Supplement: Supplementary file 12 [file pone.0355674.s010.docx]

Dear Editor,

Thank you very much for your email and for the repeated rounds of constructive feedback on our manuscript. We sincerely appreciate the time, effort, and thoughtful guidance that you and the reviewers have invested in evaluating our work.

We have carefully addressed each comment and made substantial revisions throughout the manuscript. The iterative process of responding to your feedback has significantly strengthened the quality and clarity of our paper.

We believe that the manuscript is now in a publishable form in your esteemed journal. Although if it requires further revisions, we will make further revisions.

Thank you once again for your valuable input and consideration. Please find the comment matrix attached below.

Warm regards,

Authors

| Comment Matrix | | | |
| --- | --- | --- | --- |
| **Serial Number** | **Section where revision has been indicated** | **Revision suggested** | **The section after revision was made according to the reviewers' comments** |
| 1. | Reviewing the Literature | 1. Add references within the last 5 years. | Reference numbers 31, 32, 33, 34 & 35 are added  31. Hallinger, P., Liu, S., Aung, P. N., & Yan, M. (2025). A systematic review of instructional leadership research conducted with the principal instructional Management Rating Scale (PIMRS), 1983–2024. *Educational Management Administration & Leadership*. Advance online publication. https://doi.org/10.1177/17411432251358575  32. Guo, H., Alias, B. S., & Mamud, M. I. (2025). Interplay of principal instructional leadership, school organizational climate, and teacher job satisfaction: Evidence from secondary schools in Northwest China. *International Journal of Learning, Teaching and Educational Research, 24*(4). https://doi.org/10.26803/ijlter.24.4.9  33. Kusumaningrum, D. E., Gunawan, I., Sumarsono, R. B., & Latifah, R. (2025). Exploring how change leadership influences instructional leadership effects on school culture and teachers’ teaching performance in pesantren education settings in Indonesia: A moderated-mediation analysis. *Journal on Efficiency and Responsibility in Education and Science, 18*(2), 100–111. https://doi.org/10.7160/eriesj.2025.180204  34. Dorukbaşi, E., & Cansoy, R. (2024). Examining the mediating role of teacher professional learning between perceived instructional leadership and teacher instructional practices. *European Journal of Education, 59*(3), e12672. https://doi.org/10.1111/ejed.12672  35. Demir, K., & Kalman, M. (2025). Instructional leadership and teachers' instructional practices: A serial mediation by teacher autonomy and collective teacher innovativeness. *European Journal of Education 60*(3), Article e70155. https://doi.org/10.1111/ejed.70155 |
|  | Comment by: Reviewer 5 | | |
| 2. | Implications and Limitations of the study | 2. Add future perspective of the Research. | Since this study is confined to public secondary schools in Kathmandu, such a study could in the future also be conducted in institutional or public schools in other provinces for comparative analysis. Additionally, the effect of instructional leadership on students’ academic achievement can also be studied in different contexts. |
|  | Comment by: Reviewer 5 | | |
| 3. | Result | 3. Tables missing: Tables 1–3 (rotated component matrices) must be included. | Table showing rotated component matrices of all three factors (Factor1, Factor 2, and Factor 3) are there in the result section. |
|  | Comment by: Reviewer 5 | | |
| 4. | Research methods | 4. Delphi clarity: Provide panel size, selection criteria, and consensus definition | The literature lacks consensus on the exact number of experts in the Delphi process [62]. However, following [63], a total of 20 panelists, one teacher, and a head teacher from 10 schools across diverse areas of Kathmandu were selected for the Delphi process. The criteria for their selection as experts in instructional leadership were more than five years of experience in the teaching-learning process in public secondary schools and their professional expertise as head teachers or teachers. The consensus in the Delphi process was measured as percent agreement, and the cut-off value in this study was 75%. It is in line with [62], which, on the basis of a review study, suggested that there is no widely accepted cut-off value (the value from which consensus is assumed) for defining consensus. It varies considerably; however, a threshold of 60% or higher was identified in most cases. |
|  | Comment by: Reviewer 5 | | |
| 5. |  | 5. Data availability: The statement "Data is with authors" does not meet PLOS ONE policy. Deposit data in a public repository or justify restrictions. | Data file is submitted along with other files with the file name S1_dataset. We have no problem to upload data to public repository. Actually, we do not have idea on that online data- storage platform where we should upload that. |
|  | Comment by: Reviewer 5 | | |
| 6. |  | 6. Writing quality: Numerous grammatical errors and typos need correction (e.g., "of of" in title). | Correction is done thoroughly. |
|  | Comment by: Reviewer 5 | | |
| 7. | Discussion | 7. Briefly explain why parental involvement emerged as a factor in Nepal's cultural context. | In Nepal, there is a family-oriented society, and within the family, there is a culture of obedience to parents/guardians. Parents/guardians instill values such as discipline, respect, hard work, and responsibility in their children. Such guidance and care affect children’s learning habits and achievement. Parents/guardians take an interest in monitoring their children’s academic performance and solving their problems. Therefore, parents’/guardians’ involvement emerged as a factor in Nepal’s cultural and societal context. |
|  | Comment by: Reviewer 6 | | |
| 8. | Abstract | 8. 5th sentence in the abstract starts, "It indicated..." What is "it"? Maybe this sentence needs to connect to the previous sentence and combine them for it to make more sense. | The existing instructional leadership models are primarily Western and not entirely suitable for implementation in Nepal. Therefore, it seemed essential to develop a model by identifying the determinants of instructional leadership in Nepal’s local context. |
|  | Comment by: Reviewer 6 | | |
| 9. |  | 9. I see "Nepali Government" and "Nepal Government". Please choose one and stick with it. | It is made Nepal Government. |
|  | Comment by: Reviewer 6 | | |
| 10. | Literature Review | 10. Pg. 7 shows an "r-cal" statistic. What is "r-cal"? It can't be a Pearson Correlation. Is it a t-statistic? Is it supposed to be "r=0.344" instead of 3.44? | Yes, these are t-values and could correspond to significant correlations at p < 0.05. We removed values from the sentence because the statement gives complete sense even without mentioning these values. |
|  | Comment by: Reviewer 6 | | |
| 11. | Literature Review and Research Methods | 11. Literature Review and Research Methods could use headings to break them down. Right now, they read a bit disjointed as paragraphs run into each other. For example, the Ethical Standards paragraph comes out of nowhere. A heading would help readability. | Sub- headings are added in both literature review, and research methods sections. |
|  | Comment by: Reviewer 6 | | |
| 12. |  | 12. The last three paragraphs of the Literature Review begin explaining the survey measures and the methods. Those belong in the methods. Maybe a "Measures" section in the methods is where this could be situated. | Arranged accordingly. |
|  | Comment by: Reviewer 6 | | |
| 13. |  | 13. On P. 10, second paragraph - the first and last sentences aren't necessary as they don't add anything substantive. | Those two sentences are removed. |
|  | Comment by: Reviewer 6 | | |
| 14. |  | 14. In Table 2, "SN" and "Items" run together. There appears to be room to not have to abbreviate "SN", but I could be wrong. | It has been corrected. |
|  | Comment by: Reviewer 6 | | |
| 15. |  | 15. P.15, "[69, 70, 71, 72]" is missing a period. | Full stop is added. |
