# Supplementary material for: Determinants of instructional leadership: A survey of public secondary schools
Source: PLoS One. 2026 Aug 14;21(8):e0355674. doi: 10.1371/journal.pone.0355674 (PMC13475869; doi:10.1371/journal.pone.0355674)
Supplement: Peer review history

# Peer review: Decision Letter 0

**DOI:** 10.1371/journal.pone.0355674.r001

**Authors:** Muhammad Zammad Aslam

27 Jul 2025

Dear Dr. Pande,

Thank you for submitting your manuscript to PLOS ONE. After careful consideration, we feel that it has merit but does not fully meet PLOS ONE’s publication criteria as it currently stands. Therefore, we invite you to submit a revised version of the manuscript that addresses the points raised during the review process.

Please submit your revised manuscript by Sep 10 2025 11:59PM. If you will need more time than this to complete your revisions, please reply to this message or contact the journal office at plosone@plos.org. When you're ready to submit your revision, log on to https://www.editorialmanager.com/pone/ and select the 'Submissions Needing Revision' folder to locate your manuscript file.

- A rebuttal letter that responds to each point raised by the academic editor and reviewer(s). You should upload this letter as a separate file labeled 'Response to Reviewers'.
- A marked-up copy of your manuscript that highlights changes made to the original version. You should upload this as a separate file labeled 'Revised Manuscript with Track Changes'.
- An unmarked version of your revised paper without tracked changes. You should upload this as a separate file labeled 'Manuscript'.

If you would like to make changes to your financial disclosure, please include your updated statement in your cover letter. Guidelines for resubmitting your figure files are available below the reviewer comments at the end of this letter.

If applicable, we recommend that you deposit your laboratory protocols in protocols.io to enhance the reproducibility of your results. Protocols.io assigns your protocol its own identifier (DOI) so that it can be cited independently in the future. For instructions see: https://journals.plos.org/plosone/s/submission-guidelines#loc-laboratory-protocols. Additionally, PLOS ONE offers an option for publishing peer-reviewed Lab Protocol articles, which describe protocols hosted on protocols.io. Read more information on sharing protocols at https://plos.org/protocols?utm_medium=editorial-email&utm_source=authorletters&utm_campaign=protocols.

We look forward to receiving your revised manuscript.

Kind regards,

Muhammad Zammad Aslam, Ph.D.

Academic Editor

PLOS ONE

Journal Requirements:

When submitting your revision, we need you to address these additional requirements.

1. Please ensure that your manuscript meets PLOS ONE's style requirements, including those for file naming. The PLOS ONE style templates can be found at

https://journals.plos.org/plosone/s/file?id=wjVg/PLOSOne_formatting_sample_main_body.pdf and

https://journals.plos.org/plosone/s/file?id=ba62/PLOSOne_formatting_sample_title_authors_affiliations.pdf

2. In the online submission form, you indicated that [Data is with the authors and can be provided if necessary].

All PLOS journals now require all data underlying the findings described in their manuscript to be freely available to other researchers, either 1. In a public repository, 2. Within the manuscript itself, or 3. Uploaded as supplementary information.

This policy applies to all data except where public deposition would breach compliance with the protocol approved by your research ethics board. If your data cannot be made publicly available for ethical or legal reasons (e.g., public availability would compromise patient privacy), please explain your reasons on resubmission and your exemption request will be escalated for approval.

3. When completing the data availability statement of the submission form, you indicated that you will make your data available on acceptance. We strongly recommend all authors decide on a data sharing plan before acceptance, as the process can be lengthy and hold up publication timelines. Please note that, though access restrictions are acceptable now, your entire data will need to be made freely accessible if your manuscript is accepted for publication. This policy applies to all data except where public deposition would breach compliance with the protocol approved by your research ethics board. If you are unable to adhere to our open data policy, please kindly revise your statement to explain your reasoning and we will seek the editor's input on an exemption. Please be assured that, once you have provided your new statement, the assessment of your exemption will not hold up the peer review process.

4. Please include captions for your Supporting Information files at the end of your manuscript, and update any in-text citations to match accordingly. Please see our Supporting Information guidelines for more information: http://journals.plos.org/plosone/s/supporting-information.

5. If the reviewer comments include a recommendation to cite specific previously published works, please review and evaluate these publications to determine whether they are relevant and should be cited. There is no requirement to cite these works unless the editor has indicated otherwise.

[Note: HTML markup is below. Please do not edit.]

Reviewers' comments:

Reviewer's Responses to Questions

**Comments to the Author**

1. Is the manuscript technically sound, and do the data support the conclusions?

Reviewer #1: Partly

Reviewer #2: Partly

Reviewer #3: Yes

**********

2. Has the statistical analysis been performed appropriately and rigorously?

Reviewer #1: No

Reviewer #2: Yes

Reviewer #3: No

**********

3. Have the authors made all data underlying the findings in their manuscript fully available?

The PLOS Data policy

Reviewer #1: No

Reviewer #2: No

Reviewer #3: Yes

**********

4. Is the manuscript presented in an intelligible fashion and written in standard English?

Reviewer #1: No

Reviewer #2: Yes

Reviewer #3: No

**********

Reviewer #1: The manuscript presents a quantitative study on the determinants of instructional leadership in public secondary schools in Kathmandu, Nepal, with Hallinger and Murphy’s 1985 model as its theoretical foundation. The research is technically sound and methodologically rigorous, employing a Classical Delphi process to adapt the Principal Instructional Management Rating Scale (PIMRS) to the Nepali context. This adaptation involved expert consultation and the addition and removal of items to ensure cultural and contextual relevance. Data were collected from 172 randomly selected schools in Kathmandu Valley, with both head teachers and teachers participating as respondents. The study design, sampling, and data collection procedures are clearly described, and ethical standards were maintained throughout, including prior approval from the university’s research committee and informed consent from all participants.

The statistical analysis is appropriate and robust. The study uses exploratory factor analysis (EFA) to identify the underlying factors of instructional leadership, with all necessary conditions for factor analysis thoroughly checked and met. The Kaiser-Meyer-Olkin measure of sampling adequacy is 0.896, indicating excellent suitability for factor analysis, and Bartlett’s test of sphericity is significant. The sample size and variable ratio are within recommended guidelines, and factor loadings, eigenvalues, and communalities all meet accepted thresholds. Cronbach’s alpha values for the identified factors range from 0.75 to 0.92, demonstrating strong internal consistency and reliability. The analysis results in three main factors: planning, managing, and supervising instructional programs; motivating and developing competence of teachers and students; and involving parents. These factors are well supported by both the data and the literature, and the discussion contextualizes the findings within existing research, highlighting the unique emergence of parental involvement as a core factor in the Nepali context.

Regarding data availability, the manuscript states that data are with the authors and can be provided if necessary. However, this does not fully comply with PLOS ONE’s data policy, which requires that all data underlying the findings be made publicly available without restriction. The authors should deposit the anonymized dataset in a public repository or provide it as supplementary information to ensure compliance with journal requirements.

The manuscript is generally intelligible and written in standard English, though there are occasional grammatical errors and redundancies, particularly in the literature review and policy background sections. The structure of the results section could be improved with clearer subheadings, and some sentences would benefit from greater clarity and conciseness. Terms such as “post-positivist paradigm” should be defined for broader accessibility. Overall, the manuscript communicates its findings effectively, but a thorough language and style revision is recommended to address minor issues and improve readability.

Reviewer #2: The authors should provide the outputs of the factor analysis, which was completed using the Statistical Package for Social Sciences 29.0. Based on the information provided regarding the factor loadings of the questionnaire items, reviewers need to verify the factor loadings outputs to ensure the data analysis's accuracy.

Reviewer #3: Dear author,

Thank you for the opportunity to review this manuscript. Below are my comments on my review:

1. The topic addressed in the article is relevant for publication in the journal. However, it requires significant revisions, which are mentioned in this review.

2. The title clearly reflects the topic addressed in the article. It is well-defined and clearly defines the subject matter of the manuscript.

3. The abstract and keywords do not provide adequate information about the article. Although the objective and topic addressed in the manuscript are mentioned, the research methodology, main results, and conclusions found in the study are missing.

4. Check whether all authors cited in the article are included in the manuscript's references, and vice versa.

5. The article has issues with clarity and coherence in its language, which need to be revised. The Introduction doesn't clarify the exact objective of the research (it differs from the objective stated in the Abstract) and the central research question. Please review this.

Regarding the methodology, it's necessary to clarify how the questionnaires were designed and administered to the research participants, and how the data analysis and interpretation were performed.

The conclusions lack clarity on whether the central research question was answered, whether the hypotheses were confirmed, and the relevance of the research to the field of study.

6. There is a connection between the theme and the theoretical foundation. However, I don't understand why the author included two literature review topics (Literature Review and Theoretical Foundation), when they are the same thing. It's important to review this so that the topic discussed in this theoretical section isn't redundant and doesn't confuse the reader when reading the research.

7. There are some issues with the data analysis and the coherence of the argument. The following statement is confusing: "Factor analysis resulted in 25 items under three factors. Fourteen of these twenty-five items fall under factor one, eight under factor two, and three under factor three." Please explain better what this means and what you mean by it. Make this information clear to the reader, as well as how you arrived at this conclusion. Furthermore, it is important to review all the variables obtained. Finally, the discussion of the collected data needs to be improved, in dialogue with the scientific literature in the field.

8. The bibliography used in the article is adequate and up-to-date.

This is an interesting study. However, I hope these comments will help improve your manuscript.

**********

what does this mean?). If published, this will include your full peer review and any attached files.

If you choose “no”, your identity will remain anonymous but your review may still be made public.

**Do you want your identity to be public for this peer review?**  For information about this choice, including consent withdrawal, please see our Privacy Policy

Reviewer #1: No

Reviewer #2: **Yes:**  Olugbenga IGE

Reviewer #3: **Yes:**  Gustavo Cunha de Araujo

**********

[NOTE: If reviewer comments were submitted as an attachment file, they will be attached to this email and accessible via the submission site. Please log into your account, locate the manuscript record, and check for the action link "View Attachments". If this link does not appear, there are no attachment files.]

While revising your submission, please upload your figure files to the Preflight Analysis and Conversion Engine (PACE) digital diagnostic tool, https://pacev2.apexcovantage.com/. PACE helps ensure that figures meet PLOS requirements. To use PACE, you must first register as a user. Registration is free. Then, login and navigate to the UPLOAD tab, where you will find detailed instructions on how to use the tool. If you encounter any issues or have any questions when using PACE, please email PLOS at figures@plos.org. Please note that Supporting Information files do not need this step.

Attachment Submitted filename: PONE-D-25-14755_reviewer 21 July 2025.pdf

Attachment Submitted filename: IL_Factors and items.docx

# Author comment: Author response to Decision Letter 1

**DOI:** 10.1371/journal.pone.0355674.r002

11 Sep 2025

Comment Matrix

Comment by: Reviewer 1

S.No. Section where revision has been indicated The section presented before revision was advised Revision suggested The section after revision was made according to the reviewers' comments

1. Literature Review Researchers attempted to examine school effectiveness or program improvement in the late 1970s. They first identified the effective schools and then analyzed what made them effective. They noticed that a skillful head teacher was a key factor in accounting for successful change or school improvement [23]. This effective school research base assigned the role of instructional leader to the head teachers to develop their skills because it was found that a head teacher would seem to be capable of fulfilling the head teacher’s functions of managing the facility, controlling the operation, taking charge of community relations and overseeing the student progress only when he or she also becomes responsible for instructional leadership [24]. As a result, instructional leadership appeared as an essential construct for making schools effective, but it was still unclear what the actual instructional leadership roles are.

Instructional leadership is a term formed by combining "instruction" and "leadership." There have been multiple efforts to define instruction, but four key dimensions of instruction remain consistent: setting learning objectives, choosing teaching methods, selecting reinforcement activities, and evaluating outcomes [25]. Researchers hold varying views on ledership as well. Some have defined leadership as how a leader leads; others have described it as the job done through people [25]. Leadership includes followers acting toward goals that represent the values of the leaders and the followers [26]. Such uncertainty surrounding the concept of leadership has made the combined construct of “instructional leadership” difficult to define and clarify. The early instructional leadership models tended to focus on the leadership portion of the construct, and they attempted to define it in terms of general leadership theory. Those models focused on personality/ leadership style, behavioral/ situational or contextual components of the head teacher [24].

In this manner, the combined construct of "instructional leadership" has been defined in various ways. Instructional leadership means providing the necessary resources, possessing knowledge and skill in curriculum and instructional matters, being a skilled communicator and being a visionary who is out and around creating a visible presence [26]. Instructional leadership is direct assistance to teachers in their day-to-day activities, development of collaborative groups among staff, design and procurement of effective staff development, curriculum development, and the use of action research [27]. These definitions emphasized not only improving the instructional leadership role of the head teacher through the monitoring of instructional activities but also focused on the managerial task of the head teacher within it. Later scholars of instructional leadership began linking it to activities aimed at enhancing teaching and learning, leading to a slight shift in how instructional leadership was defined. Here are some examples. The instructional leadership begins when principals talk about being in the classroom, visit with students about their academic pursuits, and prioritise instruction [28]. Instructional leadership is important for effective practices in curriculum, instruction, and assessment and the ability to work with teachers on the day-to-day problems related to these topics [29]. The latter two perspectives on instructional leadership place a strong emphasis on teaching and learning activities. In this way the concept of instructional leadership evolved through various research developments, ultimately establishing that leadership roles connected to teaching and learning within the school fall under the domain of instructional leadership [30, 31, 2, 32].

The instructional leadership notion has dominated the educational literature since the creation of instructional leadership models, and there is ample proof of its effectiveness regarding teacher and student results [33, 34]. The notable models of instructional leadership have been proposed by various researchers [ e.g., 35, 36, 37, 38, 39, 40, 41]. They have offered a variety of indications of instructional leadership, but the theoretical underpinnings are often the same [40, 41]. Researchers most extensively use the Hallinger and Murphy (1985) model of instructional leadership to do research on instructional leadership. The widespread acceptance of this model is primarily due to Hallinger and Murphy's (1985) core concept, which provides head teachers with valuable insights into all aspects of leadership related to teaching and learning in schools. The main theme of this model is to develop and communicate school goals, coordinate and supervise the school curriculum, monitor and evaluate student progress, encourage teachers and students, and maintain high visibility and a hands-on approach [23, 44]. Their model is in the form of a scale known as PIMRS (Principal/ Head Teacher Instructional Management Rating Scale). This PIMRS has been utilized in at least 130 PhD dissertations over almost three decades [45].

Several academics have mentioned their view on the characteristics of an instructional leader. The instructional leaders perform at high levels in four areas: a) resource provider, b) instructional resource, c) communicator, and d) visible presence in the school [35]. The instructional leaders emphasize on: a) goal establishment curriculum development, b) frequent observations, c) program evaluation, and d) support for teachers [41]. The instructional leader perform seven crucial functions,which are: a) establishing and implementing instructional goals; b) being available to staff; c) creating a school culture and climate conducive to learning; d) communicating the vision and mission of the school to students, staff, and parents; e) setting high expectations for the staff; f) developing teacher leaders; g) maintaining positive attitudes toward student, staff, and parents [39]. There are four tenets of an instructional leader: a) focus on developing, directing, and supervising the curriculum and instruction in the classroom; b) interact with their teachers to improve teaching and learning c) goal-oriented; d) establish high expectations and standards for teachers and students [38]. The effective instructional leader plays five important roles, which are: a) establishing goals and expectations; b) strategic resourcing; c) planning, coordinating, and evaluating teaching and the curriculum; d) promoting and participating in teacher learning and development; and e) ensuring an orderly and supportive environment [40]. There are six characteristics of effective instructional leaders: a) identify and communicate the mission and vision of the school; b) maintain a school environment that is conducive to learning; c) promote the professional development of staff; d) monitor teachers’ instruction; e) maintain a positive school climate and f) initiate organizational change [36]. From these scholars' view it can be inferred that an instructional leader's job is improving instruction to increase student achievement. Hence, an instructional leader is a person who seeks to ensure effective teaching and learning in the school by meeting all the requirements necessary to enhance students' academic performance.

Among the models of instructional leadership proposed by various researchers devoted to the study of instructional leadership, Hallinger and Murphy's (1985) model of instructional leadership was used as a theoretical guide for this research because, this model has attained top priority and is more dominated in the literature of instructional leadership. This model of instructional leadership consists of three factors: defining the school's mission, managing the instructional program, and promoting a positive learning climate. There are two functions under the factor defining the school's mission, which are framing the school goals and communicating the school goals, and three functions under the factor managing the instructional program, which are supervising and evaluating instruction, coordinating curriculum, and monitoring students’ progress, and five functions under the factor promoting a positive learning climate which are protecting instructional time; providing incentives for teachers; providing incentives for learning; promoting professional development of teachers; and maintaining high visibility. There are five items under each of these ten functions, forming fifty variables altogether.

These fifty items were studied thoroughly, and classical Delphi was conducted, followed by three round meetings with experienced head teachers and teachers to get their concrete ideas on it. Based on their opinion, some items, for example, (i) draw upon the results of class wise testing when making curriculum decisions, (ii) participate actively in the review of curricular materials, (iii) acknowledge teachers’ exceptional performance by writing memos for their personal files, (iv) recognize superior students’ achievement or improvement by seeing in the office the students with their work, (v) review student work products when evaluating classroom instruction were unselected from this PIMRS. Some items, for example, (i) head teacher announces mandatory presence of parents during terminal report card distribution, (ii) head teacher meets the parents of poor-performing students and shares with them what roles the school and parents may together take for their improvements, (iii) head teacher learns students’ family problems and talks to their parents to resolve them, (iv) head teacher is transparent about every financial detail of the school to all stakeholders were added in new PIMRS.

Some items were changed to best suit in Nepali schools, such as (i) monitoring the classroom curriculum to see that it covers the school's curricular objectives was changed to the head teacher checking teachers' log books regularly to see if they are going in accordance with the syllabus, (ii) limit interruption of instructional time by public address announcements was changed to head teacher tries his best to ensure uninterrupted instruction time, (iv) lead or attend teacher in- service activities concerned with instruction was changed to head teacher seeks to provide training to teachers during vacations or so as not to interrupt daily classes, (v) conduct informal observations in classrooms regularly was changed to head teacher regularly monitors activities of teachers and students while they are in class. In this way, finally a new PIMRS was developed for this research. This PIMRS consists of 11 factors and 33 items.

The manuscript is generally intelligible and written in standard English, though there are occasional grammatical errors and redundancies, particularly in the literature review and policy background sections Researchers began examining school effectiveness and program improvement in the late 1970s. They first identified effective schools and then analyzed what made them successful, noting that a skillful head teacher was a key factor in driving school improvement [23]. It was also emphasized that instructional leadership plays a pivotal role in fostering school improvement, promoting better academic outcomes both directly, by supporting teachers, and indirectly, by improving conditions for teaching and learning. Successful school leadership involves improving conditions for teaching and learning, redesigning and enriching the curriculum, redesigning the rolesw and responsibilities, enhancing teacher quality, building relationships inside the school and community, enhancing teaching and learning and building relationship outside the school community [24].

Instructional leadership is a construct derived form the combination of the concepts of "instruction" and "leadership." There have been multiple efforts to define instruction, but four key dimensions of instruction remain consistent: setting learning objectives, choosing teaching methods, selecting reinforcement activities, and evaluating outcomes [25]. Researchers hold varying views on ledership as well. Some have defined leadership as how a leader leads; others have described it as the job done through people [25]. Leadership includes followers acting toward goals that represent the values of the leaders and the followers [26]. Such uncertainty surrounding the concept of leadership has made the combined construct of “instructional leadership” difficult to define and clarify. The early instructional leadership models tended to focus on the leadership portion of the construct, and they attempted to define it in terms of general leadership theory. Those models focused on personality/leadership style, behavioral/situational, or contextual components of the head teacher [24].

In this manner, the combined construct of "instructional leadership" has been defined in various ways. Instructional leadership means providing the necessary resources, possessing knowledge and skill in curriculum and instructional matters, being a skilled communicator, and being a visionary who is out and around creating a visible presence [26]. Instructional leadership is direct assistance to teachers in their day-to-day activities, the development of collaborative groups among staff, the design and procurement of effective staff development, curriculum development, and the use of action research [27]. These definitions emphasized not only improving the instructional leadership role of the head teacher through the monitoring of instructional activities but also focused on the managerial tasks of the head teacher within it. Later scholars of instructional leadership began linking it to activities aimed at enhancing teaching and learning, leading to a slight shift in how instructional leadership was defined. Here are some examples: instructional leadership begins when principals talk about being in the classroom, visit with students about their academic pursuits, and prioritize instruction [28]. Instructional leadership is important for effective practices in curriculum, instruction, and assessment, and for the ability to work with teachers on the day-to-day problems related to these topics [29]. The latter two perspectives on instructional leadership place a strong emphasis on teaching and learning activities. In this way, the concept of instructional leadership evolved through various research developments, ultimately establishing that leadership roles connected to teaching and learning within the school fall under the domain of instructional leadership [30, 31, 2, 32].

The instructional leadership notion has dominated the educational literature since the creation of instructional leadership models, and there is ample proof of its effectiveness regarding teacher and student results [33, 34]. The notable models of instructional leadership have been proposed by various researchers [ e.g.,35, 36, 37, 38, 39, 40, 41]. They have offered a variety of indications of instructional leadership, but the theoretical underpinnings are often the same [40, 41]. Researchers most extensively use the Hallinger and Murphy (1985) model of instructional leadership to conduct research on instructional leadership. The widespread acceptance of this model is primarily due to Hallinger and Murphy's (1985) core concept, which provides head teachers with valuable insights into all aspects of leadership related to teaching and learning in schools. The main theme of this model is to develop and communicate school goals, coordinate and supervise the school curriculum, monitor and evaluate student progress, encourage teachers and students, and maintain high visibility and a hands-on approach [23, 44]. Their model is in the form of a scale known as PIMRS (Principal/Head Teacher Instructional Management Rating Scale). This PIMRS has been utilized in at least 130 PhD dissertations over almost three decades [45].

Several academics have mentioned their views on the characteristics of an instructional leader. I I

Attachment Submitted filename: S3@IL_ReviewMatrix.docx

# Peer review: Decision Letter 1

**DOI:** 10.1371/journal.pone.0355674.r003

**Authors:** Annesha Sil

9 Oct 2025

Dear Dr. Pande,

Thank you for submitting your manuscript to PLOS ONE. After careful consideration, we feel that it has merit but does not fully meet PLOS ONE’s publication criteria as it currently stands. Therefore, we invite you to submit a revised version of the manuscript that addresses the points raised during the review process.

Please submit your revised manuscript by Nov 23 2025 11:59PM. If you will need more time than this to complete your revisions, please reply to this message or contact the journal office at plosone@plos.org. When you're ready to submit your revision, log on to https://www.editorialmanager.com/pone/ and select the 'Submissions Needing Revision' folder to locate your manuscript file.

- A rebuttal letter that responds to each point raised by the academic editor and reviewer(s). You should upload this letter as a separate file labeled 'Response to Reviewers'.
- A marked-up copy of your manuscript that highlights changes made to the original version. You should upload this as a separate file labeled 'Revised Manuscript with Track Changes'.
- An unmarked version of your revised paper without tracked changes. You should upload this as a separate file labeled 'Manuscript'.

If you would like to make changes to your financial disclosure, please include your updated statement in your cover letter. Guidelines for resubmitting your figure files are available below the reviewer comments at the end of this letter.

If applicable, we recommend that you deposit your laboratory protocols in protocols.io to enhance the reproducibility of your results. Protocols.io assigns your protocol its own identifier (DOI) so that it can be cited independently in the future. For instructions see: https://journals.plos.org/plosone/s/submission-guidelines#loc-laboratory-protocols. Additionally, PLOS ONE offers an option for publishing peer-reviewed Lab Protocol articles, which describe protocols hosted on protocols.io. Read more information on sharing protocols at https://plos.org/protocols?utm_medium=editorial-email&utm_source=authorletters&utm_campaign=protocols.

We look forward to receiving your revised manuscript.

Kind regards,

Annesha Sil, Ph.D.

Staff Editor

PLOS ONE

Journal Requirements:

If the reviewer comments include a recommendation to cite specific previously published works, please review and evaluate these publications to determine whether they are relevant and should be cited. There is no requirement to cite these works unless the editor has indicated otherwise.

[Note: HTML markup is below. Please do not edit.]

Reviewers' comments:

Reviewer's Responses to Questions

**Comments to the Author**

Reviewer #1: All comments have been addressed

Reviewer #2: (No Response)

**********

2. Is the manuscript technically sound, and do the data support the conclusions?

Reviewer #1: Yes

Reviewer #2: Partly

**********

3. Has the statistical analysis been performed appropriately and rigorously?

Reviewer #1: Yes

Reviewer #2: Yes

**********

4. Have the authors made all data underlying the findings in their manuscript fully available?

The PLOS Data policy

Reviewer #1: Yes

Reviewer #2: Yes

**********

5. Is the manuscript presented in an intelligible fashion and written in standard English?

Reviewer #1: Yes

Reviewer #2: No

**********

Reviewer #1: Thank you very much for taking my recommendations into consideration. I truly appreciate your attention to my suggestions, and I am pleased to see that you value and incorporate my feedback in this important process.

Reviewer #2: The literature review section must be strengthened with current literature on instructional leadership. Additionally, the definition or explanation of what constitutes should be reexamined, focusing on three countries or contexts before discussing what constitutes instructional leadership in Nepal. The citation "Hallinger, P., & Murphy J. (1985). Assessing the instructional management behavior of

principals. Elementary School Journal, 86(2), 217-248. https://doi.org/10.1086/461445" is stale in its contribution to knowledge in the field of instructional leadership.

The authors should note that you cannot complete a research project on "Instructional Leadership", and leave out the contributions of Lees Shulman and other scholars in the United States. I strongly suggest the literature review in this manuscript should discuss the concept of instructional leadership in the United States.

In the section on research methods, please contextualize this assertion within a research paradigm, i.e., "i.e. Therefore, it follows the postpositivistic deterministic philosophy of causes determine effects", colleagues! In this sentence, "The research design is quantitative, and research methods include the preparation of questionnaires, data collection, data analysis, and interpretation," please state the research design explicitly. What was the research architect's blueprint that you used in constructing the study reported in the research manuscript, colleagues? You may wish to refer to current literature on "Research Designs."

Please consult the second attachment provided to format a table and report the necessary coding and demographic information of the selected respondents to enable check for technical consistencies of the information provided in methodology and results section of the research manuscript.

Most importantly, the supervisors must support the PhD candidate in addressing the comments that I have provided in the research manuscript and review comments to the author. The suggested corrections require a higher level of academic expertise that the PhD, who is the corresponding author, can handle until he gains further research experience.

**********

what does this mean?). If published, this will include your full peer review and any attached files.

If you choose “no”, your identity will remain anonymous but your review may still be made public.

**Do you want your identity to be public for this peer review?**  For information about this choice, including consent withdrawal, please see our Privacy Policy

Reviewer #1: No

Reviewer #2: **Yes:**  Olugbenga IGE

**********

[NOTE: If reviewer comments were submitted as an attachment file, they will be attached to this email and accessible via the submission site. Please log into your account, locate the manuscript record, and check for the action link "View Attachments". If this link does not appear, there are no attachment files.]

While revising your submission, please upload your figure files to the Preflight Analysis and Conversion Engine (PACE) digital diagnostic tool, https://pacev2.apexcovantage.com/. PACE helps ensure that figures meet PLOS requirements. To use PACE, you must first register as a user. Registration is free. Then, login and navigate to the UPLOAD tab, where you will find detailed instructions on how to use the tool. If you encounter any issues or have any questions when using PACE, please email PLOS at figures@plos.org. Please note that Supporting Information files do not need this step.

Attachment Submitted filename: PONE-D-25-14755_R1 8 October 2025.pdf

Attachment Submitted filename: SKM_C300i25100810250.pdf

# Author comment: Author response to Decision Letter 2

**DOI:** 10.1371/journal.pone.0355674.r004

2 Dec 2025

Comment Matrix

Comment by: Reviewer 2

S.No. Section where revision has been indicated The section presented before revision was advised Revision suggested The section after revision was made according to the reviewers' comments

1. Literature Review The researchers began examining school effectiveness and program improvement in the late 1970s. They first identified effective schools and then analyzed what made them successful, noting that a skillful head teacher was a key factor in driving school improvement [23]. It was emphasized that instructional leadership plays a pivotal role in fostering school improvement, promoting better academic outcomes both directly, by supporting teachers, and indirectly, by improving conditions for teaching and learning. Successful school leadership also involves enriching the curriculum, redefining the roles and responsibilities, enhancing teacher quality, and building strong relationships within the school and with the wider community [24].

Instructional leadership is a construct derived from the combination of the concepts of "instruction" and "leadership." There have been multiple efforts to define instruction, but four key dimensions of instruction remain consistent: setting learning objectives, choosing teaching methods, selecting reinforcement activities, and evaluating outcomes [25]. Researchers hold varying views on leadership as well. Some have defined leadership as how a leader leads; others have described it as the job done through people [25]. Leadership includes followers acting toward goals that represent the values of the leaders and the followers [26]. Such uncertainty surrounding the concept of leadership has made the combined construct of “instructional leadership” difficult to define and clarify. The early instructional leadership models tended to focus on the leadership portion of the construct, and they attempted to define it in terms of general leadership theory. Those models focused on personality/leadership style, behavioral/situational, or contextual components of the head teacher [24].

In this manner, the combined construct of "instructional leadership" has been defined in various ways. Instructional leadership means providing the necessary resources, possessing knowledge and skill in curriculum and instructional matters, being a skilled communicator, and being a visionary who is out and around creating a visible presence [26]. Instructional leadership is direct assistance to teachers in their day-to-day activities, the development of collaborative groups among staff, the design and procurement of effective staff development, curriculum development, and the use of action research [27]. These definitions emphasized not only improving the instructional leadership role of the head teacher through the monitoring of instructional activities, but also focused on the managerial tasks of the head teacher within it. Later scholars of instructional leadership began linking it to activities aimed at enhancing teaching and learning, leading to a slight shift in how instructional leadership was defined. Here are some examples: instructional leadership begins when principals talk about being in the classroom, visit with students about their academic pursuits, and prioritize instruction [28]. Instructional leadership is important for effective practices in curriculum, instruction, and assessment, and for the ability to work with teachers on the day-to-day problems related to these topics [29]. The latter two perspectives on instructional leadership place a strong emphasis on teaching and learning activities. In this way, the concept of instructional leadership evolved through various research developments, ultimately establishing that leadership roles connected to teaching and learning within the school fall under the domain of instructional leadership [30, 31, 2, 32].

The instructional leadership notion has dominated the educational literature since the creation of instructional leadership models, and there is ample proof of its effectiveness regarding teacher and student results [33, 34]. The notable models of instructional leadership have been proposed by various researchers [ e.g.,35, 36, 37, 38, 39, 40, 41]. They have offered a variety of indications of instructional leadership, but the theoretical underpinnings are often the same [40, 41]. Researchers most extensively use the Hallinger and Murphy (1985) model of instructional leadership to research instructional leadership. The widespread acceptance of this model is primarily due to Hallinger and Murphy's (1985) core concept, which provides head teachers with valuable insights into all aspects of leadership related to teaching and learning in schools. The main theme of this model is to develop and communicate school goals, coordinate and supervise the school curriculum, monitor and evaluate student progress, encourage teachers and students, and maintain high visibility and a hands-on approach [23, 44]. Their model is in the form of a scale known as PIMRS (Principal/Head Teacher Instructional Management Rating Scale). This PIMRS has been utilized in at least 130 PhD dissertations over almost three decades [45].

Several academics have mentioned their views on the characteristics of an instructional leader. Instructional leaders perform at high levels in four areas: a) resource provider, b) instructional resource, c) communicator, and d) visible presence in the school [35]. The instructional leaders emphasize: a) goal establishment and curriculum development, b) frequent observations, c) program evaluation, and d) support for teachers [41]. Instructional leaders perform seven crucial functions, which are: a) establishing and implementing instructional goals; b) being available to staff; c) creating a school culture and climate conducive to learning; d) communicating the vision and mission of the school to students, staff, and parents; e) setting high expectations for the staff; f) developing teacher leaders; g) maintaining positive attitudes toward student, staff, and parents [39]. There are four tenets of an instructional leader: a) focus on developing, directing, and supervising the curriculum and instruction in the classroom; b) interacting with teachers to improve teaching and learning c) being goal-oriented; d) establishing high expectations and standards for teachers and students [38]. The effective instructional leader plays five important roles, which are: a) establishing goals and expectations; b) strategic resourcing; c) planning, coordinating, and evaluating teaching and the curriculum; d) promoting and participating in teacher learning and development; and e) ensuring an orderly and supportive environment [40]. There are six characteristics of effective instructional leaders: a) identifying and communicating the mission and vision of the school; b) maintaining a school environment that is conducive to learning; c) promoting the professional development of staff; d) monitoring teachers’ instruction; e) maintaining a positive school climate; and f) initiating organizational change [36]. From these scholars' views, it can be inferred that an instructional leader's job is to improve instruction to increase student achievement. Hence, an instructional leader is a person who seeks to ensure effective teaching and learning in the school by meeting all the requirements necessary to enhance students' academic performance.

Among the models of instructional leadership proposed by various researchers devoted to the study of instructional leadership, Hallinger and Murphy's (1985) model of instructional leadership was used as a theoretical guide for this research because this model has attained top priority and is more dominant in the literature on instructional leadership. This model of instructional leadership consists of three factors: defining the school's mission, managing the instructional program, and promoting a positive learning climate. There are two functions under the factor defining the school's mission, which are framing the school goals and communicating the school goals; three functions under the factor managing the instructional program, which are supervising and evaluating instruction, coordinating curriculum, and monitoring students’ progress; and five functions under the factor promoting a positive learning climate which are protecting instructional time, providing incentives for teachers, providing incentives for learning, promoting professional development of teachers, and maintaining high visibility. There are five items under each of these ten functions, forming fifty variables altogether.

These fifty items were studied thoroughly, and a classical Delphi was conducted, followed by three rounds of meetings with experienced head teachers and teachers to get their concrete ideas on them. Based on their opinions, some items- for example, (i) draw upon the results of class-wise testing when making curriculum decisions, (ii) participate actively in the review of curricular materials, (iii) acknowledge teachers’ exceptional performance by writing memos for their personal files, (iv) recognize superior students’ achievement or improvement by seeing in the office the students with their work, (v) review student work products when evaluating classroom instruction- were deselected from this PIMRS. Some items- for example, (i) head teacher announces mandatory presence of parents during terminal report card distribution, (ii) head teacher meets the parents of poor-performing students and shares with them what roles the school and parents may together take for their improvements, (iii) head teacher learns students’ family problems and talks to their parents to resolve them, (iv) the head teacher is transparent about every financial detail of the school to all stakeholders- were added to the in new PIMRS.

Some items were changed to best suit Nepali schools, such as (i) monitoring the classroom curriculum to see that it covers the school's curricular objectives was changed to the head teacher checking teachers' log books regularly to see if they are going in accordance with the syllabus, (ii) limiting interruption of instructional time by public address announcements was changed to head teacher trying his best to ensure uninterrupted instructional time, (iii) leading or attending teacher in- service activities concerned with instruction was changed to head teacher seeking to provide training to teachers during vacations so as not to interrupt daily classes, (v) conducting informal observations in classrooms regularly was changed to head teacher regularly monitoring the activities of teachers and students while they are in class. In this way, a new PIMRS was finally developed for this research. This PIMRS consists of 11 factors and 33 items.

The literature review section must be strengthened with current literature on instructional leadership. Additionally, the definition or explanation of what constitutes should be reexamined, focusing on three countries or contexts before discussing what constitutes instructional leadership in Nepal. The citation "Hallinger, P., & Murphy J. (1985). Assessing the instructional management behavior of

principals. Elementary School Journal, 86(2), 217-248. https://doi.org/10.1086/461445" is stale in its contribution to knowledge in the field of instructional leadership.

The authors should note that you cannot complete a research project on "Instructional Leadership", and leave out the contributions of Lees Shulman and other scholars in the United States. I strongly suggest the literature review in this manuscript should discuss the concept of instructional leadership in the United States.

Reviewing the Literature:

Instructional leadership is one of the leadership approaches that has gained steadily increasing importance within the overall role of school principals over the past six decades [23]. This growing emphasis arises from evidence demonstrating its substantial impact on student achievement. For instance, a meta-analysis by [24] found that the average effect of instructional leadership on student outcomes was three to four times greater than that of transformational leadership. A few years later, a study of elementary schools in the USA [25] yielded a similar result, showing that instructional leadership accounted for more variance in student outcomes than transformational leadership. Similarly, a study of Malaysian secondary schools by [26] revealed a positive correlation between the level of instructional leadership and students’ academic achievement, suggesting that the instructional leadership role is crucial in achieving better academic outcomes in schools.

Similarly, a meta-analysis conducted in 2017 that included 14 studies from the past decade [27] found a positive effect of instructional leadership on students’ academic outcomes, along with transformational leadership and distributed leadership. This study confirmed the argument that the more leaders are engaged in the business of teaching and learning with their students, the better the academic performance of students. Similarly, a quantitative study in Indonesia by [28] found that instructional leadership is effective in enhancing the quality of learning. In line with previous findings, a recent meta-analysis conducted in 2025 by [29] revealed that the relationships between principal leadership and student achievement have been more frequently investigated through supportive, instructional, distributed, and transformational leadership approaches (23.64%, 21.82%, and 20.00%), respectively. These findings suggest that the relationship between educational leadership and student achievement remains one of the most widely researched topics, with instructional leadership being a major type of leadership studied. Moreover, more than half of the studies included in the meta-analysis were conducted in the USA, indicating that such research is predominantly produced in that context. Further evidence of the prominence of instructional leadership research in the United States is demonstrated by the studies included in Hallinger’s [30] review, which identified 130 doctoral dissertations employing the Hallinger and Murphy instructional leadership model (PIMRS), produced by doctoral students from more than 85 American universities.

The concept of instructional leadership has been defined variously. According to [31], the pioneering scholars who initially formulated a clear understanding of instructional leadership, focusing on U.S. schools in the 1980s and 1990s, identified the principal’s role in aligning school goals, instructional practices, and student outcomes through both direct and indirect management activities. Their framework has been applied internationally. It involves defining the school’s mission, managing the instructional program, and promoting a favorable learning climate to enhance teaching and learning. This framework illustrates that instructional leaders influence classroom practices not only through direct supervision and feedback but also by developing and enforcing school-wide policies, expectations, and structures that guide teacher and student behavior toward common educational goals. However, the central concept of instructional leadership is the leadership role that focuses on teaching and learning activities with the goal of enhancing students’ academic achievement. Various educators have interpreted this concept in different ways according to their own perspectives. A Canadian researcher [32] portrayed instructional leadership as an active role in the classroom practice based on high levels of pedagogical expertise. Similarly, a researcher from Malaysia [26] defined it as leadership focused on teaching and learning activities, fostering interactions between teachers and students centered on the curriculum. Likewise, researchers from the UK [33] emphasized the importance of setting clear educational objectives, planning the curriculum, and evaluating teachers and their educational practices.

Among all, researchers from America have made significant contributions to the study of instructional leadership. They have provided various interpretations of how effective schools develop through instructional lea

Attachment Submitted filename: Response to Reviewers.docx

# Peer review: Decision Letter 2

**DOI:** 10.1371/journal.pone.0355674.r005

**Authors:** Bekalu Tadesse Moges

19 Jan 2026

Thank you for submitting your manuscript to PLOS ONE. After careful consideration, we feel that it has merit but does not fully meet PLOS ONE’s publication criteria as it currently stands. Therefore, we invite you to submit a revised version of the manuscript that addresses the points raised during the review process.

Abstract

- Problematize, as an entry to the abstract, the need to identify factors influencing instructional leadership in Kathmandu, Nepal.
- Mention the design of the study in the abstract

Methodology

- The fact that “it follows the post-positivistic deterministic philosophy that causes determine effects or outcomes” does not mean it establishes cause effect relationship. Such relationships are only determined by the design employed.
- Mention the variables by name “a causal relationship exists between independent variables (factors) and dependent variable (instructional leadership)”
- Specify the design of the study. the statement “The research design is quantitative, and the research methods include preparing questionnaires, collecting data, analyzing data, and interpreting the results” is not sufficient. There are so many types of quantitative designs.
- Bear in mind that if you stick to establishing cause effect relationships, you need to employ experimental, quasi experimental or longitudinal designs.

Results

- While the problem statement and gap identification in the introduction section is convincing in that student academic performance is still low, the results do not show that the factored dimensions of instructional leadership relate to improving/influencing student performance. As it stands, providing a three-factor solution to instructional leadership through exploratory factor analysis would not help unless the factors show some influence on student performance.

Please submit your revised manuscript by Mar 05 2026 11:59PM. If you will need more time than this to complete your revisions, please reply to this message or contact the journal office at plosone@plos.org. When you're ready to submit your revision, log on to https://www.editorialmanager.com/pone/ and select the 'Submissions Needing Revision' folder to locate your manuscript file.

- A letter that responds to each point raised by the academic editor and reviewer(s). You should upload this letter as a separate file labeled 'Response to Reviewers'.
- A marked-up copy of your manuscript that highlights changes made to the original version. You should upload this as a separate file labeled 'Revised Manuscript with Track Changes'.
- An unmarked version of your revised paper without tracked changes. You should upload this as a separate file labeled 'Manuscript'.

If you would like to make changes to your financial disclosure, please include your updated statement in your cover letter. Guidelines for resubmitting your figure files are available below the reviewer comments at the end of this letter.

If applicable, we recommend that you deposit your laboratory protocols in protocols.io to enhance the reproducibility of your results. Protocols.io assigns your protocol its own identifier (DOI) so that it can be cited independently in the future. For instructions see: https://journals.plos.org/plosone/s/submission-guidelines#loc-laboratory-protocols. Additionally, PLOS ONE offers an option for publishing peer-reviewed Lab Protocol articles, which describe protocols hosted on protocols.io. Read more information on sharing protocols at https://plos.org/protocols?utm_medium=editorial-email&utm_source=authorletters&utm_campaign=protocols.

We look forward to receiving your revised manuscript.

Kind regards,

Bekalu Tadesse Moges

Academic Editor

PLOS One

Journal Requirements:

If the reviewer comments include a recommendation to cite specific previously published works, please review and evaluate these publications to determine whether they are relevant and should be cited. There is no requirement to cite these works unless the editor has indicated otherwise.

[Note: HTML markup is below. Please do not edit.]

Reviewers' comments:

Reviewer's Responses to Questions

**Comments to the Author**

Reviewer #1: All comments have been addressed

Reviewer #4: (No Response)

**********

2. Is the manuscript technically sound, and do the data support the conclusions?

Reviewer #1: Yes

Reviewer #4: Partly

**********

3. Has the statistical analysis been performed appropriately and rigorously?

Reviewer #1: Yes

Reviewer #4: Yes

**********

4. Have the authors made all data underlying the findings in their manuscript fully available?

The PLOS Data policy

Reviewer #1: Yes

Reviewer #4: No

**********

5. Is the manuscript presented in an intelligible fashion and written in standard English?

Reviewer #1: Yes

Reviewer #4: Yes

**********

Reviewer #1: I believe all the suggestions have been implemented, and after reading the new version, I recommend its publication.

Reviewer #4: Thank you for the opportunity to review this manuscript. The focus of the study examines instructional leadership in public secondary schools in Kathmandu, Nepal, using a Delphi-adapted PIMRS instrument and exploratory factor analysis. Overall, the manuscript has improved, particularly in the literature review, theoretical grounding, and clarity around the research paradigm. The topic is relevant, the research question is straightforward, and the study contributes valuable insights from a context that remains underrepresented in the instructional leadership literature. However, several technical and policy-related issues remain unresolved before the manuscript can meet PLOS ONE’s publication standards.

The authors have addressed several key points that were raised in earlier rounds. The literature review now engages effectively with international scholarship, including work from the United States and other comparative contexts. Situated as an post-positivist framework, and the cross-sectional survey design is explicit. Also, the rationale for adapting the PIMRS instrument to the Nepali context is convincing. However, not all of the earlier concerns have been fully resolved. In particular, issues related to data availability, statistical reporting, and the use of causal language. Therefore, the manuscript is not ready for acceptance.

The study design is appropriate for identifying the associations or latent dimensions of instructional leadership. The discussion appropriately links the findings to prior research, and the interpretation of the factor structure is overall reasonable. However, the manuscript continues to use causal language ( “determinants” and “cause-and-effect”) that is not supported by the study design. Because this is a nonexperimental, cross-sectional survey using exploratory factor analysis, the findings should be framed in terms of associated factors or structural dimensions rather than causal explanations. The conclusions should be revised accordingly, and the limitations of self-reported and cross-sectional data should be stated more clearly.

Exploratory factor analysis is an appropriate method for the research aims, but the manuscript currently does not provide sufficient statistical detail to allow readers to fully assess or reproduce the analysis. To meet PLOS ONE’s standards for transparency and rigor, the authors should clearly report the following:

• The Kaiser–Meyer–Olkin (KMO) measure

• Bartlett’s test of sphericity

• The factor extraction method used

• The rotation method applied

• The criteria used for factor retention and loading thresholds

• Reliability estimates (e.g., Cronbach’s alpha) for each factor

Including this information will significantly strengthen the Methods section and improve confidence in the findings.

The current Data Availability Statement states that the authors retain the data and can provide it upon request. This does not comply with the PLOS ONE data policy. PLOS requires that all data underlying the findings be made publicly available, either as Supporting Information or through a recognized public repository, unless a clear and justified exception applies.

Before the manuscript can be accepted, the authors must either:

• Deposit an anonymized dataset in a public repository (such as OSF, Zenodo, or Dryad), or

• Please clearly explain and justify any restrictions on data sharing (e.g., due to ethical or third-party constraints).

This issue must be resolved in the next revision of the manuscript.

The manuscript would benefit from more transparent reporting of the respondent characteristics. Including a simple table summarizing key demographic and professional variables (e.g., role, years of experience, gender, school type) would improve transparency, enabling readers to better understand the sample and assess the generalizability of the findings.

The manuscript is generally readable and is written in acceptable scientific English. However, minor grammatical issues, redundancies, and awkward phrasing remain, particularly in the Results and Discussion sections of the text. Since PLOS ONE does not copyedit accepted manuscripts, a careful final language review is recommended.

To concluded, this manuscript shows evident progress and has the potential to meet the PLOS ONE criteria for publication. Acceptance is feasible once the remaining issues related to data availability, statistical reporting, causal language and sample description are addressed. Addressing these issues will enhance the manuscript’s transparency, rigor, and compliance with PLOS ONE standards.

**********

what does this mean?). If published, this will include your full peer review and any attached files.

If you choose “no”, your identity will remain anonymous but your review may still be made public.

**Do you want your identity to be public for this peer review?**  For information about this choice, including consent withdrawal, please see our Privacy Policy

Reviewer #1: No

Reviewer #4: No

**********

[NOTE: If reviewer comments were submitted as an attachment file, they will be attached to this email and accessible via the submission site. Please log into your account, locate the manuscript record, and check for the action link "View Attachments". If this link does not appear, there are no attachment files.]

To ensure your figures meet our technical requirements, please review our figure guidelines: https://journals.plos.org/plosone/s/figures

You may also use PLOS’s free figure tool, NAAS, to help you prepare publication quality figures: https://journals.plos.org/plosone/s/figures#loc-tools-for-figure-preparation.

NAAS will assess whether your figures meet our technical requirements by comparing each figure against our figure specifications.

# Author comment: Author response to Decision Letter 3

**DOI:** 10.1371/journal.pone.0355674.r006

23 Feb 2026

1) Problematize, as an entry to the abstract, the need to identify factors influencing instructional leadership in Kathmandu, Nepal.

2) Mention the design of the study in the abstract

Abstract

Instructional leadership is central to teaching and learning processes and must be undertaken by academic leaders to promote higher levels of student learning. Despite its recognized importance, students' academic performance in public schools in Nepal remains persistently low, raising concern about how instructional leadership is practiced in the schools. There is need for a validated model to implement required instructional leadership practices in the schools. The existing instructional leadership models are largely Western and not completely suitable to implement in the context of Nepal. It indicated for the need of developing a model by finding out the determining factors of instructional leadership in the local context of Nepal. The Kathmandu, where the largest concentration of public schools in the country is located, represents a critical context for examining instructional leadership practices. Given this, the present study examined the determinants of instructional leadership in Kathmandu, Nepal. The study employed a quantitative, non-experimental, cross-sectional research design to examine those factors.

The framework for this study is grounded in Hallinger and Murphy’s (1985) model of instructional leadership, which serves as the conceptual basis. Using the Classical Delphi method, the model was adapted to develop a measurement scale. At the same time, the study was situated within a post-positivist paradigm, considering that the reality of instructional leadership exists independently. Exploratory factor analysis was then conducted to extract the underlying factors. The findings reveal three major factors influencing instructional leadership: (a) planning, managing, and supervising instructional programs; (b) motivating and developing the competence of teachers and students; and (c) involving parents in the learning process. The inclusion of parental involvement emerged as a novel dimension in Nepal. These results provide meaningful insights for school headteachers, other educational stakeholders, government policymakers, and international researchers interested in instructional leadership.

Comment by: Reviewer

1) The fact that “it follows the post-positivistic deterministic philosophy that causes determine effects or outcomes” does not mean it establishes cause effect relationship. Such relationships are only determined by the design employed.

2) Mention the variables by name “a causal relationship exists between independent variables (factors) and dependent variable (instructional leadership)”

3) Specify the design of the study. the statement “The research design is quantitative, and the research methods include preparing questionnaires, collecting data, analyzing data, and interpreting the results” is not sufficient. There are so many types of quantitative designs.

4) Bear in mind that if you stick to establishing cause effect relationships, you need to employ experimental, quasi experimental or longitudinal designs.

Research Methods

This study aims to identify the determining factors of instructional leadership using a quantitative research approach. Consistent with the assumptions typically underpinning quantitative research [53], this study is situated within post-positivist philosophical paradigm. Post-positivists explore a single reality that is previously unknown [ 54]. They assume that reality exists independently, which can be approximated through careful observations and measurements of those variables [ 55]. Therefore, we made a claim that detailed observation and measurement of variables is essential to obtain knowledge [ 55, 56]. Following quantitative research approaches the study employed a non-experimental, cross-sectional survey design, serving as the architectural blueprint for the investigation.

The research design is quantitative, and the research methods include preparing questionnaires, collecting data, analyzing data, and interpreting the results. Classical Delphi was employed in Hallinger and Murphy’s (1985) instructional leadership model, known as the Principal/Head Teacher Instructional Management Rating Scale (PIMRS), to develop a PIMRS suitable for the context of Nepal. A well-structured questionnaire was prepared using the new PIMRS. The questionnaire developed from the new PIMRS with 11 factors and 33 items asked the respondents to rate their view on a 5-point Likert scale with the descriptors: 1= almost never, 2= seldom, 3= sometimes, 4= frequently, and 5= almost always, on which the head teacher was engaged in those particular activities of the school. The data were collected using a survey method, a technique that provides quantitative or numerical descriptions of population trends, attitudes, or opinions by surveying a sample of that population [57].

The population of this study includes all public secondary schools in Kathmandu Valley. Kathmandu Valley was chosen for the study because it has the greatest concentration of public secondary schools compared to any other region in the nation. In total, 303 public secondary schools in the Kathmandu Valley comprised the study population, and 172 schools formed the sample. One hundred seventy-two schools were chosen at random from a pool of 303 to visit to gather data. Data (see S1_dataset in the Appendix) were collected from one teacher and the head teacher, each from 172 schools, to examine the differences in ratings between head teachers and teachers. One teacher from each school was chosen by the lottery method. The demographic and professional variables of respondents are presented at the end of this paper. Factor analysis was conducted on the obtained data to identify the factors affecting head teachers’ instructional leadership (see S2_output@FactorAnalysis in the Appendix). The findings were analyzed and interpreted in the light of the relevant literature.

Ethical standards were maintained throughout the research process, including during data collection. Before initiating the study, ethical approval was obtained from the Research Committee of Kathmandu University, School of Education (Approval No. 181028-1). The recruitment period for the pilot study was from July 24, 2019, to August 24, 2019. Similarly, the recruitment period for the main study began on August 25, 2019, and concluded on February 5, 2020. Informed consent was secured from all participants. Head teachers, who served as respondents, were initially contacted by phone to request their time and contribution. Consent from the teacher respondents was obtained during school visits. A questionnaire with a cover letter was distributed to each respondent. Some respondents provided data on the same day, while others scheduled a later time. Thus, the data was gathered in accordance with established ethical standards.

The pilot testing of the questionnaire was conducted with the assistance of 30 head teachers and teachers from the randomly selected 172 schools. A default sample size of 30 is advised for questionnaire pilot testing [58] . The majority of the items’ mean scores on the questionnaire’s pilot test were determined to be more than four times the standard deviation. A low standard deviation indicates that the majority of the numbers are incredibly close to the average, demonstrating the normal distribution. This ensured the reliability of the questionnaire [59]. The data were collected from 172 public schools in the Kathmandu Valley using this questionnaire, and the data were subjected to factor analysis to determine the factors influencing instructional leadership.

Comment by: Reviewer

1) While the problem statement and gap identification in the introduction section is convincing in that student academic performance is still low, the results do not show that the factored dimensions of instructional leadership relate to improving/influencing student performance. As it stands, providing a three-factor solution to instructional leadership through exploratory factor analysis would not help unless the factors show some influence on student performance. Thank you for your insightful comment. It is important to note that the extracted factors do not directly influence students’ academic performance; rather, they represent key components of instructional leadership that highlight ways it can be strengthened. By effectively implementing these dimensions, school leaders can enhance the overall quality of instructional leadership, which, in turn, contributes to improved student academic outcomes. Specifically, the variables within the three factors—planning, managing, and supervising instructional programs; motivating and developing the competence of teachers and students; and involving parents in the educational process—are closely aligned with academic performance and offer actionable areas for leadership improvement. Based on your advice, this perspective has been incorporated into the discussion, further clarifying the indirect but meaningful pathway through which instructional leadership influences student learning. Thank you once again for your valuable guidance.

Comment by: Reviewer 4

Several technical and policy-related issues remain unresolved before the manuscript can meet PLOS ONE’s publication standards.

Not all of the earlier concerns have been fully resolved. In particular, issues related to data availability, statistical reporting, and the use of causal language. Therefore, the manuscript is not ready for acceptance. Al the files requested by the journal, including the ethical approval file, demographic information of the respondents and questionnaire mentioned file, factor analysis output files, and data files were already submitted while uploading revised manuscript to the system after addressing the previous round comments. However, we are submitting those files once again with the revised manuscript.

The findings should be framed in terms of associated factors or structural dimensions rather than causal explanations. The conclusions should be revised accordingly, and the limitations of self-reported and cross-sectional data should be stated more clearly. Conclusion

In response to the research question, ‘What factors determine head teachers’ instructional leadership?’ the study identified three key factors. The first factor, planning, managing, and supervising instructional programs, explains the head teacher’s role in collaborating with teachers to ensure that the school has a clear mission that focuses on students’ academic achievement. The second factor, motivating and developing the competence of teachers and students, describes the head teacher’s activities to boost the interest of teachers and students in their line of work. The third factor, involving parents, highlights the roles of parents/ guardians in collaboration with the school to support students’ academic progress. The study’s key finding is that parents’ or guardians’ involvement in students’ progress is viewed as an essential factor in schools in Nepal, which differs from Hallinger and Murphy’s (1985) model of instructional leadership. In Hallinger and Murphy’s (1985) instructional leadership model, parents’ or guardians’ involvement is not explicitly recognized as a factor in student success. Instead, the model includes only one related item, contacting parents to communicate improved or exemplary student performance or contributions, which falls under the factor ‘providing incentives for learning’. This suggests that Nepal’s unique cultural and societal framework places greater emphasis on the role of parents/ guardians in students’ academic achievement. By identifying these factors, this study makes a significant contribution to the field of instructional leadership, highlighting the specific requirements for effective teaching and learning in Nepali public schools.

Implications and limitations of the Study

The results of this study can help head teachers stay informed in their role as instructional leaders by providing recommendations for enhancing teaching and learning in schools. The finding can also assist policymakers and bureaucrats in formulating and implementing necessary policies to enhance head teachers’ instructional leadership. Additionally, it can support local authorities in improving schools, as the responsibility for school development is entrusted to local bodies in Nepal’s federal democratic system. In this way, the results of this study can be beneficial for enhancing the effectiveness of schools and achieving quality education. Furthermore, this study has contributed new knowledge to the construct of instructional leadership in the local Nepali context by identifying the factors that influence it, which may be of interest to international researchers in this field.

As the present study employed cross-sectional design, the results may be time specific, limiting their generalizability across periods. Future studies employing longitudinal approaches could allow examination of changes or developmental patterns over time. Future researchers can also employ mixed methods approaches and integrate both subjective perceptions and objective practices associated with instructional leadership. The present non-experimental, cross-sectional study also limited the causal interpretation. Use of an experimental design could provide stronger evidence of causal relationships. However, data triangulation between head teachers and teachers was done, the self-reported data bias may still have occurred. The future researches can be conducted implementing a method which supports for collecting data both from participants and observations or objective records.

Exploratory factor analysis is an appropriate method for the research aims, but the manuscript currently does not provide sufficient statistical detail to allow readers to fully assess or reproduce the analysis. To meet PLOS ONE’s standards for transparency and rigor, the authors should clearly report the following:

• The Kaiser–Meyer–Olkin (KMO) measure

• Bartlett’s test of sphericity

• The factor extraction method used

• The rotation method applied

• The criteria used for factor retention and loading thresholds

• Reliability estimates (e.g., Cronbach’s alpha) for each factor

Including this information will significantly strengthen the Methods section and improve confidence in the findings.

All the conditions required for factor analysis were checked. Factor analysis on Likert scale data is highly valued [60]. The criteria were met as a 5-point Likert scale was used in this study. A lower number of categories cannot provide more possibilities and cannot help obtain more reliable responses; on the other hand, a large range of scale impacts respondents’ responses and reduces their chances of providing the correct answer on a specific item [61]. Therefore, I created a questionnaire using a 5-point Likert scale. The ratio of sample size to variable should be 3 to 6 [62]. Since the sample size is 172 and the number of variables is 39, this ratio falls within the recommended range of 3:1 to 6:1. Exploratory factor analysis was conducted using principal component analysis as the extraction method. Regarding the criteria for factor retention, one of the most common situations is the retention of factors with Eigenvalues greater than one [63, 64]. In this study, all factors had Eigenvalues greater than 1. The rotation method applied was varimax. This study also met the requirement of item loading, with a value of 0.40 to 0.50 [65], as the item loading was 0.50.

Another condition is that the average commonalities or extraction value of items is more than 0.5 [65]. The average extraction value for items was 0.593. Therefore, this requirement was also fulfilled. Another criterion is Kaiser-Meyer-Olkin sampling adequacy. The KMO measure of sampling adequacy was 0.961. If the KMO measure is greater than 0.50, it is safe to assume that the factor ability exists in the dataset [63, 66]. So, this condition was fully satisfied. The Bartle

# Peer review: Decision Letter 3

**DOI:** 10.1371/journal.pone.0355674.r007

**Authors:** Bekalu Tadesse Moges

10 Jun 2026

Dear Dr. Pande,

Please submit your revised manuscript by Jul 25 2026 11:59PM. If you will need more time than this to complete your revisions, please reply to this message or contact the journal office at plosone@plos.org. When you're ready to submit your revision, log on to https://www.editorialmanager.com/pone/ and select the 'Submissions Needing Revision' folder to locate your manuscript file.

- A letter that responds to each point raised by the academic editor and reviewer(s). You should upload this letter as a separate file labeled 'Response to Reviewers'.
- A marked-up copy of your manuscript that highlights changes made to the original version. You should upload this as a separate file labeled 'Revised Manuscript with Track Changes'.
- An unmarked version of your revised paper without tracked changes. You should upload this as a separate file labeled 'Manuscript'.

If you would like to make changes to your financial disclosure, please include your updated statement in your cover letter. Guidelines for resubmitting your figure files are available below the reviewer comments at the end of this letter.

If applicable, we recommend that you deposit your laboratory protocols in protocols.io to enhance the reproducibility of your results. Protocols.io assigns your protocol its own identifier (DOI) so that it can be cited independently in the future. For instructions see: https://journals.plos.org/plosone/s/submission-guidelines#loc-laboratory-protocols. Additionally, PLOS ONE offers an option for publishing peer-reviewed Lab Protocol articles, which describe protocols hosted on protocols.io. Read more information on sharing protocols at https://plos.org/protocols?utm_medium=editorial-email&utm_source=authorletters&utm_campaign=protocols.

As the corresponding author, your ORCID iD is verified in the submission system and will appear in the published article. PLOS supports the use of ORCID, and we encourage all coauthors to register for an ORCID iD and use it as well. Please encourage your coauthors to verify their ORCID iD within the submission system before final acceptance, as unverified ORCID iDs will not appear in the published article. *Only*  the individual author can complete the verification step; PLOS staff *cannot*  verify ORCID iDs on behalf of authors.

We look forward to receiving your revised manuscript.

Kind regards,

Bekalu Tadesse Moges

Academic Editor

PLOS One

Journal Requirements:

1. If the reviewer comments include a recommendation to cite specific previously published works, please review and evaluate these publications to determine whether they are relevant and should be cited. There is no requirement to cite these works unless the editor has indicated otherwise.

2. Please review your reference list to ensure that it is complete and correct. If you have cited papers that have been retracted, please include the rationale for doing so in the manuscript text, or remove these references and replace them with relevant current references. Any changes to the reference list should be mentioned in the rebuttal letter that accompanies your revised manuscript. If you need to cite a retracted article, indicate the article’s retracted status in the References list and also include a citation and full reference for the retraction notice.

[Note: HTML markup is below. Please do not edit.]

Reviewers' comments:

Reviewer's Responses to Questions

**Comments to the Author**

Reviewer #5: All comments have been addressed

Reviewer #6: (No Response)

**********

2. Is the manuscript technically sound, and do the data support the conclusions?

Reviewer #5: Yes

Reviewer #6: Partly

**********

3. Has the statistical analysis been performed appropriately and rigorously?

Reviewer #5: Yes

Reviewer #6: Yes

**********

4. Have the authors made all data underlying the findings in their manuscript fully available?

The PLOS Data policy

Reviewer #5: No

Reviewer #6: Yes

**********

5. Is the manuscript presented in an intelligible fashion and written in standard English?

Reviewer #5: Yes

Reviewer #6: No

**********

Reviewer #5: PLOS ONE

Determinants of instructional leadership: A survey of public secondary schools

Review Report

Recommendation: Minor Revision

This study explores instructional leadership factors in Nepalese public secondary schools, adapting Hallinger & Murphy's model through Delphi and EFA. The identification of three factors planning/managing/supervising, motivating/developing competence, and involving parents, offers a culturally relevant contribution, particularly the emergence of parental involvement as a distinct factor.

However, several minor issues require attention:

• Add references within the last 5 years.

• Add future perspective of the Research.

• Tables missing: Tables 1–3 (rotated component matrices) must be included.

• Delphi clarity: Provide panel size, selection criteria, and consensus definition.

• Data availability: The statement "Data is with authors" does not meet PLOS ONE policy. Deposit data in a public repository or justify restrictions.

• Writing quality: Numerous grammatical errors and typos need correction (e.g., "of of" in title).

• Discussion: Briefly explain why parental involvement emerged as a factor in Nepal's cultural context.

• These revisions can be completed within 2–4 weeks. The manuscript has merit but requires these improvements before acceptance.

• Recommendation:

• Minor Revision

Reviewer #6: Thank you so much for this wonderful article. Based on the previous round of review, I believe this manuscript is almost ready for publication, but must address several items as follows:

1. This paper needs more proof reading and cleaning up. Here are several noted observations:

A. 5th sentence in the abstract starts, "It indicated..." What is "it"? Maybe this sentence needs to connect to the previous sentence and combine them for it to make more sense.

B. I see "Nepali Government" and "Nepal Government". Please choose one and stick with it.

C. Pg. 7 shows an "r-cal" statistic. What is "r-cal"? It can't be a Pearson Correlation. Is it a t-statistic? Is it supposed to be "r=0.344" instead of 3.44?

D. Literature Review and Research Methods could use headings to break them down. Right now, they read a bit disjointed as paragraphs run into each other. For example, the Ethical Standards paragraph comes out of nowhere. A heading would help readability.

E. The last three paragraphs of the Literature Review begin explaining the survey measures and the methods. Those belong in the methods. Maybe a "Measures" section in the methods is where this could be situated.

F. On P. 10, second paragraph - the first and last sentences aren't necessary as they don't add anything substantive.

G. In Table 2, "SN" and "Items" run together. There appears to be room to not have to abbreviate "SN", but I could be wrong.

H. P.15, "[69, 70, 71, 72]" is missing a period.

I. Some headings italicized and followed by non-italicized headings that seem to cover the same information. Consider combining. Please use PLOS ONE-appropriate headings.

2. There are a few conceptual issues that need commented on:

A. This study is situated in needing to increase student achievement. That is fine, but this paper addresses the connections between their new scale and student achievement by generally communicating previous research that broadly connects specific items with student achievement. That is fine, but the claim that this study will increase student achievement is too much of a stretch as there wasn't any work to support criterion validity outside of the citations.

B. P. 12 states, "Thus, the reliability of factor analysis was assessed." after reporting Cronbach's alpha values.

This is inaccurate. The authors assessed the internal consistency of the items within each extracted factor.

C. P. 20 suggests the authors identified factors that influence IL. This makes it sound like there was some sort of regression or prediction. The authors identified factors that closely represent how IL is conceptualized across the included Delphi participants in Nepal.

D. The abstract says there is a need for a valid IL model in Nepal. That is the only reference to validity that I found, but if you are to keep that in the abstract, there needs to be some mention of how this work contributes to validity. This mostly belongs in the future research/limitation section since this study didn't perform CFA to support that the three-factor model fits data from Nepal schools or criterion validity outside of relating the items generally to previous research.

3. Methods

A. Why 172 randomized schools? My understanding is that this sample size is fine for this study, but why not more or less since there were more to include? Was there some sort of inclusion criteria beyond randomly selection?

**********

what does this mean?). If published, this will include your full peer review and any attached files.

If you choose “no”, your identity will remain anonymous but your review may still be made public.

**Do you want your identity to be public for this peer review?**  For information about this choice, including consent withdrawal, please see our Privacy Policy

Reviewer #5: **Yes:**  Dr. Muhammad Adnan Maqbool

Reviewer #6: No

**********

[NOTE: If reviewer comments were submitted as an attachment file, they will be attached to this email and accessible via the submission site. Please log into your account, locate the manuscript record, and check for the action link "View Attachments". If this link does not appear, there are no attachment files.]

To ensure your figures meet our technical requirements, please review our figure guidelines: https://journals.plos.org/plosone/s/figures

You may also use PLOS’s free figure tool, NAAS, to help you prepare publication quality figures: https://journals.plos.org/plosone/s/figures#loc-tools-for-figure-preparation.

NAAS will assess whether your figures meet our technical requirements by comparing each figure against our figure specifications.

Attachment Submitted filename: REVIEW REPORT-PLOS ONE.docx

# Author comment: Author response to Decision Letter 4

**DOI:** 10.1371/journal.pone.0355674.r008

21 Jul 2026

1. Add references within last 5 years

Reference numbers 31, 32, 33, 34 & 35 are added

31. Hallinger, P., Liu, S., Aung, P. N., & Yan, M. (2025). A systematic review of instructional leadership research conducted with the principal instructional Management Rating Scale (PIMRS), 1983–2024. Educational Management Administration & Leadership. Advance online publication. https://doi.org/10.1177/17411432251358575

32. Guo, H., Alias, B. S., & Mamud, M. I. (2025). Interplay of principal instructional leadership, school organizational climate, and teacher job satisfaction: Evidence from secondary schools in Northwest China. International Journal of Learning, Teaching and Educational Research, 24(4). https://doi.org/10.26803/ijlter.24.4.9

33. Kusumaningrum, D. E., Gunawan, I., Sumarsono, R. B., & Latifah, R. (2025). Exploring how change leadership influences instructional leadership effects on school culture and teachers’ teaching performance in pesantren education settings in Indonesia: A moderated-mediation analysis. Journal on Efficiency and Responsibility in Education and Science, 18(2), 100–111. https://doi.org/10.7160/eriesj.2025.180204

34. Dorukbaşi, E., & Cansoy, R. (2024). Examining the mediating role of teacher professional learning between perceived instructional leadership and teacher instructional practices. European Journal of Education, 59(3), e12672. https://doi.org/10.1111/ejed.12672

35. Demir, K., & Kalman, M. (2025). Instructional leadership and teachers' instructional practices: A serial mediation by teacher autonomy and collective teacher innovativeness. European Journal of Education 60(3), Article e70155. https://doi.org/10.1111/ejed.70155

2. Add future perspective of the Research

Since this study is confined to public secondary schools in Kathmandu, such a study could in the future also be conducted in institutional or public schools in other provinces for comparative analysis. Additionally, the effect of instructional leadership on students’ academic achievement can also be studied in different contexts.

3. Tables missing: Tables 1–3 (rotated component matrices) must be included.

Table showing rotated component matrices of all three factors (Factor1, Factor 2, and Factor 3) are there in the result section.

4. Delphi clarity: Provide panel size, selection criteria, and consensus definition.

The literature lacks consensus on the exact number of experts in the Delphi process [62]. However, following [63], a total of 20 panelists, one teacher, and a head teacher from 10 schools across diverse areas of Kathmandu were selected for the Delphi process. The criteria for their selection as experts in instructional leadership were more than five years of experience in the teaching-learning process in public secondary schools and their professional expertise as head teachers or teachers. The consensus in the Delphi process was measured as percent agreement, and the cut-off value in this study was 75%. It is in line with [62], which, on the basis of a review study, suggested that there is no widely accepted cut-off value (the value from which consensus is assumed) for defining consensus. It varies considerably; however, a threshold of 60% or higher was identified in most cases.

5. Data availability: The statement "Data is with authors" does not meet PLOS ONE policy. Deposit data in a public repository or justify restrictions.

Data file is submitted along with other files with the file name S1_dataset. We have no problem to upload data to public repository. Actually, we do not have idea on that online data- storage platform where we should upload that.

6. Writing quality: Numerous grammatical errors and typos need correction (e.g., "of of" in title).

Correction is done thoroughly.

7. Briefly explain why parental involvement emerged as a factor in Nepal's cultural context.

In Nepal, there is a family-oriented society, and within the family, there is a culture of obedience to parents/guardians. Parents/guardians instill values such as discipline, respect, hard work, and responsibility in their children. Such guidance and care affect children’s learning habits and achievement. Parents/guardians take an interest in monitoring their children’s academic performance and solving their problems. Therefore, parents’/guardians’ involvement emerged as a factor in Nepal’s cultural and societal context.

8. 5th sentence in the abstract starts, "It indicated..." What is "it"? Maybe this sentence needs to connect to the previous sentence and combine them for it to make more sense.

The existing instructional leadership models are primarily Western and not entirely suitable for implementation in Nepal. Therefore, it seemed essential to develop a model by identifying the determinants of instructional leadership in Nepal’s local context.

9. I see "Nepali Government" and "Nepal Government". Please choose one and stick with it.

It is made Nepal Government.

10. Pg. 7 shows an "r-cal" statistic. What is "r-cal"? It can't be a Pearson Correlation. Is it a t-statistic? Is it supposed to be "r=0.344" instead of 3.44?

Yes, these are t-values and could correspond to significant correlations at p < 0.05. We removed values from the sentence because the statement gives complete sense even without mentioning these values.

11. Literature Review and Research Methods could use headings to break them down. Right now, they read a bit disjointed as paragraphs run into each other. For example, the Ethical Standards paragraph comes out of nowhere. A heading would help readability.

Sub- headings are added in both literature review, and research methods sections .

12. The last three paragraphs of the Literature Review begin explaining the survey measures and the methods. Those belong in the methods. Maybe a "Measures" section in the methods is where this could be situated.

Arranged accordingly.

13. On P. 10, second paragraph - the first and last sentences aren't necessary as they don't add anything substantive.

Those two sentences are removed.

14. In Table 2, "SN" and "Items" run together. There appears to be room to not have to abbreviate "SN", but I could be wrong.

It has been corrected.

15. P.15, "[69, 70, 71, 72]" is missing a period.

Full stop is added.

Attachment Submitted filename: Response_to_Reviewers_auresp_4.docx

# Editor report: Decision Letter 4

**DOI:** 10.1371/journal.pone.0355674.r009

**Authors:** Bekalu Tadesse Moges

27 Jul 2026

<p>Determinants of instructional leadership: A survey of public secondary schools

PONE-D-25-14755R4

Dear Dr. Suman Pande,

We’re pleased to inform you that your manuscript has been judged scientifically suitable for publication and will be formally accepted for publication once it meets all outstanding technical requirements.

Within one week, you’ll receive an e-mail detailing the required amendments. When these have been addressed, you’ll receive a formal acceptance letter and your manuscript will be scheduled for publication.

An invoice will be generated when your article is formally accepted. Please note, if your institution has a publishing partnership with PLOS and your article meets the relevant criteria, all or part of your publication costs will be covered. Please make sure your user information is up-to-date by logging into Editorial Manager at Editorial Manager® and clicking the ‘Update My Information' link at the top of the page. For questions related to billing, please contact billing support.

If your institution or institutions have a press office, please notify them about your upcoming paper to help maximize its impact. If they’ll be preparing press materials, please inform our press team as soon as possible -- no later than 48 hours after receiving the formal acceptance. Your manuscript will remain under strict press embargo until 2 pm Eastern Time on the date of publication. For more information, please contact onepress@plos.org.

Kind regards,

Bekalu Tadesse Moges

Academic Editor

PLOS One

Additional Editor Comments (optional):

Reviewers' comments:

# Editor report: Acceptance letter

**DOI:** 10.1371/journal.pone.0355674.r010

**Authors:** Bekalu Tadesse Moges

PONE-D-25-14755R4

PLOS One

Dear Dr. Pande,

I'm pleased to inform you that your manuscript has been deemed suitable for publication in PLOS One. Congratulations! Your manuscript is now being handed over to our production team.

At this stage, our production department will prepare your paper for publication. This includes ensuring the following:

* All references, tables, and figures are properly cited

* All relevant supporting information is included in the manuscript submission,

* There are no issues that prevent the paper from being properly typeset

You will receive further instructions from the production team, including instructions on how to review your proof when it is ready. Please keep in mind that we are working through a large volume of accepted articles, so please give us a few days to review your paper and let you know the next and final steps.

Lastly, if your institution or institutions have a press office, please let them know about your upcoming paper now to help maximize its impact. If they'll be preparing press materials, please inform our press team within the next 48 hours. Your manuscript will remain under strict press embargo until 2 pm Eastern Time on the date of publication. For more information, please contact onepress@plos.org.

You will receive an invoice from PLOS for your publication fee after your manuscript has reached the completed accept phase. If you receive an email requesting payment before acceptance or for any other service, this may be a phishing scheme. Learn how to identify phishing emails and protect your accounts at https://explore.plos.org/phishing.

If we can help with anything else, please email us at customercare@plos.org.

Thank you for submitting your work to PLOS ONE and supporting open access.

Kind regards,

PLOS ONE Editorial Office Staff

on behalf of

Dr. Bekalu Tadesse Moges

Academic Editor

PLOS One
